# Supplementary material for: Tissue-location-specific transcription programs drive tumor dependencies in colon cancer
Source: Nat Commun. 2024 Feb 15;15:1384. doi: 10.1038/s41467-024-45605-4 (PMC10869357; doi:10.1038/s41467-024-45605-4)
Supplement: Supplementary file 1 — Supplementary Information [file 41467_2024_45605_MOESM1_ESM.pdf]

Tissue-location specific transcription programs drive tumor dependencies in colon cancer

Lijing Yang,[1,2,6] Lei Tu,[3,6] Shilpa Bisht,[1] Yiqing Mao,[1] Daniel Petkovich,[1] Sara-Jayne Thursby,[1] Jinxiao Liang, [1] Nibedita Patel,[1] Ray-Whay Chiu Yen,[1] Tina Largent,[1] Cynthia Zahnow,[1] Malcolm Brock,[1] Kathy Gabrielson,[4] Kevan J. Salimian,[5] Stephen B. Baylin,[1] Hariharan Easwaran[1,*]

Contents	Pages
Supplementary Figures and Figure legends	2 to 19
Supplementary Tables	20 to 27
Raw Data for Supplemental Figures	28 to 32
Supplementary References	33 to 34

Supplementary materials: 7 Figures; 4 Tables.

Supplementary Figures and Figure legends

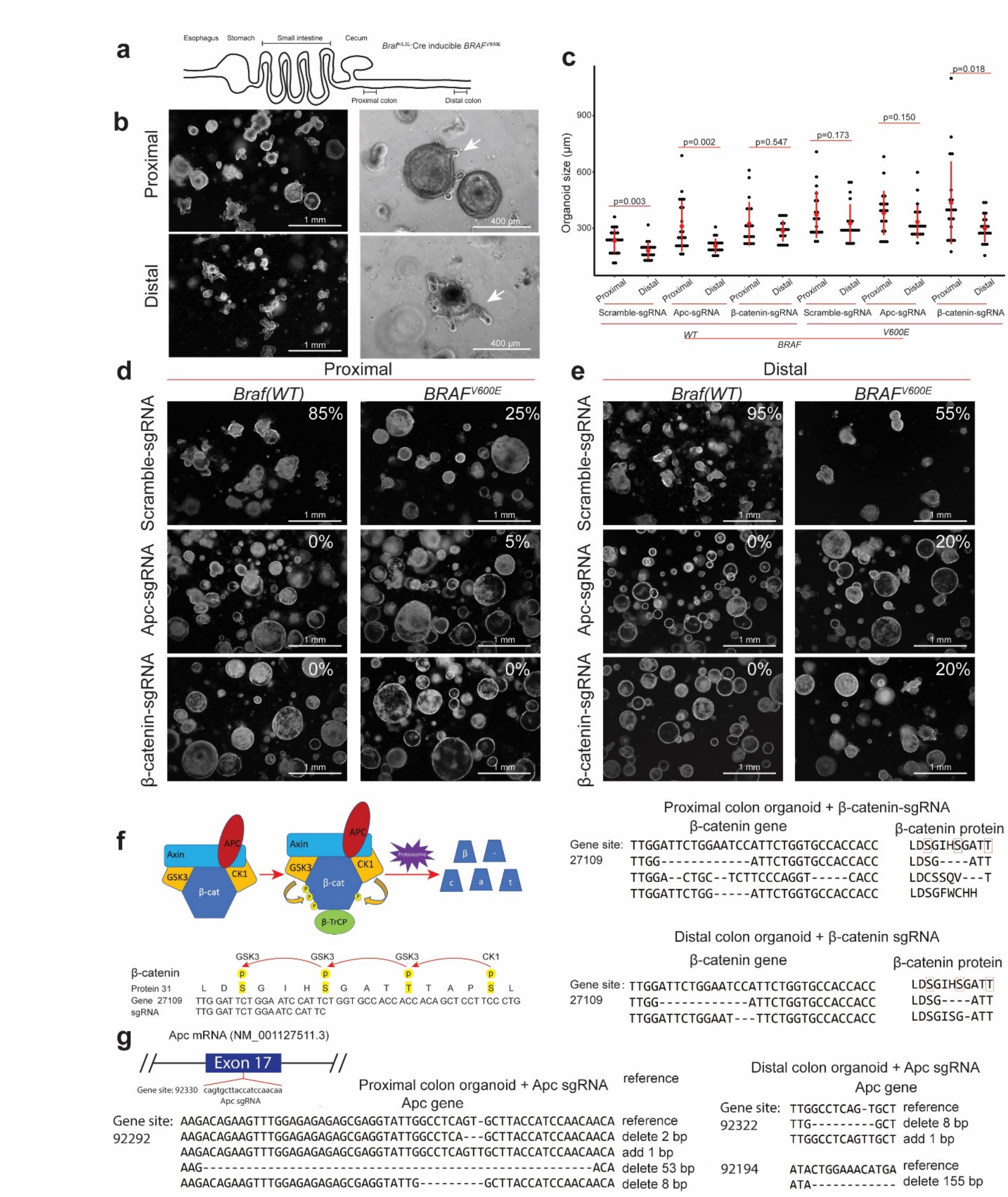

Supplementary Figure 1. Design of targeted mutations in *Apc* and *Ctnnb1* (b-catenin) and morphology of proximal and distal colon organoids upon abnormal Wnt-pathway activation, related to Figure 1.

(A) Schematic representation of the anatomic location of *Braf*$^{+/V600E\_LSL}$ loci containing mouse from which proximal and distal colon organoids were isolated.

(B) Representative images of proximal colon organoid (up) growing into larger structures with small buds (white arrow), while distal colon organoids (down) with more budded protrusions (white arrow) (growth assay data are representative of three biological replicates) (scale bar for left panel = 1mm; scale bar for right panel = 400 μm).

(C) Dot plot showing the size (long plus short diameter divided by 2) of proximal and distal colon organoids edited by Apc-sgRNA or β-Catenin-sgRNA, with or without induction of *BRAF*^{V600E}. Error bars indicate means ± SD. Two-sided Wilcoxon rank sum exact test (n=20 organoids analyzed).

(D and E) Representative images of proximal (D) and distal (E) colon organoids edited by Apc-sgRNA or b-catenin-sgRNA, with or without induction of *BRAF*^{V600E}. Percentage organoids with budded structures shown on top right. Organoid growth in D and E are representative of two biological replicates (scale bar = 1mm for all panels).

(F) Schematic representation of b-catenin destruction complex which can phosphorylate four of the N-terminal amino acids of b-catenin protein step by step (left). Subsequently the phosphorylated b-catenin is lysed by proteasome. sgRNA targeting the exon 1 of *Ctnnb1* gene encoding b-catenin resulted in deletion of the phosphorylation site and thus blocking the lysis process stimulated by b-catenin destruction complex, followed by the abnormal continuous activation of Wnt-pathway. Sanger sequence results in the right panel shows the resulting mutations and the corresponding amino acid sequence in proximal and distal organoids.

(G) Similar to F, sgRNA targeting exon 17 in *Apc* and the resulting mutations and the corresponding amino acid sequence in proximal and distal organoids.

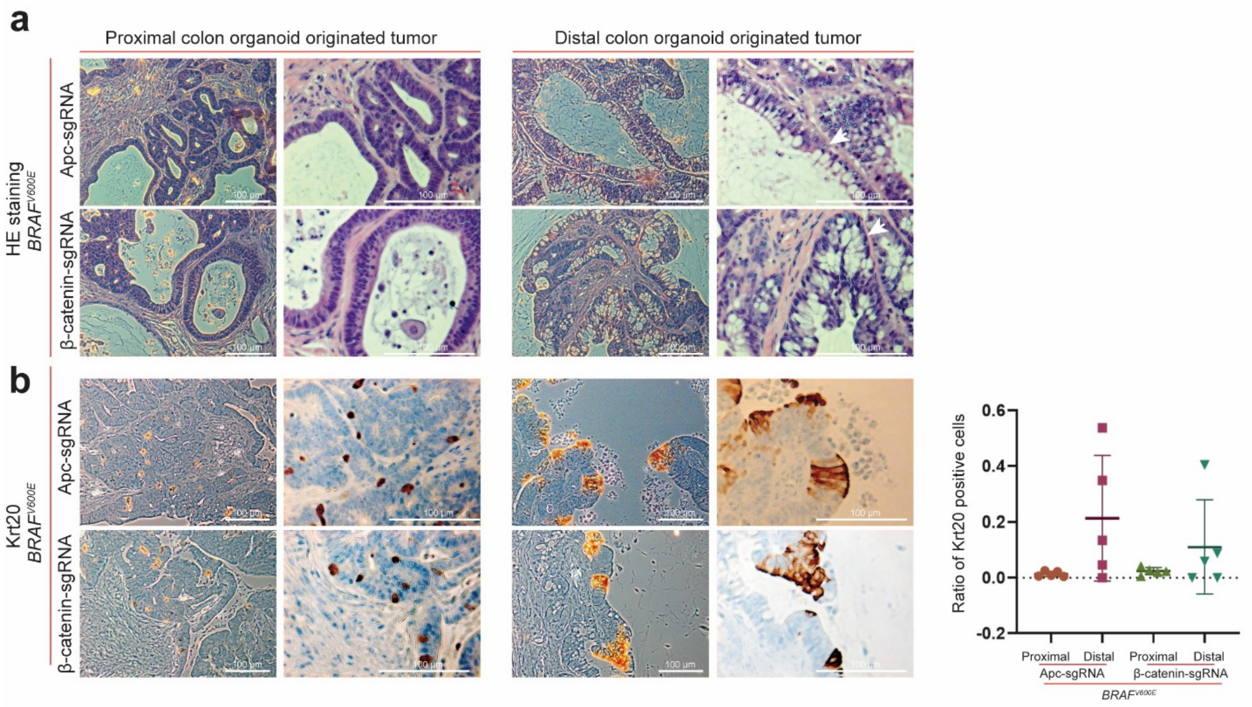
a
Proximal colon organoid originated tumor
Distal colon organoid originated tumor
HE staining
BRAF^V600E
Apc-sgRNA
β-catenin-sgRNA
100 μm
b
Krt20
BRAF^V600E
Apc-sgRNA
β-catenin-sgRNA
100 μm
Ratio of Krt20 positive cells
0.6
0.4
0.2
0.0
-0.2
Proximal
Distal
Proximal
Distal
Apc-sgRNA
β-catenin-sgRNA
BRAF^V600E

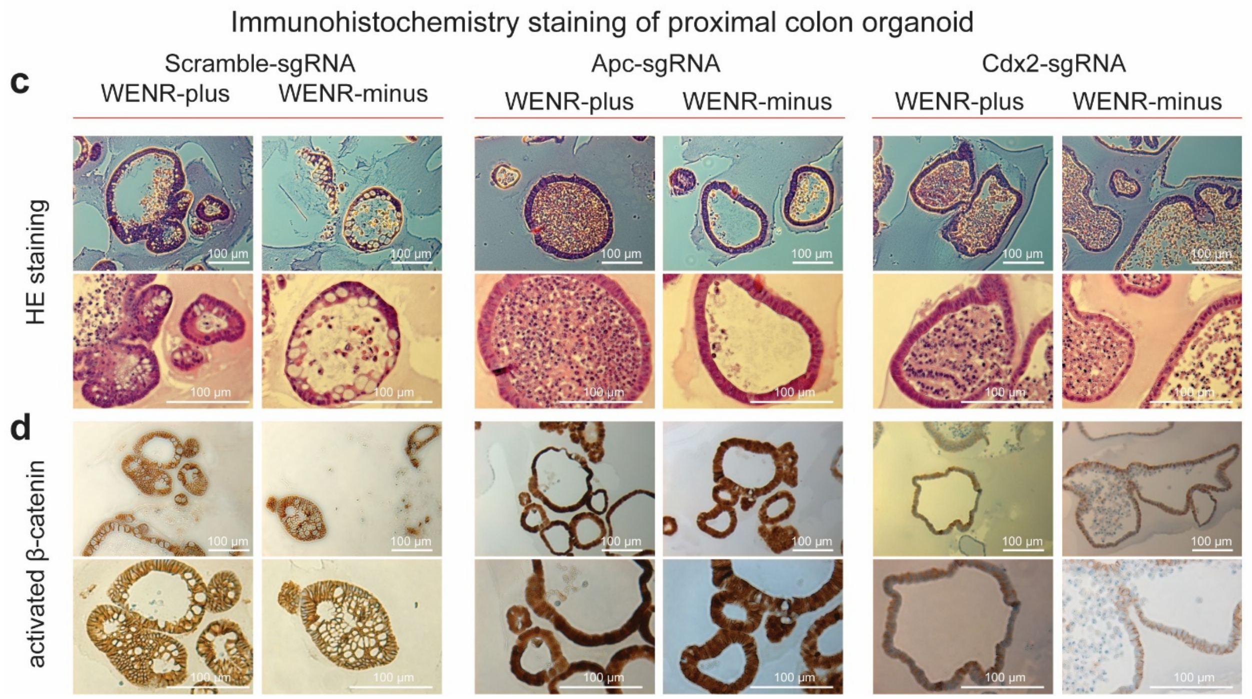
Immunohistochemistry staining of proximal colon organoid
c
Scramble-sgRNA
WENR-plus
WENR-minus
Apc-sgRNA
WENR-plus
WENR-minus
Cdx2-sgRNA
WENR-plus
WENR-minus
HE staining
100 μm
d
activated β-catenin
100 μm

Supplementary Figure 2. Proximal and distal colon stem cells show differences in tumors driven by *$BRAF^{V600E}$* in the context of abnormal Wnt pathway activation.

(A-B) Representative H&E (A) and immunohistochemistry staining (Krt20) (B) of tumors growing as xenografts derived from proximal (left) and distal (right) colon organoids with *$BRAF^{V600E}$* combined with *Apc* mutation or β-catenin mutation. (B, right part) shows the comparison of ratio of Krt20 positive cells. Arrow head showing the vesicle structure of tumor cells derived from distal colon organoids. H&E are representative of tumors from two xenograft replicates. Five areas of the H&E images were used for counting the ratio of Krt20 positive cells. Scale bar = 100 μm for all panels.

(C) Representative H&E staining of proximal colon organoids with Scramble-sgRNA, Apc-sgRNA and Cdx2-sgRNA separately cultured in WENR-plus or -minus medium. Images are derived from organoid from one mouse. Scale bar = 100 μm for all panels.

(D) Representative immunohistochemistry staining using activated b-catenin (D) antibody separately for proximal colon organoid treated with Scramble-sgRNA, APC-sgRNA, and Cdx2-sgRNA separately cultured in WENR-plus or -minus medium. Images are derived from organoid from one mouse. Scale bar = 100 μm for all panels.

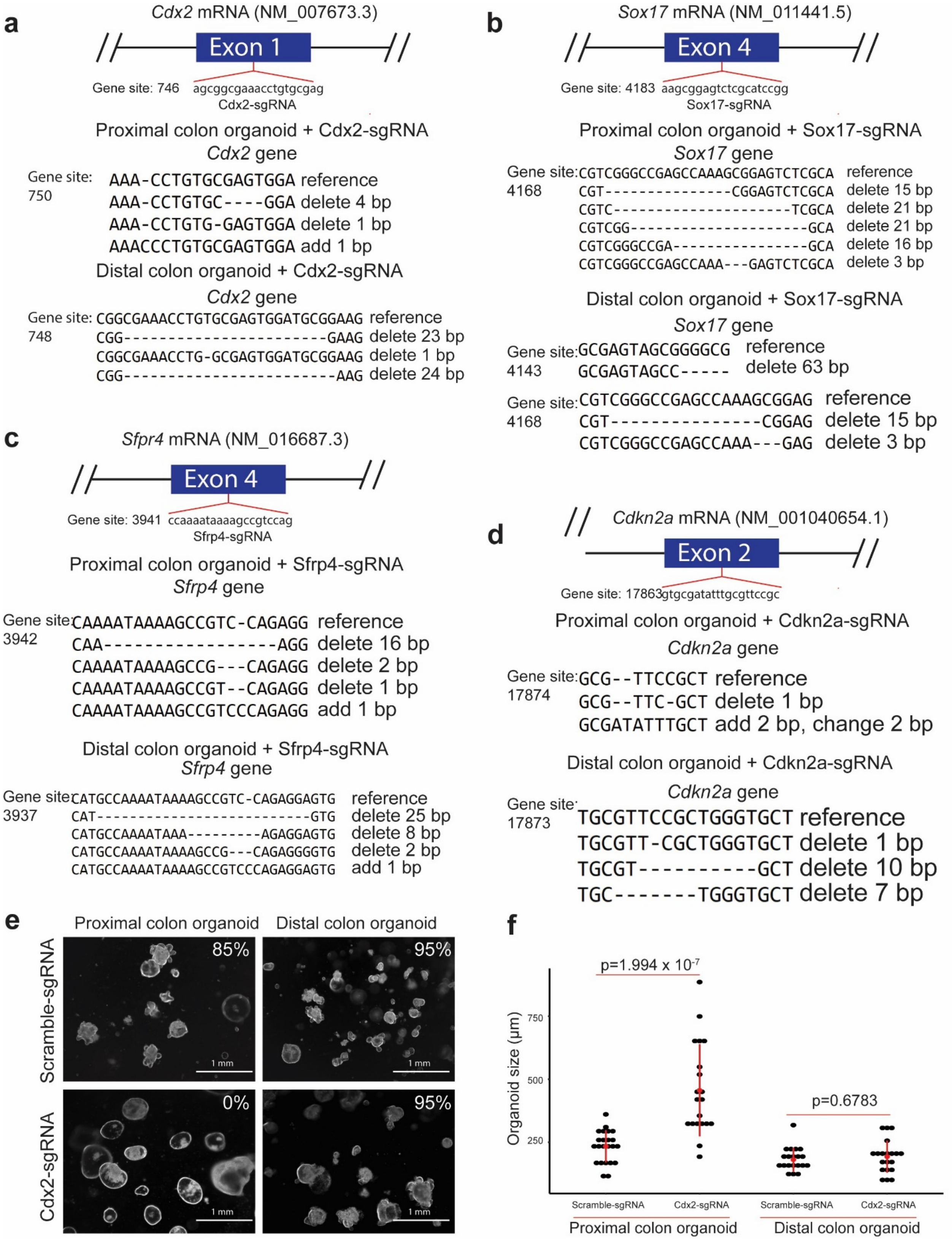
a
Cdx2 mRNA (NM_007673.3)
Exon 1
Gene site: 746 agcggcgaaacctgtgcgag
Cdx2-sgRNA
Proximal colon organoid + Cdx2-sgRNA
Cdx2 gene
Gene site: 750
AAA-CCTGTGCGAGTGGA reference
AAA-CCTGTGC----GGA delete 4 bp
AAA-CCTGTG-GAGTGGA delete 1 bp
AAACCCTGTGCGAGTGGA add 1 bp
Distal colon organoid + Cdx2-sgRNA
Cdx2 gene
Gene site: 748
CGGCGAAACCTGTGCGAGTGGATGCGGAAG reference
CGG-----------------------GAAG delete 23 bp
CGGCGAAACCTG-GCGAGTGGATGCGGAAG delete 1 bp
CGG------------------------AAG delete 24 bp
b
Sox17 mRNA (NM_011441.5)
Exon 4
Gene site: 4183 aagcggagtctcgcatccgg
Sox17-sgRNA
Proximal colon organoid + Sox17-sgRNA
Sox17 gene
Gene site: 4168
CGTCGGGCCGAGCCAAAGCGGAGTCTCGCA reference
CGT---------------CGGAGTCTCGCA delete 15 bp
CGTC---------------------TCGCA delete 21 bp
CGTCGG---------------------GCA delete 21 bp
CGTCGGGCCGA----------------GCA delete 16 bp
CGTCGGGCCGAGCCAAA---GAGTCTCGCA delete 3 bp
Distal colon organoid + Sox17-sgRNA
Sox17 gene
Gene site: 4143
GCGAGTAGCGGGGCG reference
GCGAGTAGCC----- delete 63 bp
Gene site: 4168
CGTCGGGCCGAGCCAAAGCGGAG reference
CGT---------------CGGAG delete 15 bp
CGTCGGGCCGAGCCAAA---GAG delete 3 bp
c
Sfpr4 mRNA (NM_016687.3)
Exon 4
Gene site: 3941 ccaaaataaaagccgtccag
Sfrp4-sgRNA
Proximal colon organoid + Sfrp4-sgRNA
Sfrp4 gene
Gene site: 3942
CAAAATAAAAGCCGTC-CAGAGG reference
CAA-----------------AGG delete 16 bp
CAAAATAAAAGCCG---CAGAGG delete 2 bp
CAAAATAAAAGCCGT--CAGAGG delete 1 bp
CAAAATAAAAGCCGTCCCAGAGG add 1 bp
Distal colon organoid + Sfrp4-sgRNA
Sfrp4 gene
Gene site: 3937
CATGCCAAAATAAAAGCCGTC-CAGAGGAGTG reference
CAT-------------------------GTG delete 25 bp
CATGCCAAAATAAA---------AGAGGAGTG delete 8 bp
CATGCCAAAATAAAAGCCG---CAGAGGGGTG delete 2 bp
CATGCCAAAATAAAAGCCGTCCCAGAGGAGTG add 1 bp
d
Cdkn2a mRNA (NM_001040654.1)
Exon 2
Gene site: 17863 gtgcgatatttgcgttccgc
Proximal colon organoid + Cdkn2a-sgRNA
Cdkn2a gene
Gene site: 17874
GCG--TTCCGCT reference
GCG--TTC-GCT delete 1 bp
GCGATATTTGCT add 2 bp, change 2 bp
Distal colon organoid + Cdkn2a-sgRNA
Cdkn2a gene
Gene site: 17873
TGCGTTCCGCTGGGTGCT reference
TGCGTT-CGCTGGGTGCT delete 1 bp
TGCGT----------GCT delete 10 bp
TGC-------TGGGTGCT delete 7 bp
e
Proximal colon organoid
Distal colon organoid
Scramble-sgRNA
Cdx2-sgRNA
85%
95%
0%
95%
1 mm
f
p=1.994 x 10-7
p=0.6783
Organoid size (μm)
750
500
250
Scramble-sgRNA
Cdx2-sgRNA
Scramble-sgRNA
Cdx2-sgRNA
Proximal colon organoid
Distal colon organoid

Supplementary Figure 3. Validation of CRISPR/Cas9 mediated targeted gene mutation in the proximal and distal colon organoids transduced with Cdx2-sgRNA and gross morphological differences in the corresponding organoids.

(A-D) Schematic diagrams of the sequence for Cdx2-sgRNA (A), Sox17-sgRNA (B), Sfrp4-sgRNA (C) and Cdkn2a-sgRNA (D), and Sanger sequencing results showing corresponding gene mutations in the selected organoids.

(E) Representative images of the proximal and distal colon organoids with Scramble-sgRNA and Cdx2-sgRNA separately. Percentage organoids with budded structures shown on top right. Representative images are from organoids from three mice (scale bar = 1 mm for all panels).

(F) Dot plot showing the size (long plus short diameter divided by 2) of proximal and distal colon organoids edited by Cdx2-sgRNA. P-values calculated using two-sided Wilcoxon rank sum exact test. Error bars indicate means ± SD. N=20 organoids in each condition were counted for analyzing organoid size.

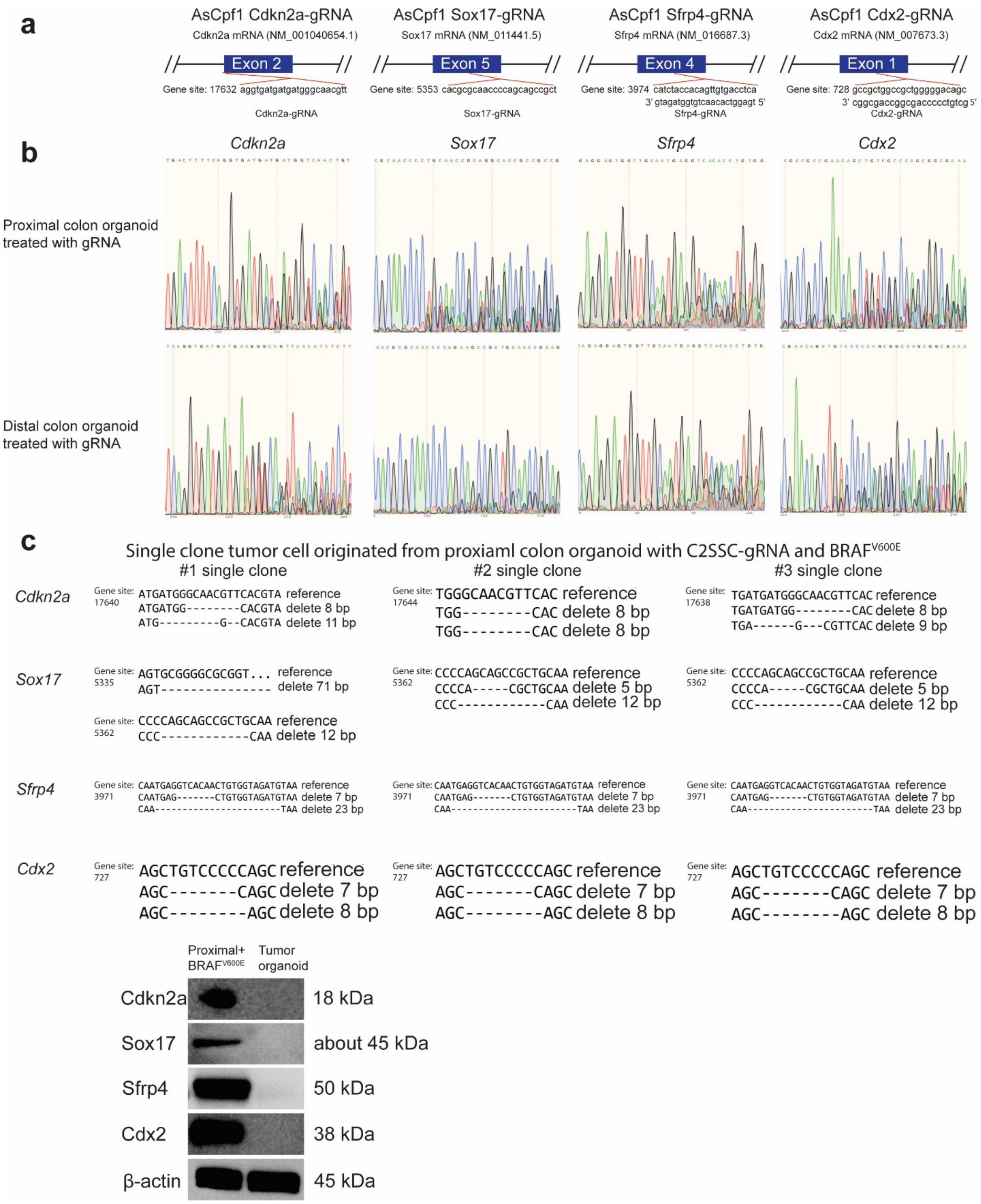

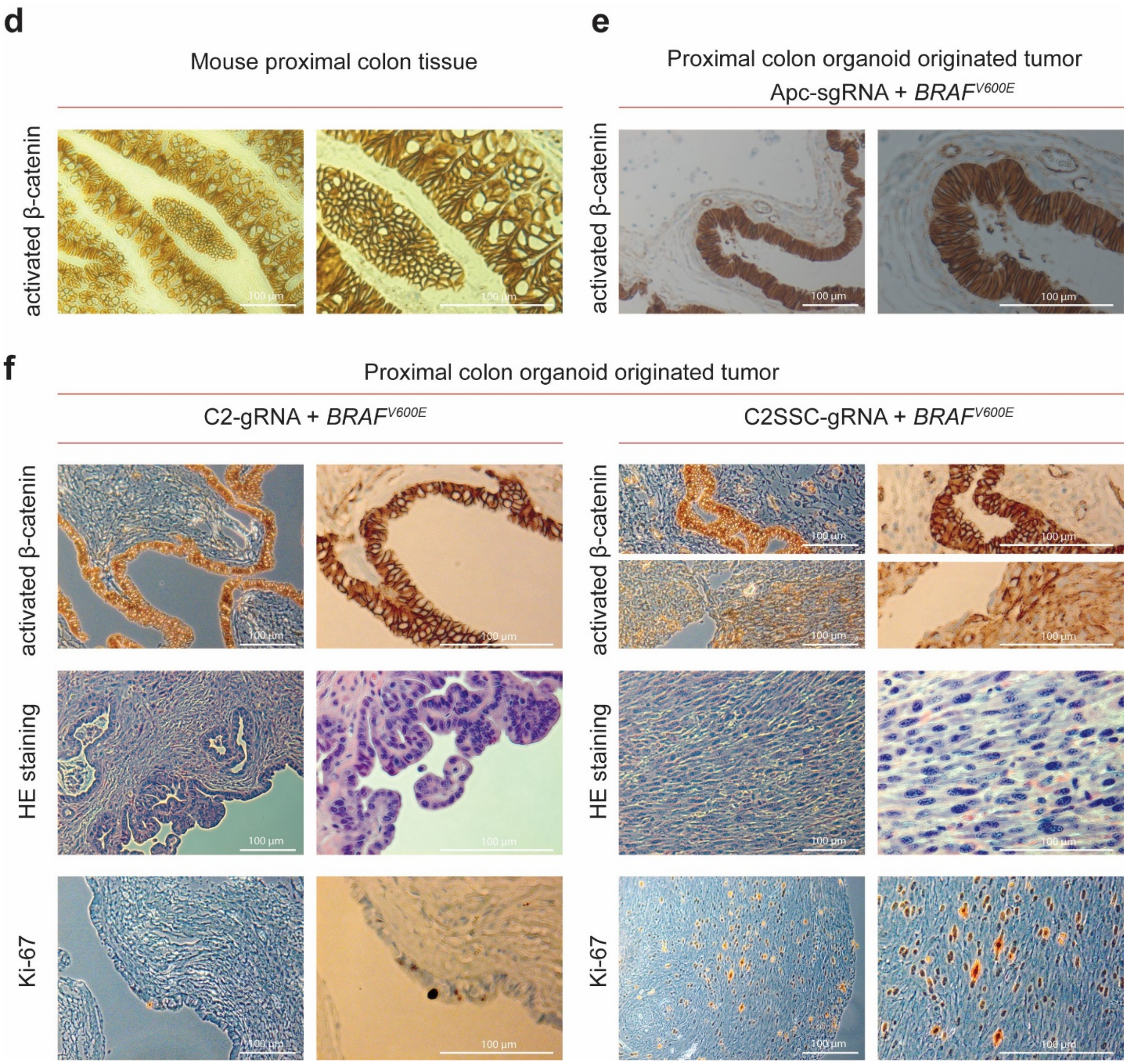

Supplementary Figure 4. Design and validation of *Cdkn2a*, *Sox17*, *Sfrp4*, and *Cdx2* guide RNAs for CRISPR-AsCpf1 approach, and tumors derived from proximal colon organoids in the context of combination of *Cdx2* mutation and $BRAF^{V600E}$ do not show canonical Wnt-activation.

(A) Schematic diagrams showing the exon in *Cdkn2a*, *Sox17*, *Sfrp4*, and *Cdx2* targeted by corresponding guide RNAs using CRISPR-Ascpf1.

(B) Sanger sequence results checking efficiency of gene editing of Cdkn2a-gRNA, Sox17-gRNA, Sfrp4-gRNA and Cdx2-gRNA in proximal and distal colon organoids.

(C) Sanger sequencing results showing the mutations in *Cdkn2a*, *Sox17*, *Sfrp4* and *Cdx2* in three of single cell derived organoids that were generated from the tumor cells derived from proximal colon organoids edited with C2SSC-gRNA in the context of $BRAF^{V600E}$. Western-blot results (bottom panel) showing Cdkn2a, Sox17, Sfrp4 and Cdx2 are abolished in #1 single clone tumor cell derived organoids, while positive in proximal colon organoid with $\mathrm{BRAF}^{\mathrm{V600E}}$.

(D) Representative immunohistochemistry staining using activated β-catenin antibody in mouse proximal colon tissue. Representative image from two mice (scale bar = 100 μm).

(E) Representative immunohistochemistry staining using activated β-catenin antibody in tumor derived from proximal colon organoid with *Apc* mutation in the context of $BRAF^{V600E}$. Representative image of tumors derived from organoids from two mice (scale bar = 100 μm).

(F) Representative H&E staining and immunohistochemistry (activated b-catenin, Ki-67) images of the xenograft implants from proximal colon organoids edited with C2-gRNA (forming nodular growth at implanted site) or with C2SSC-gRNA (forming tumor growth at implanted site) in the context of $BRAF^{V600E}$. Representative image of tumors derived from organoids from two mice (scale bar = 100 μm for all the images).

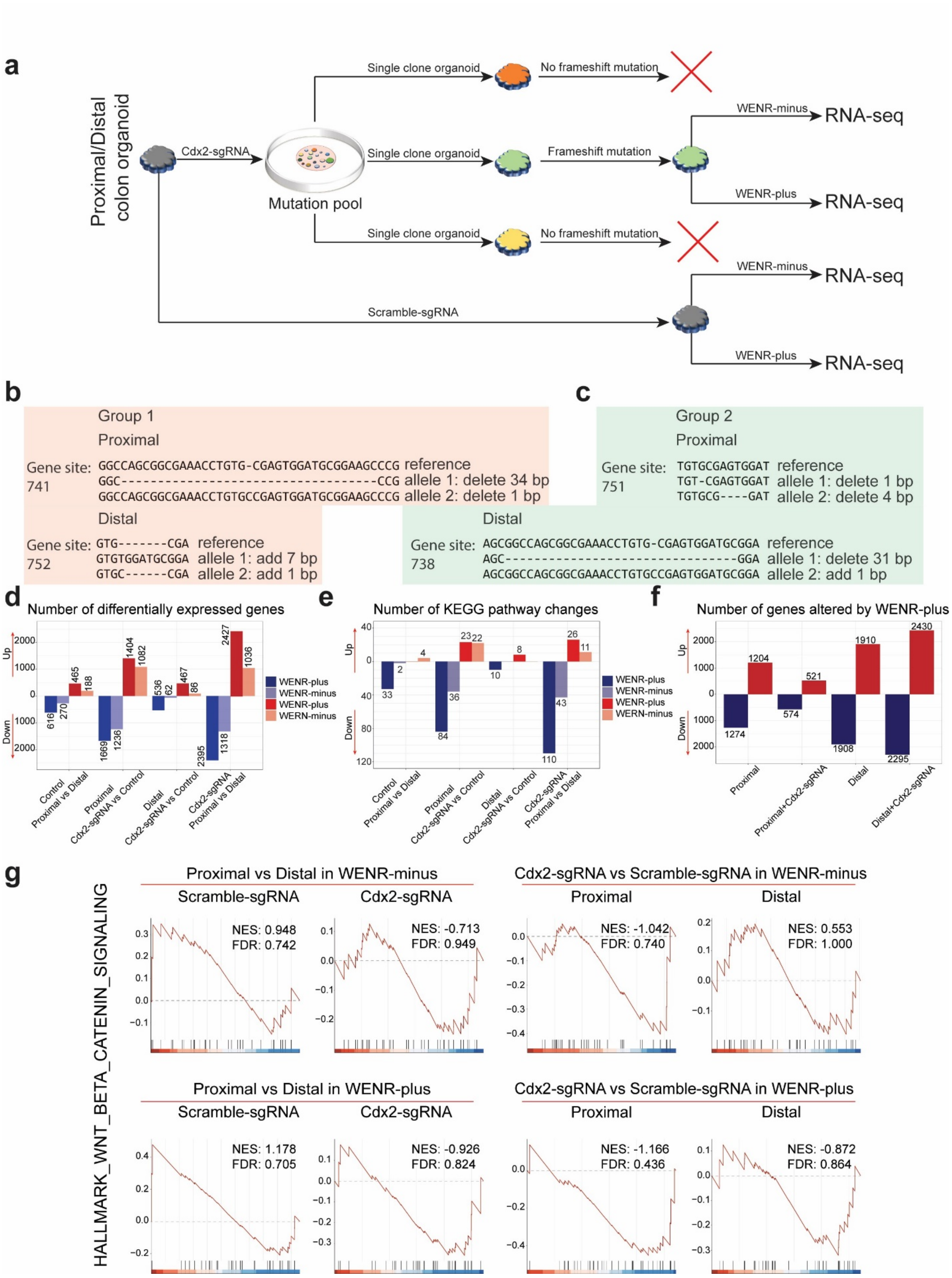
a
Proximal/Distal colon organoid
Cdx2-sgRNA
Mutation pool
Single clone organoid
No frameshift mutation
Frameshift mutation
WENR-minus
WENR-plus
RNA-seq
Scramble-sgRNA
b
Group 1
Proximal
Gene site: 741
GGCCAGCGGCGAAACCTGTG-CGAGTGGATGCGGAAGCCCG reference
GGC------------------------------------CCG allele 1: delete 34 bp
GGCCAGCGGCGAAACCTGTGCCGAGTGGATGCGGAAGCCCG allele 2: delete 1 bp
Distal
Gene site: 752
GTG-------CGA reference
GTGTGGATGCGGA allele 1: add 7 bp
GTGC------CGA allele 2: add 1 bp
c
Group 2
Proximal
Gene site: 751
TGTGCGAGTGGAT reference
TGT-CGAGTGGAT allele 1: delete 1 bp
TGTGCG----GAT allele 2: delete 4 bp
Distal
Gene site: 738
AGCGGCCAGCGGCGAAACCTGTG-CGAGTGGATGCGGA reference
AGC-------------------------------GGA allele 1: delete 31 bp
AGCGGCCAGCGGCGAAACCTGTGCCGAGTGGATGCGGA allele 2: add 1 bp
d
Number of differentially expressed genes
Up
Down
WENR-plus
WENR-minus
WENR-plus
WERN-minus
Control Proximal vs Distal
Proximal Cdx2-sgRNA vs Control
Distal Cdx2-sgRNA vs Control
Cdx2-sgRNA Proximal vs Distal
e
Number of KEGG pathway changes
f
Number of genes altered by WENR-plus
Proximal
Proximal+Cdx2-sgRNA
Distal
Distal+Cdx2-sgRNA
g
HALLMARK_WNT_BETA_CATENIN_SIGNALING
Proximal vs Distal in WENR-minus
Scramble-sgRNA
Cdx2-sgRNA
NES: 0.948 FDR: 0.742
NES: -0.713 FDR: 0.949
Cdx2-sgRNA vs Scramble-sgRNA in WENR-minus
Proximal
Distal
NES: -1.042 FDR: 0.740
NES: 0.553 FDR: 1.000
Proximal vs Distal in WENR-plus
NES: 1.178 FDR: 0.705
NES: -0.926 FDR: 0.824
Cdx2-sgRNA vs Scramble-sgRNA in WENR-plus
NES: -1.166 FDR: 0.436
NES: -0.872 FDR: 0.864

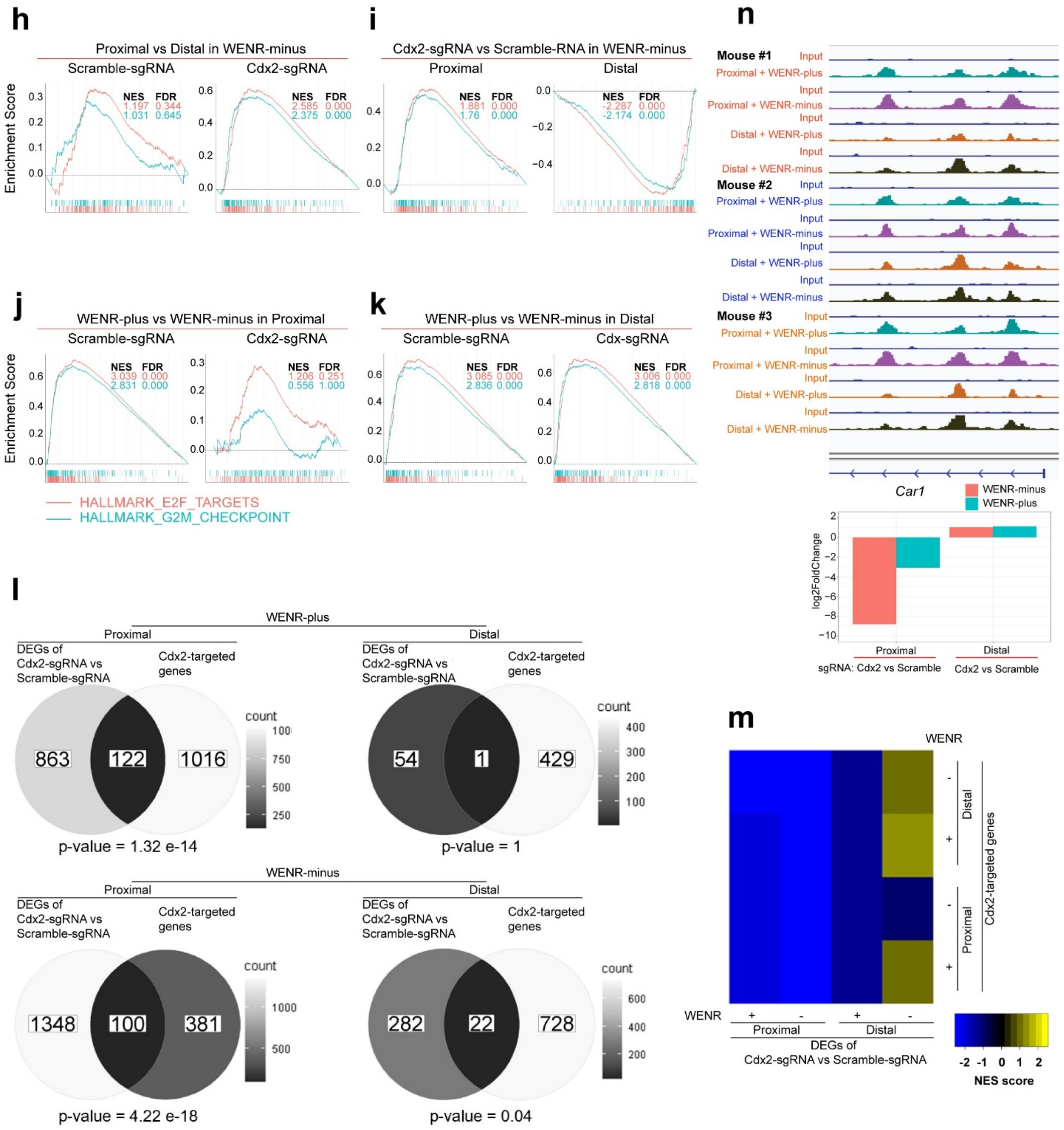

Supplementary Figure 5. Gene expression analysis for Cdx2-loss and -targeted genes.

(A) Schematic of sample collection.

(B-C) Sanger sequence results of the biological replicates (independent mice) of proximal and distal colon organoids used for the RNA-seq.

(D) Number of genes with altered expression after *Cdx2* loss (Cdx2-sgRNA).

(E) Number of altered KEGG pathways altered upon Cdx2 loss. Pathways were corrected for multiple hypotheses using BH-method. KEGG pathway enrichment analyses data is available in the Source Data as file Supplementary Figure 5e data.xlsx.

(F) Number of genes with altered expression upon *Cdx2* loss (Cdx2-sgRNA) in the organoids transferred from WENR-plus to -minus medium.

(G) GSEA results of Wnt pathway in organoids with *Cdx2* loss.

(H-K) GSEA results for E2F targets and G2M in organoids with *Cdx2* loss and the different comparisons listed on the top of each figure.

(L) Venn diagram showing intersection between genes differentially expressed upon Cdx2-loss and the Cdx2-targeted genes.

(M) Heat map showing Normalized Enrichment Score (NES) from GSEA analyses of gene expression data for the Cdx2-targeted genes identified in the proximal and distal organoids grown in WENR-plus and minus conditions.

(N) Top panel shows ChIP-seq enrichment traces for Cdx2-binding in the Car1 gene locus. N=3 biological replicates shown. Bottom panel shows mean log2-fold change differences in Car1 expression upon Cdx2-loss (DESeq2 analyses; n = 2 biological replicates for each comparison).

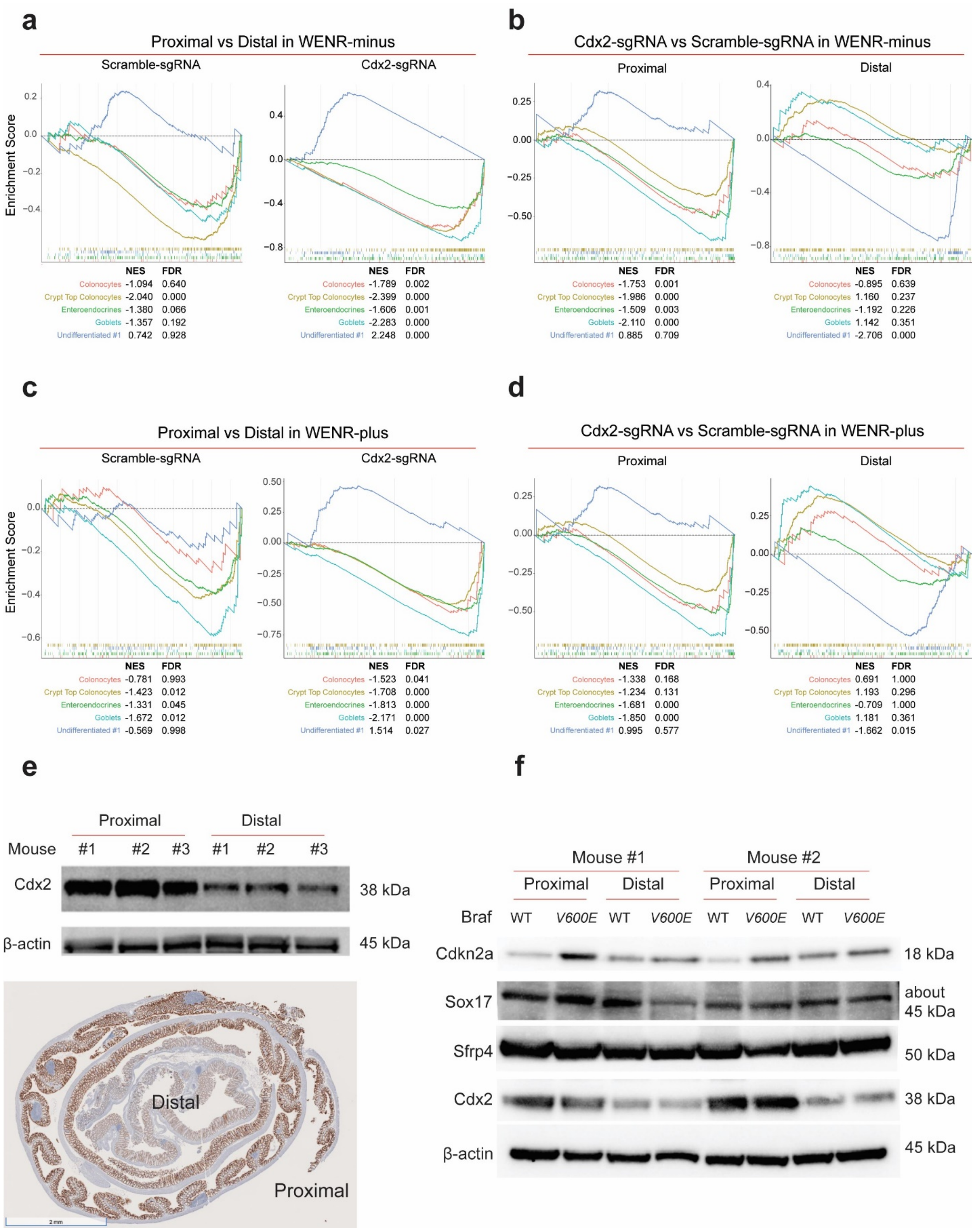
a
Proximal vs Distal in WENR-minus
Scramble-sgRNA
Cdx2-sgRNA
Enrichment Score
NES FDR
Colonocytes -1.094 0.640
Crypt Top Colonocytes -2.040 0.000
Enteroendocrines -1.380 0.066
Goblets -1.357 0.192
Undifferentiated #1 0.742 0.928
NES FDR
Colonocytes -1.789 0.002
Crypt Top Colonocytes -2.399 0.000
Enteroendocrines -1.606 0.001
Goblets -2.283 0.000
Undifferentiated #1 2.248 0.000
b
Cdx2-sgRNA vs Scramble-sgRNA in WENR-minus
Proximal
Distal
NES FDR
Colonocytes -1.753 0.001
Crypt Top Colonocytes -1.986 0.000
Enteroendocrines -1.509 0.003
Goblets -2.110 0.000
Undifferentiated #1 0.885 0.709
NES FDR
Colonocytes -0.895 0.639
Crypt Top Colonocytes 1.160 0.237
Enteroendocrines -1.192 0.226
Goblets 1.142 0.351
Undifferentiated #1 -2.706 0.000
c
Proximal vs Distal in WENR-plus
Scramble-sgRNA
Cdx2-sgRNA
Enrichment Score
NES FDR
Colonocytes -0.781 0.993
Crypt Top Colonocytes -1.423 0.012
Enteroendocrines -1.331 0.045
Goblets -1.672 0.012
Undifferentiated #1 -0.569 0.998
NES FDR
Colonocytes -1.523 0.041
Crypt Top Colonocytes -1.708 0.000
Enteroendocrines -1.813 0.000
Goblets -2.171 0.000
Undifferentiated #1 1.514 0.027
d
Cdx2-sgRNA vs Scramble-sgRNA in WENR-plus
Proximal
Distal
NES FDR
Colonocytes -1.338 0.168
Crypt Top Colonocytes -1.234 0.131
Enteroendocrines -1.681 0.000
Goblets -1.850 0.000
Undifferentiated #1 0.995 0.577
NES FDR
Colonocytes 0.691 1.000
Crypt Top Colonocytes 1.193 0.296
Enteroendocrines -0.709 1.000
Goblets 1.181 0.361
Undifferentiated #1 -1.662 0.015
e
Proximal
Distal
Mouse #1 #2 #3 #1 #2 #3
Cdx2 38 kDa
β-actin 45 kDa
Distal
Proximal
2 mm
f
Mouse #1
Mouse #2
Proximal Distal Proximal Distal
Braf WT V600E WT V600E WT V600E WT V600E
Cdkn2a 18 kDa
Sox17 about 45 kDa
Sfrp4 50 kDa
Cdx2 38 kDa
β-actin 45 kDa

Supplementary Figure 6. Cdx2 expression in proximal and distal colon regions and effects of its loss on stem and differentiated cell signatures in the proximal and distal colon organoids.

(A-D) GSEA results for gene expression signatures for the stem cell and differentiated cell types in the colon epithelium. Gene expression signatures of following cell types shown: stem cells (Undifferentiated #1) and differentiated cell types (colonocytes, crypt top colonocytes, enteroendocrine and goblet cells). (A) GSEA results from comparing gene expression in proximal organoids compared to distal organoids upon Cdx2 loss (Cdx2-sgRNA) (right panel) or control treatment (Scramble-sgRNA) (left panel) showing upregulation of stem cells (Undifferentiated #1) signature in proximal organoids with Cdx2 loss growing in WENR-minus medium. (B) GSEA results from comparing gene expression in proximal and distal organoids with Cdx2 loss to their respective controls (Scramble-sgRNA). Compared to control, Cdx2 deficient proximal organoids grown in WENR-minus medium show significant downregulation of differentiated cell signatures. In contrast, Cdx2 loss does not affect these gene signatures in distal organoids grown in WENR-minus medium, but results in downregulation of stem cell signature. (C) Same comparison as in (A) but for organoids grown in WENR-plus medium. Proximal organoids inherently show reduced signatures for differentiated cells compared to distal organoids as seen in control (Scramble-sgRNA, left panel) organoids, and these signatures are further downregulated upon Cdx2 loss (right panel). (D) Same comparison as in (B) but for organoids grown in WENR-plus medium. Cdx2 deficient proximal organoids show only marginal downregulation (NES values compared to (B)) of the differentiated cells gene signatures in the presence of Wnt-factors in medium (WENR-plus). In contrast, in distal organoids Cdx2 loss results in downregulation of stem cell signature even in the presence of Wnt-factors in medium (WENR-plus). The cell type signatures used are from previously published work Parikh et al., reference 43 in main article).

(E) Western blot (top) and immunohistochemistry staining (bottom) showing different Cdx2 expression between mouse proximal and distal colon organoid/tissue. Western blot is representative of three mice. IHC is representative of two mice (scale bar = 2mm).

(F) Cdkn2a, Sox17, Sfrp4 and Cdx2 protein expression between proximal and distal colon organoids with/without $Braf^{V600E}$. Western blot is representative of two mice.

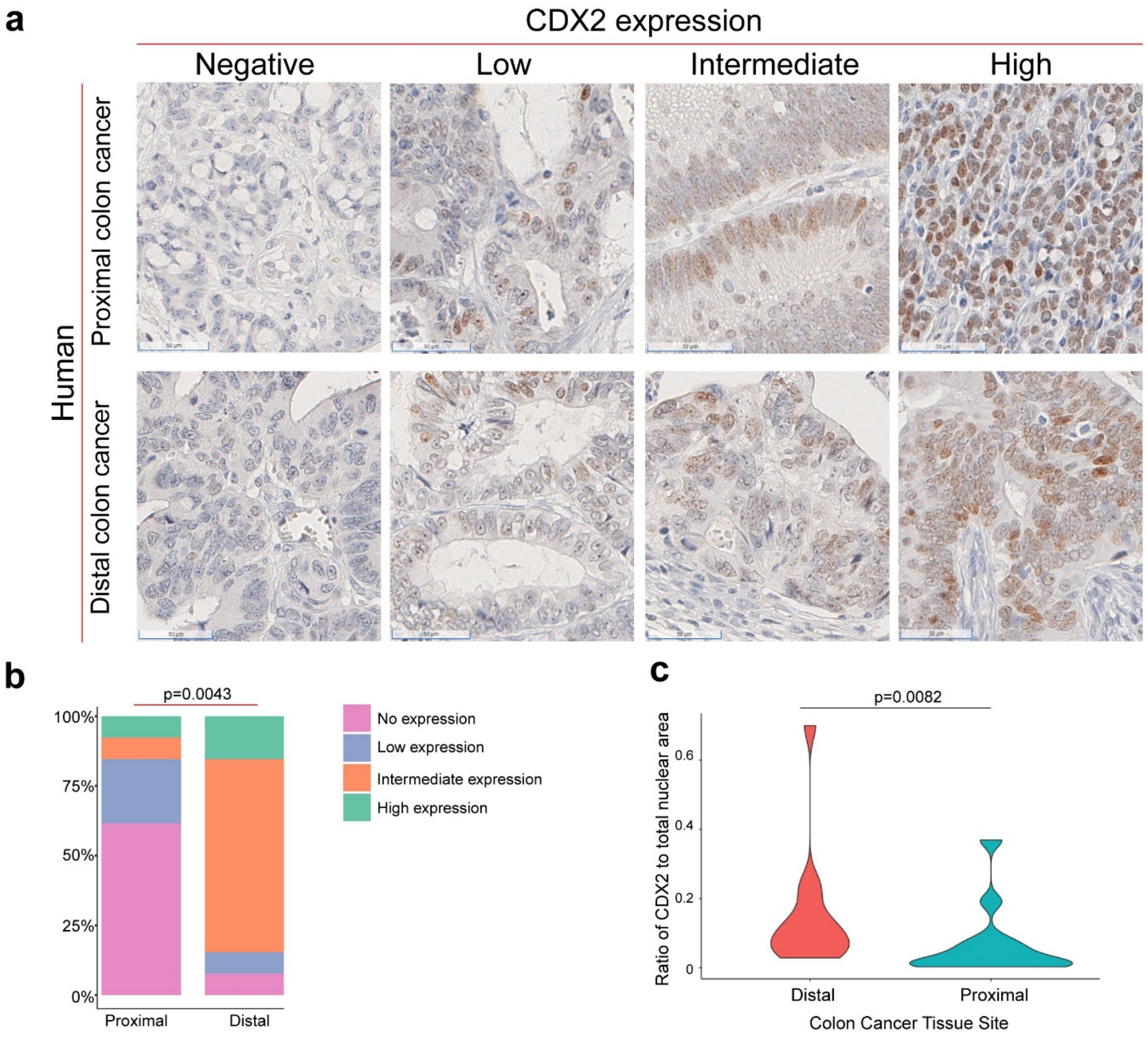

Supplementary Figure 7. Differential CDX2 expression between human proximal and distal colon cancers.

(A) Representative IHC images of CDX2 staining showing different expression intensities observed in the human proximal and distal colon cancers. Images are representative of 13 proximal and 13 distal colon cancer samples. Scale bar = 50 μm.

(B) Column bar showing the different distribution of CDX2 expression intensities in human proximal and distal colon cancer. Chi-Squared test p-values shown. 13 proximal and 13 distal colon cancer samples were analyzed.

(C) Ratio of CDX2 positive nuclear area to total nuclear area. P-value estimates using one-sided (alternative = less) Wilcoxon rank sum test for proximal vs. distal differences. 13 proximal and 13 distal colon cancer samples were analyzed.

Supplementary Tables

Supplementary Table 1

Oligos used for generating guide RNA for CRISPR/Cas9 genome editing and corresponding PCR primers for Sanger sequence.

Gene name	Oligo name	Sequence
Cdx2	Cas9_Cdx2_gRNA_F	CACCGAGCGGCGAAACCTGTGCGAG
	Cas9_Cdx2_gRNA_R	AAACCTCGCACAGGTTTCGCCGCTC
	Primer_Cdx2_F	CGAGGACTGGAATGGCTACG
	Primer_Cdx2_R	TGCTTAGGACACTACCGGGA
Sox17	Cas9_Sox17_gRNA_F	CACCGCCGGATGCGAGACTCCGCTT
	Cas9_Sox17_gRNA_R	AAACAAGCGGAGTCTCGCATCCGGC
	Primer_ Sox17_F	AAAGGTGAAAGGCGAGGTGG
	Primer_ Sox17_R	TGGCCCAAAGACCAAAGTGT
Sfrp4	Cas9_ Sfrp4_gRNA_F	CACCGCCAAAATAAAAGCCGTCCAG
	Cas9_ Sfrp4_gRNA_R	AAACCTGGACGGCTTTTATTTTGGC
	Primer_ Sfrp4_F	TTGGCCCCAAAACACTGTCT
	Primer_ Sfrp4_R	ACAGCATCTCAAAAGAGACGTCT
Cdkn2a	Cas9_Cdkn2a_gRNA_F	CACCGGTGCGATATTTGCGTTCCGC
	Cas9_Cdkn2a_gRNA_R	AAACGCGGAACGCAAATATCGCACC
	Primer_ Cdkn2a _F	GGTGATGATGATGGGCAACG
	Primer_ Cdkn2a _R	AGACCTCTGAACCCTCCTGC
Control	Cas9_Scramble_gRNA_F	CACCGGCACTCACATCGCTACATCA
	Cas9_Scramble_gRNA_R	AAACTGATGTAGCGATGTGAGTGCC

Supplementary Table 2

Oligos used for generating guide RNA for CRISPR/Cpf1 genome editing and corresponding PCR primers for Sanger sequence.

Gene name	Oligo name	Sequence
Cdx2	Cpf1_Cdx2_gRNA_F	AGATGCCGCTGGCCGCTGGGGGACAGC
	Cpf1_Cdx2_gRNA_R	AAAAGCTGTCCCCCAGCGGCCAGCGGC
	Primer_Cdx2_F	CGAGGACTGGAATGGCTACG
	Primer_Cdx2_R	AGGTTGCCAATGGGGTACTG
Sox17	Cpf1_Sox17_gRNA_F	AGATCACGCGCAACCCCAGCAGCCGCT
	Cpf1_Sox17_gRNA_R	AGCGGCTGCTGGGGTTGCGCGTG
	Primer_ Sox17_F	GCTCCAGTCTCGGACTATGC
	Primer_ Sox17_R	GTAACACTGCTTCTGGCCCT
Sfrp4	Cpf1_ Sfrp4_gRNA_F	AGATCATCTACCACAGTTGTGACCTCA
	Cpf1_ Sfrp4_gRNA_R	AAAATGAGGTCACAACTGTGGTAGATG
	Primer_ Sfrp4_F	TTGGCCCCAAAACACTGTCT
	Primer_ Sfrp4_R	ACAGCATCTCAAAAGAGACGTCT
Cdkn2a	Cpf1_Cdkn2a_gRNA_F	AGATAGGTGATGATGATGGGCAACGTT
	Cpf1_Cdkn2a_gRNA_R	AAAAAACGTTGCCCATCATCATCACCT
	Primer_ Cdkn2a _F	ACACAATCCCAGTTCGGCTT
	Primer_ Cdkn2a _R	ACGATGTCTTGATGTCCCCG
KO-3	Cpf1_KO-3_gRNA_F	AGATAGGTGATGATGATGGGCAACGTTAATTTCTACTCTTGTAGATCACGCGCAACCCCAGCAGCCGCTAATTTCTACTCTTGTAGATCATCTACCACAGTTGTGACCTCA
	Cpf1_KO-3_gRNA_R	AAAATGAGGTCACAACTGTGGTAGATGATCTACAAGAGTAGAAATTAGCGGCTGCTGGGGTT

		GCGCGTGATCTACAAGAGTAGAAATTAACGT TGCCCATCATCATCACCT
KO-4	Cpf1_KO-4_gRNA_F	AGATAGGTGATGATGATGGGCAACGTTAATT TCTACTCTTGTAGATCACGCGCAACCCCAGC AGCCGCTAATTTCTACTCTTGTAGATCATCTA CCACAGTTGTGACCTCAAATTTCTACTCTTGT AGATGCCGCTGGCCGCTGGGGGACAGC
	Cpf1_KO-4_gRNA_R	AAAAGCTGTCCCCCAGCGGCCAGCGGCATC TACAAGAGTAGAAATTTGAGGTCACAACTGT GGTAGATGATCTACAAGAGTAGAAATTAGCG GCTGCTGGGGTTGCGCGTGATCTACAAGAGT AGAAATTAACGTTGCCCATCATCATCACCT

Supplementary Table 3

Methods resource listing details of reagents, public datasets and software used in this work.

REAGENT or RESOURCE	SOURCE	IDENTIFIER
**Antibodies**		
Rabbit anti-CDX2 Antibody (WB: 1:1000)	Bethyl Laboratories	Cat# A300-691A
Rabbit monoclonal anti-CDX2 Antibody (EPR2764Y) (IHC 1:1000)	Abcam	Cat# ab76541
Anti-KRT20 antibody (D9Z1Z) XP® Rabbit mAb (IHC: 1:1000)	Cell Signaling Technology	Cat# 13063
Non-phospho β-Catenin Rabbit mAb (IHC: 1:750)	Cell Signaling Technology	Cat# 8814
Anti-CDKN2A antibody (WB: 1:1000)	Abcam	Cat# ab211542
Anti- SFRP4 antibody	Abcam	Cat# ab154167

(WB: 1:1000)		
Anti-SOX17 antibody<br>(WB: 1:1000)	R&D systems	Cat# AF1924
Recombinant Anti-Ki67 antibody<br>(IHC: 1:200)	Abcam	Cat# ab16667
β-Actin Rabbit mAb<br>(WB: 1:1000)	Cell Signaling Technology	Cat# 4970S
Anti-mouse IgG, HRP-linked Antibody<br>(WB: 1:2000)	Cell Signaling Technology	Cat# 7076
Anti-rabbit IgG, HRP-linked Antibody<br>(WB: 1:2000)	Cell Signaling Technology	Cat# 7074
Anti-goat IgG, HRP-linked Antibody<br>(WB: 1:2000)	Abcam	Cat# ab6741
**Bacterial and Virus Strains**		
NEB® Stable Competent E. coli	New England Biolabs	Cat# C3040I
NEB 5-alpha Competent E. coli	New England Biolabs	Cat# C2987I
JM109 Competent Cells	Promega	Cat# L2005
Puro.Cre empty vector	Addgene	Cat# 17408
pY108 (lenti-AsCpf1)	Addgene	Cat# 84739
LentiCRISPR v2-Blast	Addgene	Cat# 83480
PMD2.G	Addgene	Cat# 12259
PsPAX2	Addgene	Cat# 12260
pBABE-hygro-hTERT	Addgene	Cat# 1773
**Chemical, Peptides and Recombinant Proteins**		
Opti-MEM™ I Reduced Serum Medium	Thermo Fisher Scientific	Cat# 31985062
Lipofectamine™ 3000 Transfection Reagent	Thermo Fisher Scientific	Cat# L3000008
Cell Recovery Solution	Corning	Cat# 354253
Dispase	Stemcell Technologies	Cat# 07913
Basement Membrane Matrix	Corning	Cat# 356231
Puromycin dihydrochloride	Millipore Sigma	Cat# P8833
Blasticidin S HCl	Thermo Fisher Scientific	Cat# R21001
Hygromycin B	Thermo Fisher Scientific	Cat# 10687010

B-27™ Supplement (50X)	Thermo Fisher Scientific	Cat# 17504044
Advanced DMEM/F-12	Thermo Fisher Scientific	Cat# 12634010
ACCUMAX™	Stemcell Technologies	Cat# 07921
TransDux™ MAX Lentivirus Transduction Reagent	SBI System Biosciences	Cat# LV860A-1
Y-27632	Cell Signaling Technology	Cat# 13624
Platinum™ Taq DNA Polymerase High Fidelity	Thermo Fisher Scientific	Cat# 11304011
T4 DNA Ligase	New England Biolabs	Cat# M0202S
T4 Polynucleotide Kinase	New England Biolabs	Cat# M0201S
BsmBI-v2	New England Biolabs	Cat# R0739S
T7 DNA Ligase	New England Biolabs	Cat# M0318S
Dynabeads™ Protein A for Immunoprecipitation	Thermo Fisher Scientific	Cat# 10002D
AMPure XP Reagent	Beckman Coulter	Cat# A63880
Primocin	InvivoGen	Cat# ant-pm-05
A 83-01	Millipore Sigma	Cat# SML0788-5MG
hEGF	Millipore Sigma	Cat# E9644-.2MG
CHIR99021	Millipore Sigma	Cat# SML1046
Terrific Broth 500ml	Quality Biological	Cat# 340-071-101
LB agar plates with 100ug/ml ampicillin	Vitascientific	Cat# 340-108-231
Precision plus protein™ standard	Bio-Rad	Cat# 161-0394
SOC Outgrowth Medium	New England Biolabs	Cat# B9020S
Opti-MEM I Reduced Serum Medium	Thermo Fisher Scientific	Cat# 31985062
Penicillin-Streptomycin	Thermo Fisher Scientific	Cat# 15140122
SB 202190	Millipore Sigma	Cat# S7067-5MG
Pierce™ 16% Formaldehyde (w/v)	Thermo Fisher Scientific	Cat# 28906
ZR 100 bp DNA Marker	Zymo Research	Cat# M5002-200
iTaq universal SYBR green supermix	Bio-Rad	Cat# 1725124
qScript cDNA SuperMix	Quantabio	Cat# 95048-100
SYBR™ Green I Nucleic Acid Gel Stain	Thermo Fisher Scientific	Cat# S7563
KAPA HiFi HotStart ReadyMix	Roche Sequencing Solutions	Cat# KK2601

**Critical Commercial Assays**		
Gibson Assembly® Cloning Kit	New England Biolabs	Cat# E5510S
RNeasy Plus Mini kit	QIAGEN	Cat# 74104
Pierce™ BCA Protein Assay Kit	Thermo Fisher Scientific	Cat# 23227
pGEM-T easy vector system	Promega	Cat# A1380
Monarch® DNA Gel Extraction Kit	New England Biolabs	Cat# T1020S
Nextera XT DNA Library Preparation Kit (24 samples)	Illumina	Cat# FC-131-1024
Nextera XT Index Kit (24 Indexes, 96 samples	Illumina	Cat# FC-131-1001
Illumina Tagment DNA TDE1 Enzyme and Buffer Kits	Illumina	Cat# 20034197
SMARTer Stranded Total RNA Sample Prep Kit	Takara Bio USA	Cat# 634875
PureLink Quick Plasmid Miniprep Kit	Thermo Fisher Scientific	Cat# K210011
MinElute PCR Purification Kit	QIAGEN	Cat# 28004
ZymoPURE II Plasmid Midiprep Kit	Zymo Research	Cat# D4200
MycoAlert® Mycoplasma Detection Kit	Lonza	Cat#: LT07-318
**Deposited Data**		
Raw and analyzed Chip-seq data	Illumina NovaSeq 6000	GEO database: GSE218479
Raw and analyzed RNA-seq	Illumina HiSeq 4000	GEO database: GSE218480
**Reused Published Data**		
Cdx2 expression in colon cancer	Reference[1]	GEO database: GSE39582
Cdx2 expression in colon cancer	Reference[2]	TCGA-COAD
**Experimental Models: Cell lines**		
Lenti-X^{TM} 293T Cell line	Clontech	Cat# 632180
**Experimental Models: Organisms/Strains**		

B6;129S6-Gt(ROSA)26Sor$^{tm9(CAG-tdTomato)}$Hze/J	The Jackson Laboratory	Cat# 007905
B6.129P2(Cg)-Braf^{tm1Mmcm}/J	The Jackson Laboratory	Cat# 017837
NOD.Cg-Prkdc^{scid} Il2rg^{tm1Wjl}/SzJ	The Jackson Laboratory	Cat# 005557
**Oligonucleotides**		
Guide RNAs for Cas9 and corresponding primers for Sanger sequence, see Table S1	IDT	http://crispor.tefor.net/crispor.py
Primers for RT-qPCR, see Table S2	IDT	https://www.primer3plus.com/index.html
Guide RNAs for Cpf1 and corresponding primers for Sanger sequence, see Table S3	IDT	http://crispor.tefor.net/crispor.py
**Software and Algorithms**		
R statistical software	Open source	http://www.r-project.org
GraphPad Prism	GraphPad Software	https://www.graphpad.com/scientifific software/prism/
Fiji	Open source	http://fifiji.sc/Fiji
Adobe illustrator	Adobe	https://www.adobe.com/products/illustrator.html
Salmon (v1.1.0)	Reference[3]	N/A
tximport (1.10.1)	Reference[4]	N/A
DESeq2 (1.28.1)	Reference[5]	N/A
clusterProfiler (v3.16.1)	Reference[6]	N/A
FastQC (v0.11.9)	N/A	https://www.bioinformatics.babraham.ac.uk/projects/fastqc/
Trimmomatic (0.39)	Reference[7]	N/A
Bowtie2 (v2.4.1)	Reference[8]	N/A
Samtools (v1.9)	Reference[9]	N/A
Macs2 (v2.2.7)	Reference[10]	N/A
ChIPseeker (1.18.0)	Reference[11]	N/A
DiffBind (2.10.0)	Reference[12]	N/A

Supplementary Table 4

Primers for RT-PCR

Gene name	Oligo name	Sequence
Ephb2	Primer_Ephb2_F	AACCTTCAAGGCCAACCAAG
	Primer_ Ephb2_R	ATGGTTGTGCAAGGCATGTC
Lgr5	Primer_ Lgr5_F	AATCGCGGTAGTGGACATTC
	Primer_ lgr5_R	AACGATTTGGCAGCCGATTC
Ascl2	Primer_Ascl2_F	AGCACACCTTGACTGGTACG
	Primer_Ascl2_R	GTGGACGTTTGCACCTTCAC
Muc2	Primer_Muc2_F	TCGCTGGGTGTGTAAAGACC
	Primer_Muc2_R	GTTCGCTCTTGGTCAGGACA
Fabp2	Primer_Fabp2_F	GCCTGGACCATTGAGGGAAA
	Primer_Fabp2_R	GCTTGGCCTCAACTCCTTCA
Krt20	Primer_Krt20_F	TGGGCAACAATGTCAACGTG
	Primer_Krt20_R	TGTTCTTTGGCCTCTTGCAG
Car1	Primer_Car1_F	GTGACCTGGGTGATCTGCAA
	Primer_Car1_R	AACTGGAACTGCAGGCTCTC
β-actin	Primer_β-actin_F	GGCTGTATTCCCCTCCATCG
	Primer_β-actin_R	CCAGTTGGTAACAATGCCATGT

Raw data for supplemental figures

Supplementary Figure 4c:

Supplementary Figure 4c

β-actin

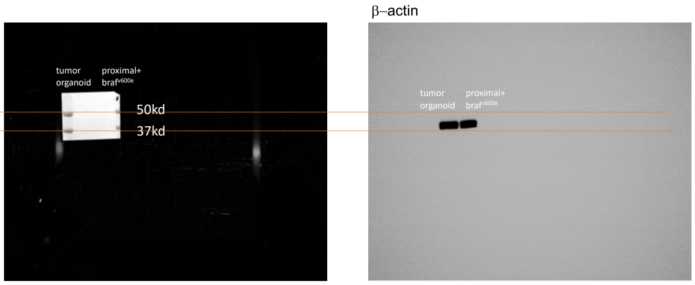

Cdkn2a

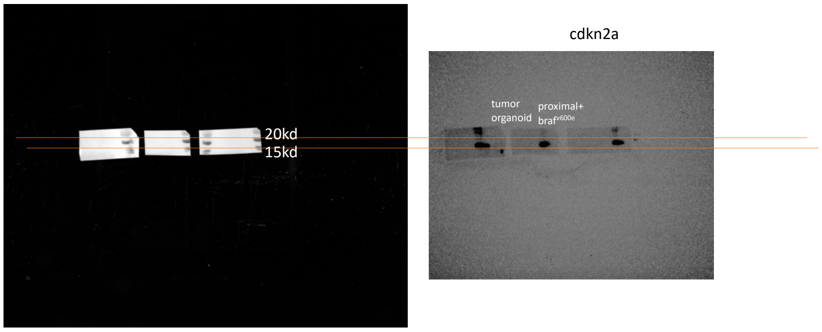

Cdx2

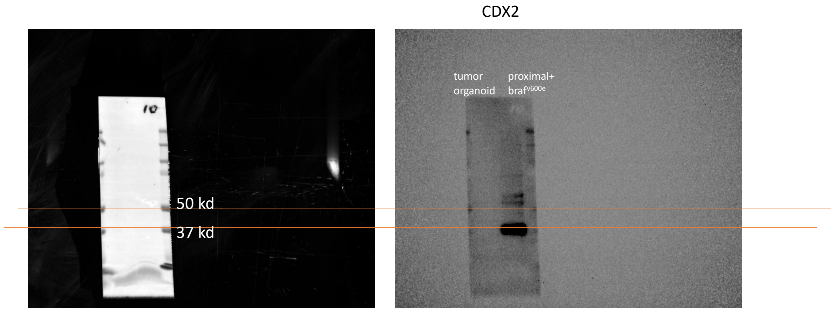

Supplementary Figure 4c

Sfrp4

Sfrp4

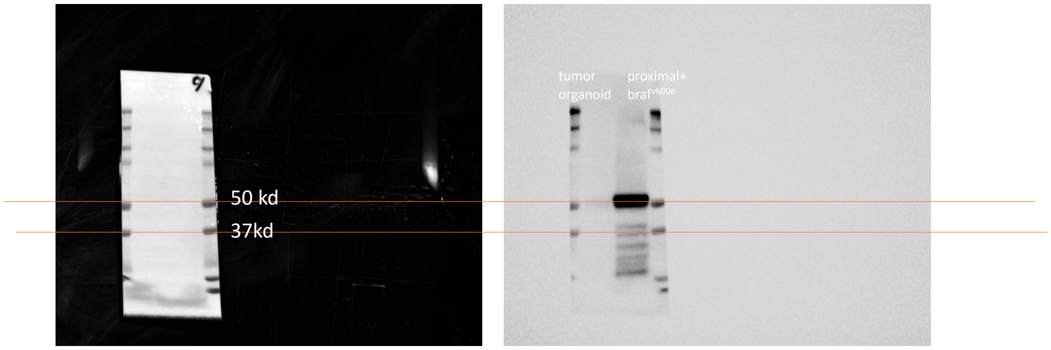

Sox17

sox17

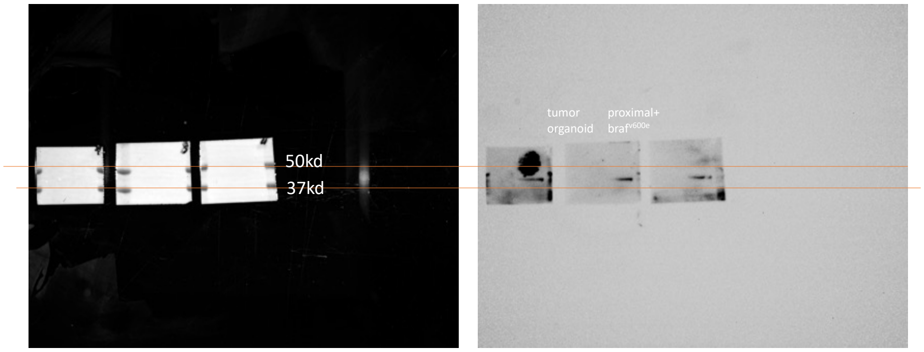

Marker

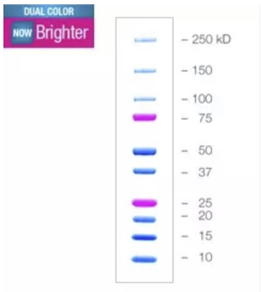

Supplementary Figure 6e:

Supplementary Figure 6e

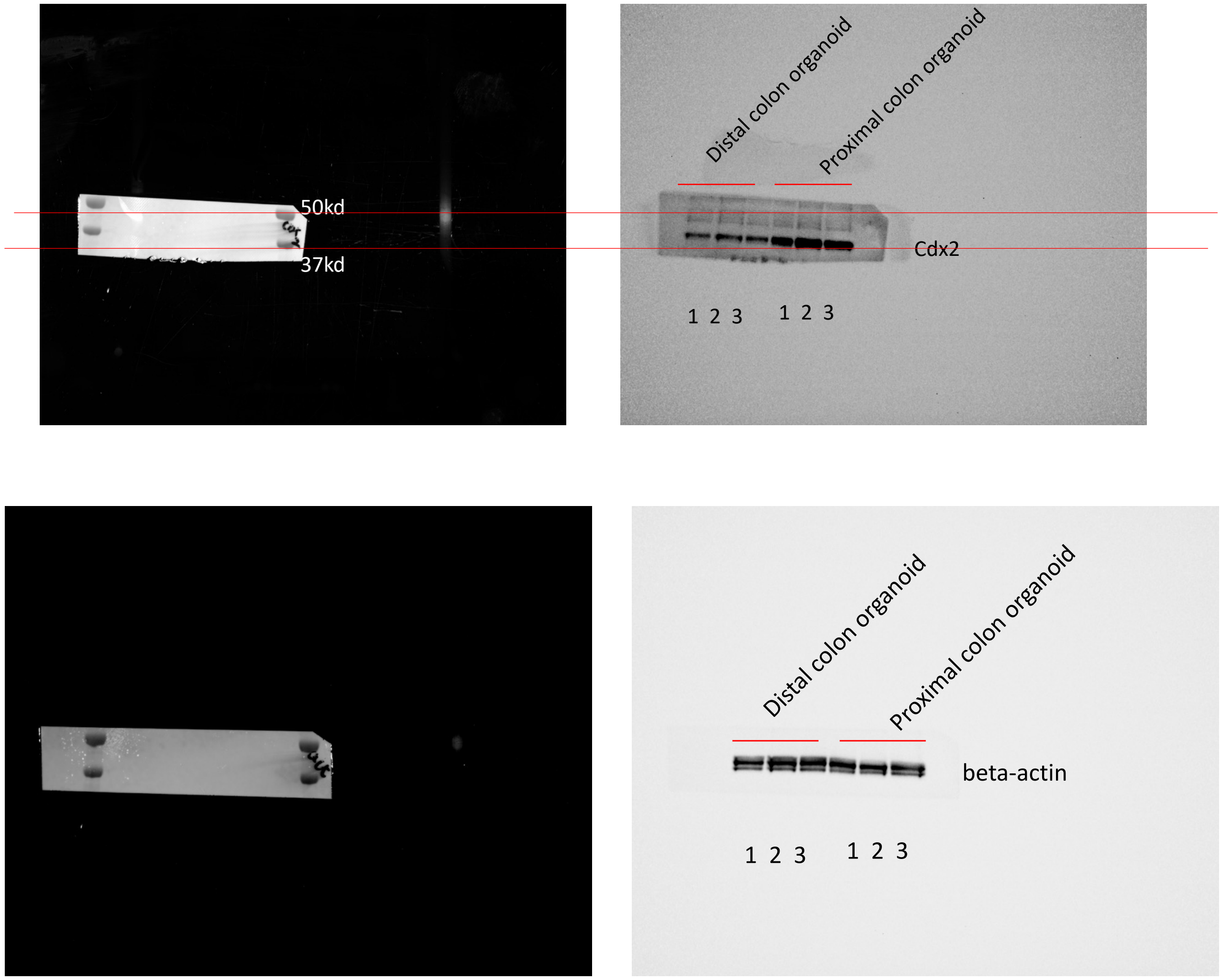

Supplementary Figure 6f:

Supplementary Figure 6f

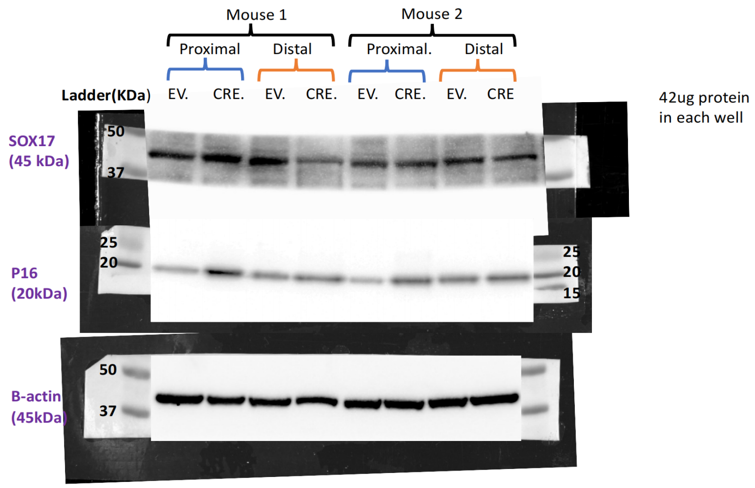

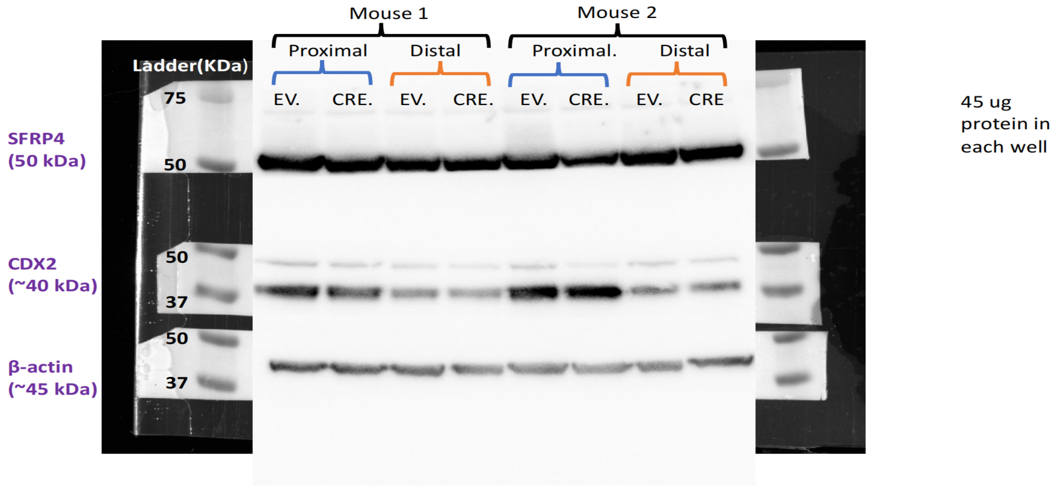

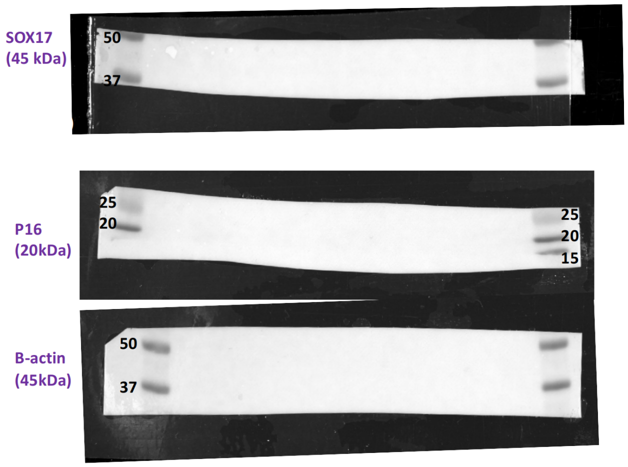
SOX17
(45 kDa)
50
37
P16
(20kDa)
25
20
25
20
15
B-actin
(45kDa)
50
37

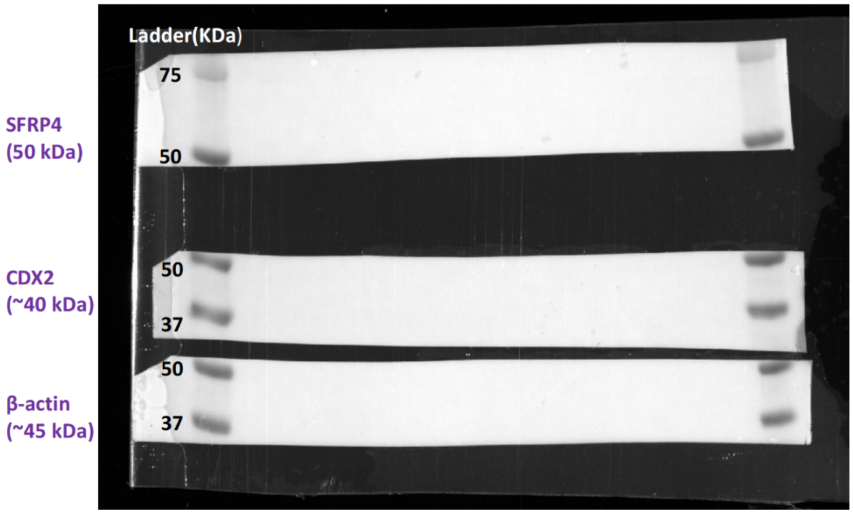
Ladder(KDa)
75
50
SFRP4
(50 kDa)
CDX2
(~40 kDa)
50
37
β-actin
(~45 kDa)
50
37

Supplementary References

1. Marisa, L. *et al.* Gene expression classification of colon cancer into molecular subtypes: characterization, validation, and prognostic value. *PLoS Med* **10**, e1001453 (2013).
2. Cancer Genome Atlas Network. Comprehensive molecular characterization of human colon and rectal cancer. *Nature* **487**, 330–337 (2012).
3. Patro, R., Duggal, G., Love, M. I., Irizarry, R. A. & Kingsford, C. Salmon provides fast and bias-aware quantification of transcript expression. *Nat Methods* **14**, 417–419 (2017).
4. Soneson, C., Love, M. I. & Robinson, M. D. Differential analyses for RNA-seq: transcript-level estimates improve gene-level inferences. *F1000Res* **4**, 1521 (2015).
5. Love, M. I., Huber, W. & Anders, S. Moderated estimation of fold change and dispersion for RNA-seq data with DESeq2. *Genome Biol* **15**, 550 (2014).
6. Yu, G., Wang, L.-G., Han, Y. & He, Q.-Y. clusterProfiler: an R package for comparing biological themes among gene clusters. *OMICS* **16**, 284–287 (2012).
7. Bolger, A. M., Lohse, M. & Usadel, B. Trimmomatic: a flexible trimmer for Illumina sequence data. *Bioinformatics* **30**, 2114–2120 (2014).
8. Langmead, B. & Salzberg, S. L. Fast gapped-read alignment with Bowtie 2. *Nat Methods* **9**, 357–359 (2012).
9. Li, H. *et al.* The Sequence Alignment/Map format and SAMtools. *Bioinformatics* **25**, 2078–2079 (2009).

10. Feng, J., Liu, T., Qin, B., Zhang, Y. & Liu, X. S. Identifying ChIP-seq enrichment using MACS. *Nat Protoc* **7**, 1728–1740 (2012).
11. Yu, G., Wang, L.-G. & He, Q.-Y. ChIPseeker: an R/Bioconductor package for ChIP peak annotation, comparison and visualization. *Bioinformatics* **31**, 2382–2383 (2015).
12. Ross-Innes, C. S. *et al.* Differential oestrogen receptor binding is associated with clinical outcome in breast cancer. *Nature* **481**, 389–393 (2012).
