# Supplementary material for: Tissue-location-specific transcription programs drive tumor dependencies in colon cancer
Source: Nat Commun. 2024 Feb 15;15:1384. doi: 10.1038/s41467-024-45605-4 (PMC10869357; doi:10.1038/s41467-024-45605-4)
Supplement: Supplementary file 3 — Reporting Summary [file 41467_2024_45605_MOESM3_ESM.pdf]

Corresponding author(s): Hariharan Easwaran

Last updated by author(s): Dec 15, 2023

Reporting Summary

Nature Portfolio wishes to improve the reproducibility of the work that we publish. This form provides structure for consistency and transparency in reporting. For further information on Nature Portfolio policies, see our Editorial Policies and the Editorial Policy Checklist.

Statistics

For all statistical analyses, confirm that the following items are present in the figure legend, table legend, main text, or Methods section.

n/a	Confirmed	
☐	☒	The exact sample size (*n*) for each experimental group/condition, given as a discrete number and unit of measurement
☐	☒	A statement on whether measurements were taken from distinct samples or whether the same sample was measured repeatedly
☐	☒	The statistical test(s) used AND whether they are one- or two-sided<br>*Only common tests should be described solely by name; describe more complex techniques in the Methods section.*
☒	☐	A description of all covariates tested
☐	☒	A description of any assumptions or corrections, such as tests of normality and adjustment for multiple comparisons
☐	☒	A full description of the statistical parameters including central tendency (e.g. means) or other basic estimates (e.g. regression coefficient) AND variation (e.g. standard deviation) or associated estimates of uncertainty (e.g. confidence intervals)
☐	☒	For null hypothesis testing, the test statistic (e.g. *F*, *t*, *r*) with confidence intervals, effect sizes, degrees of freedom and *P* value noted<br>*Give P values as exact values whenever suitable.*
☒	☐	For Bayesian analysis, information on the choice of priors and Markov chain Monte Carlo settings
☒	☐	For hierarchical and complex designs, identification of the appropriate level for tests and full reporting of outcomes
☐	☒	Estimates of effect sizes (e.g. Cohen's *d*, Pearson's *r*), indicating how they were calculated

Our web collection on statistics for biologists contains articles on many of the points above.

Software and code

Policy information about availability of computer code

Data collection	For data collection, standard molecular biology techniques such as RNA-seq, ChIP-seq, were used.<br>The information on data collection s described in the Methods and the data is available in public databases.
Data analysis	We have used open source R/Bioconductor and Unix command line-based sequence analyses softwares. All these softwares are published, and are listed below:<br>Salmon (v1.1.0)<br>tximport (1.10.1)<br>DESeq2 (1.28.1)<br>clusterProfiler (v3.16.1)<br>FastQC (v0.11.9)<br>Trimmomatic (0.39)<br>Bowtie2 (v2.4.1)<br>Samtools (v1.9)<br>Macs2 (v2.2.7)<br>ChIPseeker (1.18.0)<br>DiffBind (2.10.0)

For manuscripts utilizing custom algorithms or software that are central to the research but not yet described in published literature, software must be made available to editors and reviewers. We strongly encourage code deposition in a community repository (e.g. GitHub). See the Nature Portfolio guidelines for submitting code & software for further information.

Data

Policy information about availability of data

All manuscripts must include a data availability statement. This statement should provide the following information, where applicable:

- Accession codes, unique identifiers, or web links for publicly available datasets
- A description of any restrictions on data availability
- For clinical datasets or third party data, please ensure that the statement adheres to our policy

The accession number in GEO database for the RNAs-seq data reported in this paper is GSE218480, Chip-seq data is GSE218479. The data has been made avaialable without restrictions.
Data used in figures are available within the Article, Supplementary Information or Source Data file. Source data are provided with this paper. All source data and figures are also uploaded to Figshare (DOI: 10.6084/m9.figshare.24850143). The Figshare data is available at https://figshare.com/s/bcda4b948ffdcf124faa.

All software codes used in the analyses of the data deposited in GitHub (https://github.com/Baylin-Easwaran-Labs/Lijing-Yang-Project). The Zenodo DOI for the software code submitted in GitHub is DOI: 10.5281/zenodo.10309481

Additional publicly available datasets used:
1) The publicly available gene expression data from 443 colon cancer patients used in this study are available in the GEO database under accession GSE39582 at this hyperlink: https://www.ncbi.nlm.nih.gov/geo/query/acc.cgi?acc=GSE39582.

2) The The Crypt Top and Bottom GeneSetList markers used in our study are available at https://www.pnas.org/doi/suppl/10.1073/pnas.0707210104/suppl_file/07210table1.xls).

3) The single cell based gene expression markers for colon epithelial cell types are from are available at https://static-content.springer.com/esm/art%3A10.1038%2Fs41586-019-0992-y/MediaObjects/41586_2019_992_MOESM3_ESM.xlsx.

Research involving human participants, their data, or biological material

Policy information about studies with human participants or human data. See also policy information about sex, gender (identity/presentation), and sexual orientation and race, ethnicity and racism.

Reporting on sex and gender	Not applicable
Reporting on race, ethnicity, or other socially relevant groupings	Not applicable
Population characteristics	Not applicable
Recruitment	Not applicable
Ethics oversight	Not applicable

Note that full information on the approval of the study protocol must also be provided in the manuscript.

Field-specific reporting

Please select the one below that is the best fit for your research. If you are not sure, read the appropriate sections before making your selection.

☒ Life sciences ☐ Behavioural & social sciences ☐ Ecological, evolutionary & environmental sciences

For a reference copy of the document with all sections, see nature.com/documents/nr-reporting-summary-flat.pdf

Life sciences study design

All studies must disclose on these points even when the disclosure is negative.

Sample size

(1) For proximal vs. distal organoid assays, sample sizes were chosen based on empirical observation in previous studies (PMID: 30753828) showing that two independent mice-derived organoid replicates captured biological differences, as reiterated in our observations. To our knowledge there are no previous studies comparing proximal vs. distal organoid growth differences and tumorigenicity.

(2) For gene expression analyses, two organoid replicates (from independent mice) were used to detect differentially expressed genes which is powered to identify significant differences assuming log2 variance and expected fold-change (log2-FC) in expression of 0.35 and +/-1, respectively.

(3) For ChIPseq, a priori power calculation is difficult as it highly varies from experiment to experiment. We have used three independent

biological replicates and detected significantly enriched regions in our comparisons using DiffBind which was corrected for multiple hypotheses.

(4) Number of mice for xenograft experiments are n=5 per control-experiment group (a priori power=0.95, effect size = 2.8, p-value < 0.05 for one-tailed t-test, alternative hypothesis being mean tumor volume greater than control).

Data exclusions	No data was excluded.
Replication	The experiments were replicated 3-4 times at independent times over a period of 2-3 years.
Randomization	All experiments were done in parallel under identical conditions.
Blinding	There was no blinding in the experiments as there are no specific group allocations used in the analyses.

Reporting for specific materials, systems and methods

We require information from authors about some types of materials, experimental systems and methods used in many studies. Here, indicate whether each material, system or method listed is relevant to your study. If you are not sure if a list item applies to your research, read the appropriate section before selecting a response.

Materials & experimental systems

n/a	Involved in the study	
☐	☒	Antibodies
☐	☒	Eukaryotic cell lines
☒	☐	Palaeontology and archaeology
☐	☒	Animals and other organisms
☒	☐	Clinical data
☒	☐	Dual use research of concern
☒	☐	Plants

Methods

n/a	Involved in the study	
☐	☒	ChIP-seq
☒	☐	Flow cytometry
☒	☐	MRI-based neuroimaging

Antibodies

Antibodies used

Rabbit anti-CDX2 Antibody
BETHYL
Cat# A300-691A
(WB: 1:1000)

Keratin 20 (D9Z1Z) XP® Rabbit mAb
Cell Signaling Technology
Cat# 13063
(IHC: 1:1000)

Non-phospho (Active) Beta-Catenin (Ser33/37/Thr41) (D13A1)
Cell Signaling Technology
Rabbit mAb
cat# 8814
(IHC: 1:750)

Recombinant Anti-CDKN2A/p16INK4a antibody [EPR20418]
abcam
Rabbit monoclonal
cat# ab211542
(WB: 1:1000)

Recombinant Anti-SFRP4 antibody [EPR9389]
abcam
Rabbit monoclonal
cat# ab154167
(WB: 1:1000)

Human Anti-SOX17 Antibody
R&D SYSTEMS
Polyclonal Goat IgG
Catalog #: AF1924
(WB: 1:1000)

Recombinant Anti-Ki67 antibody [SP6] (ab16667)

abcam
Rabbit monoclonal [SP6] to Ki67
cat# ab16667
(IHC: 1:200)

beta-Actin (13E5) Rabbit mAb #4970
Cell Signaling Technology
Rabbit mAb
cat# 4970
(WB: 1:1000)

Recombinant Anti-CDX2 antibody [EPR2764Y]
abcam
Rabbit mAb
cat# (ab76541)
(IHC 1:1000)

Anti-mouse IgG, HRP-linked Secondary Antibody
Cell Signaling Technology
Cat# 7076
(WB: 1:2000)

Anti-rabbit IgG, HRP-linked Secondary Antibody
Cell Signaling Technology
Cat# 7074
(WB: 1:2000)

Anti-goat IgG, HRP-linked Secondary Antibody
Abcam
Cat# ab6741
(WB: 1:2000)

Validation

For all antibodies the validation statements in the manufacturer's website states that the Abs work for human and mouse species for the applicaitons they are used in this project (WB, IHC, ChIP). Below are the relevant citations from the manufacturers:

Rabbit anti-CDX2 Antibody
BETHYL
Cat# A300-691A
https://fortis-datasheets.s3.us-east-2.amazonaws.com/A300-691A-1.pdf

Keratin 20 (D9Z1Z) XP® Rabbit mAb
https://www.cellsignal.com/products/primary-antibodies/keratin-20-d9z1z-xp-rabbit-mab/13063

Non-phospho (Active) Beta-Catenin (Ser33/37/Thr41) (D13A1)
https://www.cellsignal.com/products/primary-antibodies/non-phospho-active-b-catenin-ser33-37-thr41-d13a1-rabbit-mab/8814

Recombinant Anti-CDKN2A/p16INK4a antibody [EPR20418] (ab211542)
https://www.abcam.com/products/primary-antibodies/cdkn2ap16ink4a-antibody-epr20418-ab211542.html

Recombinant Anti-SFRP4 antibody [EPR9389] (ab154167)
https://www.abcam.com/products/primary-antibodies/sfrp4-antibody-epr9389-ab154167.html

Human SOX17 Antibody (R&D SYSTEMS)
https://www.rndsystems.com/products/human-sox17-antibody_af1924

Recombinant Anti-Ki67 antibody [SP6] (ab16667)
https://www.abcam.com/products/primary-antibodies/ki67-antibody-sp6-ab16667.html

beta-Actin (13E5) Rabbit mAb #4970
https://www.cellsignal.com/products/antibody-conjugates/b-actin-13e5-rabbit-mab-hrp-conjugate/5125

Recombinant Anti-CDX2 antibody [EPR2764Y]
https://www.abcam.com/products/primary-antibodies/cdx2-antibody-epr2764y-ab76541.html

Anti-mouse IgG, HRP-linked Antibody (Cell Signaling Technology Cat# 7076)
https://www.cellsignal.com/products/secondary-antibodies/anti-mouse-igg-hrp-linked-antibody/7076

Anti-rabbit IgG, HRP-linked Antibody (Cell Signaling Technology Cat #7074)
https://www.cellsignal.com/products/secondary-antibodies/anti-rabbit-igg-hrp-linked-antibody/7074

Anti-goat IgG, HRP-linked Antibody (abcam, Cat # ab6741)
https://www.abcam.com/products/secondary-antibodies/rabbit-goat-igg-hl-hrp-ab6741.html

Eukaryotic cell lines

Policy information about cell lines and Sex and Gender in Research

Cell line source(s)	The Lenti-X 293T Cell Line is a subclone of the transformed human embryonic kidney cell line, HEK 293, which is highly transfectable and supports high levels of viral protein expression taken from a female fetus in 1973.<br>Information provided in the manuscript Key Resources Table. This was the only cell line used. Cell line was cultured in DMEM medium with 10% FBS.
Authentication	Lenti-XTM 293T Cell line used were freshly obtained from Takara Bio USA, Inc. The cell line was not authenticated.
Mycoplasma contamination	All cell lines and organoid cultures are negative for Mycoplasma contamination. Tested every 3-6 months. The MycoAlert by Lonza kit was used.
Commonly misidentified lines<br>(See ICLAC register)	The only cell line used is Lenti-X 293T, which is not in the misidentified cell lines list.

Animals and other research organisms

Policy information about studies involving animals; ARRIVE guidelines recommended for reporting animal research, and Sex and Gender in Research

Laboratory animals	Following mice genotypes are used which are in the C57BL/6 background.<br><br>B6;129S6-Gt(ROSA)26Sortm9(CAG-tdTomato)Hze/J<br>Company: The Jackson Laboratory<br>Cat# 007905.<br>Six months old<br>mouse:<br>B6.129P2(Cg)-Braftm1Mmcm/J<br>Company: The Jackson Laboratory<br>Cat# 017837<br>Six month old<br>mouse:<br>NOD.Cg-Prkdcscid Il2rgtm1Wjl/SzJ<br>Company: The Jackson Laboratory<br>Cat# 005557<br>Two months old.<br><br>All details provided in Methods and Key Resources Table.
Wild animals	No wild animals used.
Reporting on sex	We used organoids generated using female mice in all studies. Detailed in methods.<br>In this work we focused on female mice because to avoid gender-based variability, and because BRAF-V600E driven proximal colon cancer has a higher incidence in female gender. Since we did not compare data in female vs. males, we do not claim that the findings are specific to female gender.
Field-collected samples	Study did not involve samples collected from the field.
Ethics oversight	All animal protocols and care were in accordance with guidelines of the institutional Animal Care and Use Committee (IACUC) and all experiments with mice were approved by the Johns Hopkins Animal Care and use Committee.

Note that full information on the approval of the study protocol must also be provided in the manuscript.

Plants

Seed stocks	Not applicable
Novel plant genotypes	Not applicable
Authentication	Not applicable

ChIP-seq

Data deposition

☒ Confirm that both raw and final processed data have been deposited in a public database such as GEO.

☒ Confirm that you have deposited or provided access to graph files (e.g. BED files) for the called peaks.

Data access links
May remain private before publication.

The accession number in GEO database for Chip-seq data is GSE218479. Data available without restriction.

Files in database submission

1 GSM6745932_chip1_align.markdup.bw
2 GSM6745932_chip1_peaks.narrowPeak.gz
3 GSM6745933_chip2_align.markdup.bw
4 GSM6745933_chip2_peaks.narrowPeak.gz
5 GSM6745934_chip3_align.markdup.bw
6 GSM6745934_chip3_peaks.narrowPeak.gz
7 GSM6745935_chip4_align.markdup.bw
8 GSM6745935_chip4_peaks.narrowPeak.gz
9 GSM6745936_chip5_align.markdup.bw
10 GSM6745936_chip5_peaks.narrowPeak.gz
11 GSM6745937_chip6_align.markdup.bw
12 GSM6745937_chip6_peaks.narrowPeak.gz
13 GSM6745938_chip7_align.markdup.bw
14 GSM6745938_chip7_peaks.narrowPeak.gz
15 GSM6745939_chip8_align.markdup.bw
16 GSM6745939_chip8_peaks.narrowPeak.gz
17 GSM6745940_chip9_align.markdup.bw
18 GSM6745940_chip9_peaks.narrowPeak.gz
19 GSM6745941_chip10_align.markdup.bw
20 GSM6745941_chip10_peaks.narrowPeak.gz
21 GSM6745942_chip11_align.markdup.bw
22 GSM6745942_chip11_peaks.narrowPeak.gz
23 GSM6745943_chip12_align.markdup.bw
24 GSM6745943_chip12_peaks.narrowPeak.gz
25 GSM6745944_input1_align.markdup.bw
26 GSM6745945_input2_align.markdup.bw
27 GSM6745946_input3_align.markdup.bw
28 GSM6745947_input4_align.markdup.bw
29 GSM6745948_input5_align.markdup.bw
30 GSM6745949_input6_align.markdup.bw
31 GSM6745950_input7_align.markdup.bw
32 GSM6745951_input8_align.markdup.bw
33 GSM6745952_input9_align.markdup.bw
34 GSM6745953_input10_align.markdup.bw
35 GSM6745954_input11_align.markdup.bw
36 GSM6745955_input12_align.markdup.bw

Genome browser session
(e.g. UCSC)

https://genome.ucsc.edu/s/Lijing%20Yang/Lijing_cdx2_mm10

Methodology

Replicates	Proximal and distal colon organoids were generated from 3 mice and cultured in WENR-plus medium for 4 months before collecting sample for ChIP-seq
Sequencing depth	We performed 150bp paired end reads.<br>chip1 (sequencing depth = 79572138 ; uniquely aligned reads = 75813121)

chip2 (sequencing depth = 94484364; uniquely aligned reads = 90180984)
chip3 (sequencing depth = 58204210; uniquely aligned reads = 53619751)
chip4 (sequencing depth = 94261618; uniquely aligned reads = 87964056)
chip5 (sequencing depth = 87787802 ; uniquely aligned reads = 82103934)
chip6 (sequencing depth = 81716414; uniquely aligned reads = 77235662)
chip7 (sequencing depth = 54174726 ; uniquely aligned reads = 51444064)
chip8 (sequencing depth = 65149356; uniquely aligned reads = 61738392)
chip9 (sequencing depth = 71001632; uniquely aligned reads = 67219594)
chip10 (sequencing depth = 70865676 ; uniquely aligned reads = 67387170)
chip11 (sequencing depth = 61517996 ; uniquely aligned reads = 57954755)
chip12 (sequencing depth = 72710780; uniquely aligned reads = 68808115)
input1 (sequencing depth = 79730256; uniquely aligned reads = 69215575)
input2 (sequencing depth = 99287244; uniquely aligned reads = 87766909)
input3 (sequencing depth = 70099254; uniquely aligned reads = 59865224)
input4 (sequencing depth = 79439928 ; uniquely aligned reads = 68457579)
input5 (sequencing depth = 63116284 ; uniquely aligned reads = 54784641)
input6 (sequencing depth = 84047654; uniquely aligned reads = 73263781)
input7 (sequencing depth = 65568960; uniquely aligned reads = 57310244)
input8 (sequencing depth = 83383114; uniquely aligned reads = 73083402)
input9 (sequencing depth = 69157338; uniquely aligned reads = 59532485)
input10 (sequencing depth = 93651122; uniquely aligned reads = 82624111)
input11 (sequencing depth = 64232450; uniquely aligned reads = 55318537)
input12 (sequencing depth = 83540080; uniquely aligned reads = 72388198)

Antibodies

Rabbit anti-CDX2 Antibody was used for ChIP-seq
BETHYL
Cat# A300-691A
https://www.fortislife.com/search?query=cdx2&pageSize=15

Peak calling parameters

The cleaned and mapped reads were subjected to peak calling performed by Macs2 peak caller with parameters ‘callpeak -f BAM -g mm -B -q 0.01’ and matched input was set as control.

Data quality

We used FASTQC to check initial quality of the seqeuncing reads, and majority of the reads have >35 Phred score.
We used FASTQC to check initial quality of the seqeuncing reads, and majority of the reads have >35 Phred score.
ChIPQC analyses (R package ChIPQC) show a RiP% in the range of 1.2-6.8% which is the expected enrichment in peaks for transcription factors.
ID SSD RiP%
chip1 1.8 5
chip2 2.2 5
chip3 1.4 1.4
chip4 2.3 3.4
chip5 2 6.8
chip6 1.6 2.7
chip7 1.5 2.8
chip8 2 2.1
chip9 1.4 3.1
chip10 1.5 2.9
chip11 1.3 1.2
chip12 1.4 1.8
FFollowing are the numbers of peaks identified at a q-value cut-off of 0.01 and above 5-fold enrichment caculated by Macs2 package.
chip1_peaks.narrowPeak 18756
chip2_peaks.narrowPeak 14048
chip3_peaks.narrowPeak 6266
chip4_peaks.narrowPeak 12156
chip5_peaks.narrowPeak 24150
chip6_peaks.narrowPeak 11217
chip7_peaks.narrowPeak 10147
chip8_peaks.narrowPeak 6045
chip9_peaks.narrowPeak 10859
chip10_peaks.narrowPeak 8214
chip11_peaks.narrowPeak 4915
chip12_peaks.narrowPeak 5700

Software

FastQC was utilized to certify quality of reads. Trimmomatic was used to trim the ChIP-Seq reads and FastQC was used again to check whether the adaptors were eliminated successfully. Bowtie2 was used to align reads to the mouse (mm10) genome. After marking and deleting the duplicate alignments using Samtools, the cleaned and mapped reads were subjected to peak calling performed by Macs2 peak caller with parameters ‘callpeak -f BAM -g mm -B -q 0.01’ and matched input was set as control. After getting the narrow peak using macs2, ChIPseeker was applied to annotate the peak calls which contain promoter, 5'UTR, 3'UTR, exon, intron and intergenic to binding and the region within ± 3 Kb from transcription start site was termed as promoter. DiffBind was used to find differential peaks between different group containing three biological repetitions.
