# Supplementary material for: Tissue-location-specific transcription programs drive tumor dependencies in colon cancer
Source: Nat Commun. 2024 Feb 15;15:1384. doi: 10.1038/s41467-024-45605-4 (PMC10869357; doi:10.1038/s41467-024-45605-4)
Supplement: Supplementary file 4 — Source Data [file 41467_2024_45605_MOESM4_ESM.zip › Source Data/Figures_Source_Data/supplemental figure 7/panel a/panel a.pptx]

## Slide 1
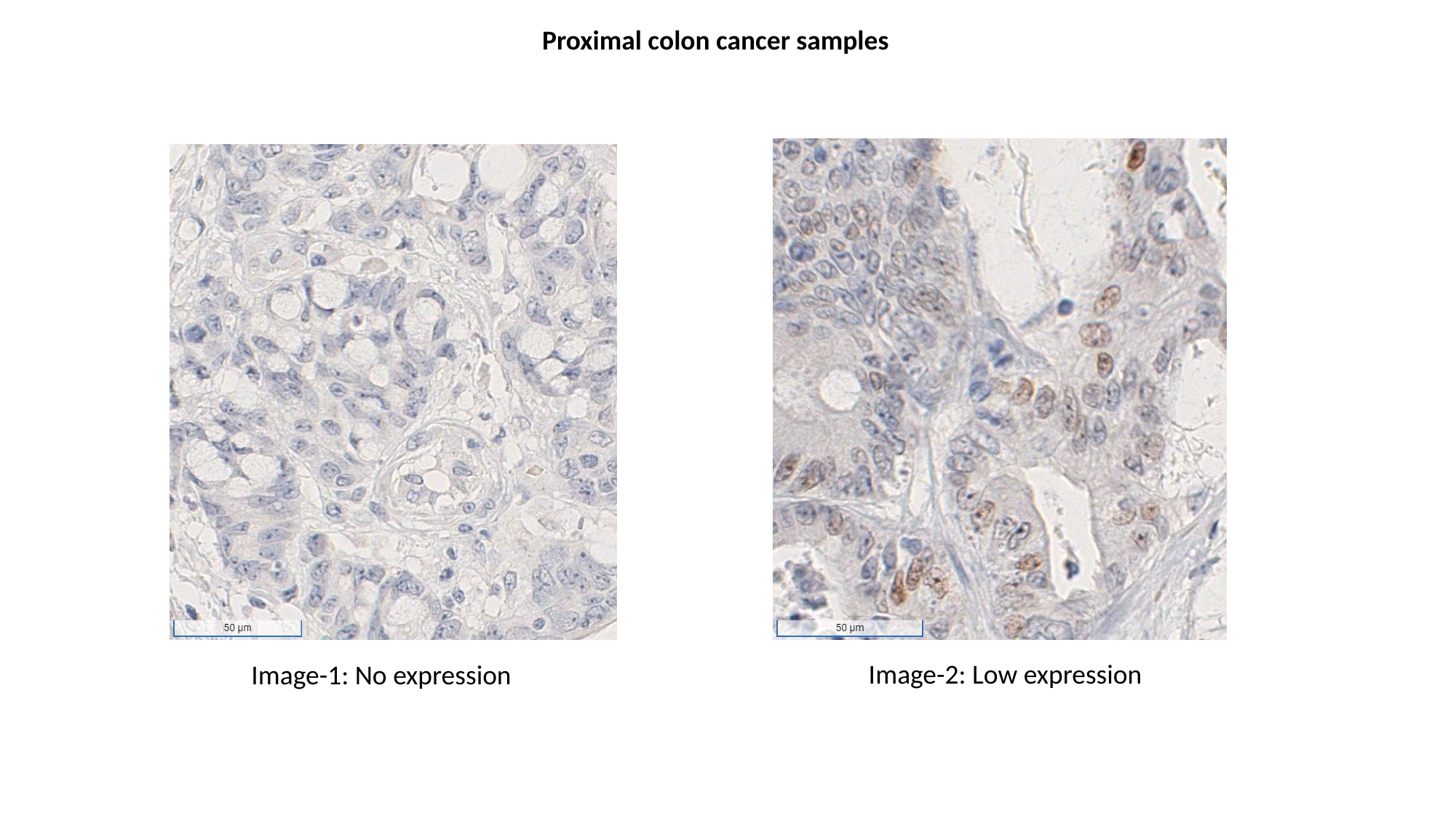

Proximal colon cancer samples
Image-2: Low expression
Image-1: No expression

## Slide 2
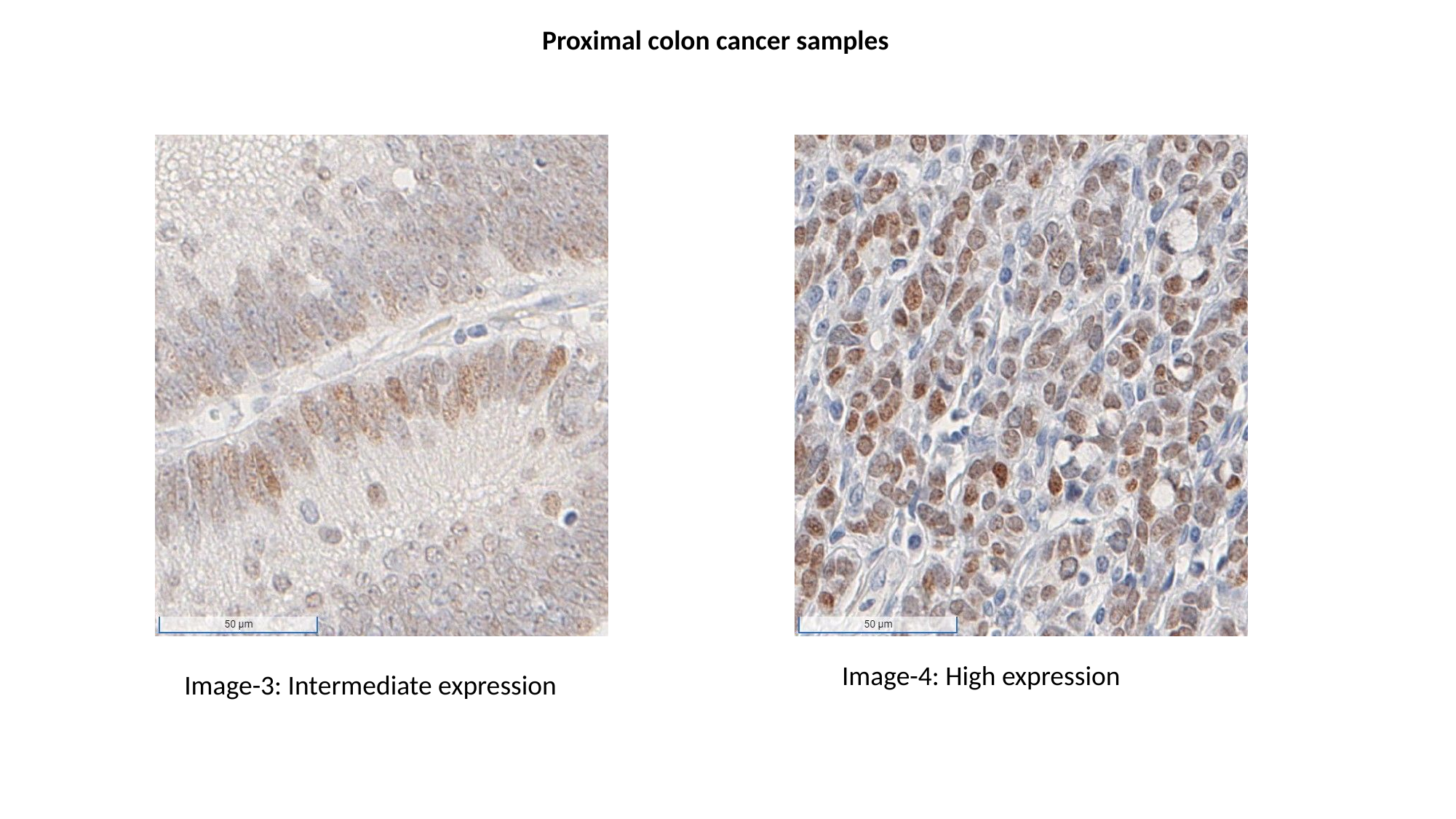

Proximal colon cancer samples
Image-4: High expression
Image-3: Intermediate expression

## Slide 3
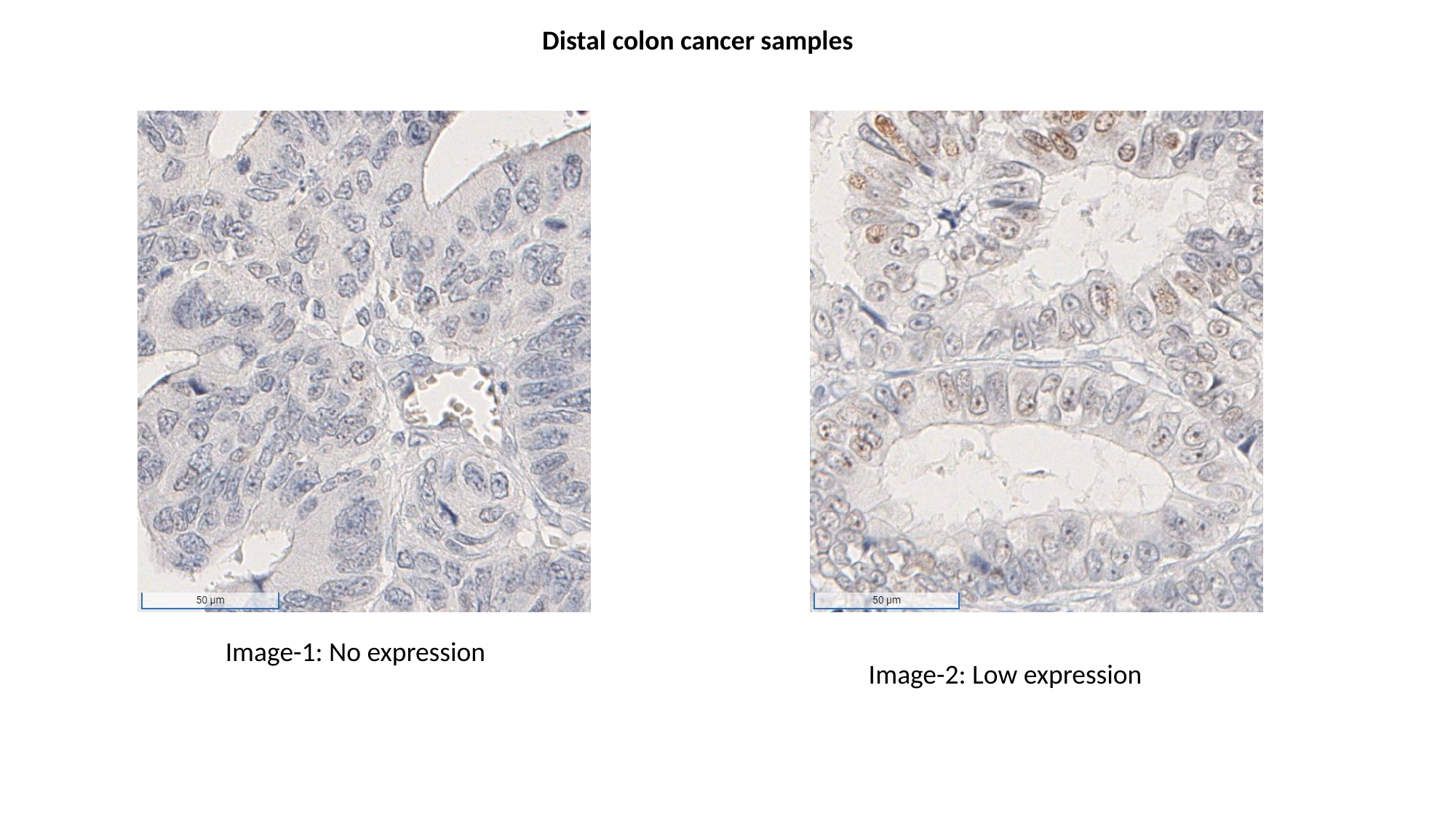

Distal colon cancer samples
Image-1: No expression
Image-2: Low expression

## Slide 4
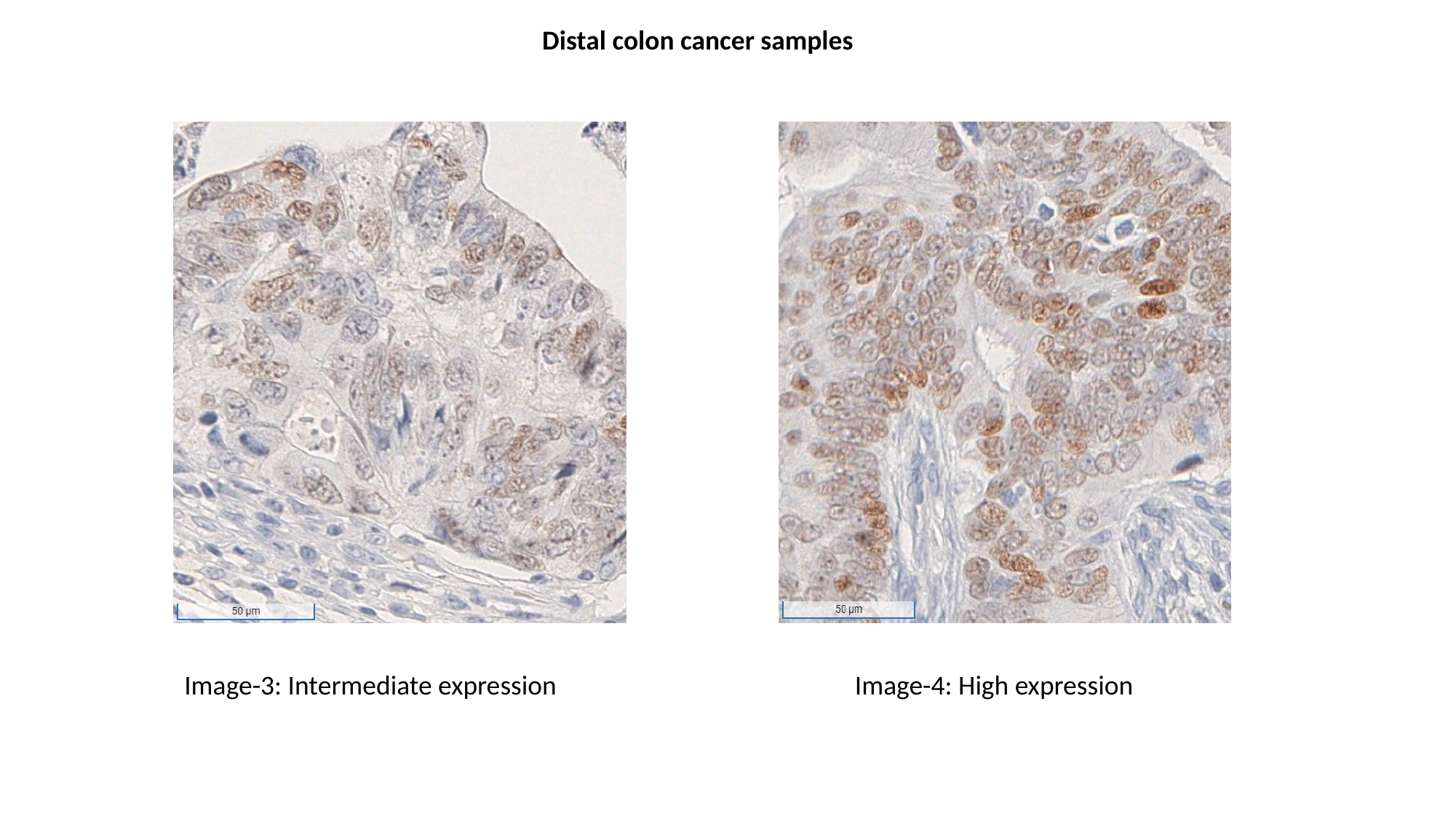

Distal colon cancer samples
Image-3: Intermediate expression
Image-4: High expression
