# Supplementary figures and images for: Tissue-location-specific transcription programs drive tumor dependencies in colon cancer (part 2 of 2)
Source: Nat Commun. 2024 Feb 15;15:1384. doi: 10.1038/s41467-024-45605-4 (PMC10869357; doi:10.1038/s41467-024-45605-4)

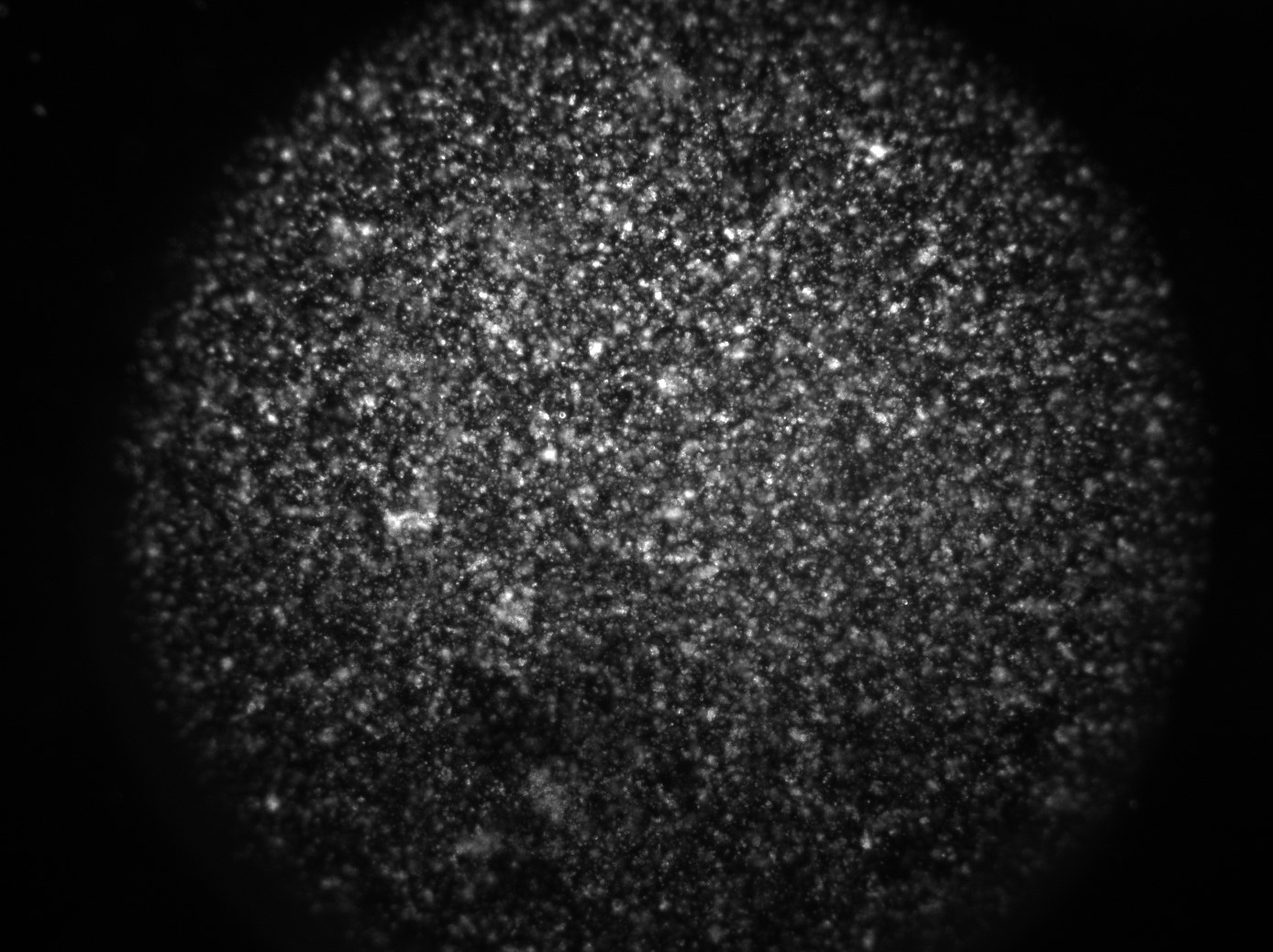

Supplement: Supplementary file 4 — Source Data [file 41467_2024_45605_MOESM4_ESM.zip › Source Data/Figures_Source_Data/figure 1/panel b/B2.jpg]

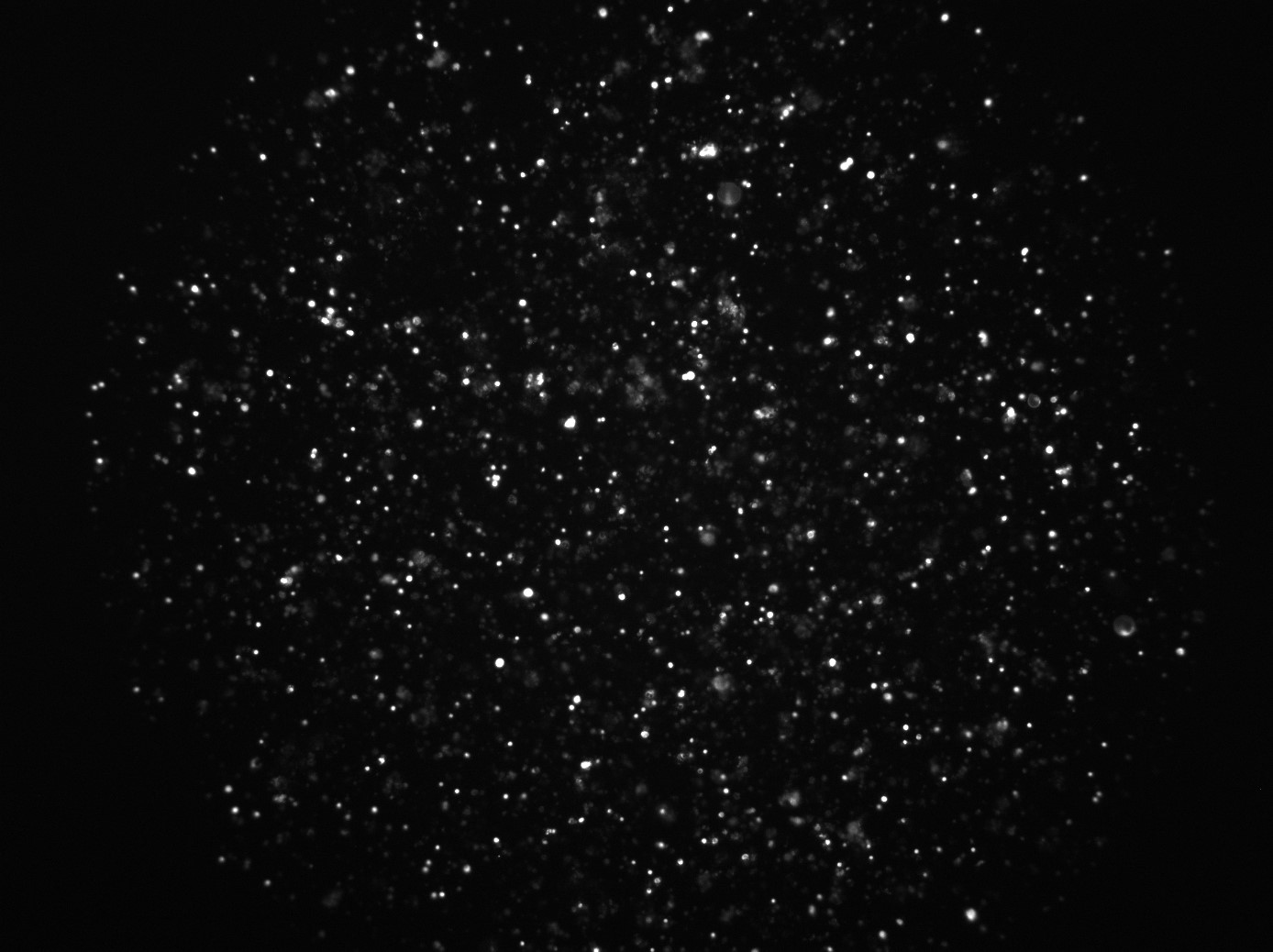

Supplement: Supplementary file 4 — Source Data [file 41467_2024_45605_MOESM4_ESM.zip › Source Data/Figures_Source_Data/figure 1/panel b/B8_TdTomato.jpg]

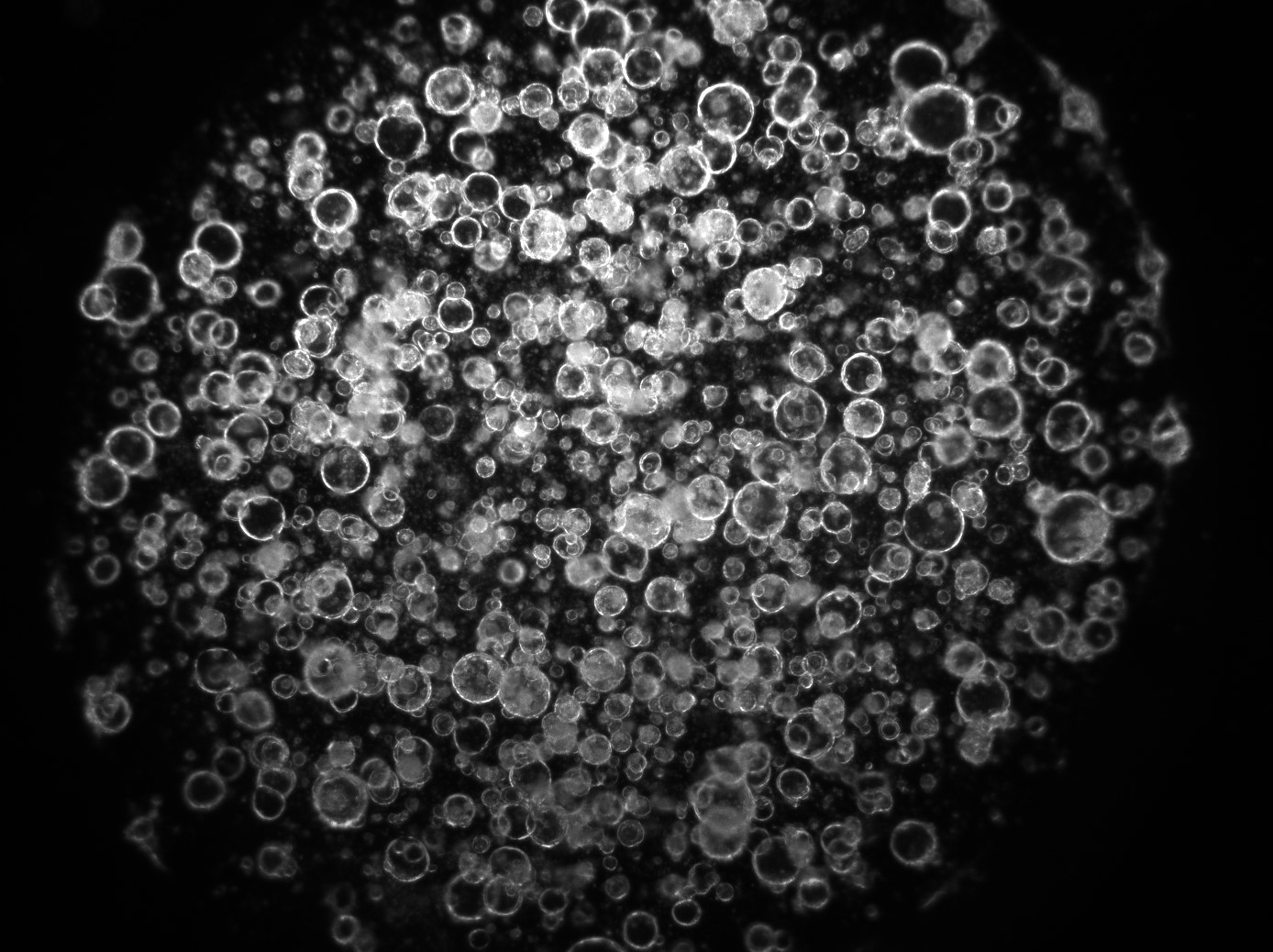

Supplement: Supplementary file 4 — Source Data [file 41467_2024_45605_MOESM4_ESM.zip › Source Data/Figures_Source_Data/figure 1/panel b/B1.jpg]

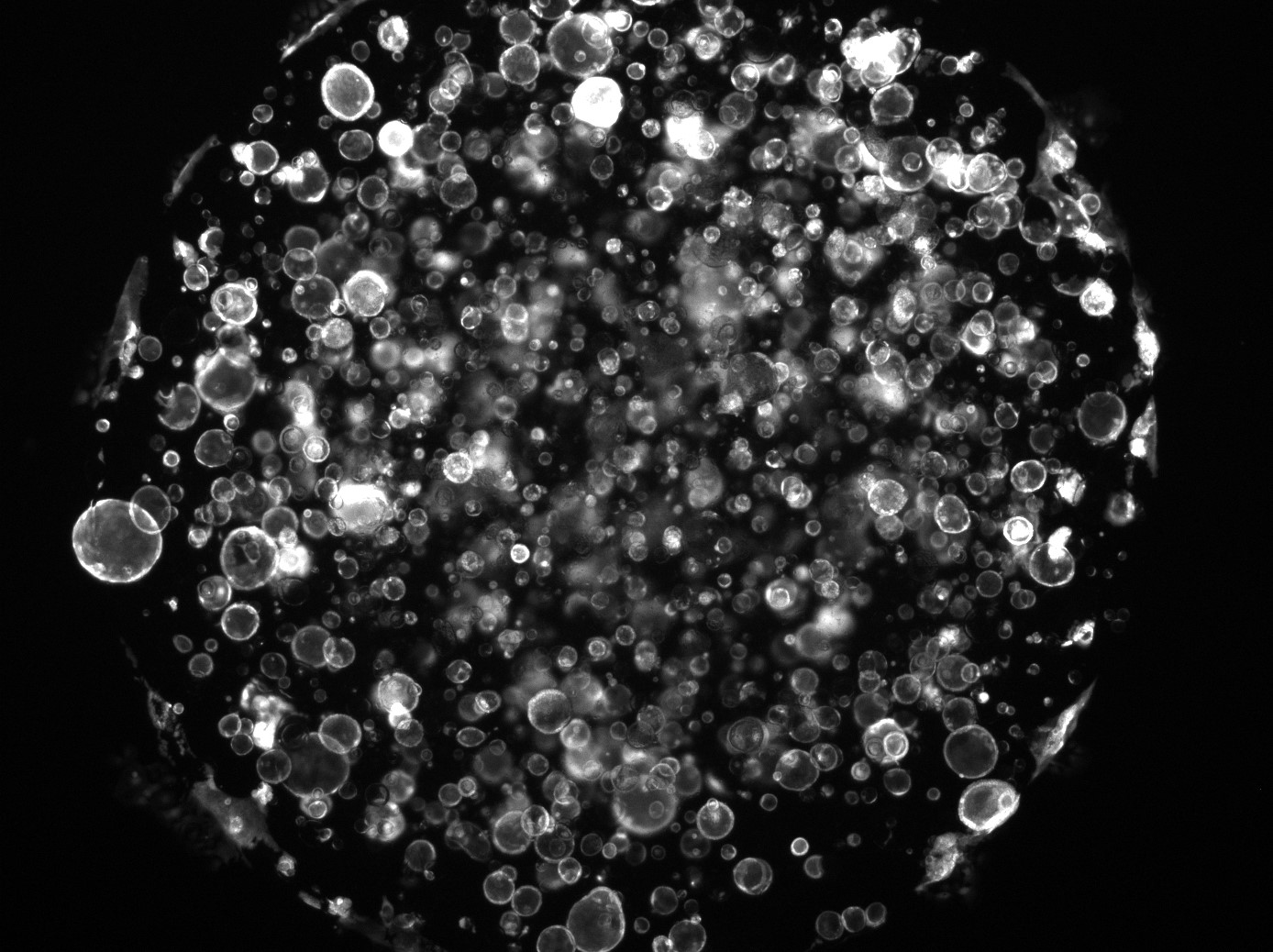

Supplement: Supplementary file 4 — Source Data [file 41467_2024_45605_MOESM4_ESM.zip › Source Data/Figures_Source_Data/figure 3/panel b/B13_TdTomato.jpg]

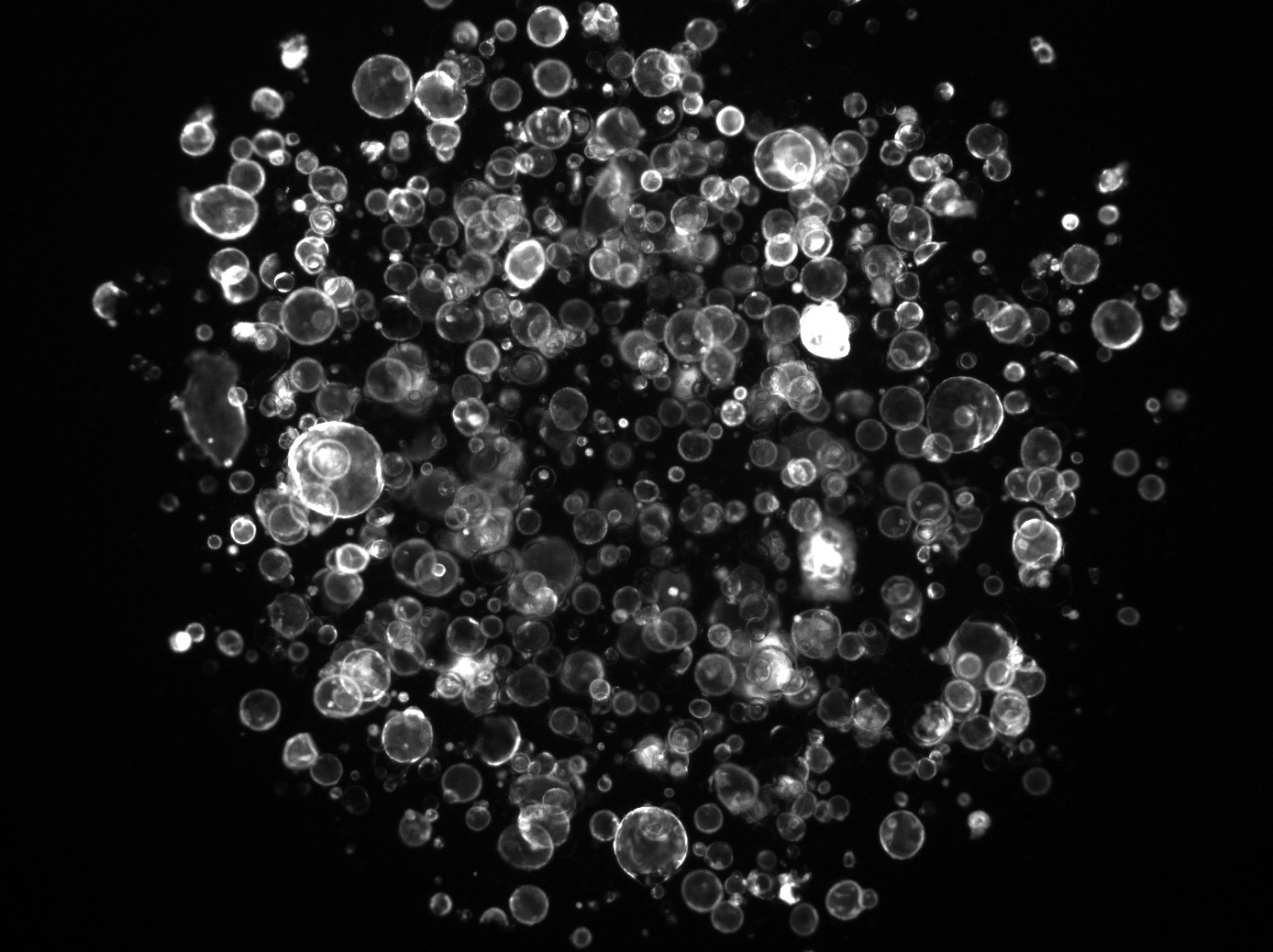

Supplement: Supplementary file 4 — Source Data [file 41467_2024_45605_MOESM4_ESM.zip › Source Data/Figures_Source_Data/figure 3/panel b/B14_TdTomato.jpg]

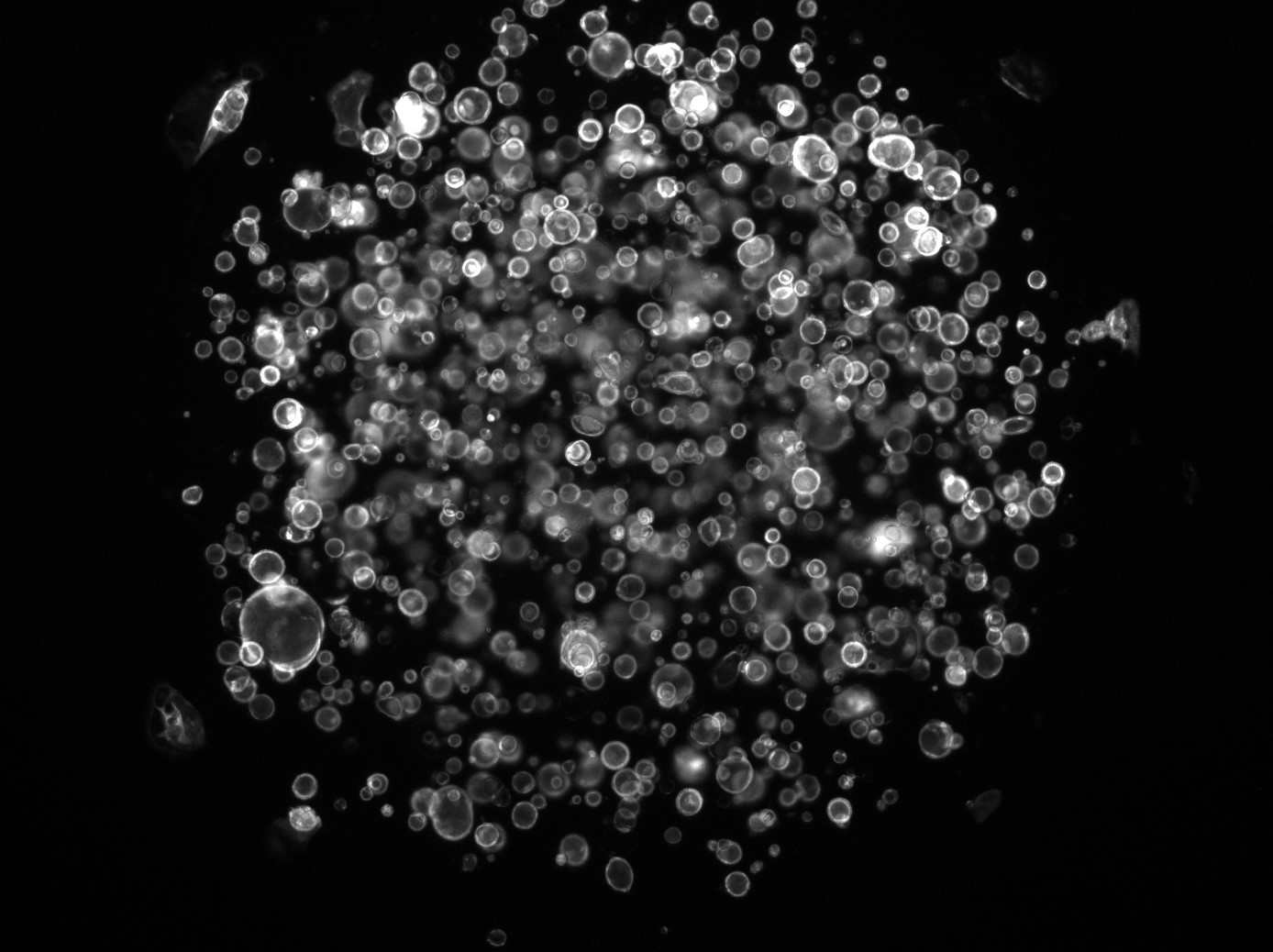

Supplement: Supplementary file 4 — Source Data [file 41467_2024_45605_MOESM4_ESM.zip › Source Data/Figures_Source_Data/figure 3/panel b/B30_TdTomato.jpg]

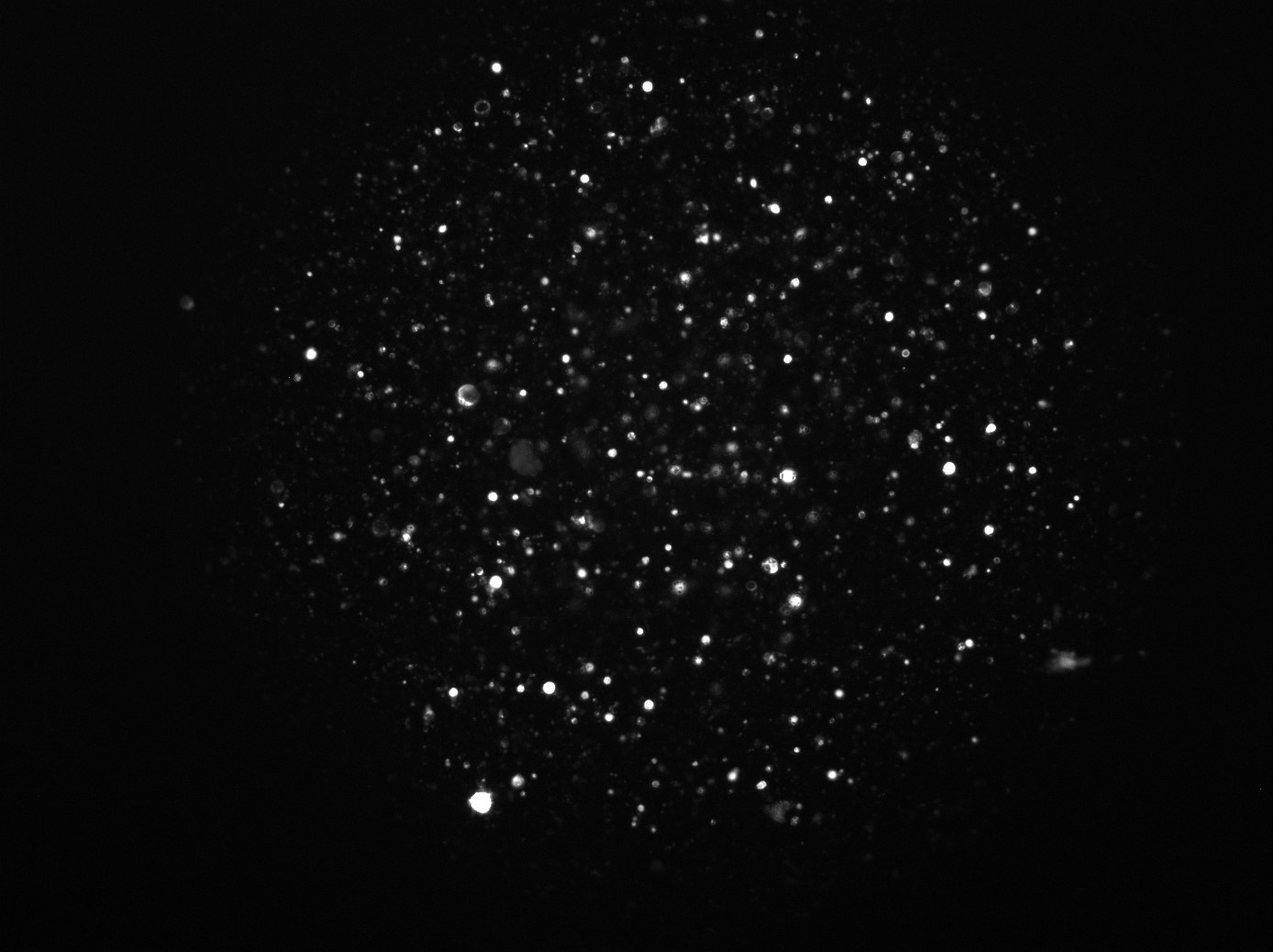

Supplement: Supplementary file 4 — Source Data [file 41467_2024_45605_MOESM4_ESM.zip › Source Data/Figures_Source_Data/figure 3/panel b/B22_TdTomato.jpg]

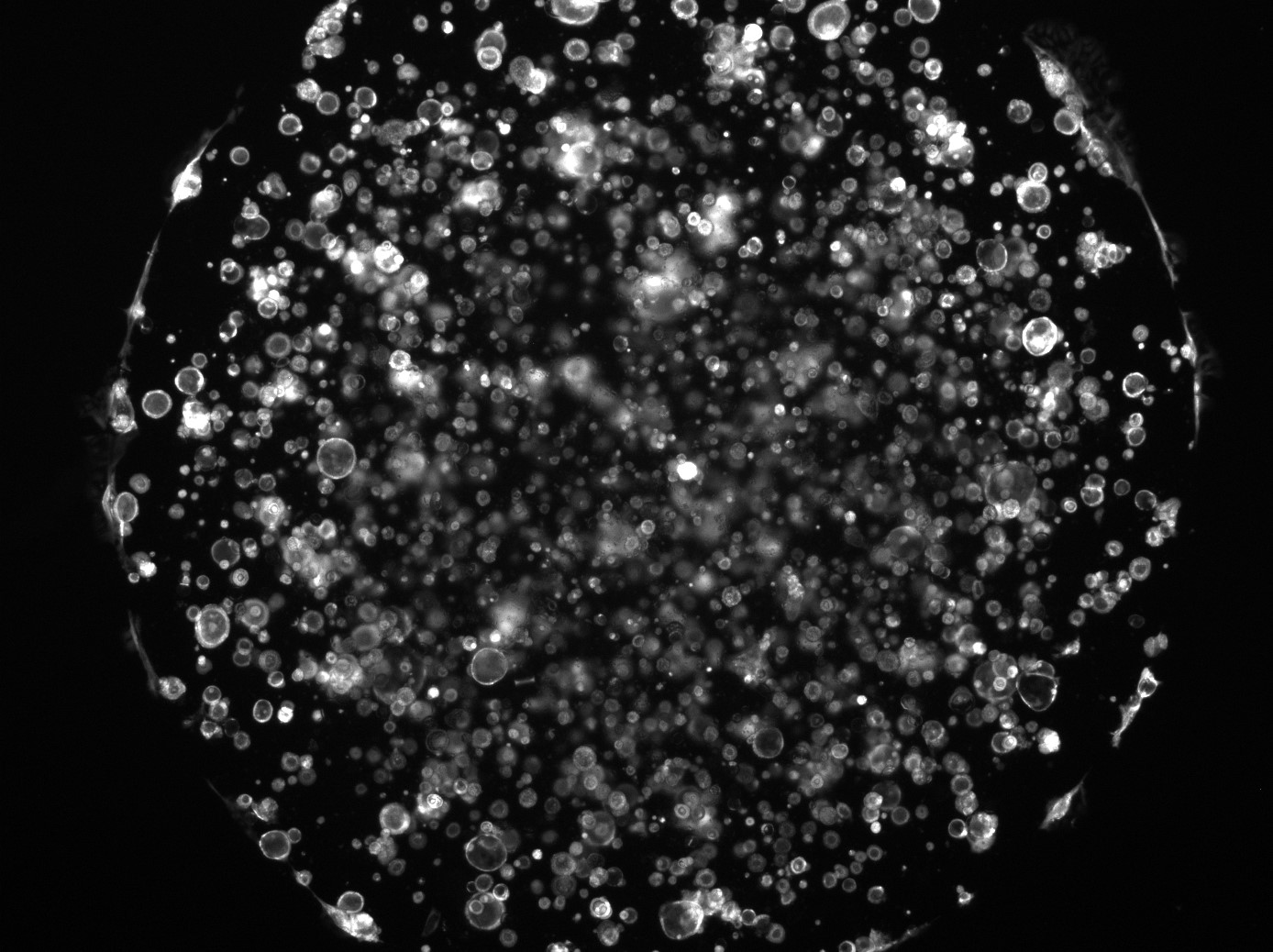

Supplement: Supplementary file 4 — Source Data [file 41467_2024_45605_MOESM4_ESM.zip › Source Data/Figures_Source_Data/figure 3/panel b/B15_TdTomato.jpg]

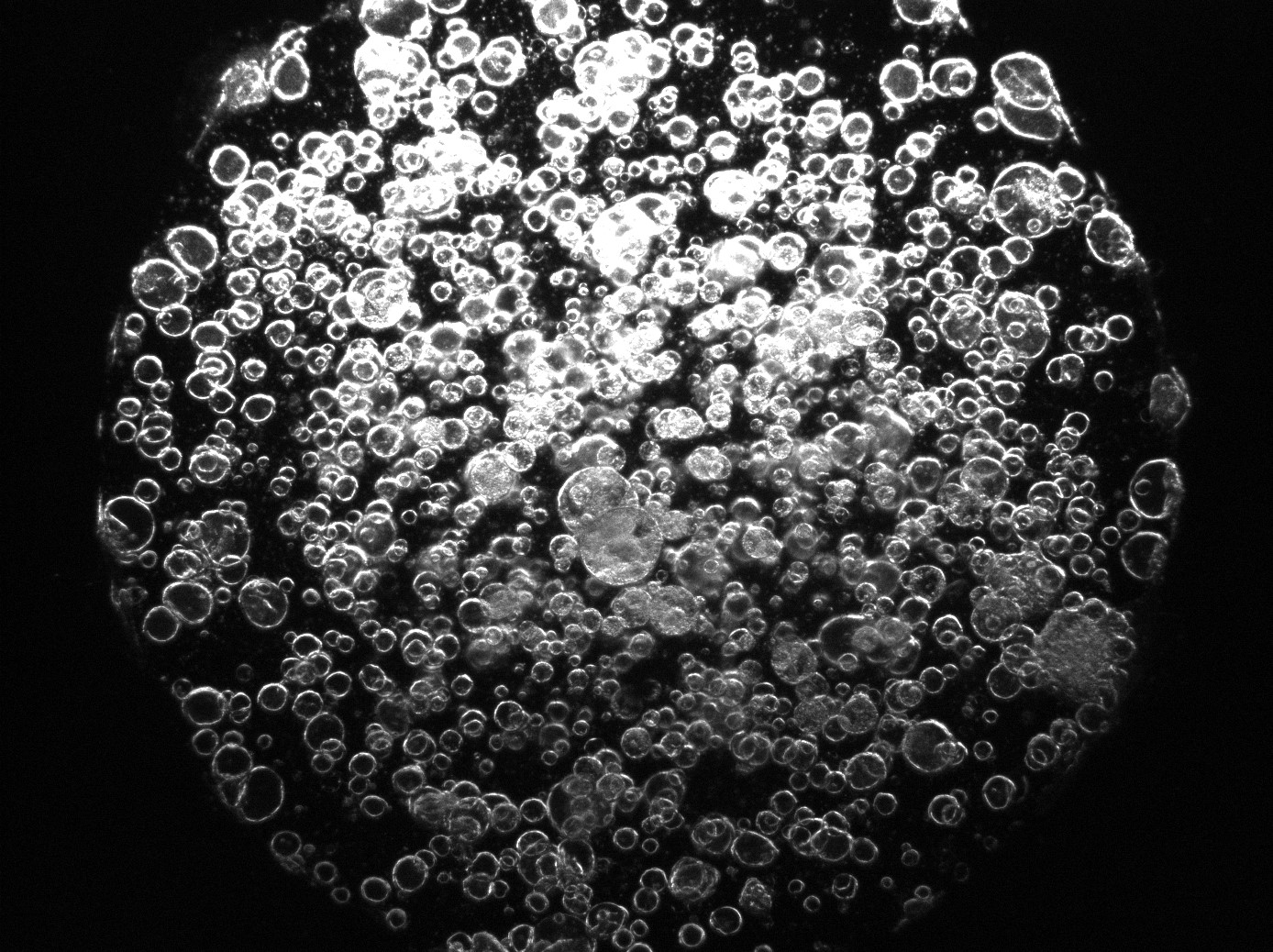

Supplement: Supplementary file 4 — Source Data [file 41467_2024_45605_MOESM4_ESM.zip › Source Data/Figures_Source_Data/figure 3/panel b/B9.jpg]

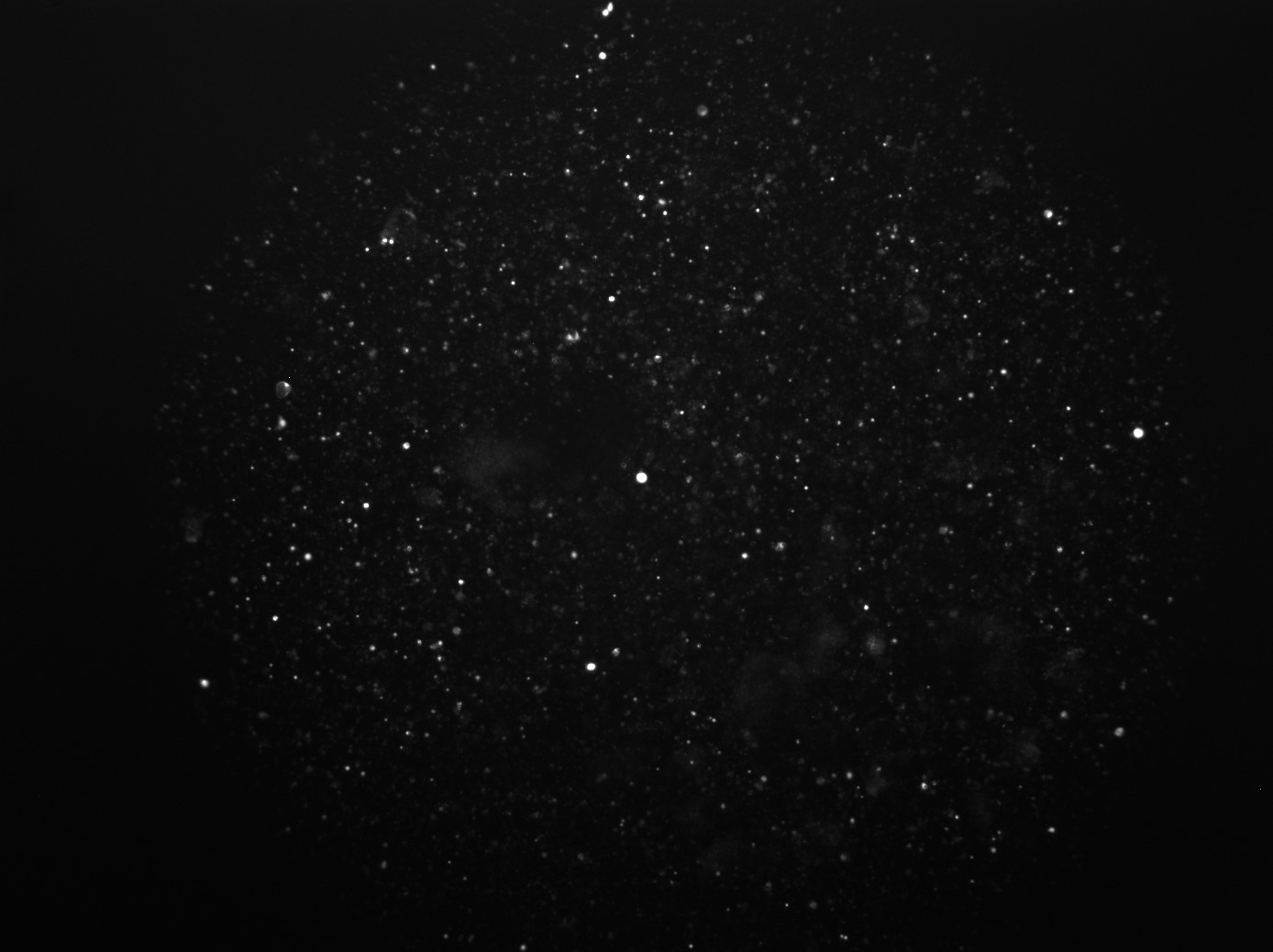

Supplement: Supplementary file 4 — Source Data [file 41467_2024_45605_MOESM4_ESM.zip › Source Data/Figures_Source_Data/figure 3/panel b/B24_TdTomato.jpg]

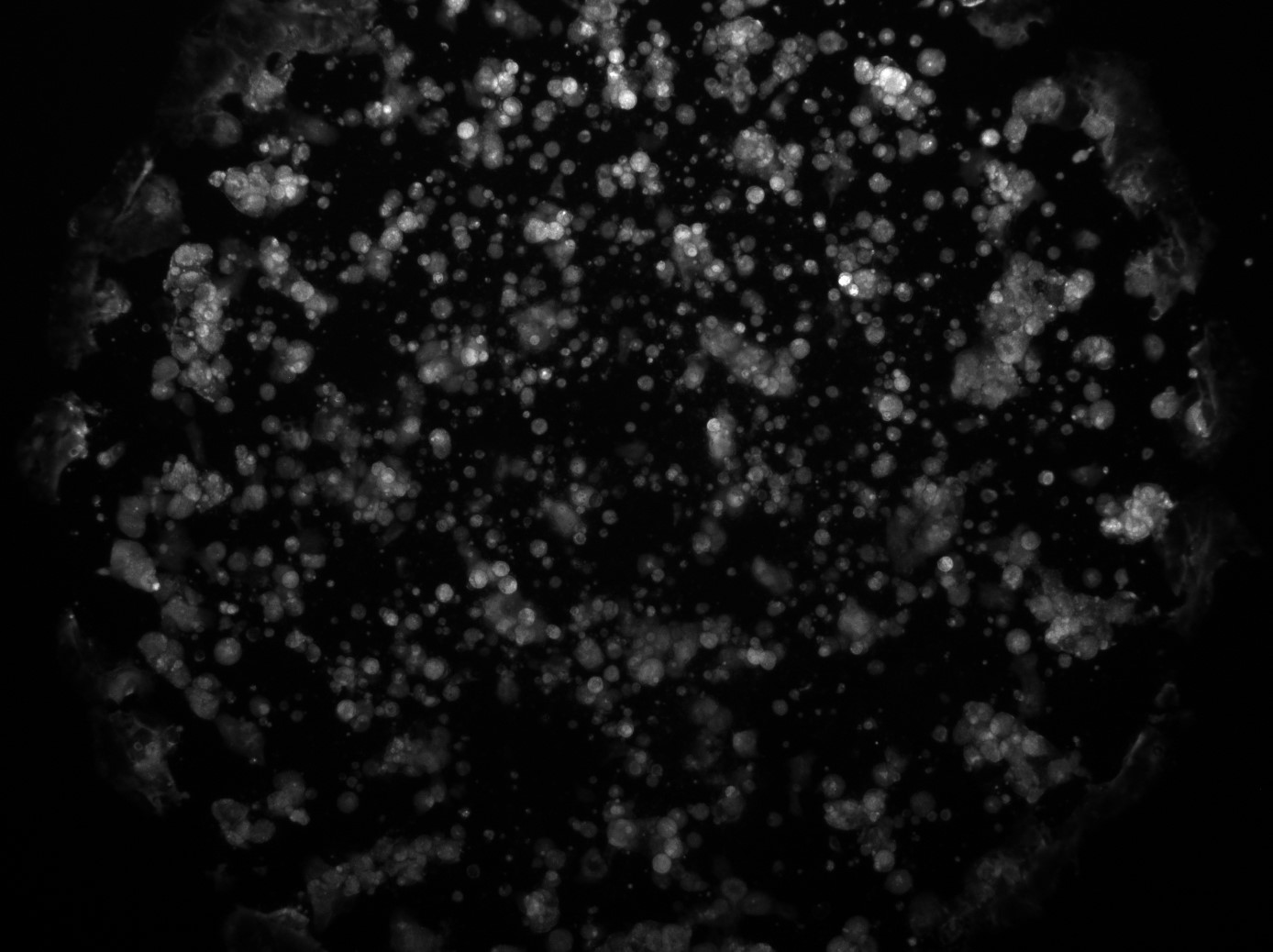

Supplement: Supplementary file 4 — Source Data [file 41467_2024_45605_MOESM4_ESM.zip › Source Data/Figures_Source_Data/figure 3/panel b/B23_TdTomato.jpg]

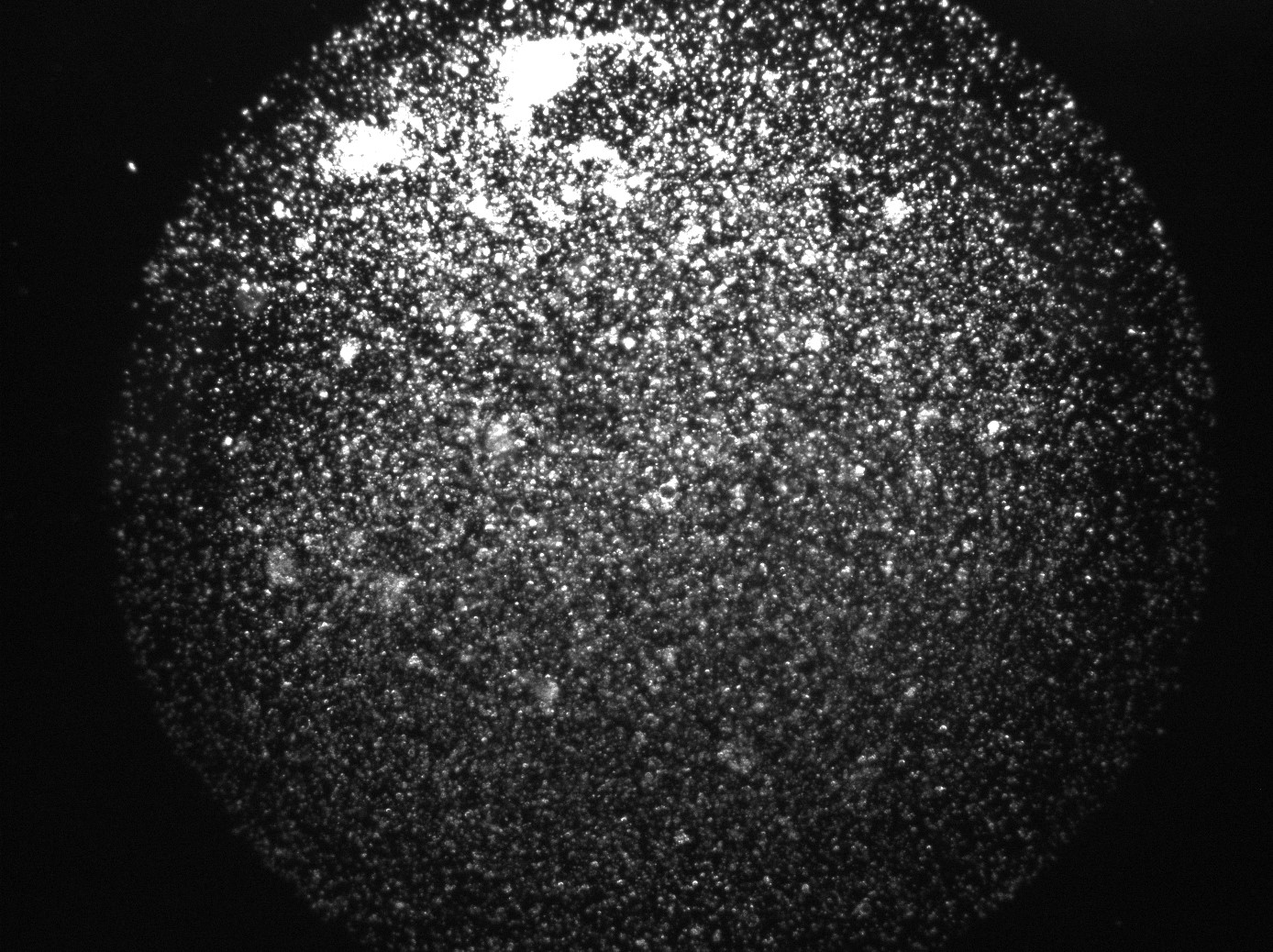

Supplement: Supplementary file 4 — Source Data [file 41467_2024_45605_MOESM4_ESM.zip › Source Data/Figures_Source_Data/figure 3/panel b/B8.jpg]

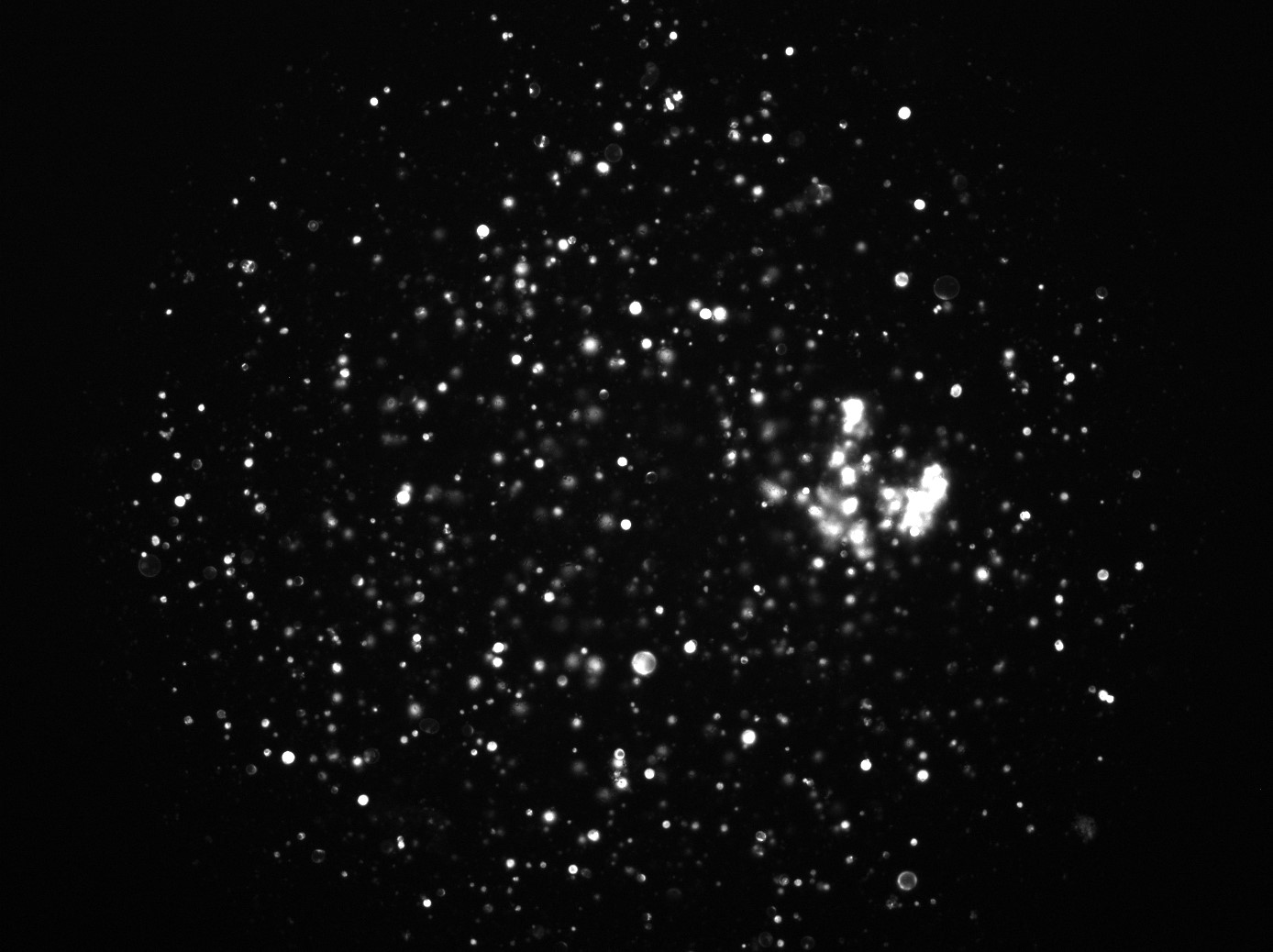

Supplement: Supplementary file 4 — Source Data [file 41467_2024_45605_MOESM4_ESM.zip › Source Data/Figures_Source_Data/figure 3/panel b/B6_TdTomato.jpg]

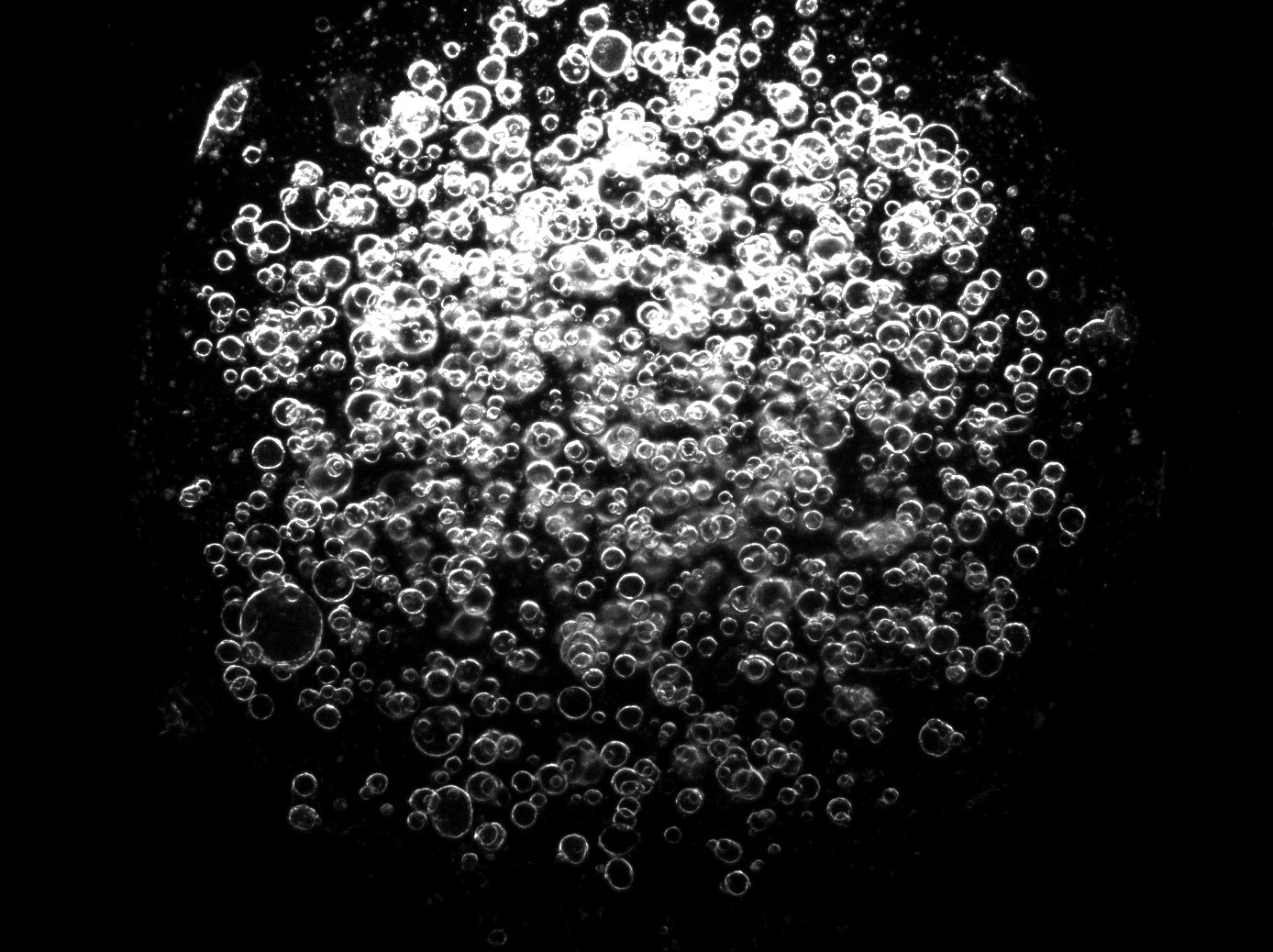

Supplement: Supplementary file 4 — Source Data [file 41467_2024_45605_MOESM4_ESM.zip › Source Data/Figures_Source_Data/figure 3/panel b/B30.jpg]

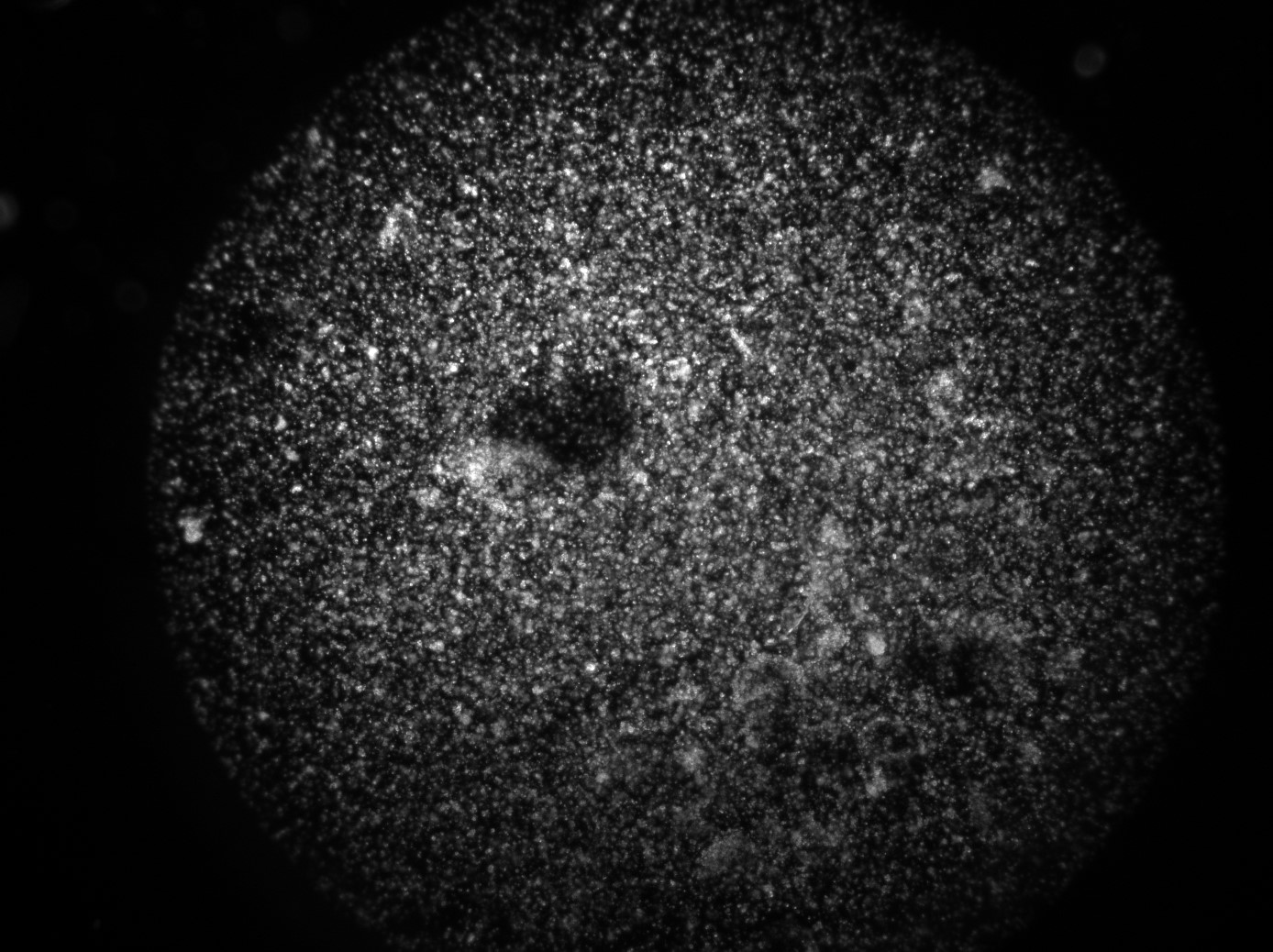

Supplement: Supplementary file 4 — Source Data [file 41467_2024_45605_MOESM4_ESM.zip › Source Data/Figures_Source_Data/figure 3/panel b/B24.jpg]

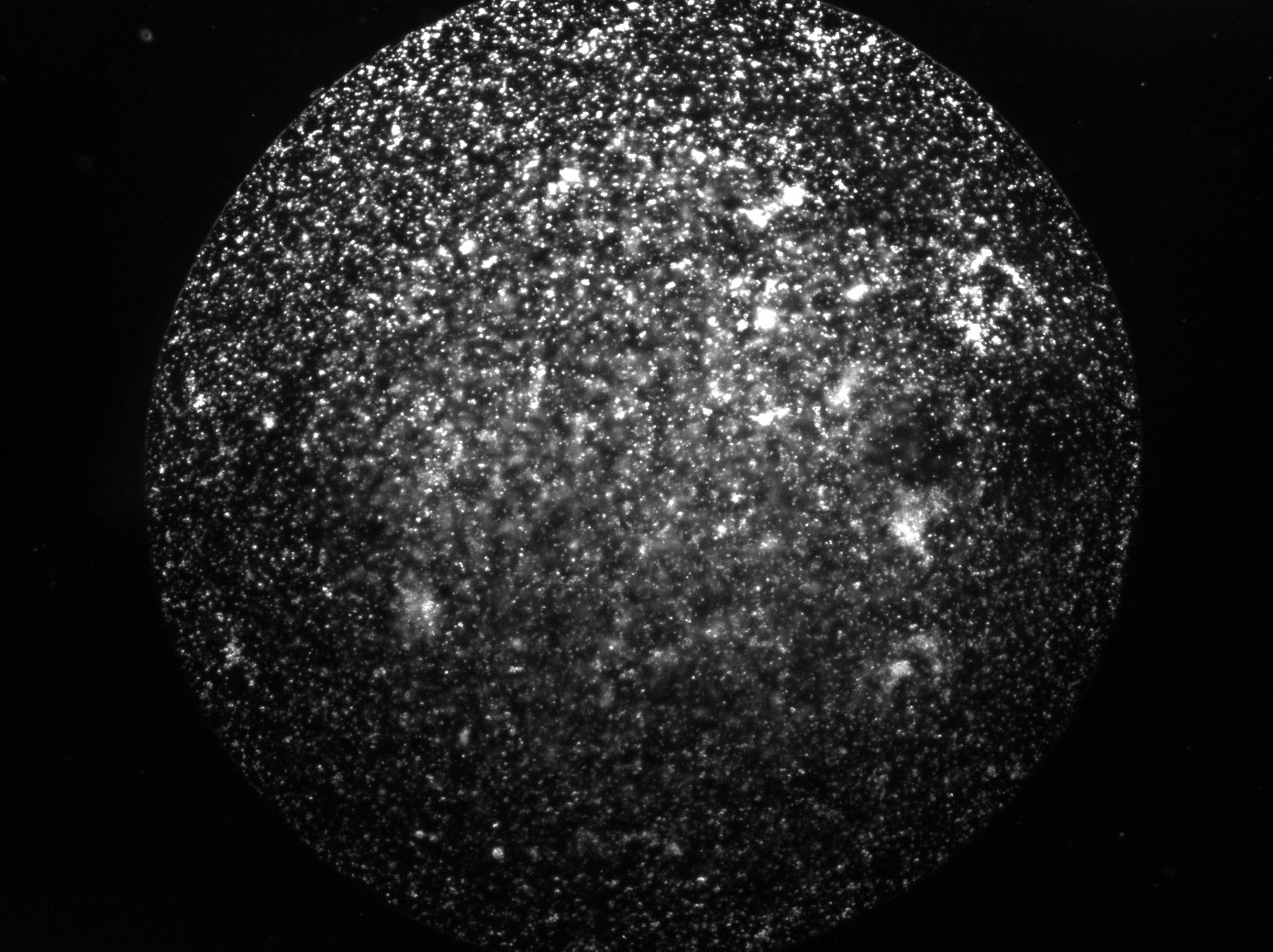

Supplement: Supplementary file 4 — Source Data [file 41467_2024_45605_MOESM4_ESM.zip › Source Data/Figures_Source_Data/figure 3/panel b/B18.jpg]

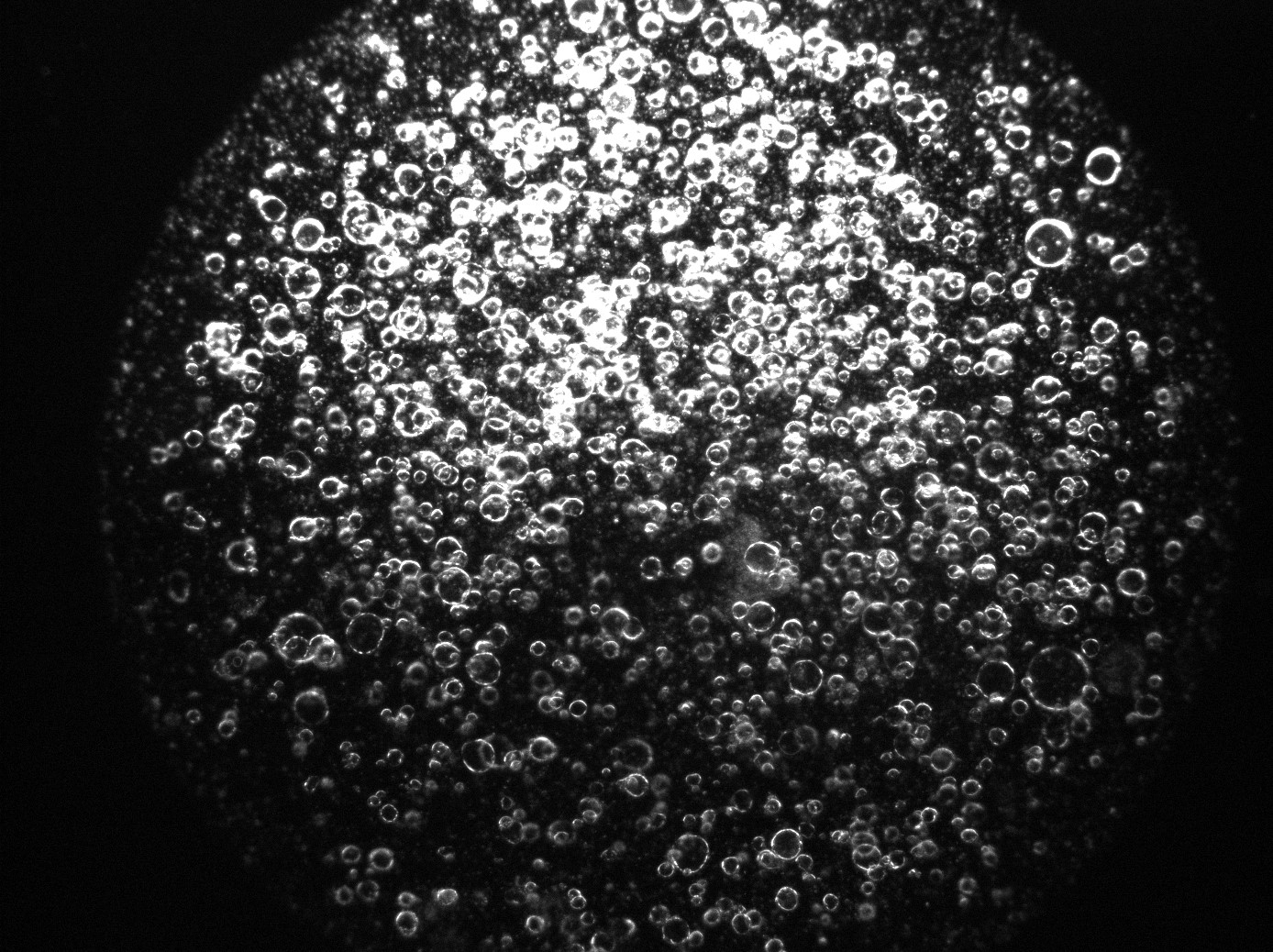

Supplement: Supplementary file 4 — Source Data [file 41467_2024_45605_MOESM4_ESM.zip › Source Data/Figures_Source_Data/figure 3/panel b/B19.jpg]

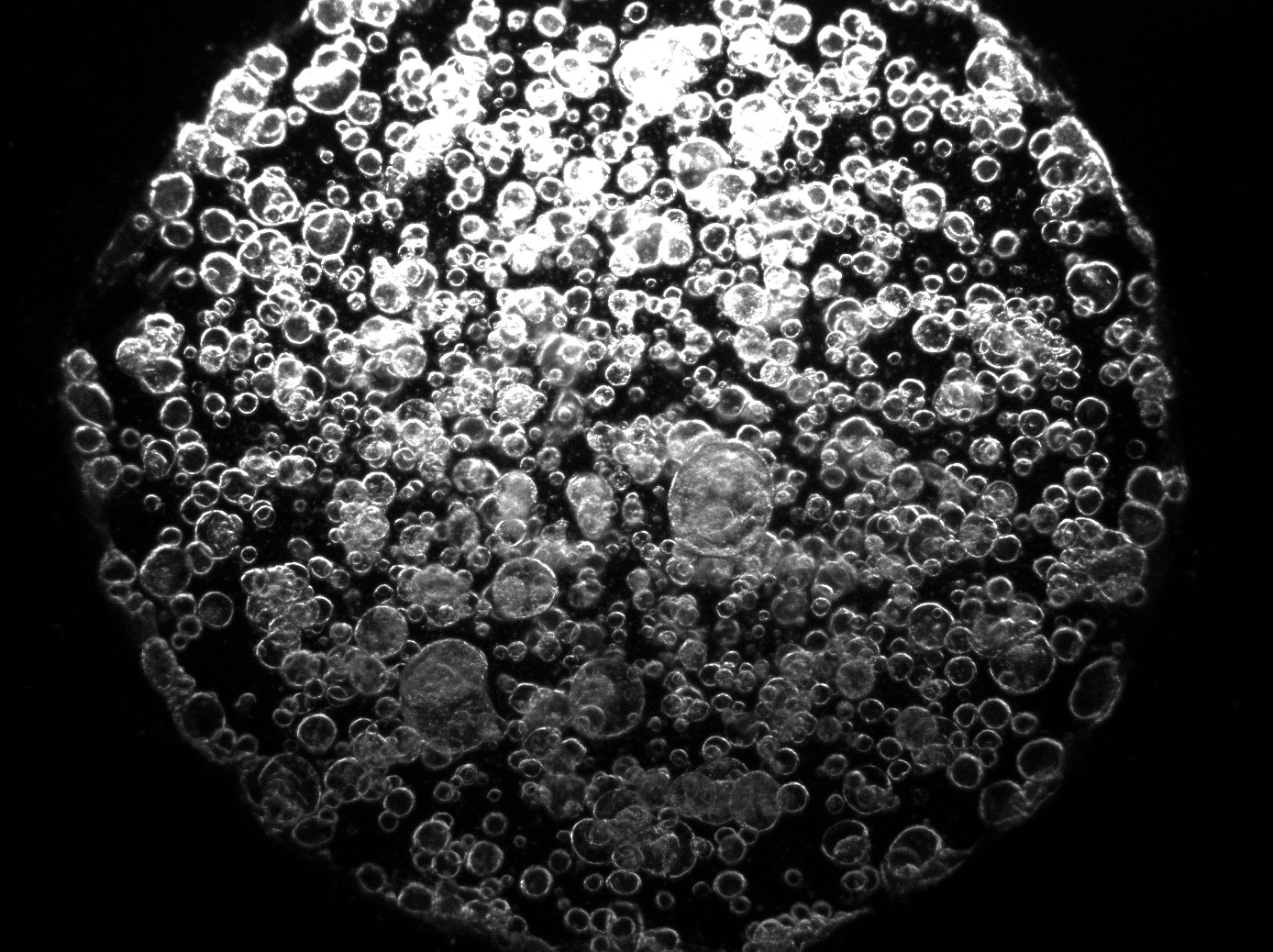

Supplement: Supplementary file 4 — Source Data [file 41467_2024_45605_MOESM4_ESM.zip › Source Data/Figures_Source_Data/figure 3/panel b/B25.jpg]

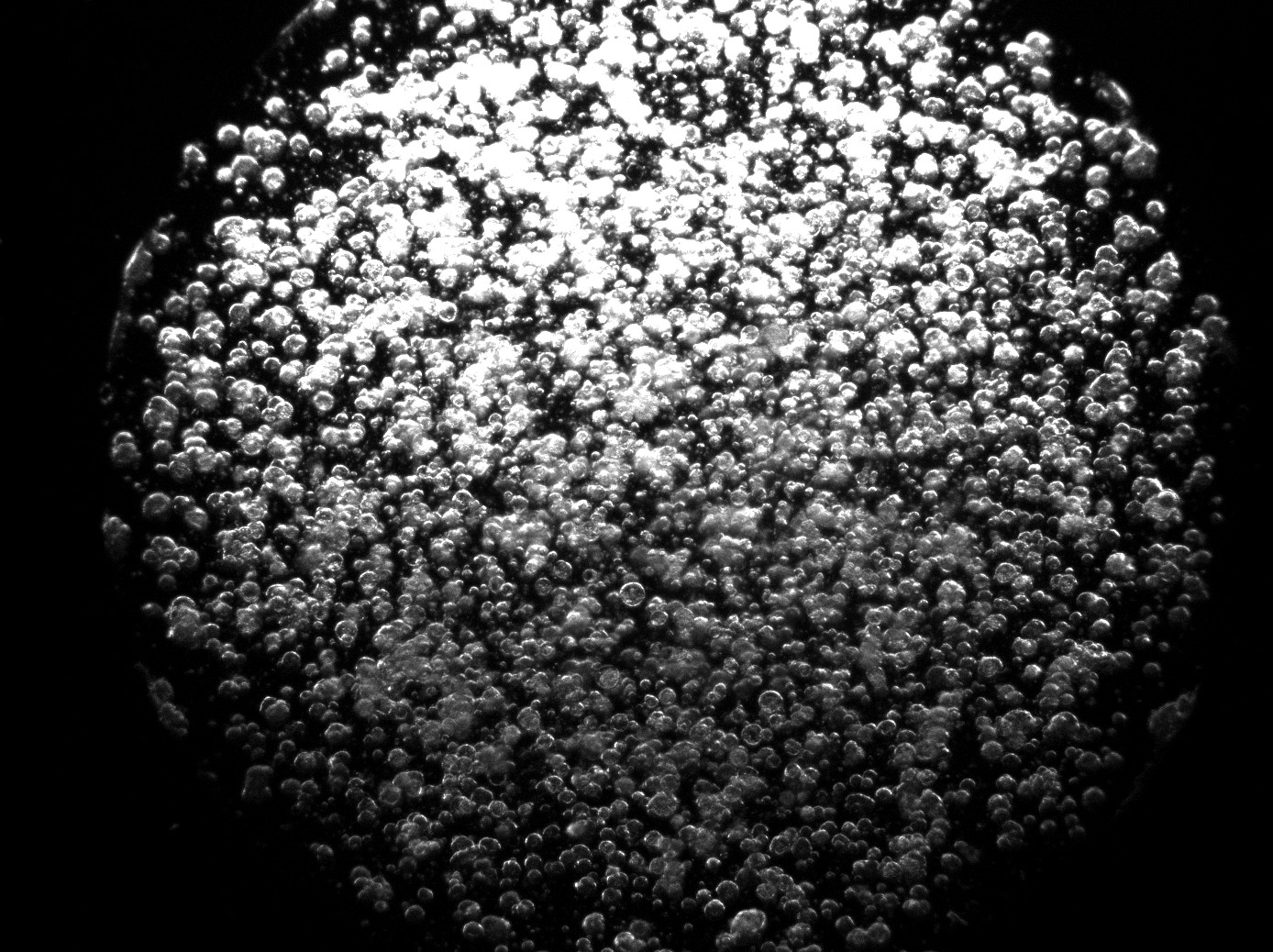

Supplement: Supplementary file 4 — Source Data [file 41467_2024_45605_MOESM4_ESM.zip › Source Data/Figures_Source_Data/figure 3/panel b/B31.jpg]

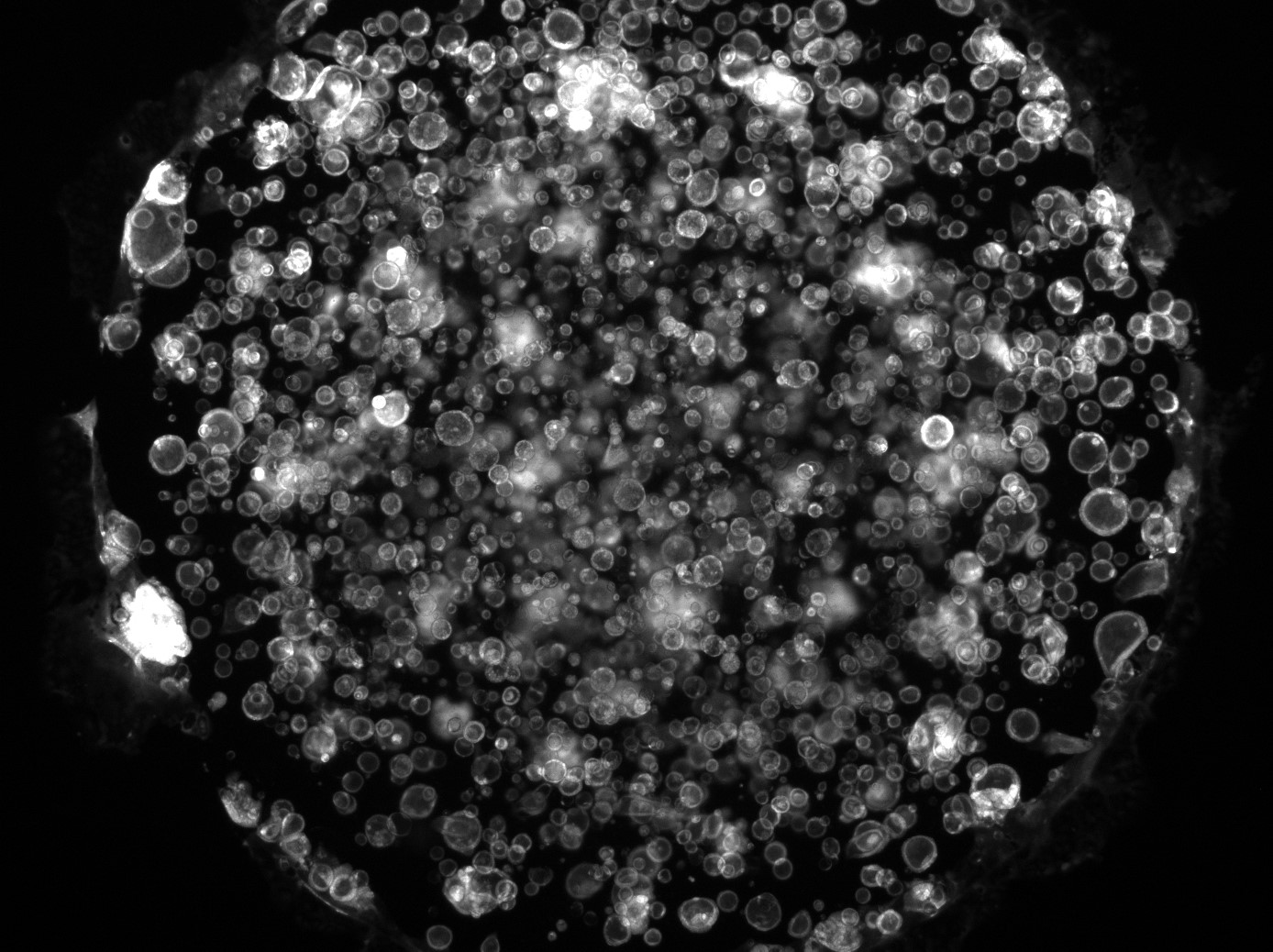

Supplement: Supplementary file 4 — Source Data [file 41467_2024_45605_MOESM4_ESM.zip › Source Data/Figures_Source_Data/figure 3/panel b/B29_TdTomato.jpg]

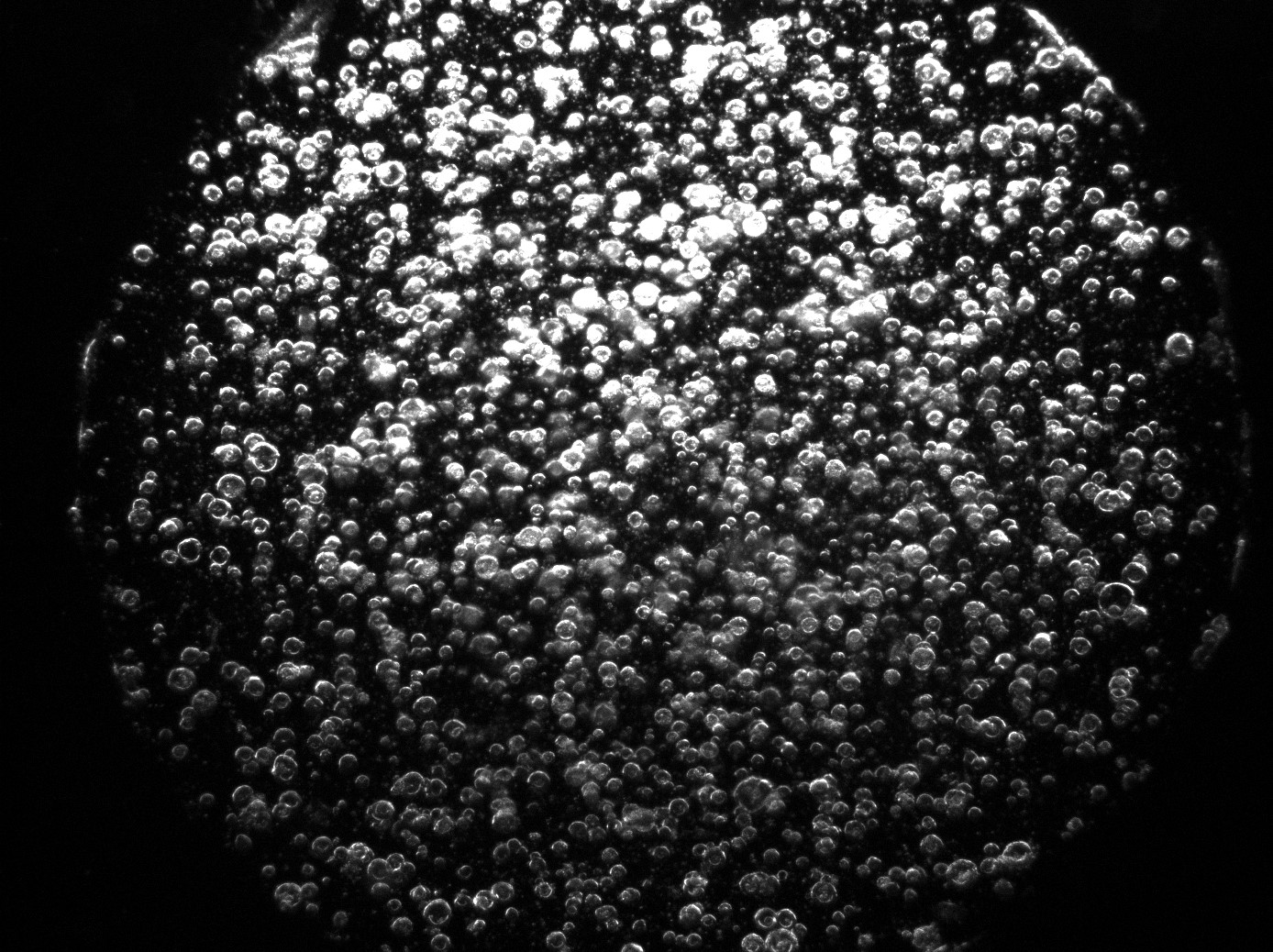

Supplement: Supplementary file 4 — Source Data [file 41467_2024_45605_MOESM4_ESM.zip › Source Data/Figures_Source_Data/figure 3/panel b/B27.jpg]

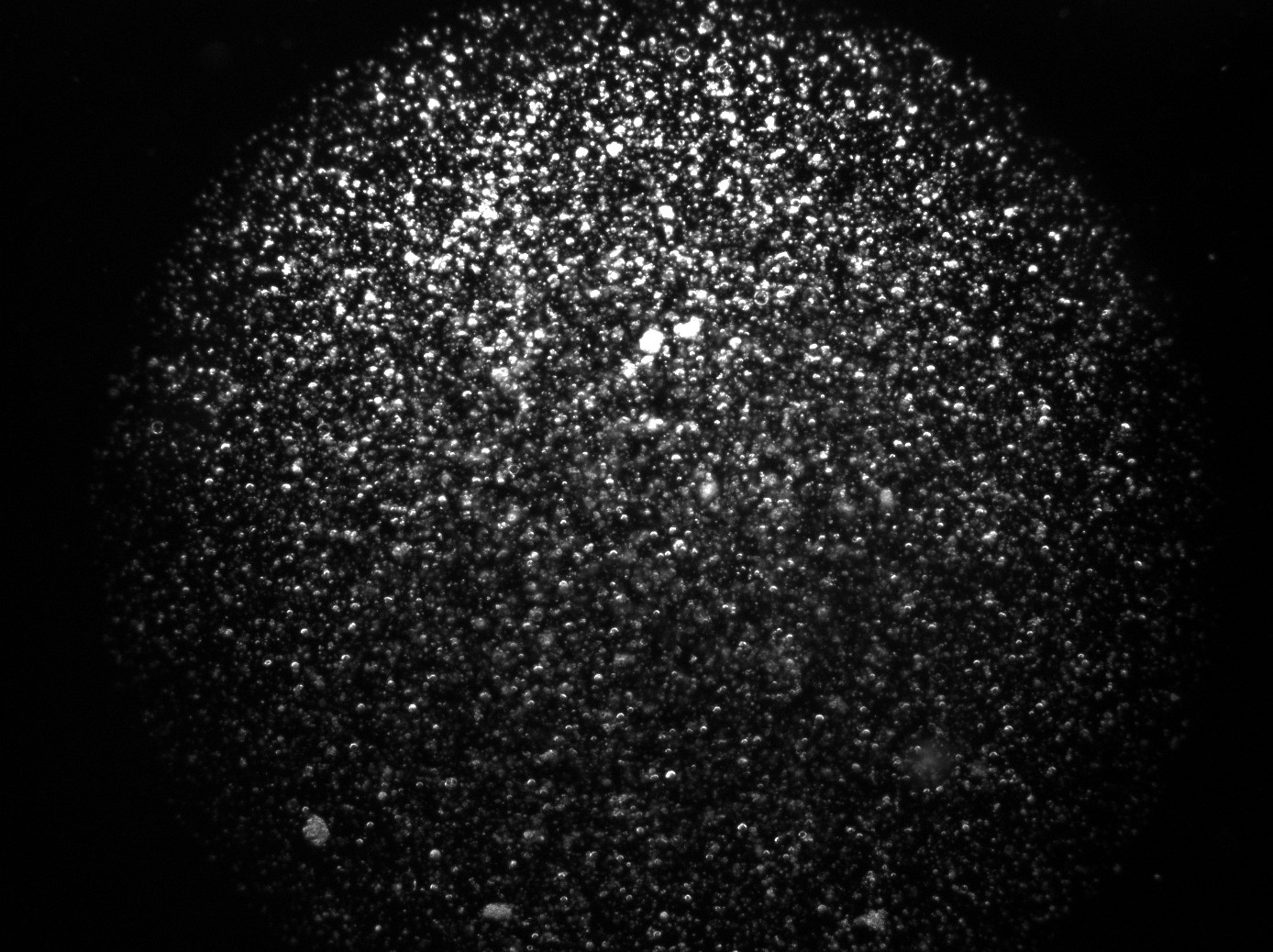

Supplement: Supplementary file 4 — Source Data [file 41467_2024_45605_MOESM4_ESM.zip › Source Data/Figures_Source_Data/figure 3/panel b/B32.jpg]

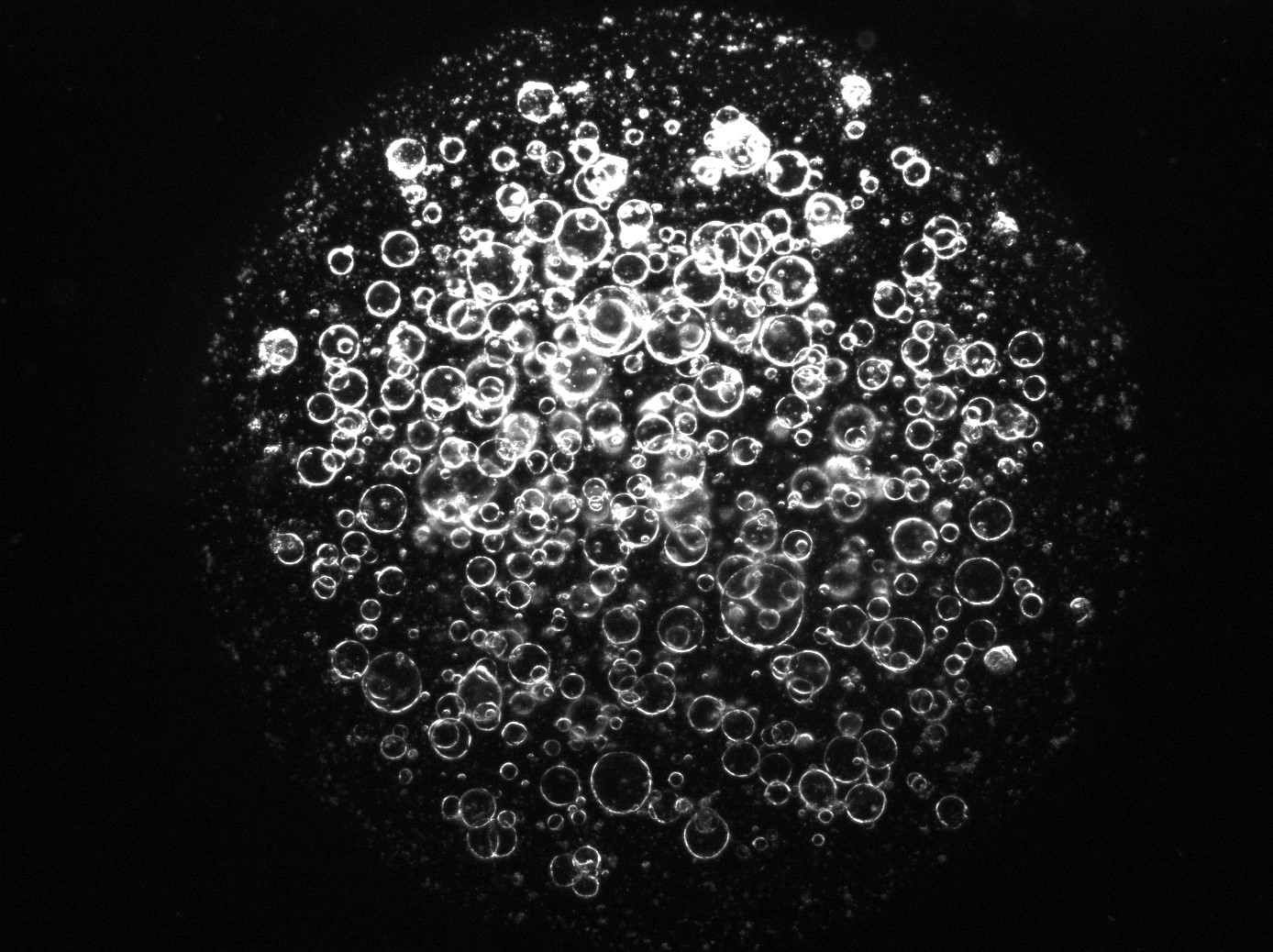

Supplement: Supplementary file 4 — Source Data [file 41467_2024_45605_MOESM4_ESM.zip › Source Data/Figures_Source_Data/figure 3/panel b/B26.jpg]

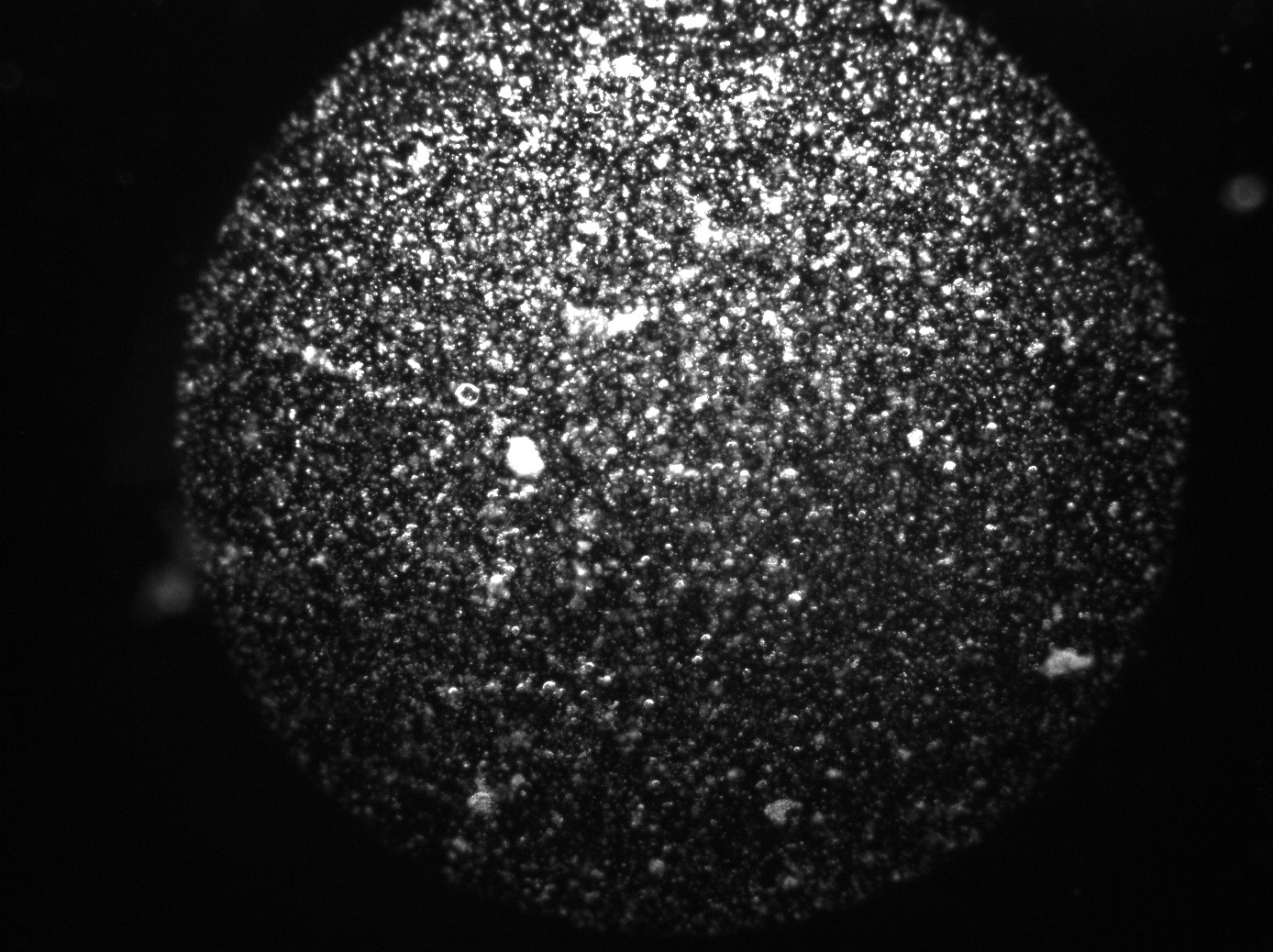

Supplement: Supplementary file 4 — Source Data [file 41467_2024_45605_MOESM4_ESM.zip › Source Data/Figures_Source_Data/figure 3/panel b/B22.jpg]

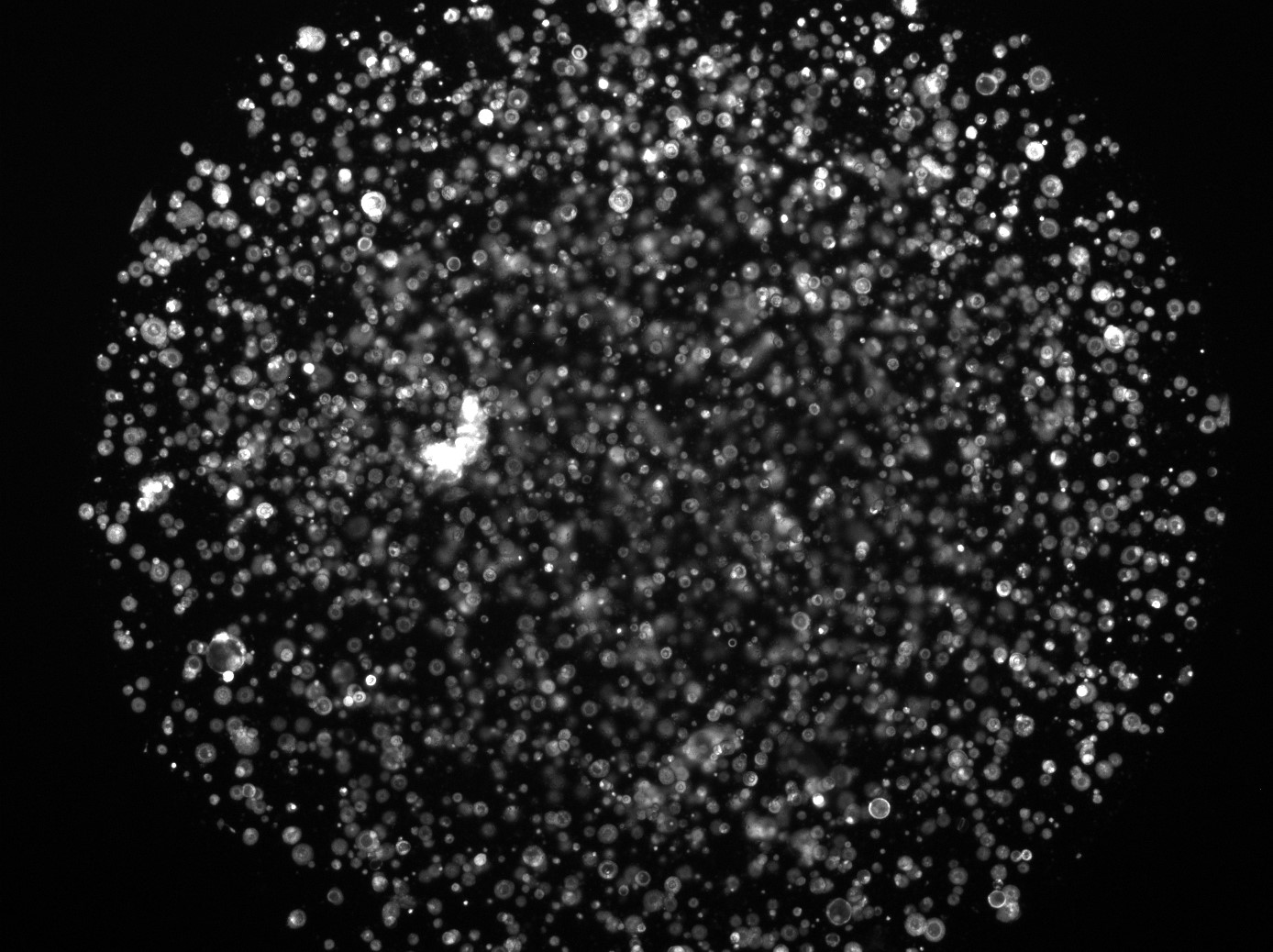

Supplement: Supplementary file 4 — Source Data [file 41467_2024_45605_MOESM4_ESM.zip › Source Data/Figures_Source_Data/figure 3/panel b/B7_TdTomato.jpg]

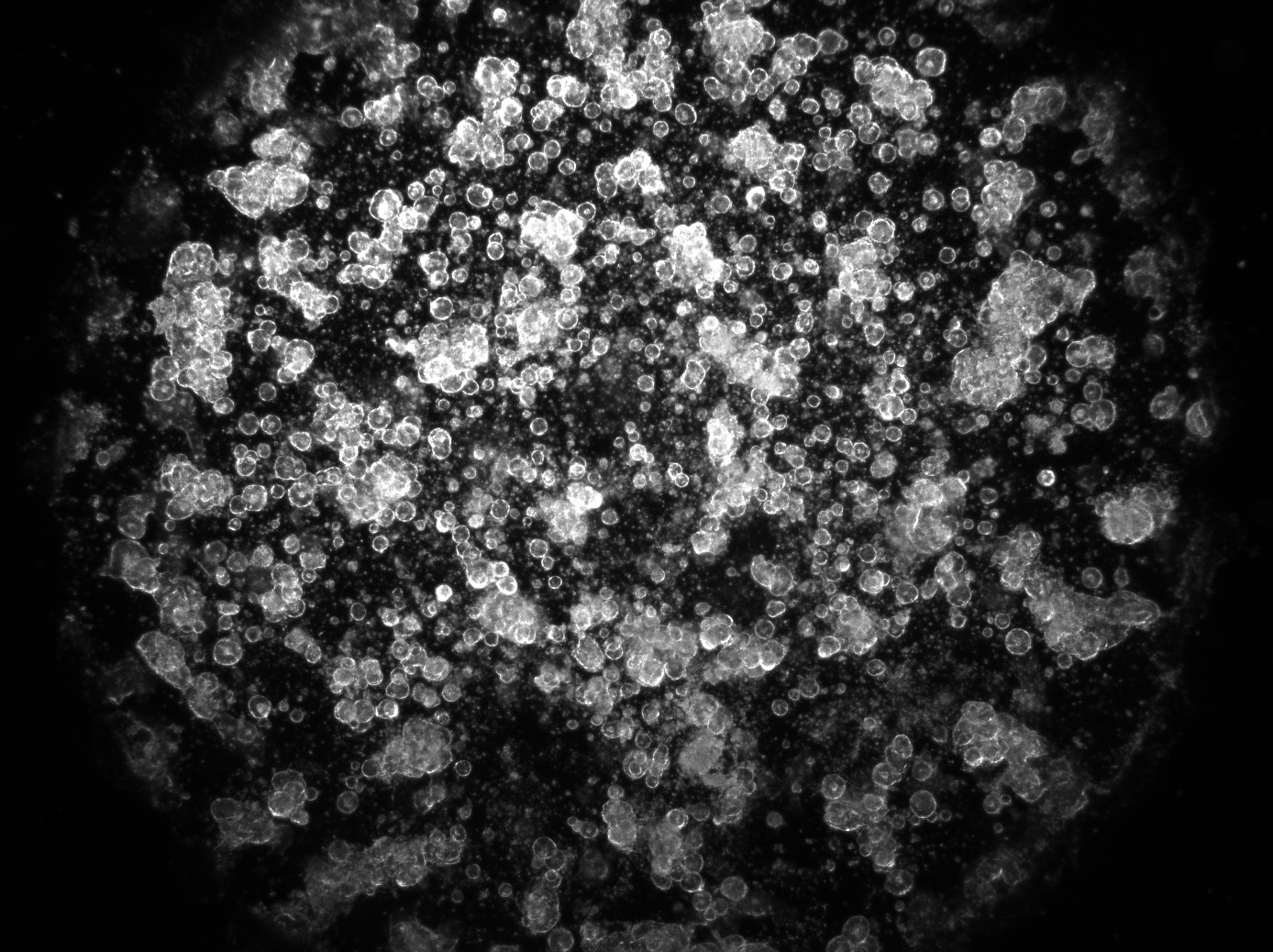

Supplement: Supplementary file 4 — Source Data [file 41467_2024_45605_MOESM4_ESM.zip › Source Data/Figures_Source_Data/figure 3/panel b/B23.jpg]

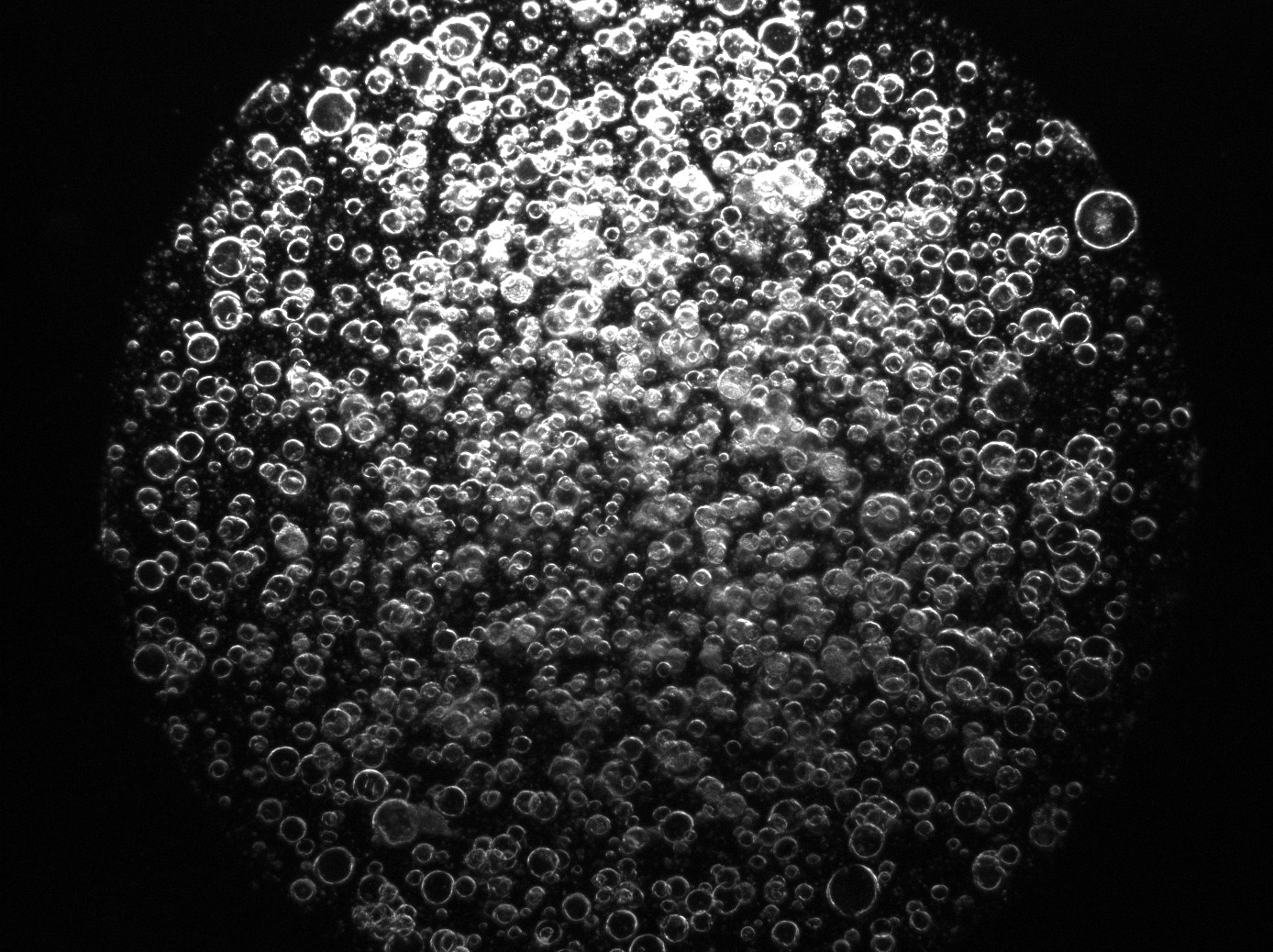

Supplement: Supplementary file 4 — Source Data [file 41467_2024_45605_MOESM4_ESM.zip › Source Data/Figures_Source_Data/figure 3/panel b/B21.jpg]

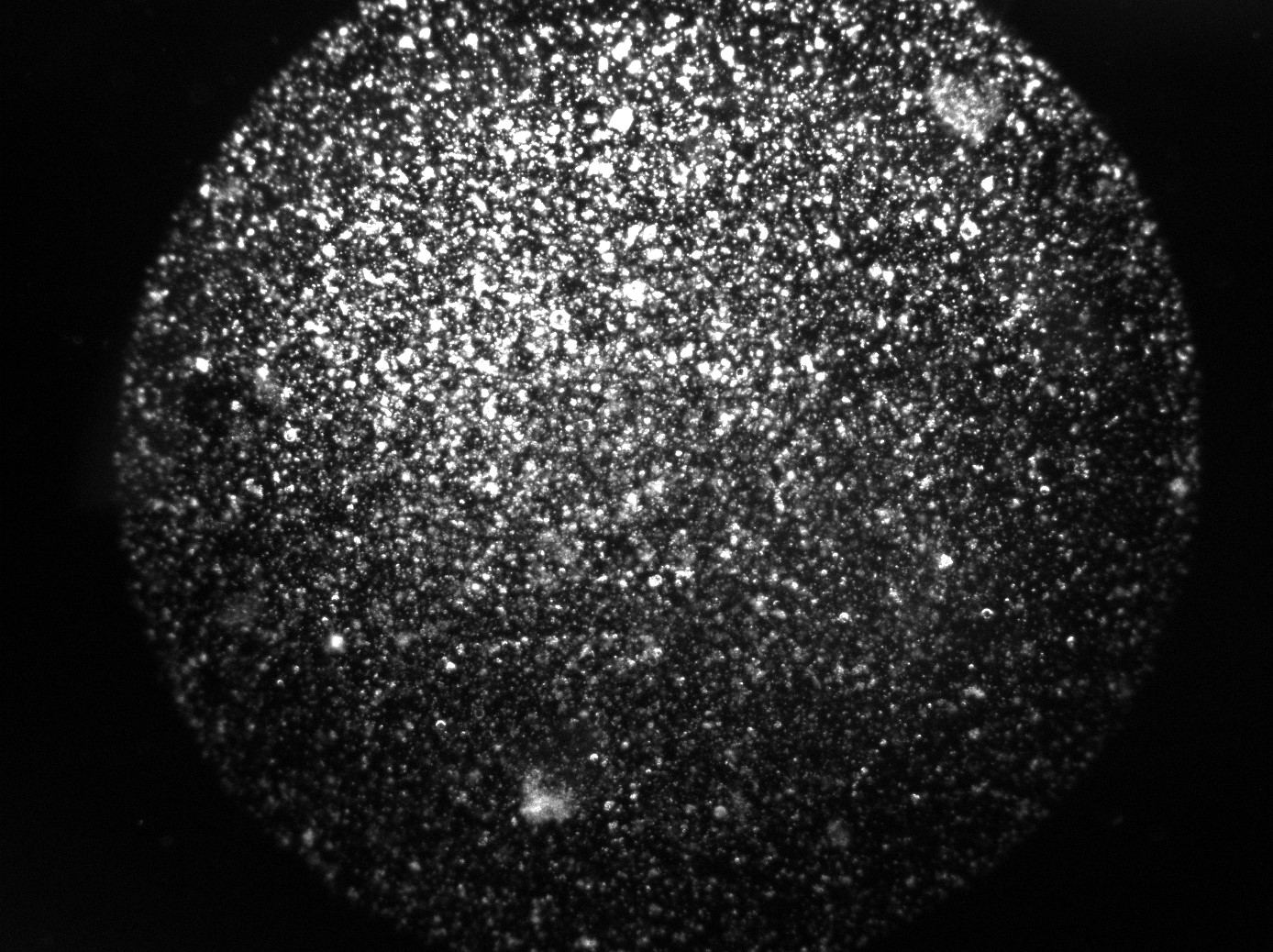

Supplement: Supplementary file 4 — Source Data [file 41467_2024_45605_MOESM4_ESM.zip › Source Data/Figures_Source_Data/figure 3/panel b/B20.jpg]

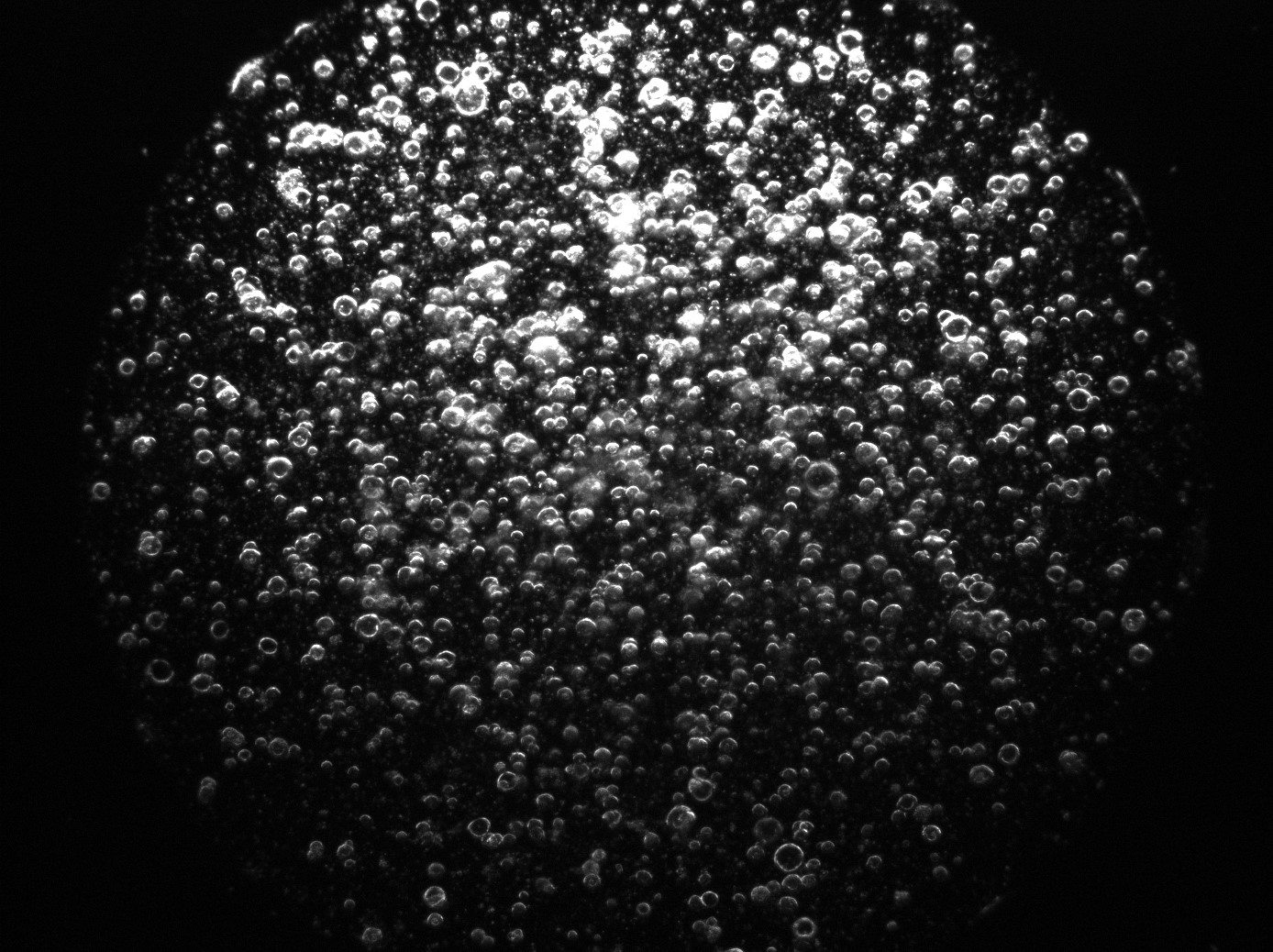

Supplement: Supplementary file 4 — Source Data [file 41467_2024_45605_MOESM4_ESM.zip › Source Data/Figures_Source_Data/figure 3/panel b/B11.jpg]

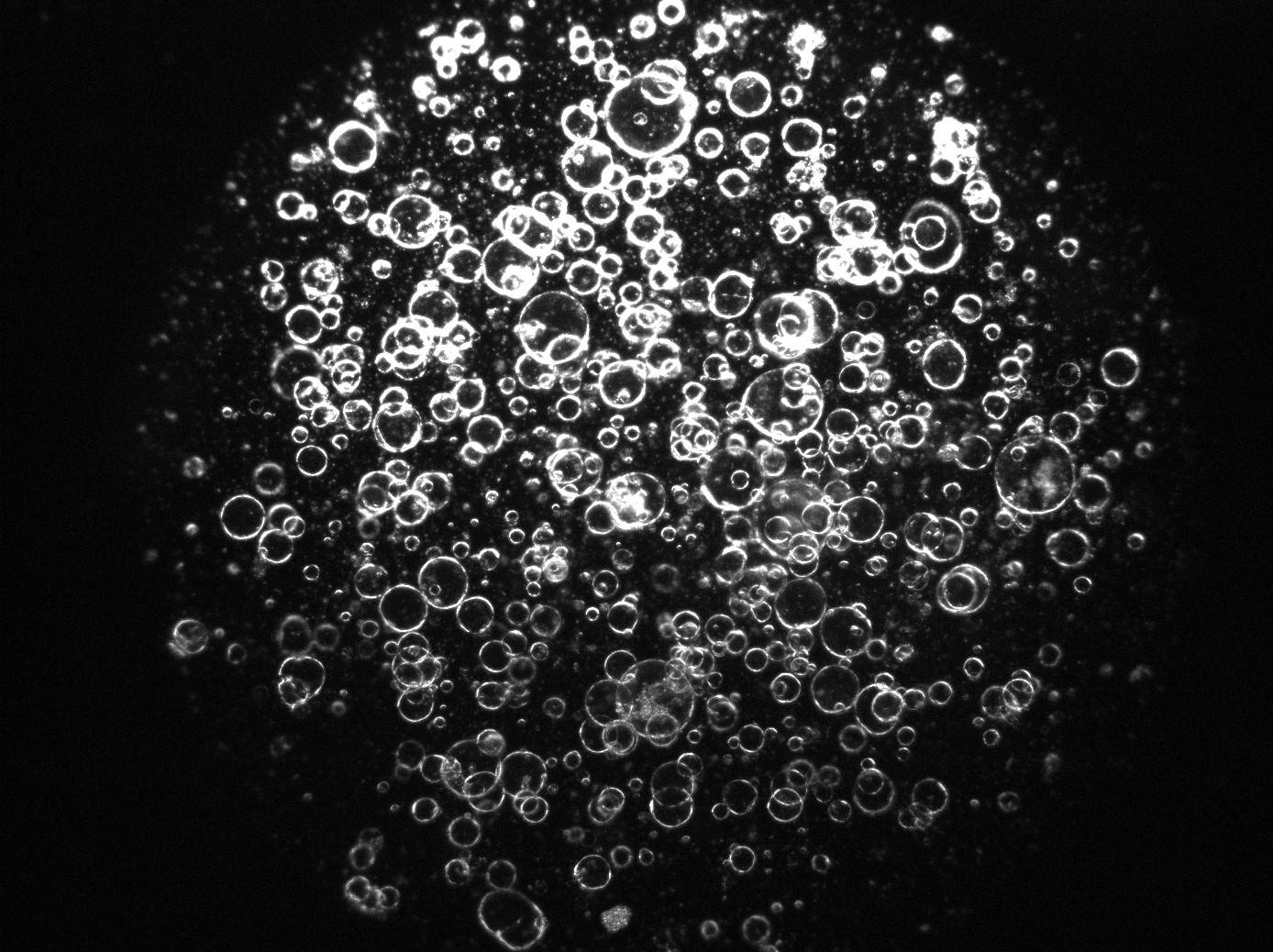

Supplement: Supplementary file 4 — Source Data [file 41467_2024_45605_MOESM4_ESM.zip › Source Data/Figures_Source_Data/figure 3/panel b/B10.jpg]

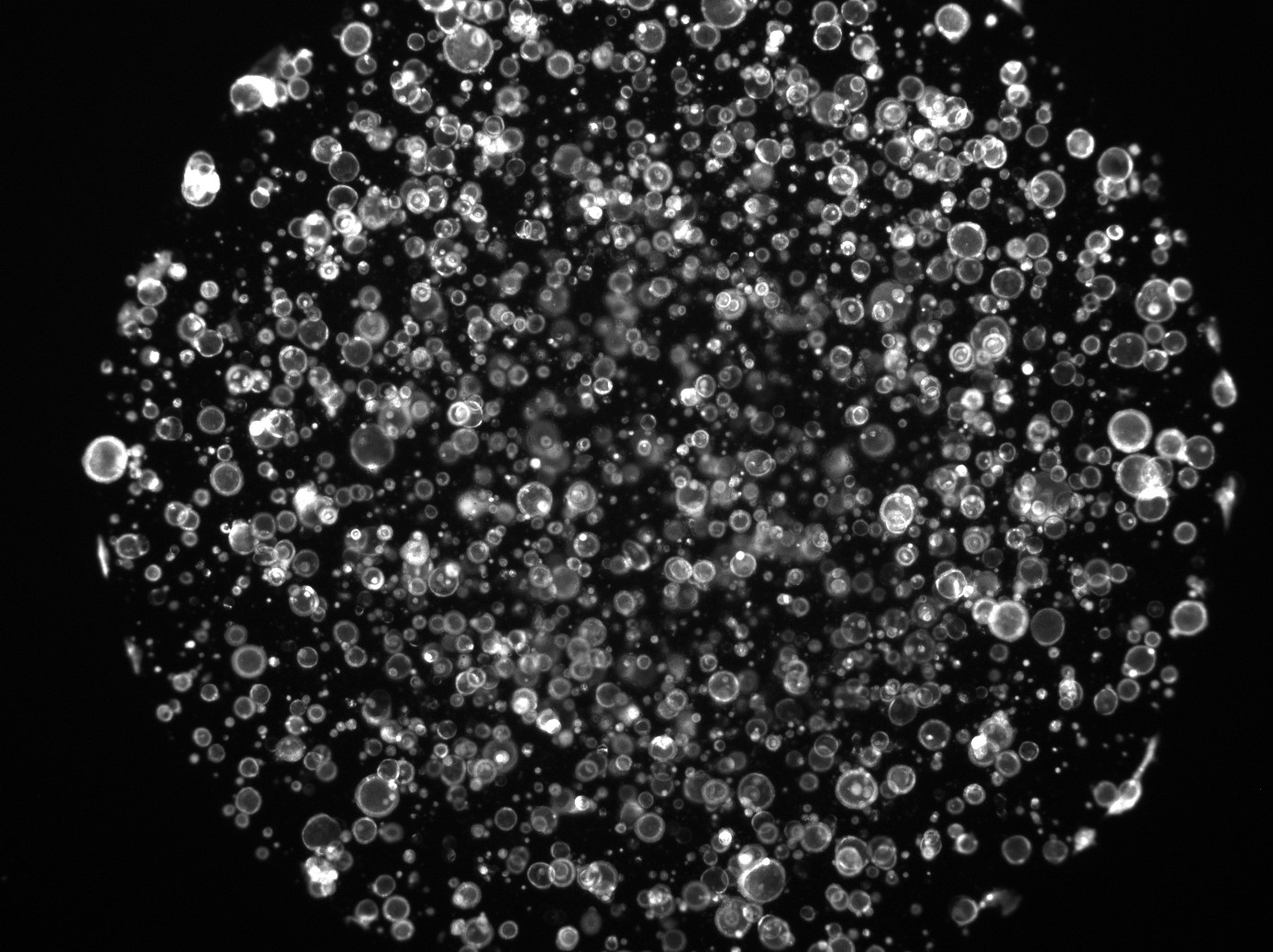

Supplement: Supplementary file 4 — Source Data [file 41467_2024_45605_MOESM4_ESM.zip › Source Data/Figures_Source_Data/figure 3/panel b/B5_TdTomato.jpg]

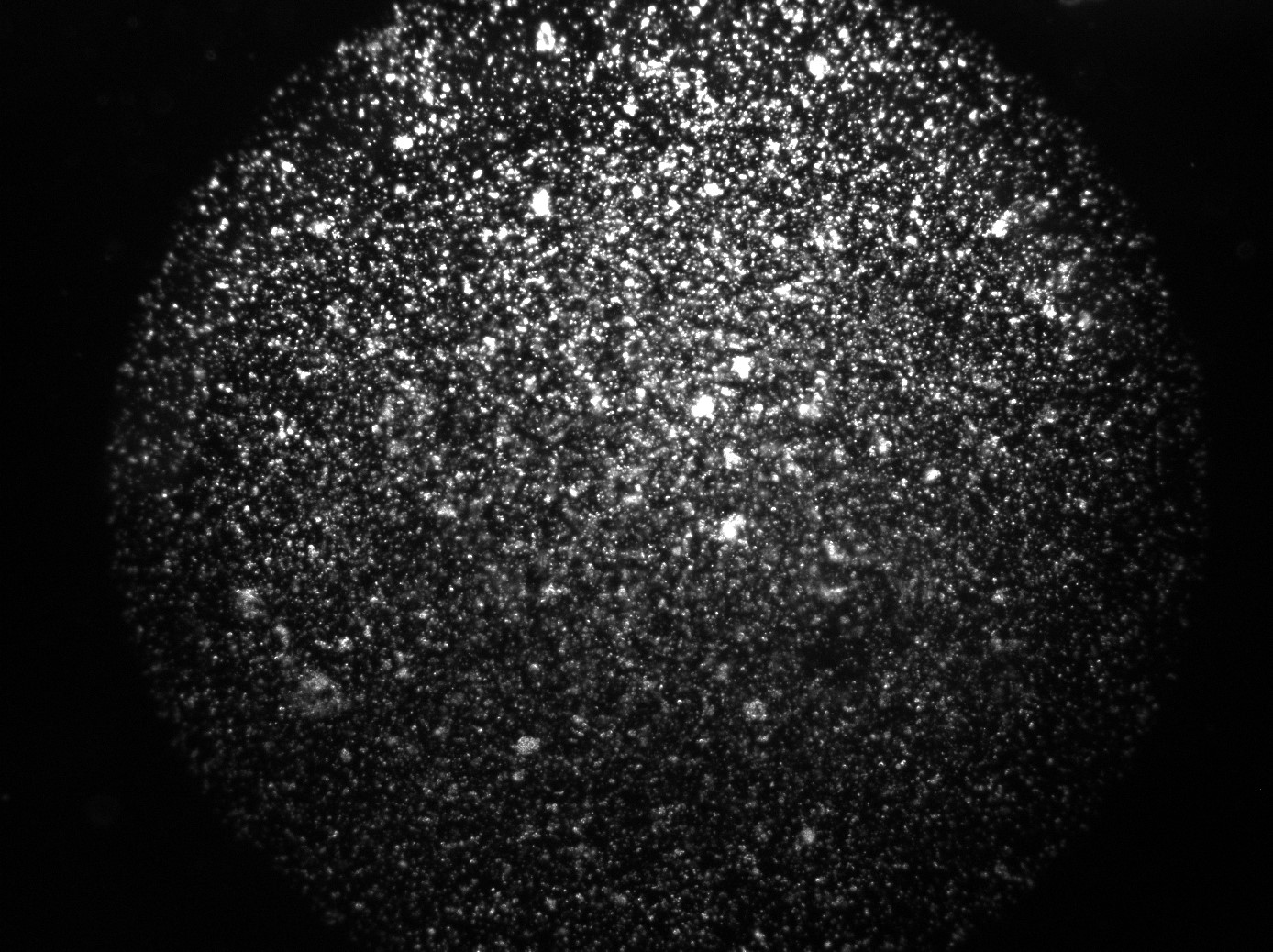

Supplement: Supplementary file 4 — Source Data [file 41467_2024_45605_MOESM4_ESM.zip › Source Data/Figures_Source_Data/figure 3/panel b/B12.jpg]

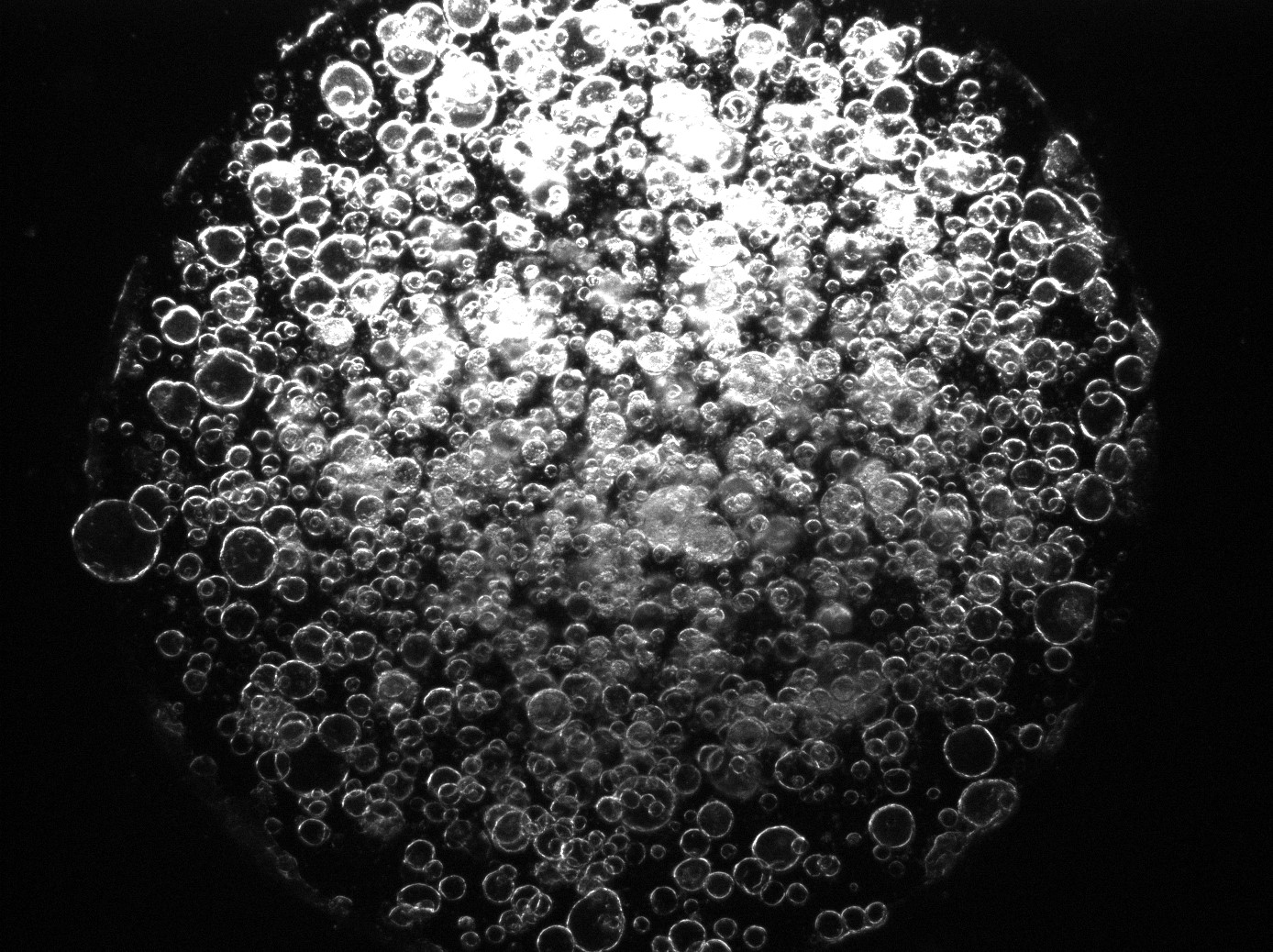

Supplement: Supplementary file 4 — Source Data [file 41467_2024_45605_MOESM4_ESM.zip › Source Data/Figures_Source_Data/figure 3/panel b/B13.jpg]

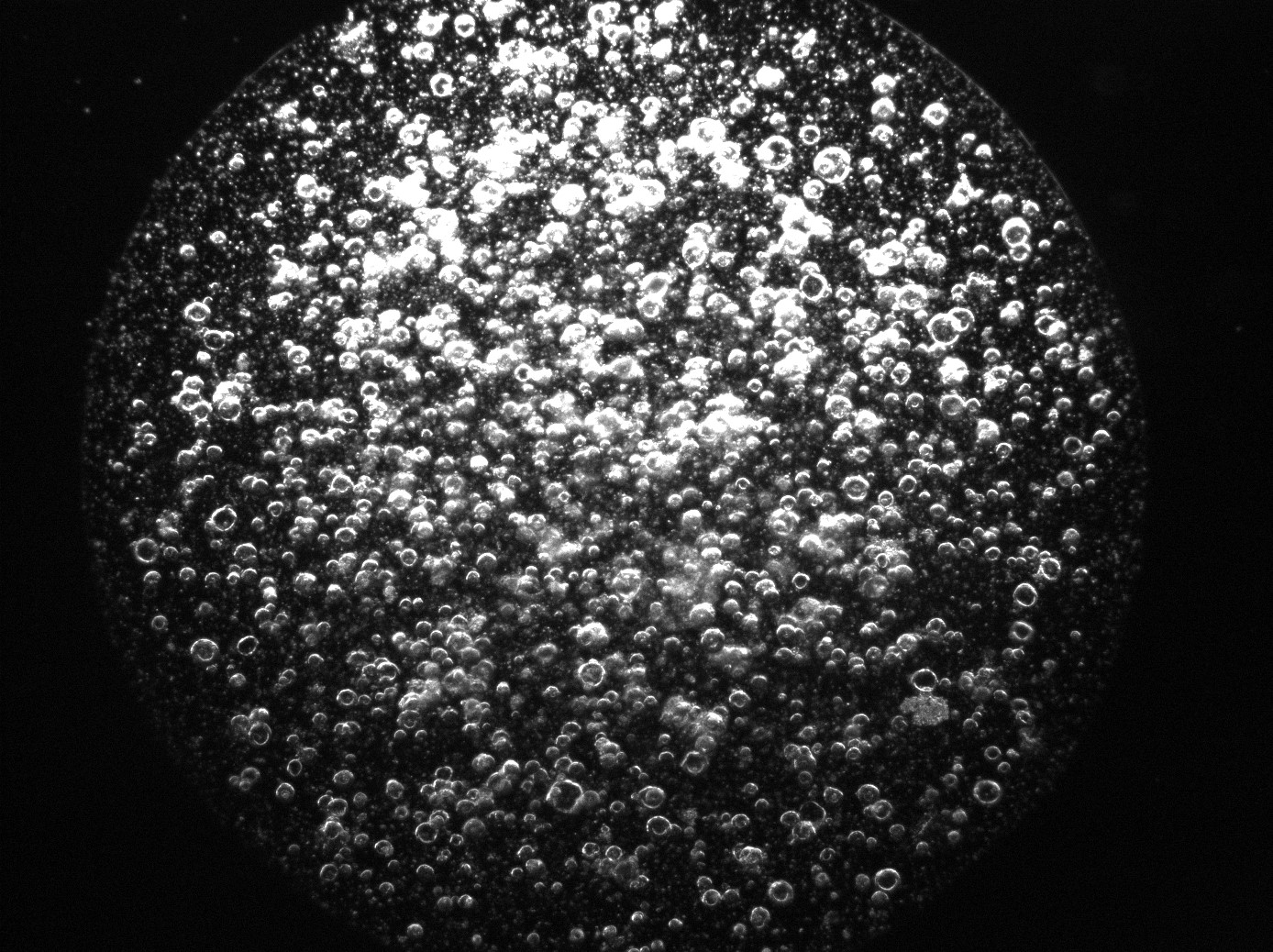

Supplement: Supplementary file 4 — Source Data [file 41467_2024_45605_MOESM4_ESM.zip › Source Data/Figures_Source_Data/figure 3/panel b/B17.jpg]

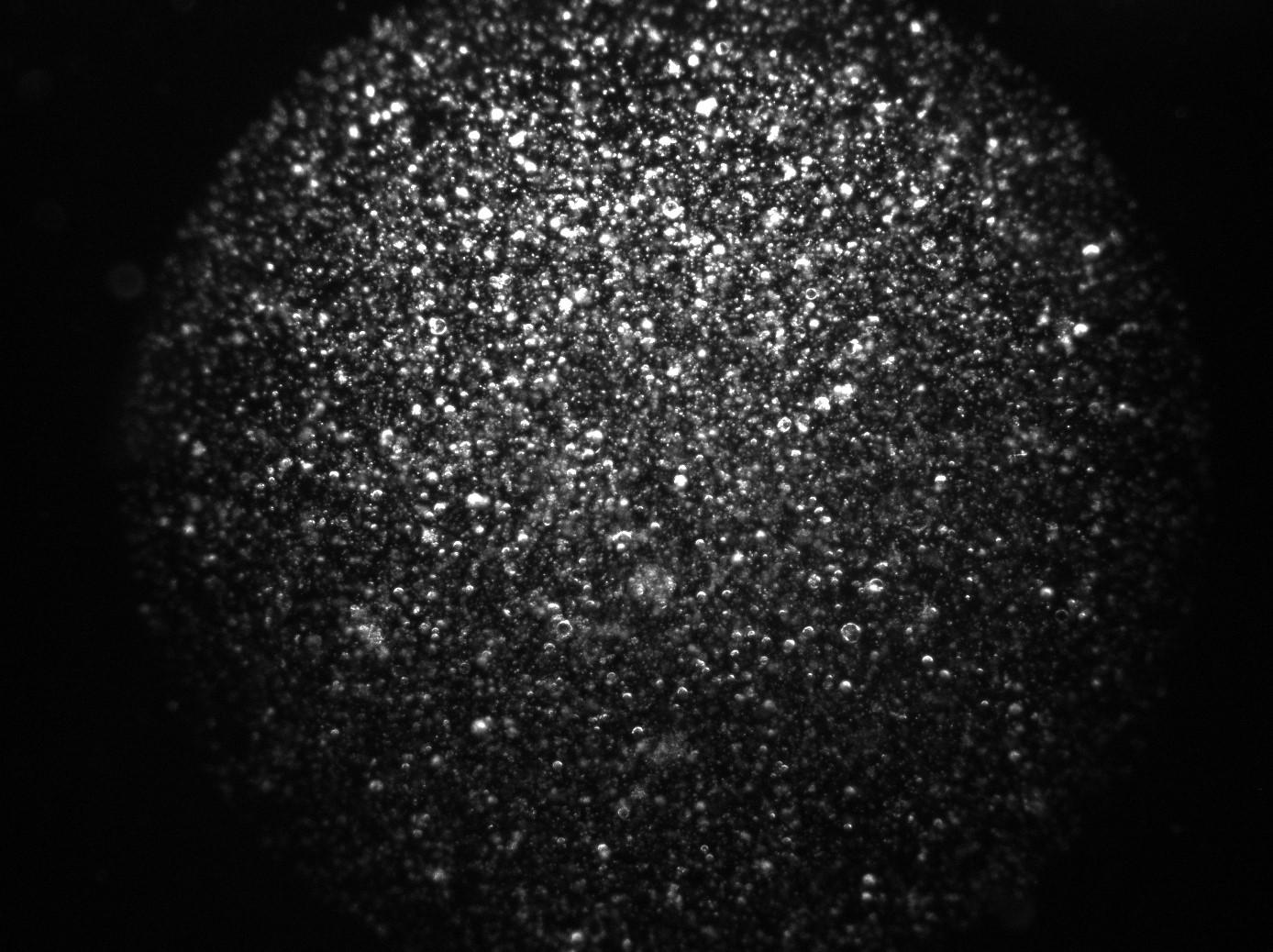

Supplement: Supplementary file 4 — Source Data [file 41467_2024_45605_MOESM4_ESM.zip › Source Data/Figures_Source_Data/figure 3/panel b/B16.jpg]

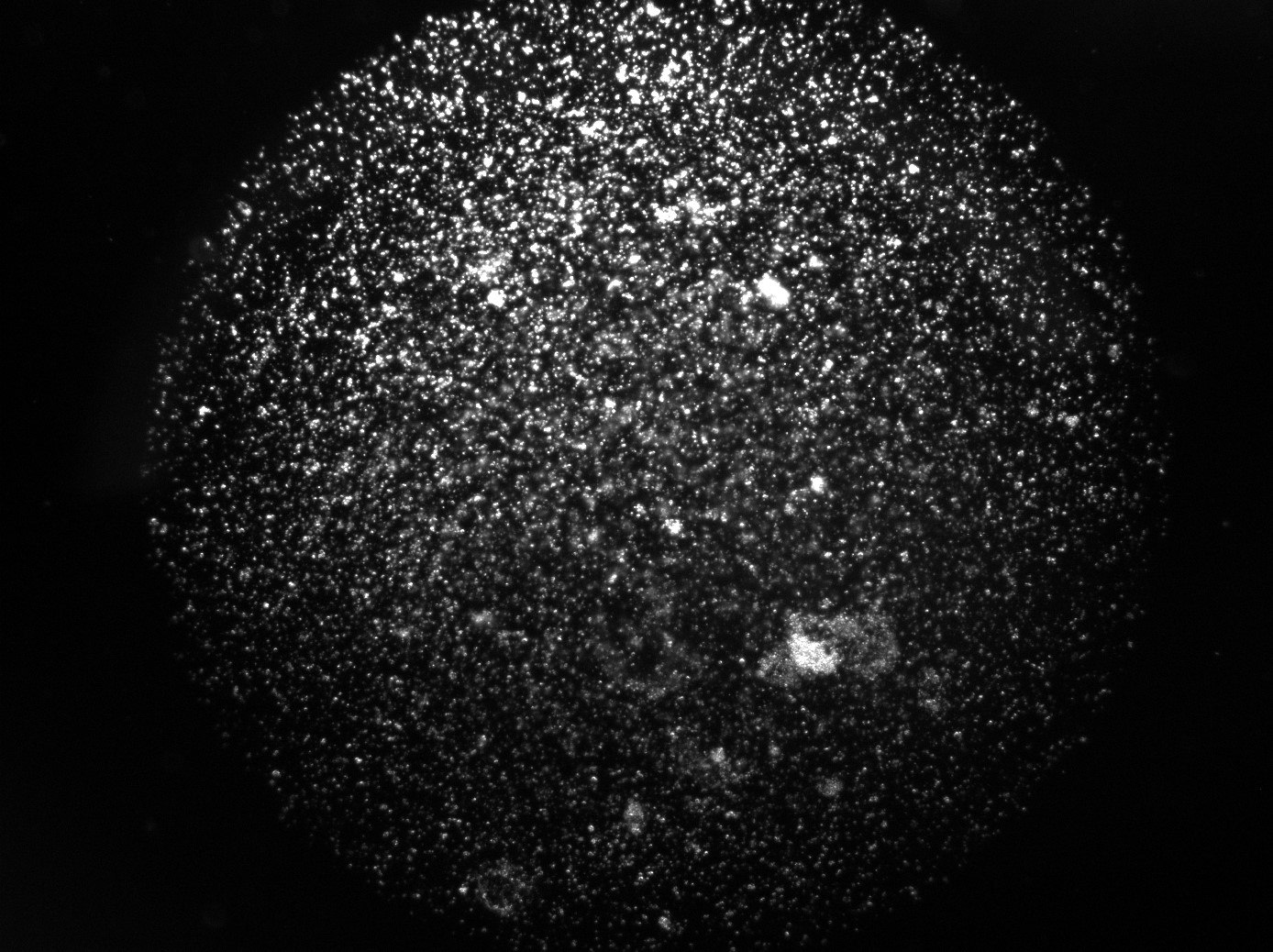

Supplement: Supplementary file 4 — Source Data [file 41467_2024_45605_MOESM4_ESM.zip › Source Data/Figures_Source_Data/figure 3/panel b/B28.jpg]

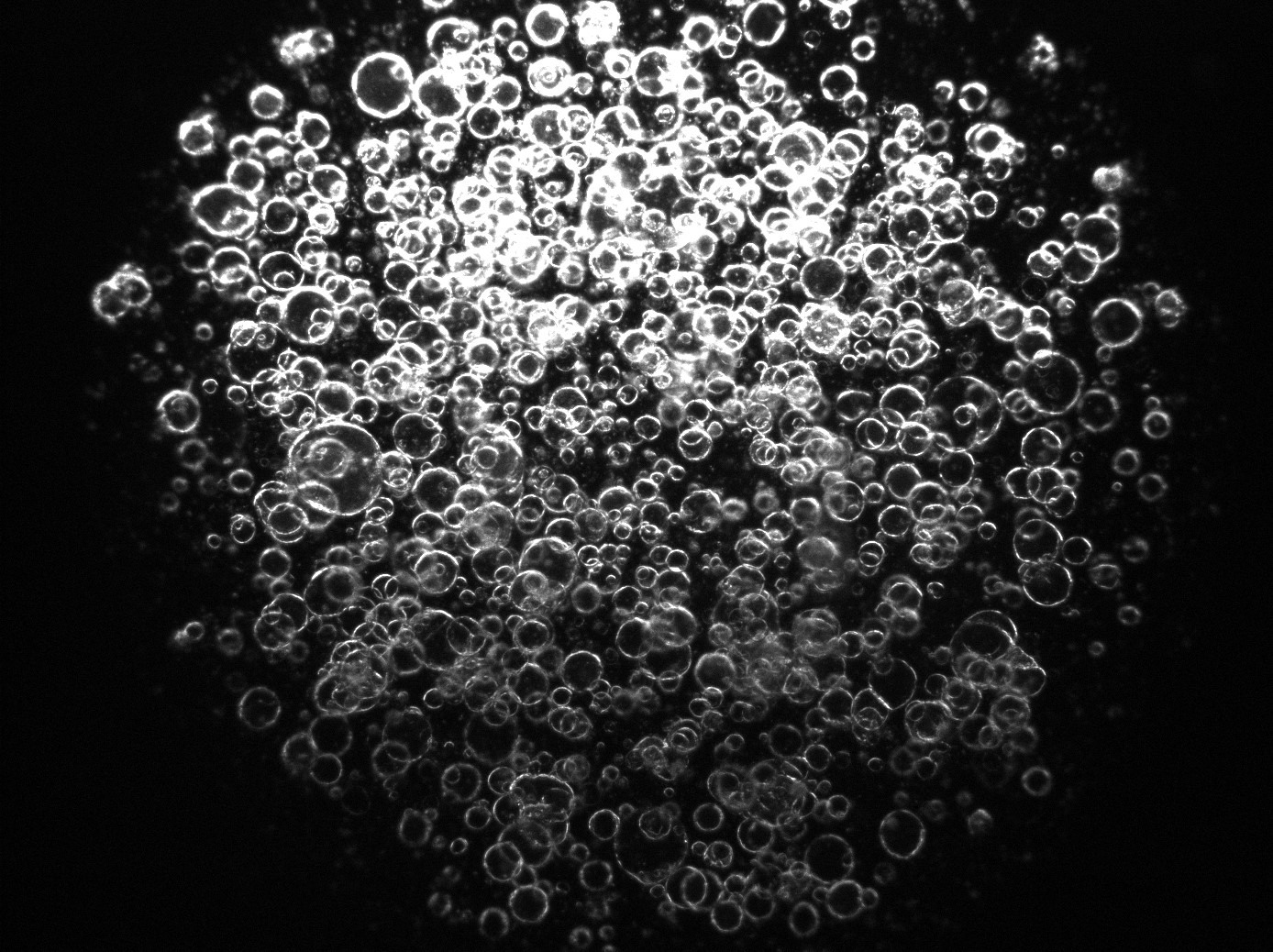

Supplement: Supplementary file 4 — Source Data [file 41467_2024_45605_MOESM4_ESM.zip › Source Data/Figures_Source_Data/figure 3/panel b/B14.jpg]

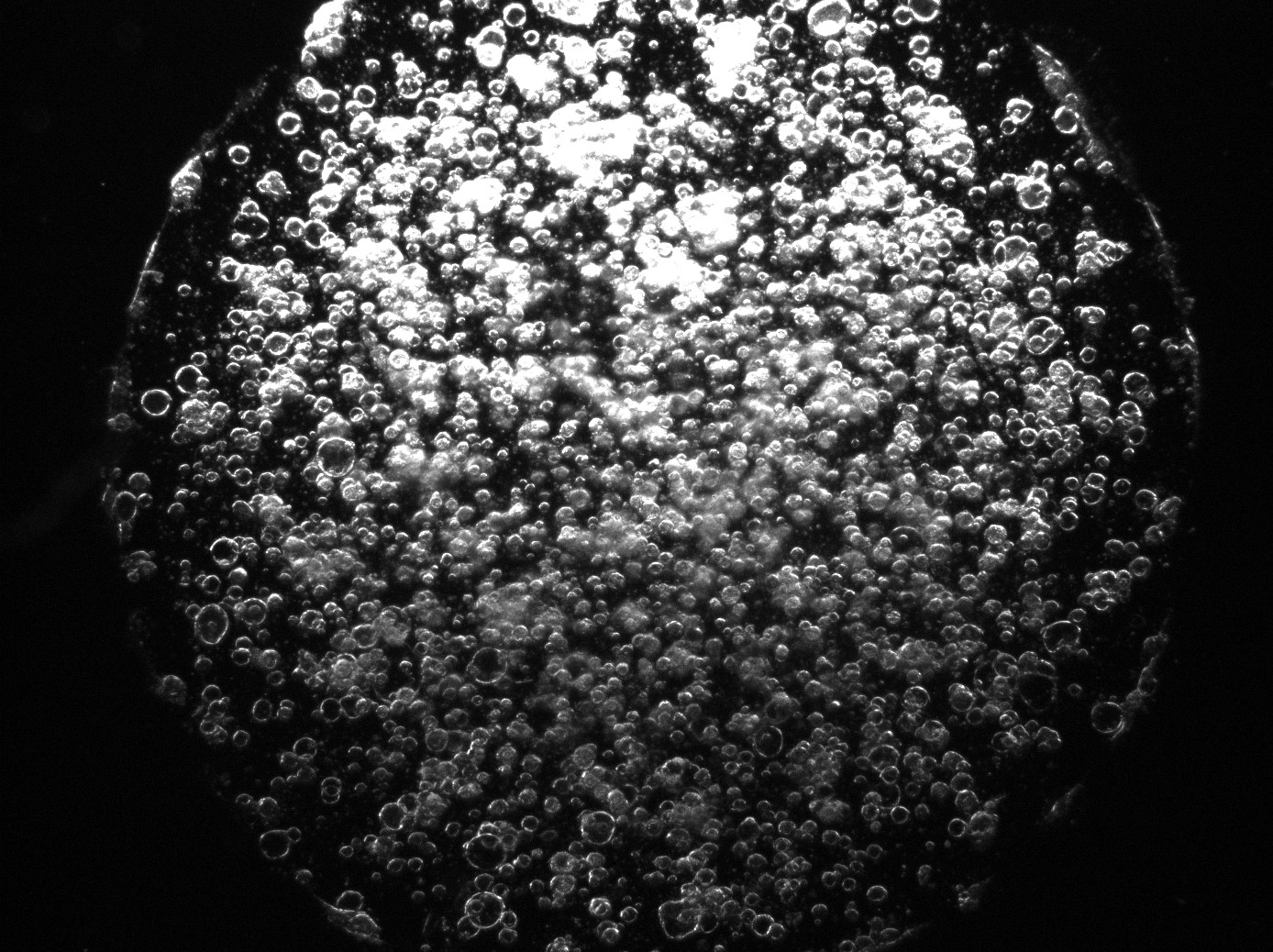

Supplement: Supplementary file 4 — Source Data [file 41467_2024_45605_MOESM4_ESM.zip › Source Data/Figures_Source_Data/figure 3/panel b/B15.jpg]

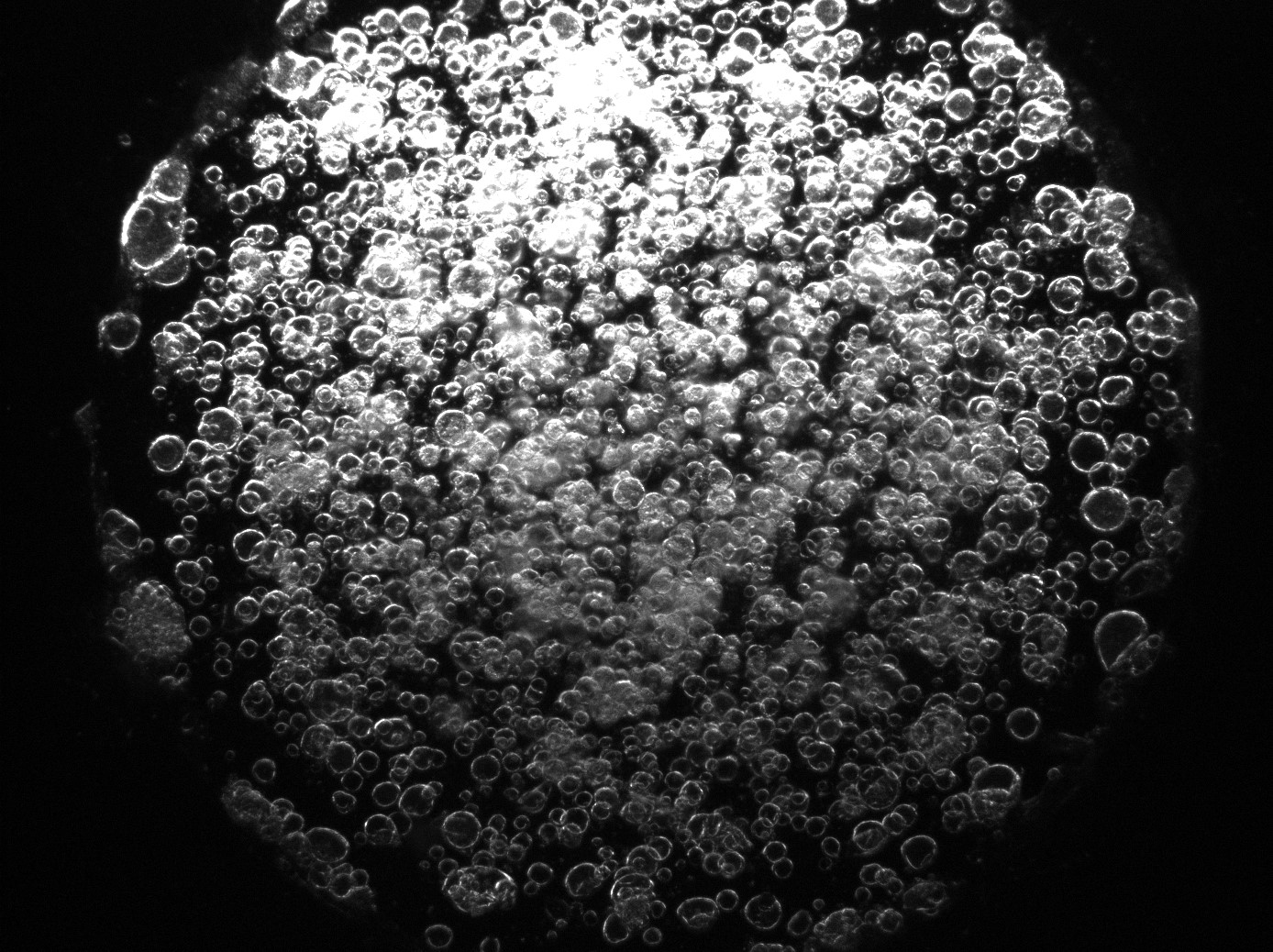

Supplement: Supplementary file 4 — Source Data [file 41467_2024_45605_MOESM4_ESM.zip › Source Data/Figures_Source_Data/figure 3/panel b/B29.jpg]

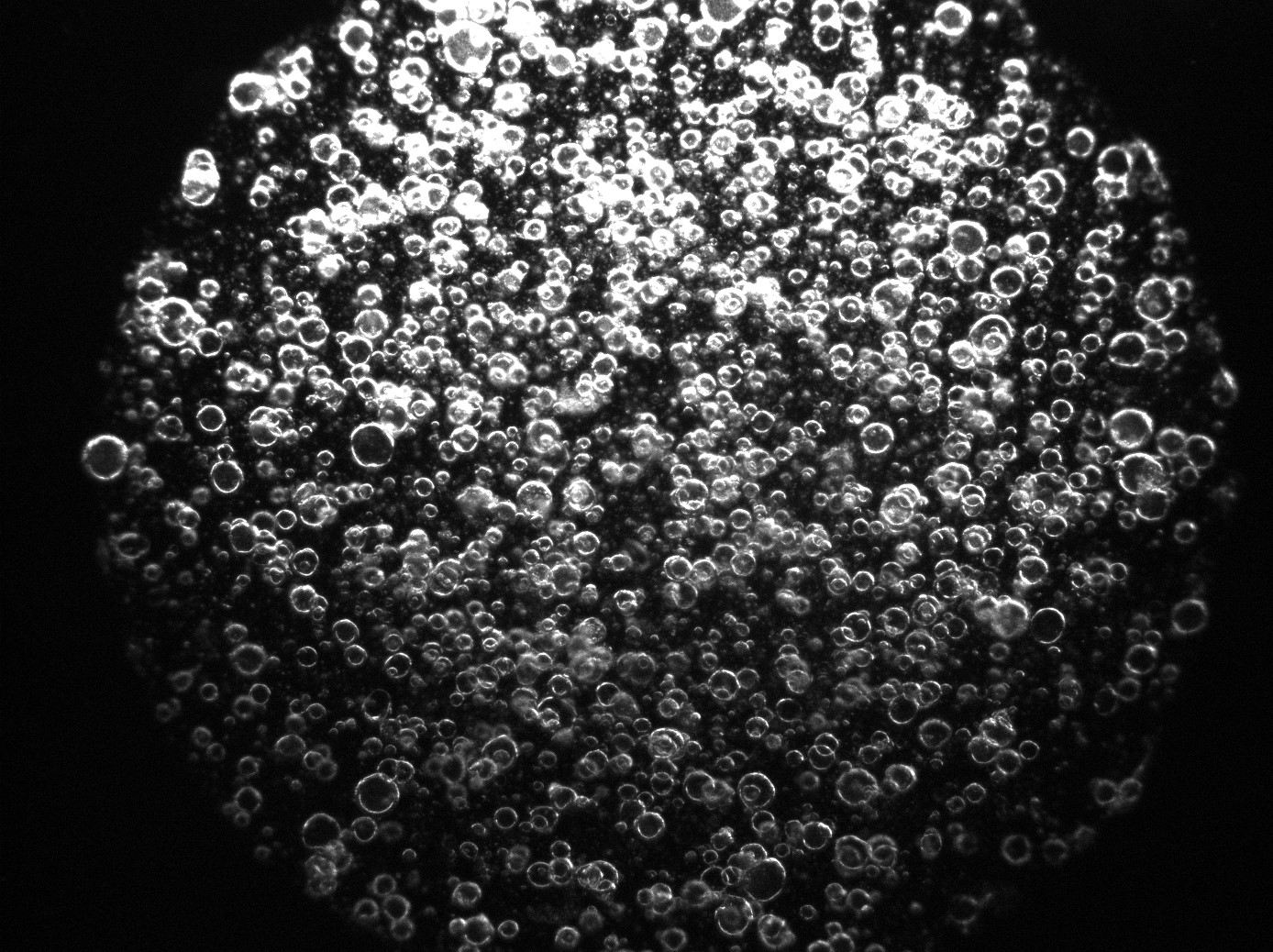

Supplement: Supplementary file 4 — Source Data [file 41467_2024_45605_MOESM4_ESM.zip › Source Data/Figures_Source_Data/figure 3/panel b/B5.jpg]

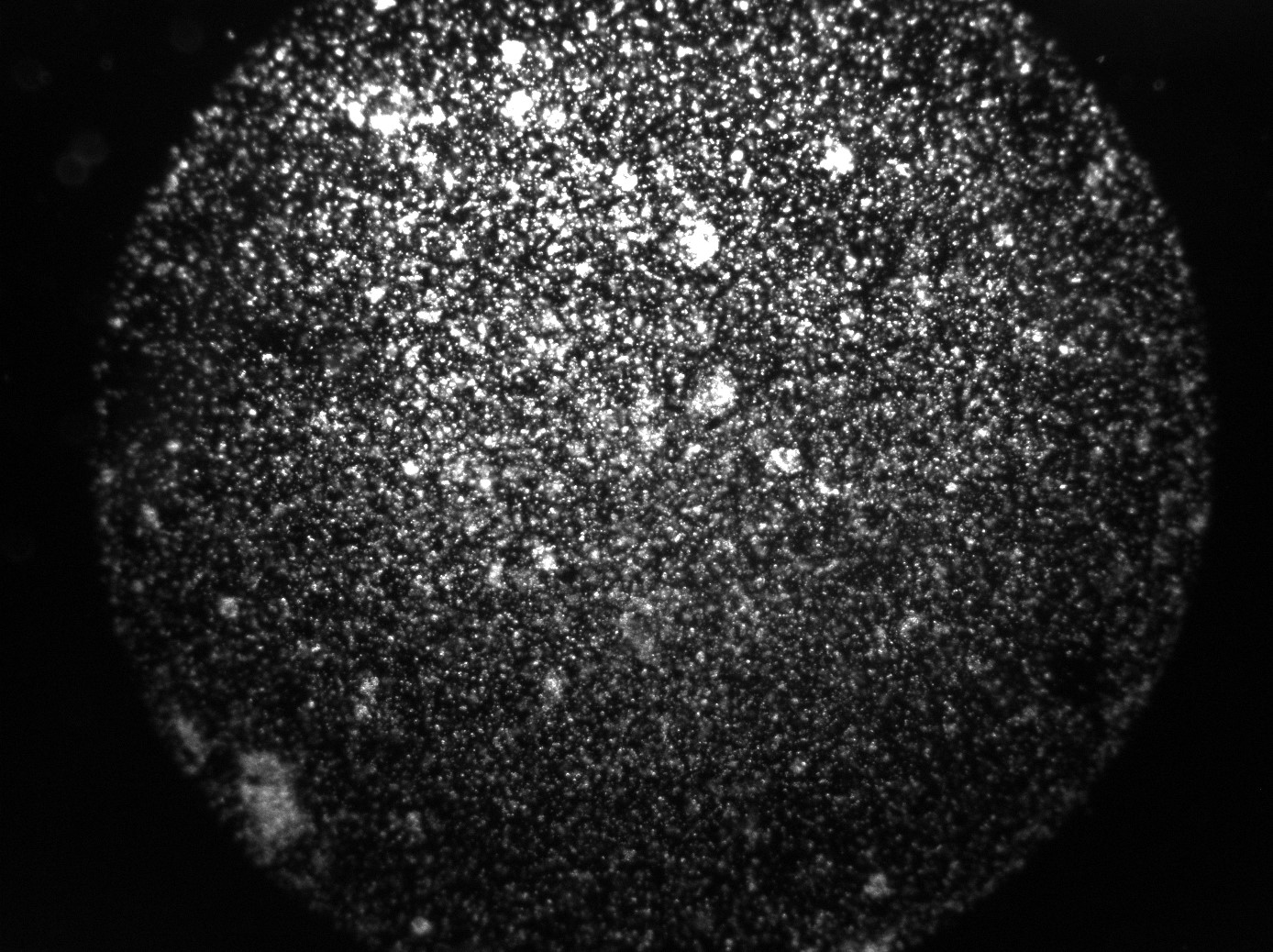

Supplement: Supplementary file 4 — Source Data [file 41467_2024_45605_MOESM4_ESM.zip › Source Data/Figures_Source_Data/figure 3/panel b/B4.jpg]

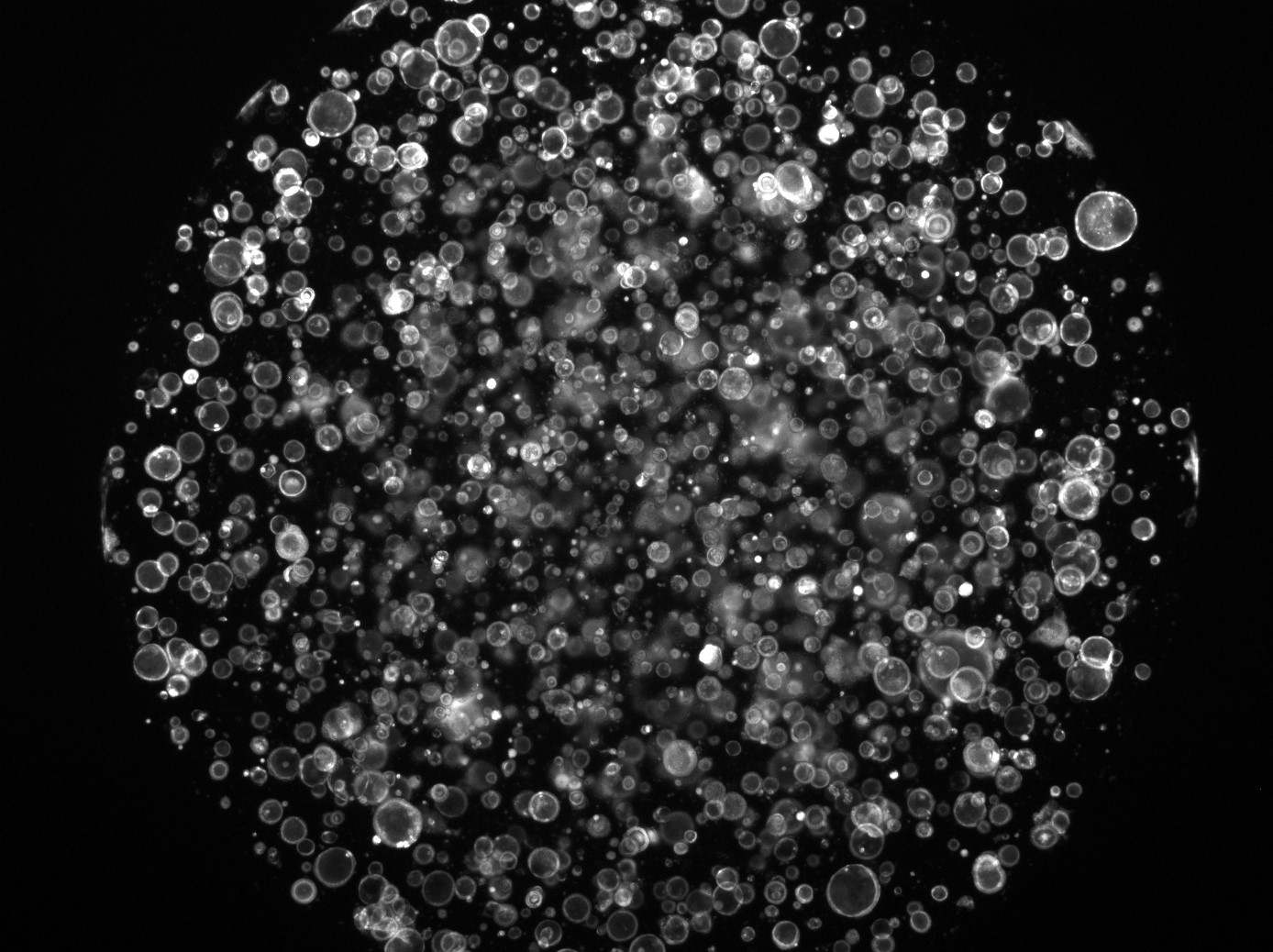

Supplement: Supplementary file 4 — Source Data [file 41467_2024_45605_MOESM4_ESM.zip › Source Data/Figures_Source_Data/figure 3/panel b/B21_TdTomato.jpg]

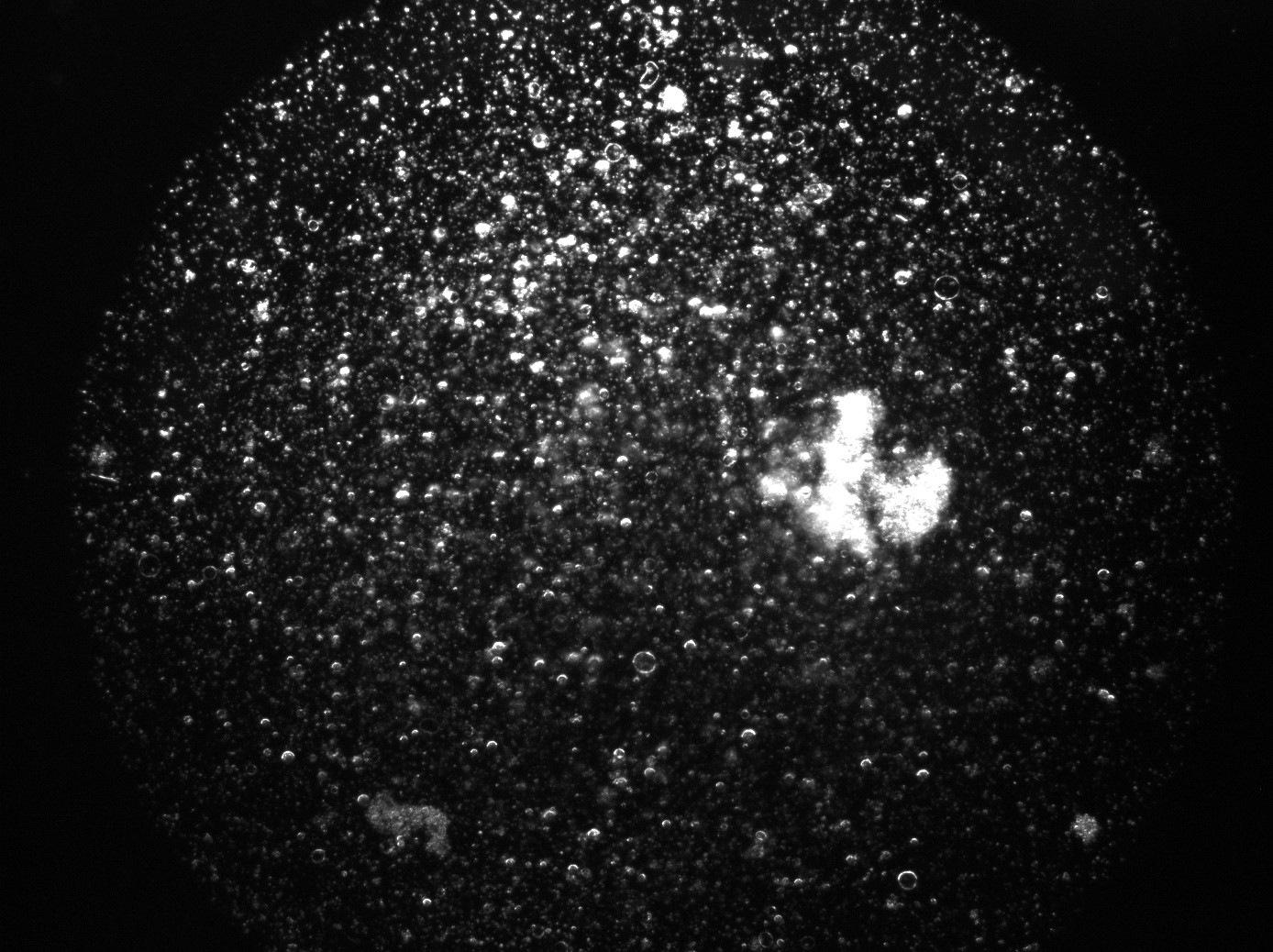

Supplement: Supplementary file 4 — Source Data [file 41467_2024_45605_MOESM4_ESM.zip › Source Data/Figures_Source_Data/figure 3/panel b/B6.jpg]

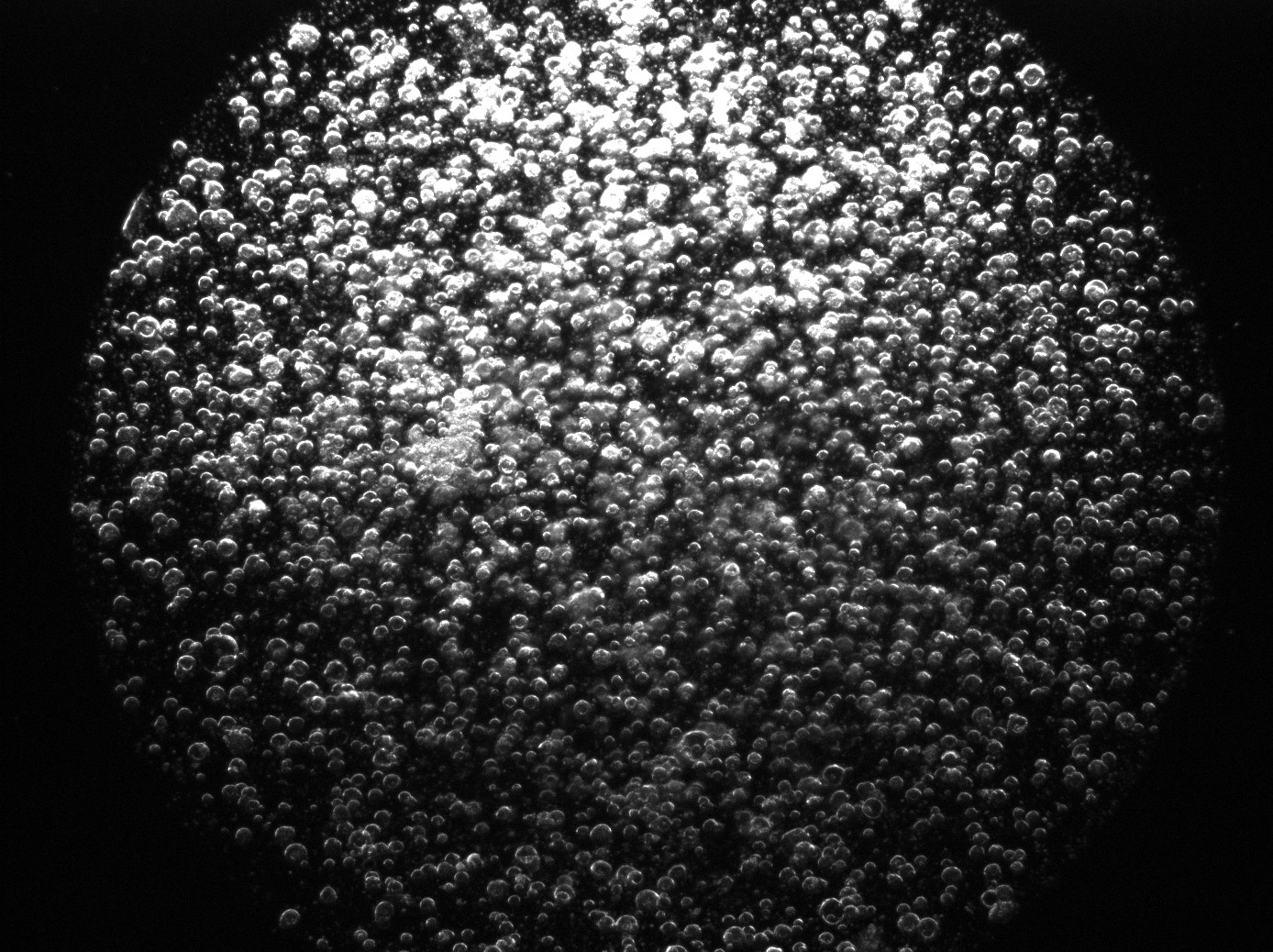

Supplement: Supplementary file 4 — Source Data [file 41467_2024_45605_MOESM4_ESM.zip › Source Data/Figures_Source_Data/figure 3/panel b/B7.jpg]

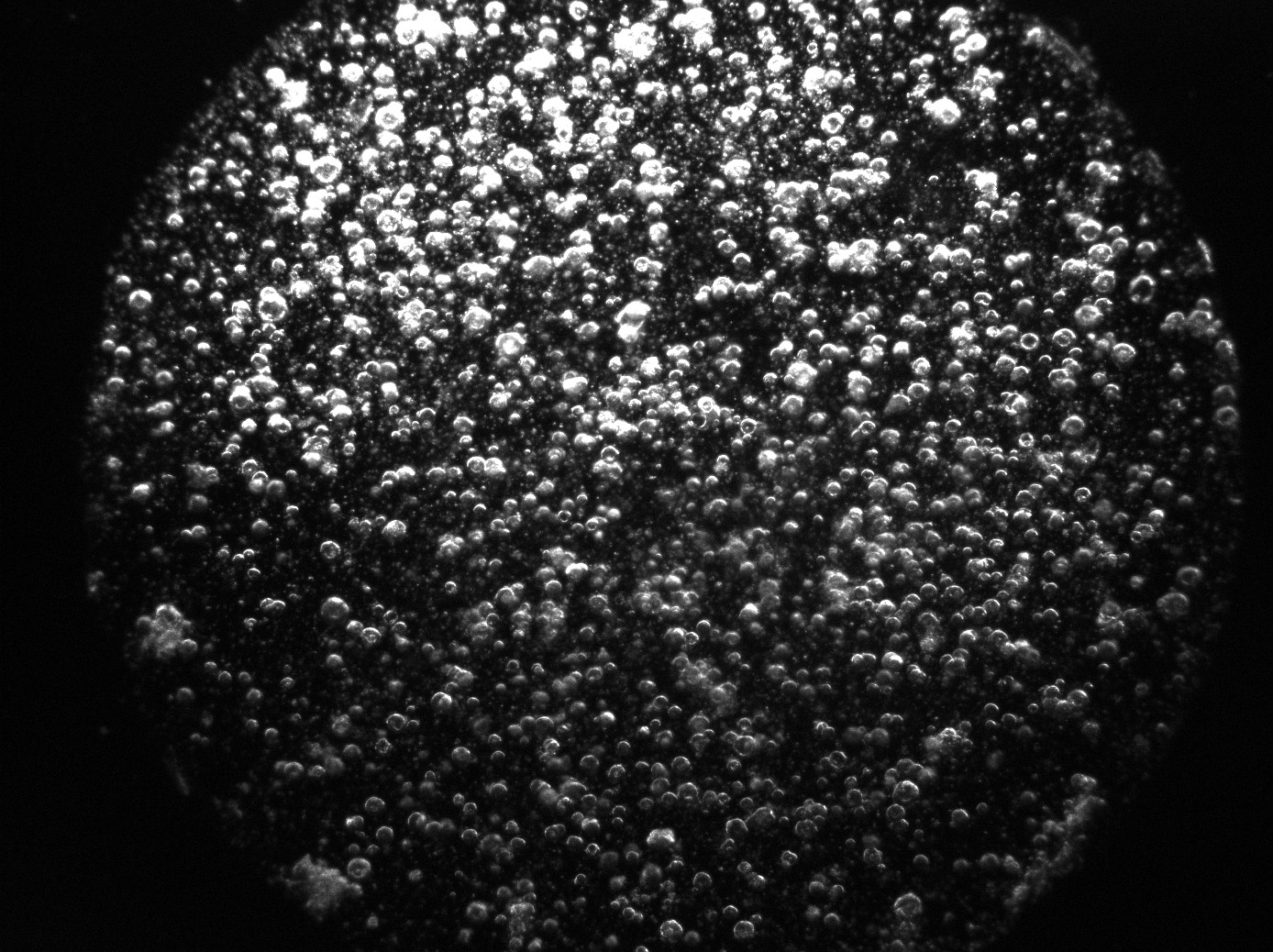

Supplement: Supplementary file 4 — Source Data [file 41467_2024_45605_MOESM4_ESM.zip › Source Data/Figures_Source_Data/figure 3/panel b/B3.jpg]

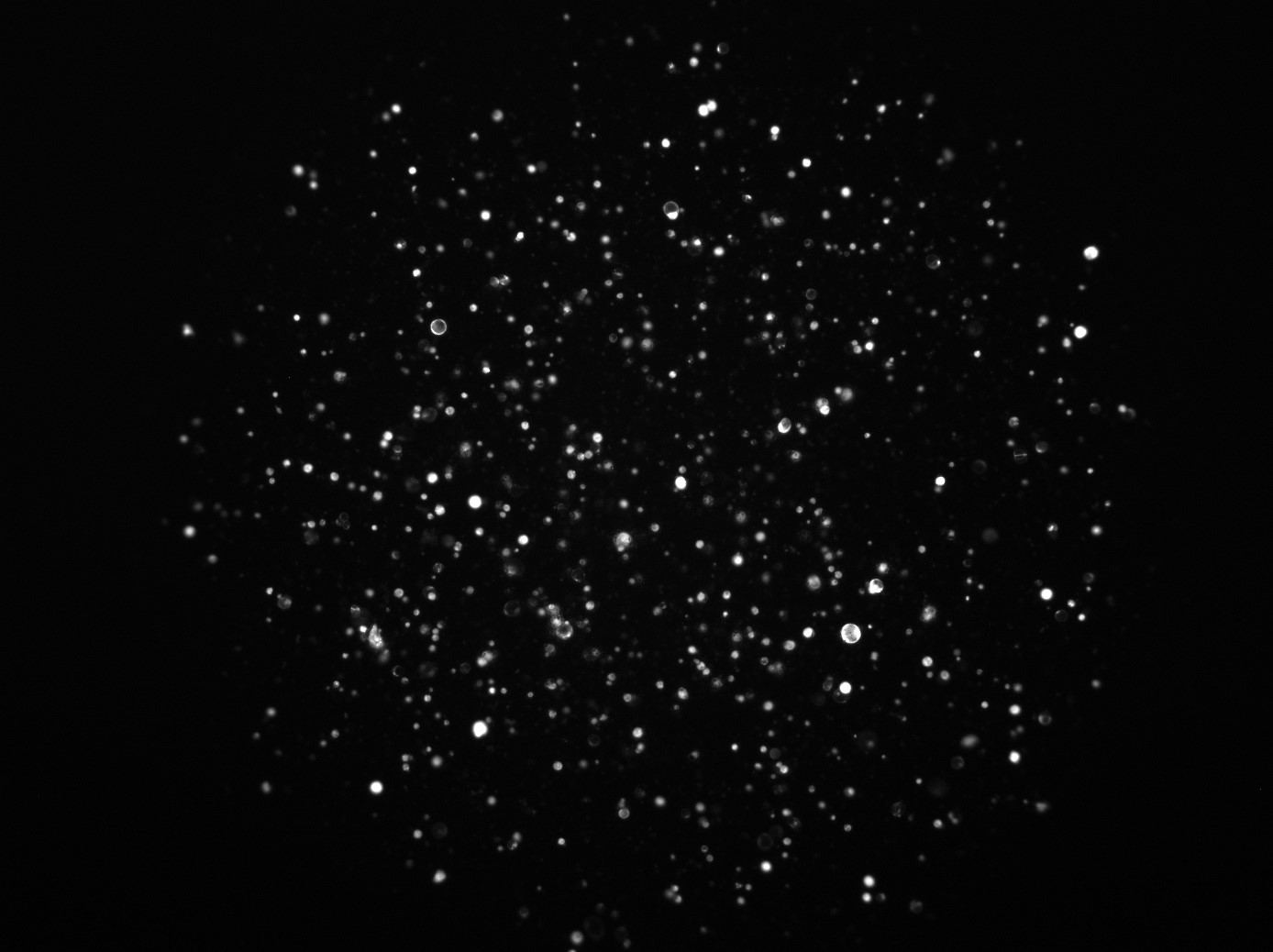

Supplement: Supplementary file 4 — Source Data [file 41467_2024_45605_MOESM4_ESM.zip › Source Data/Figures_Source_Data/figure 3/panel b/B16_TdTomato.jpg]

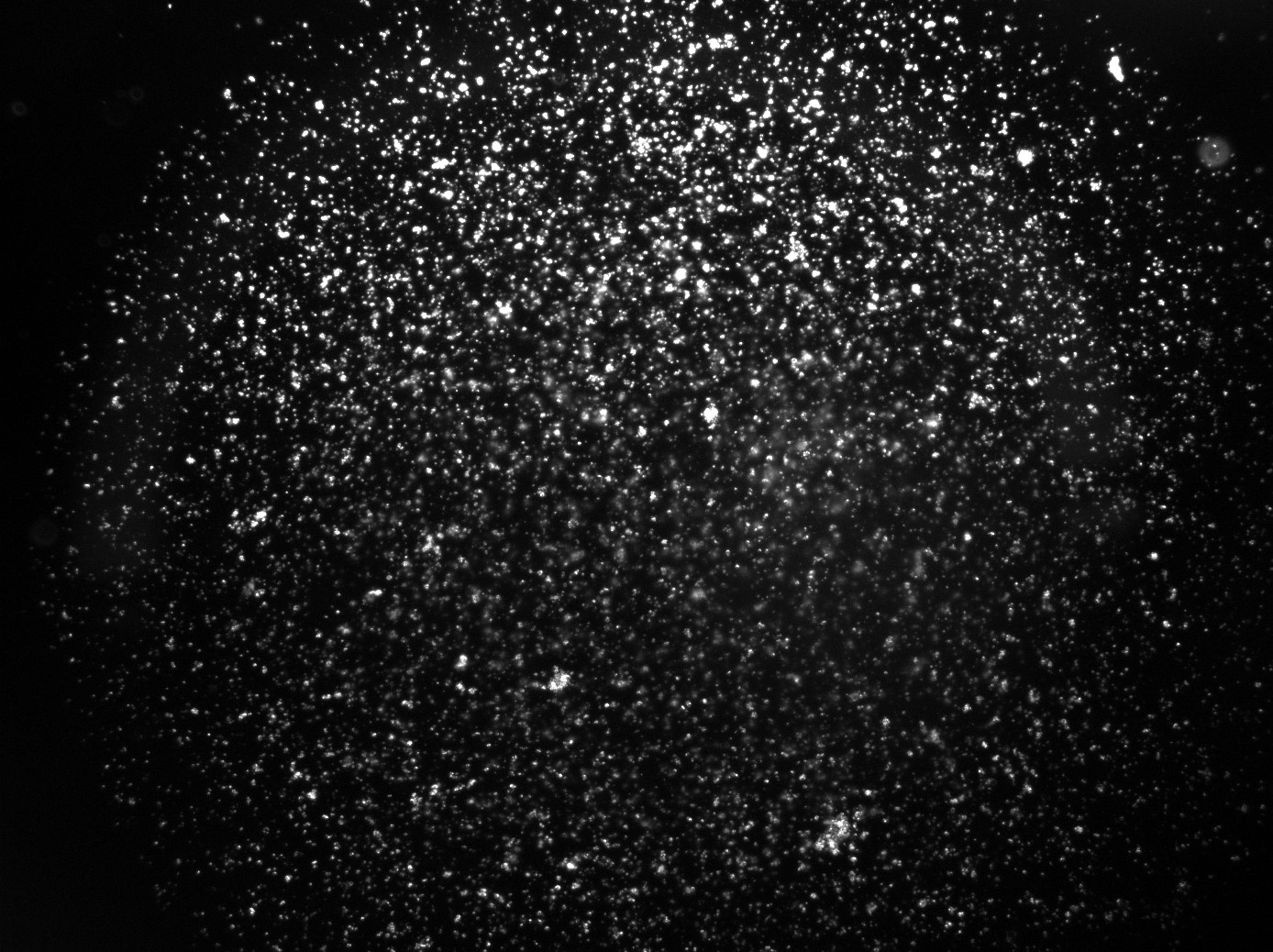

Supplement: Supplementary file 4 — Source Data [file 41467_2024_45605_MOESM4_ESM.zip › Source Data/Figures_Source_Data/figure 3/panel b/B2.jpg]

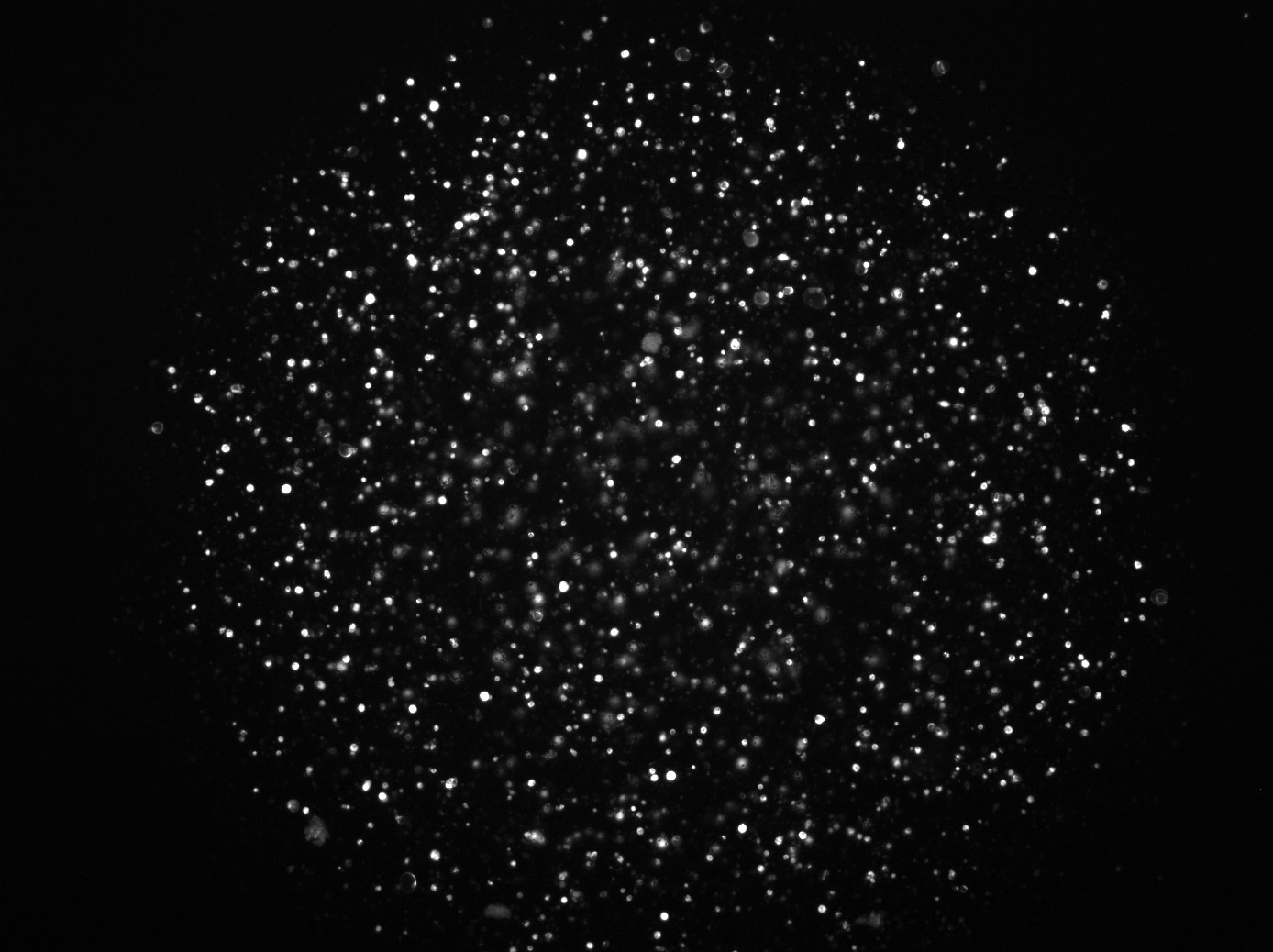

Supplement: Supplementary file 4 — Source Data [file 41467_2024_45605_MOESM4_ESM.zip › Source Data/Figures_Source_Data/figure 3/panel b/B32_TdTomato.jpg]

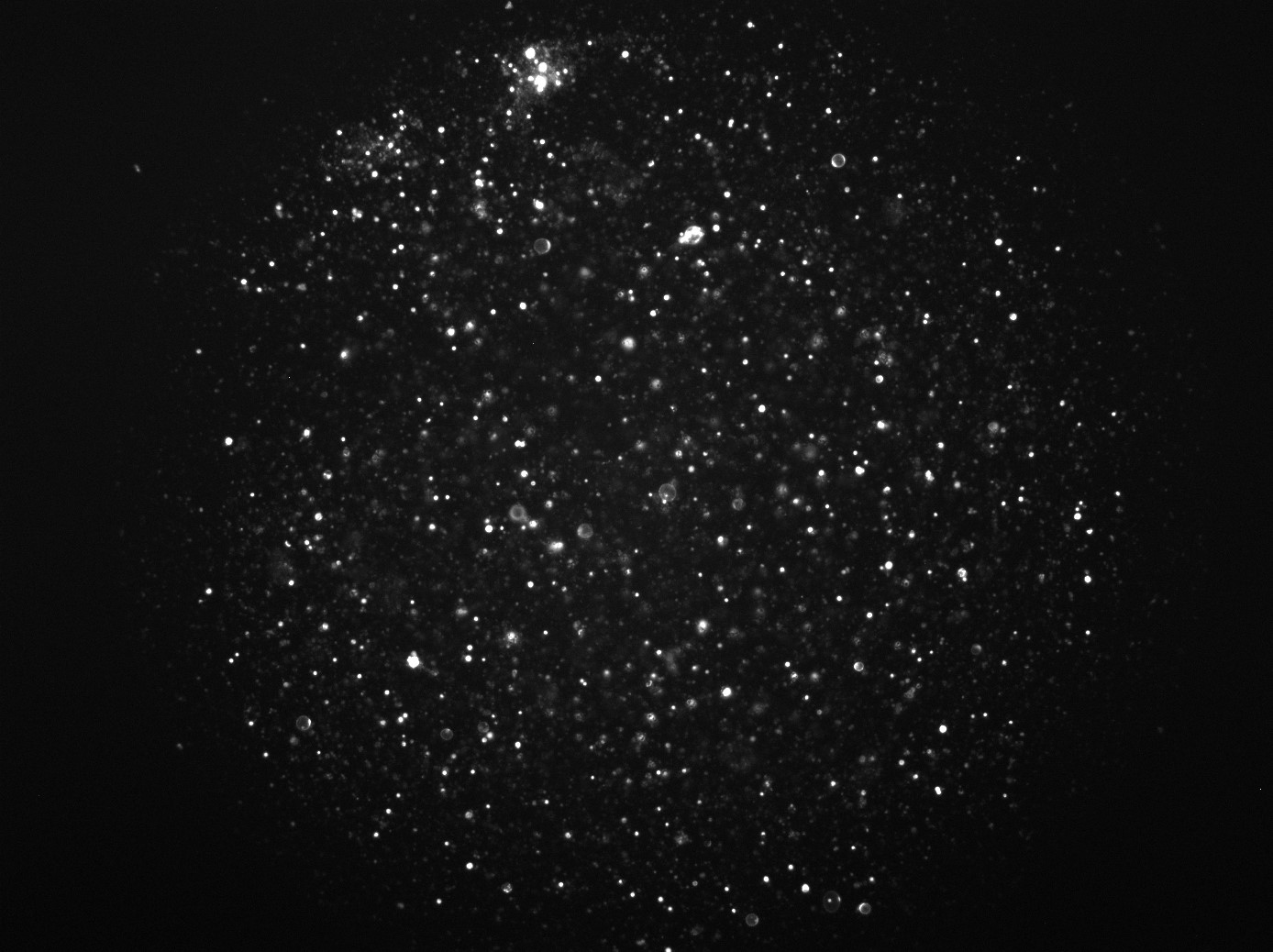

Supplement: Supplementary file 4 — Source Data [file 41467_2024_45605_MOESM4_ESM.zip › Source Data/Figures_Source_Data/figure 3/panel b/B8_TdTomato.jpg]

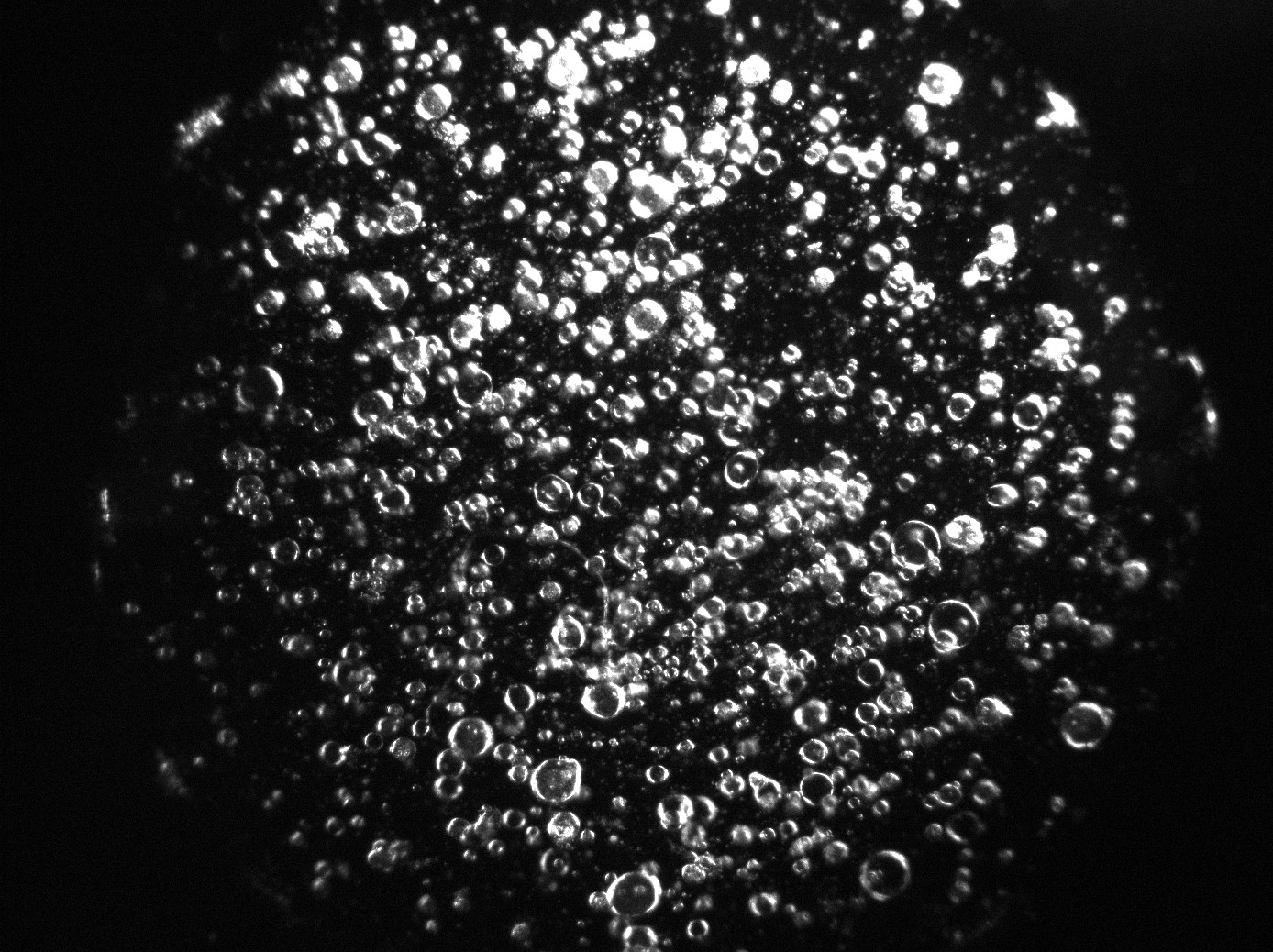

Supplement: Supplementary file 4 — Source Data [file 41467_2024_45605_MOESM4_ESM.zip › Source Data/Figures_Source_Data/figure 3/panel b/B1.jpg]

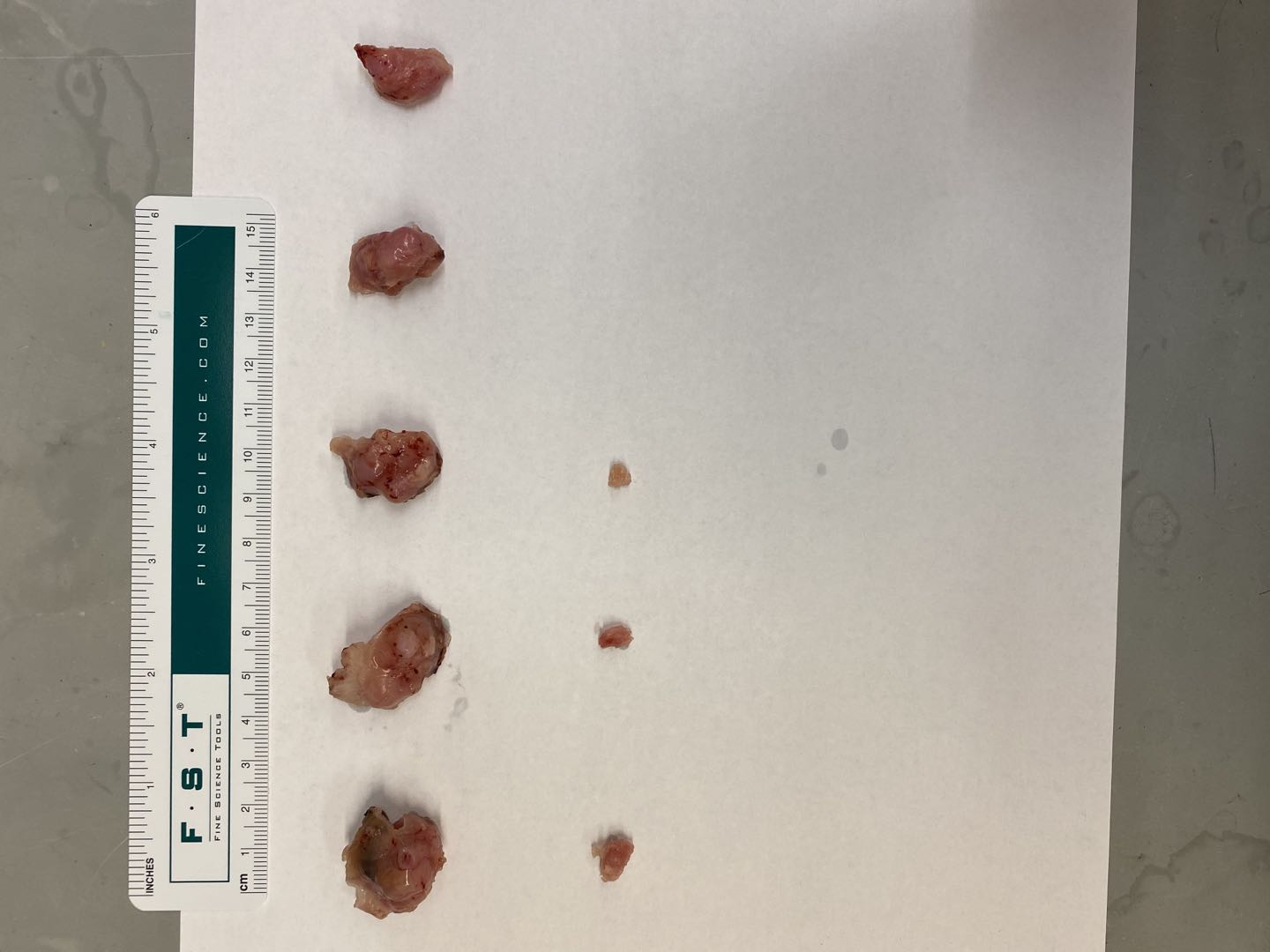

Supplement: Supplementary file 4 — Source Data [file 41467_2024_45605_MOESM4_ESM.zip › Source Data/Figures_Source_Data/figure 3/panel e/E1.jpg]

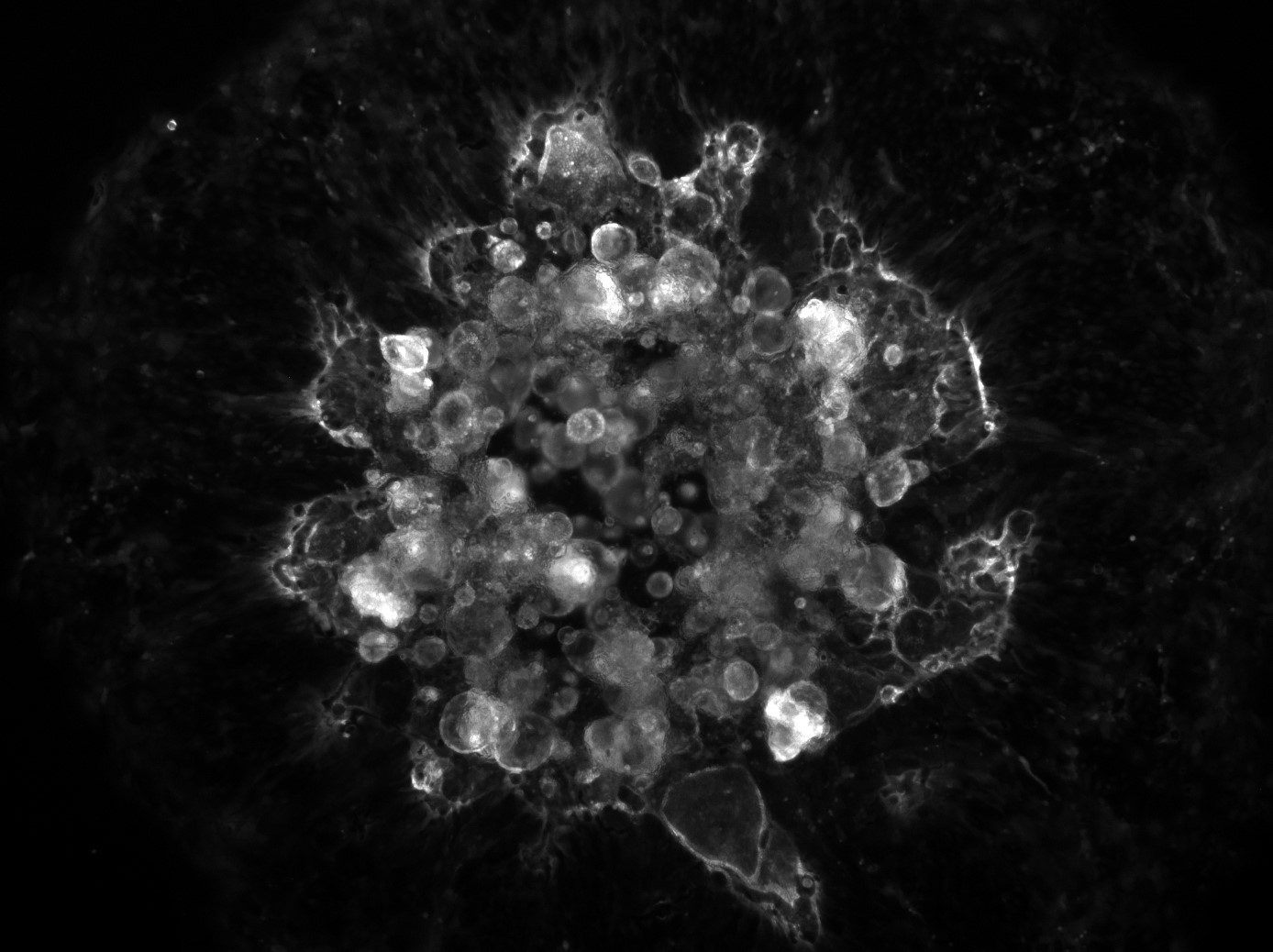

Supplement: Supplementary file 4 — Source Data [file 41467_2024_45605_MOESM4_ESM.zip › Source Data/Figures_Source_Data/figure 3/panel c/C4-tdTomato.jpg]

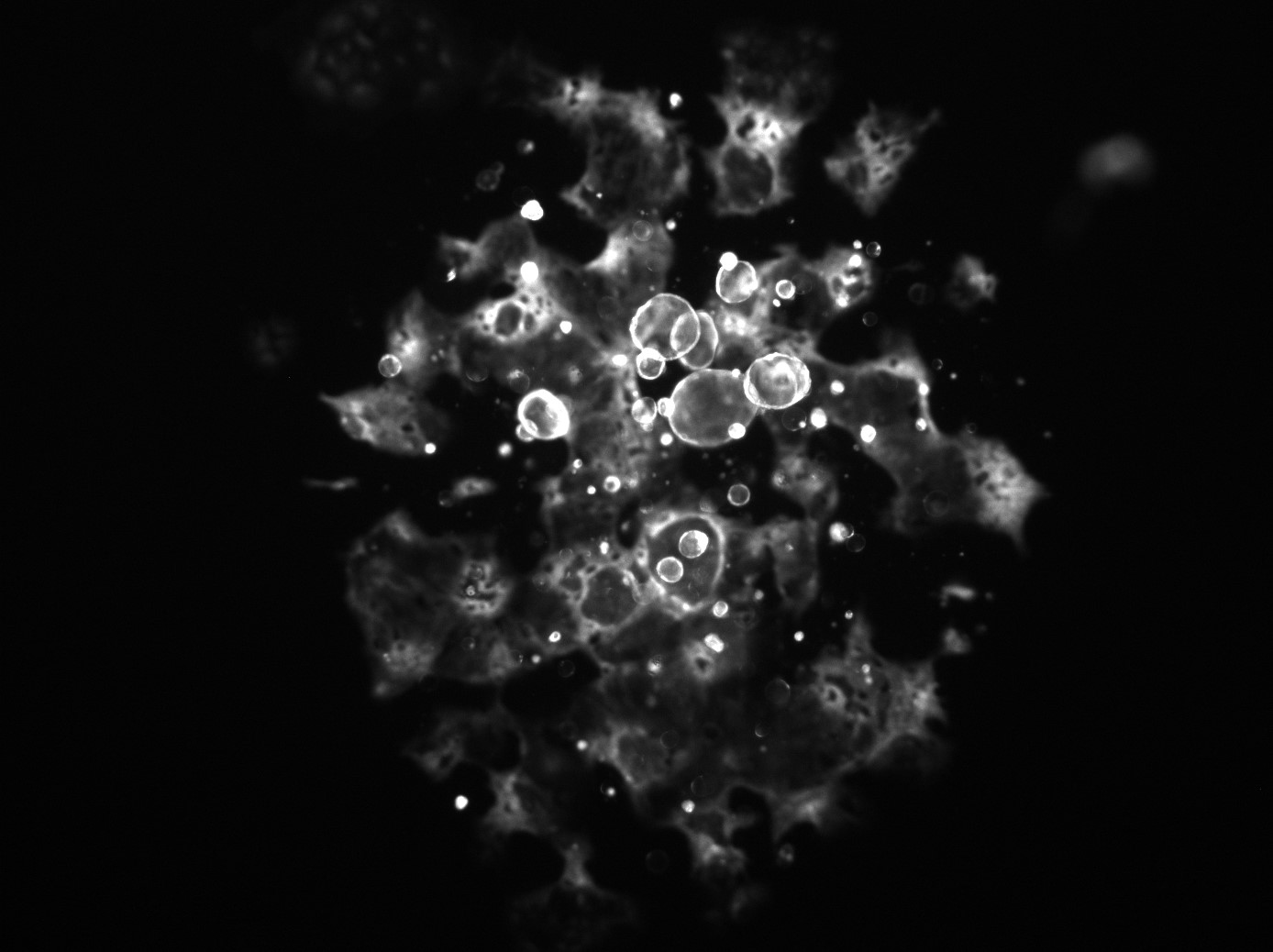

Supplement: Supplementary file 4 — Source Data [file 41467_2024_45605_MOESM4_ESM.zip › Source Data/Figures_Source_Data/figure 3/panel c/C2-tdTomato.jpg]

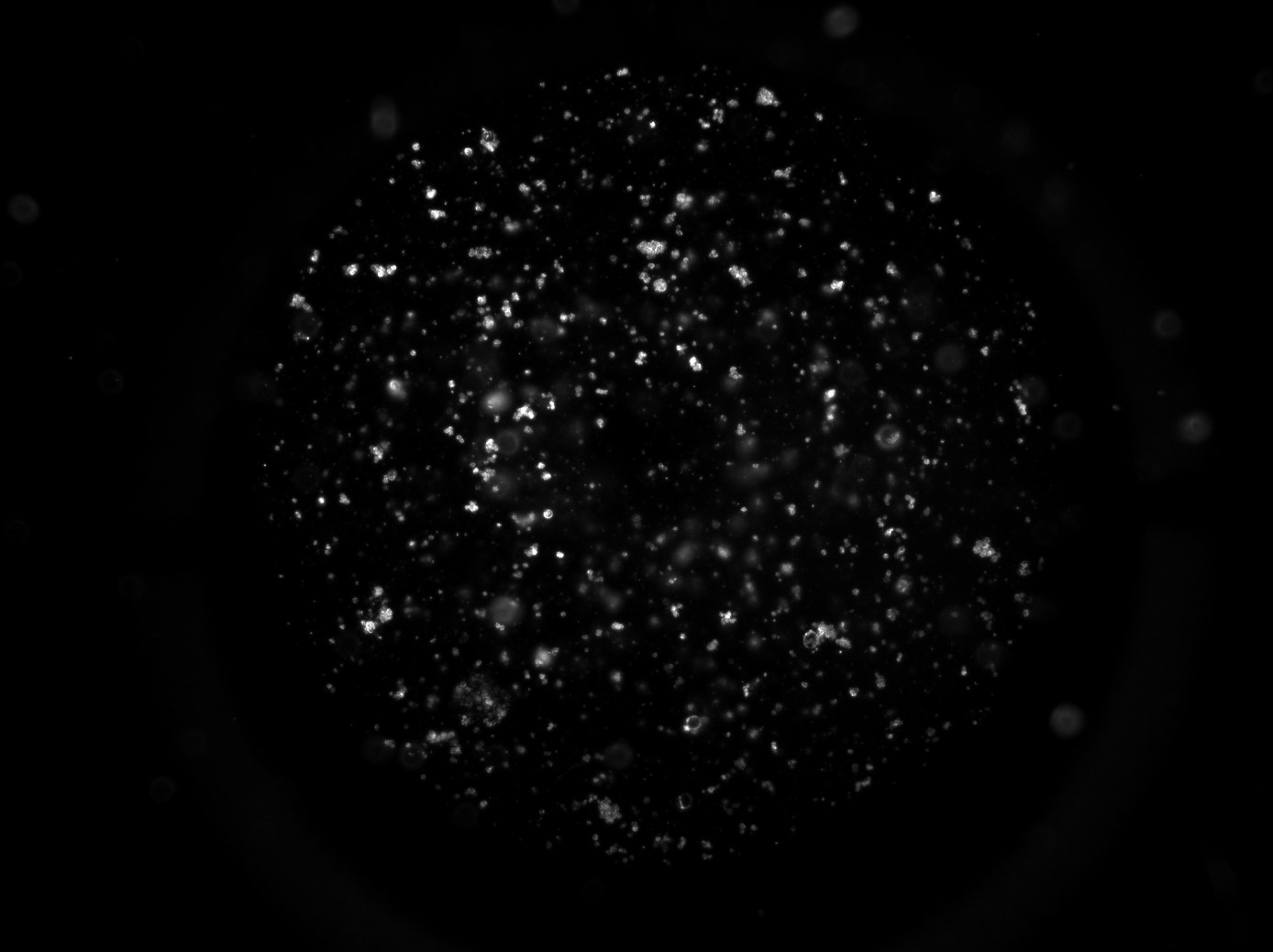

Supplement: Supplementary file 4 — Source Data [file 41467_2024_45605_MOESM4_ESM.zip › Source Data/Figures_Source_Data/figure 3/panel c/C1.jpg]

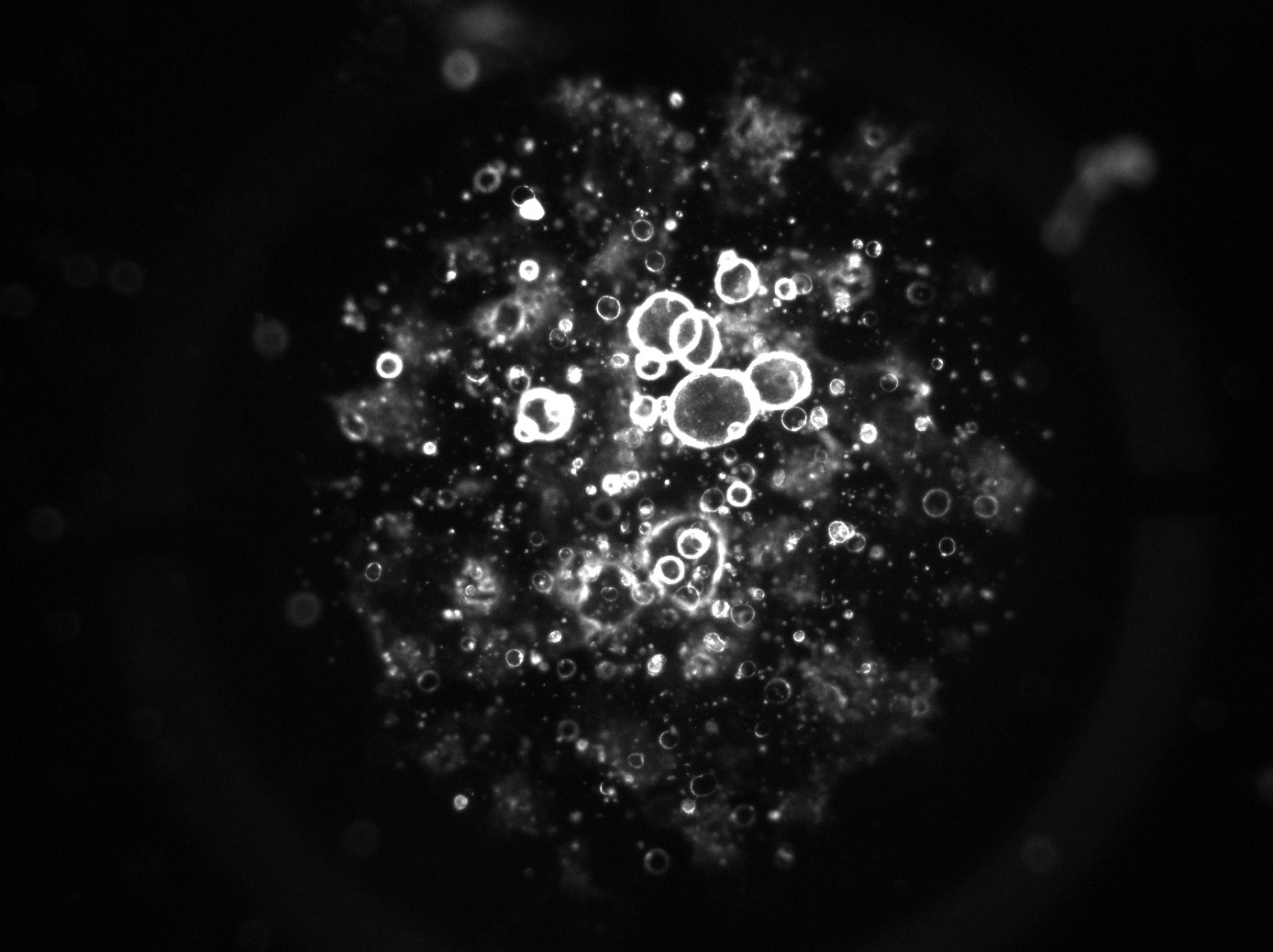

Supplement: Supplementary file 4 — Source Data [file 41467_2024_45605_MOESM4_ESM.zip › Source Data/Figures_Source_Data/figure 3/panel c/C2.jpg]

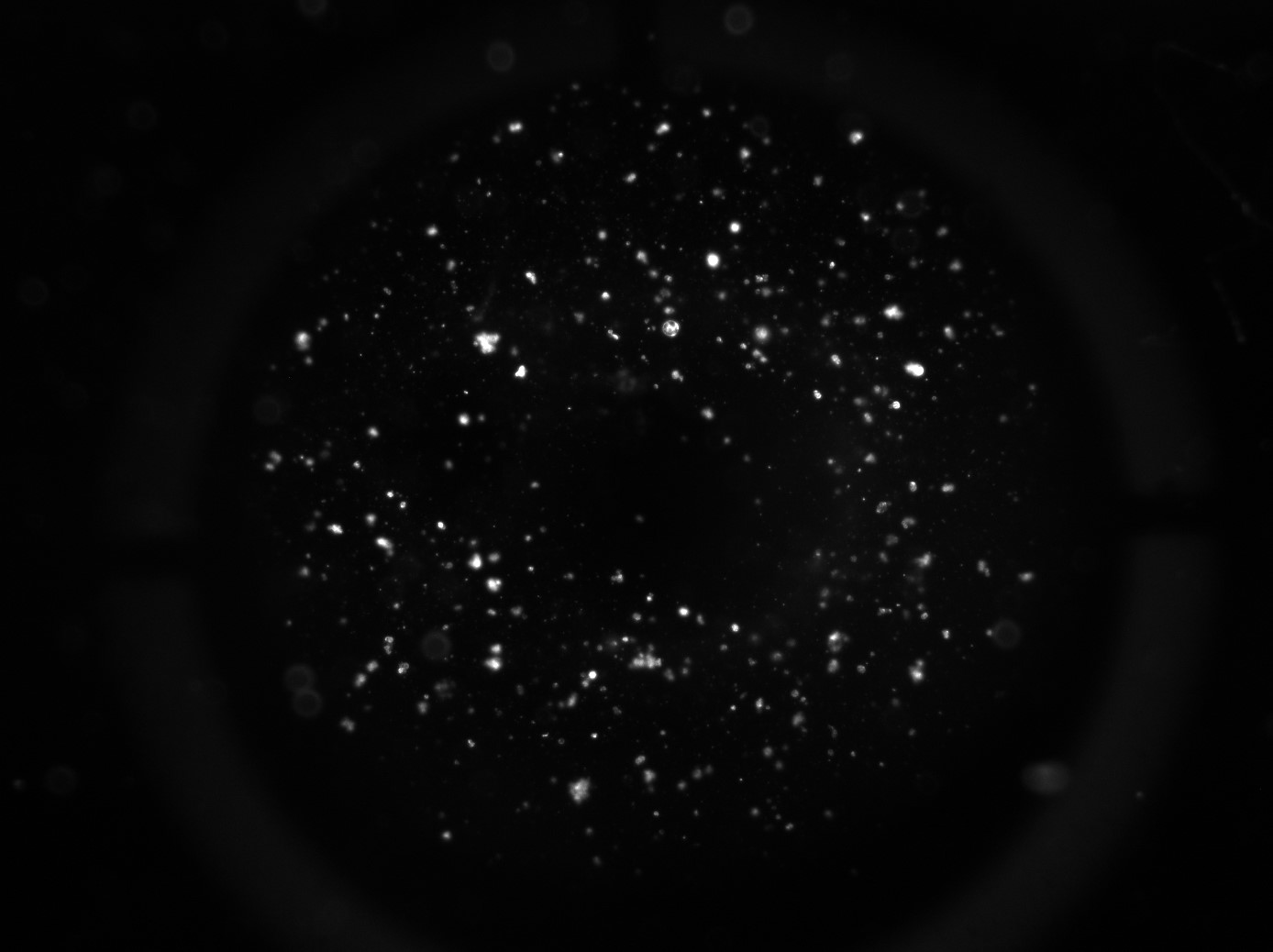

Supplement: Supplementary file 4 — Source Data [file 41467_2024_45605_MOESM4_ESM.zip › Source Data/Figures_Source_Data/figure 3/panel c/C3.jpg]

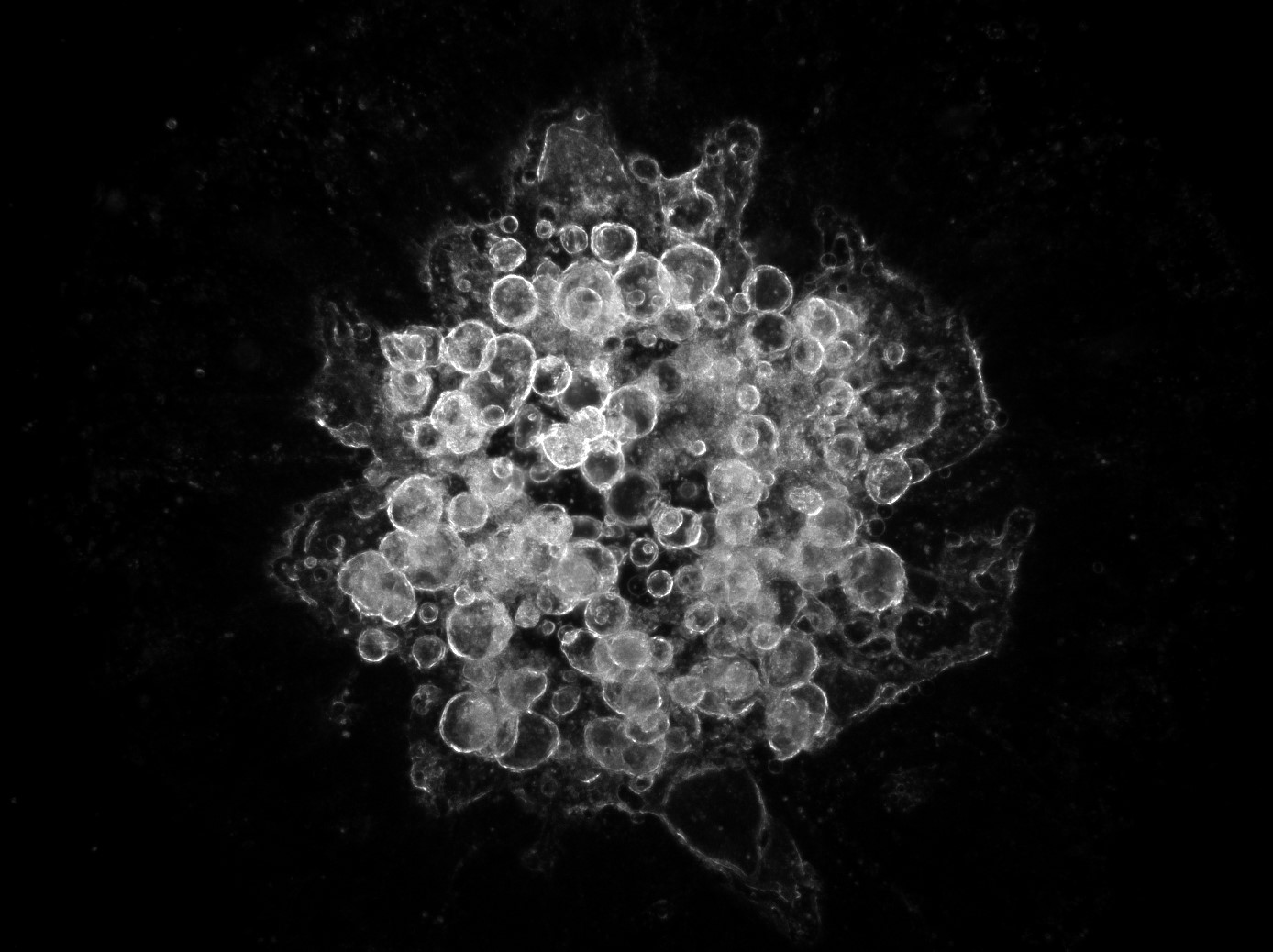

Supplement: Supplementary file 4 — Source Data [file 41467_2024_45605_MOESM4_ESM.zip › Source Data/Figures_Source_Data/figure 3/panel c/C4.jpg]

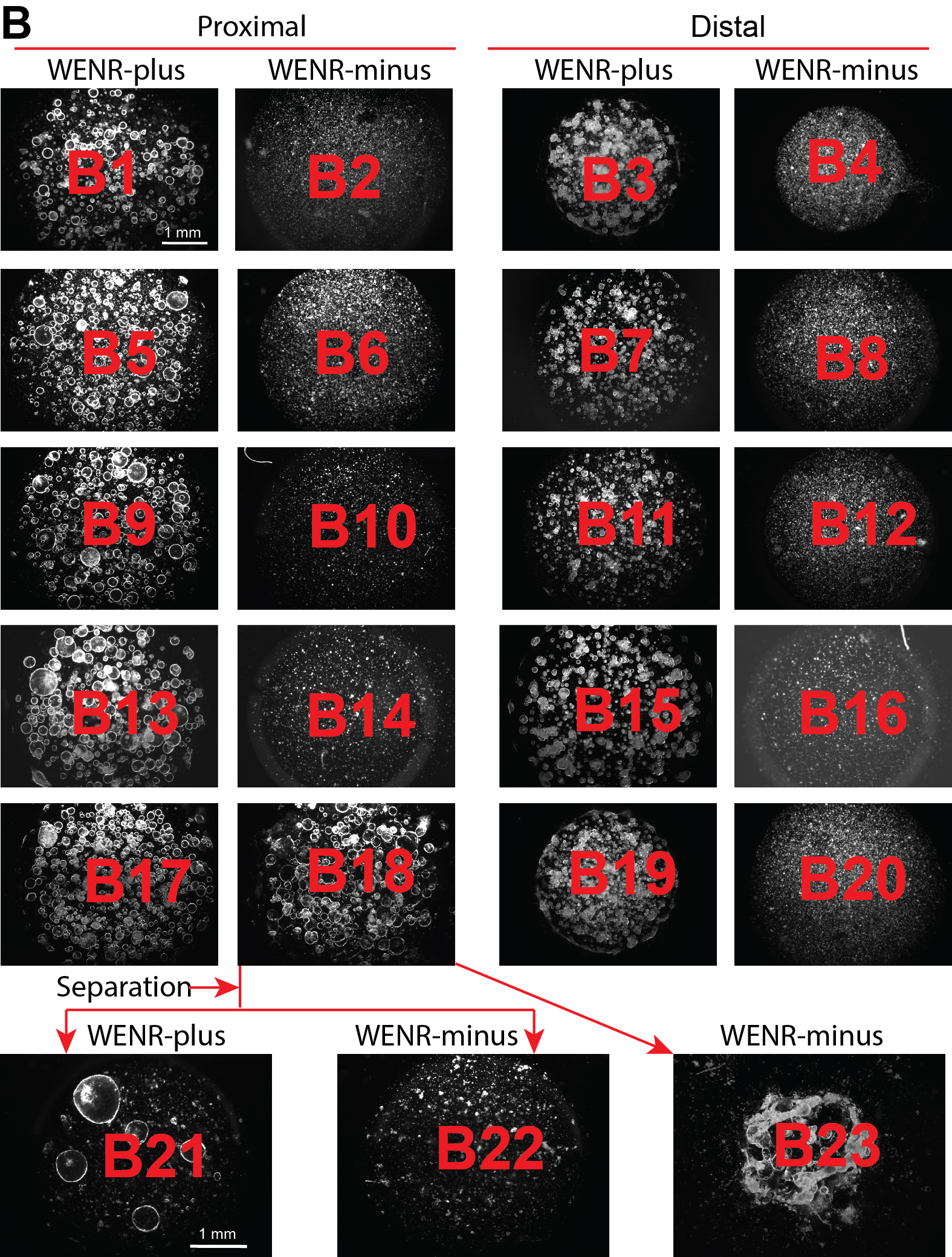

Supplement: Supplementary file 4 — Source Data [file 41467_2024_45605_MOESM4_ESM.zip › Source Data/Figures_Source_Data/figure 2/panel b/figure 2_panel B.jpg]

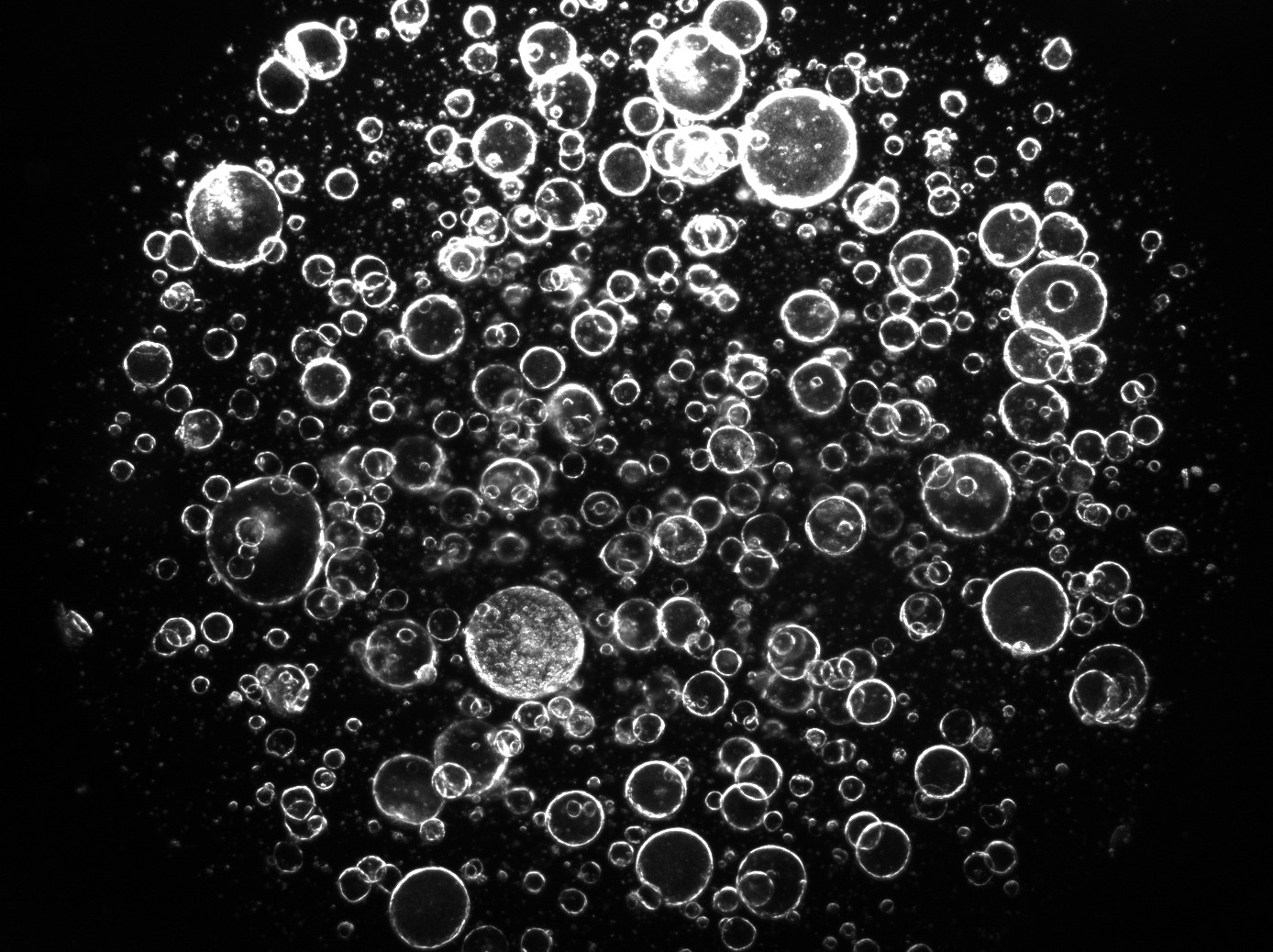

Supplement: Supplementary file 4 — Source Data [file 41467_2024_45605_MOESM4_ESM.zip › Source Data/Figures_Source_Data/figure 2/panel b/B9.jpg]

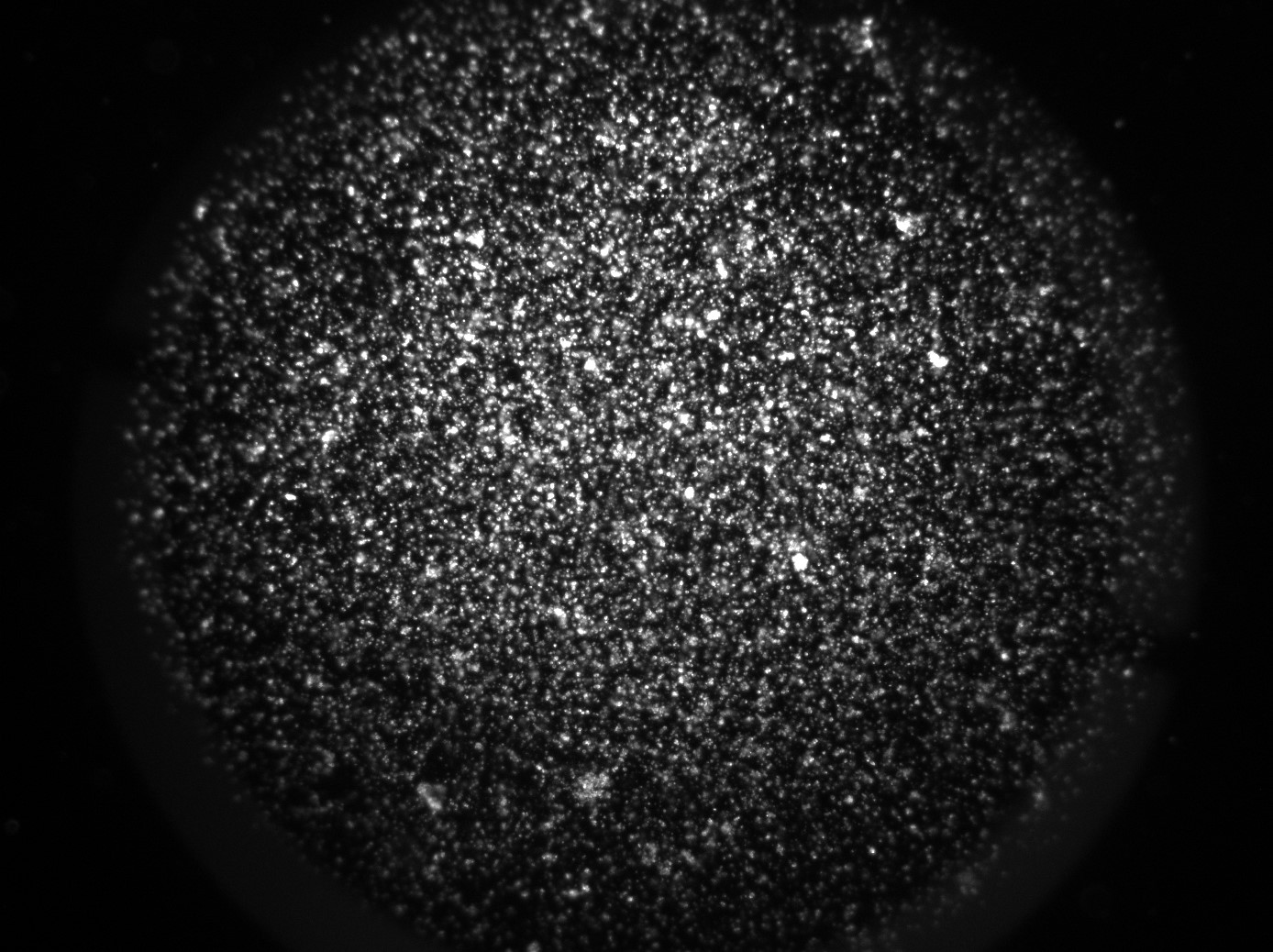

Supplement: Supplementary file 4 — Source Data [file 41467_2024_45605_MOESM4_ESM.zip › Source Data/Figures_Source_Data/figure 2/panel b/B8.jpg]

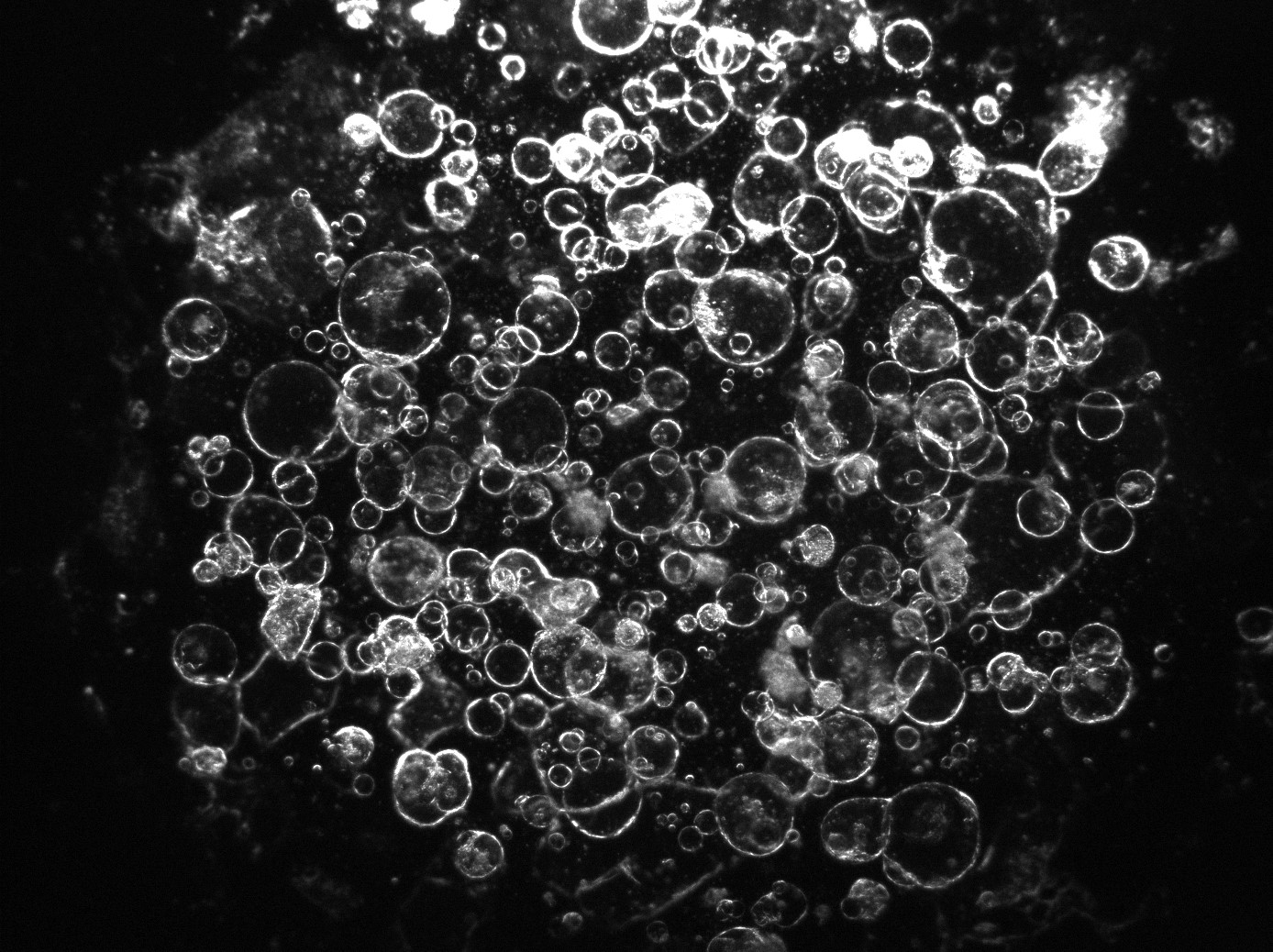

Supplement: Supplementary file 4 — Source Data [file 41467_2024_45605_MOESM4_ESM.zip › Source Data/Figures_Source_Data/figure 2/panel b/B18.jpg]

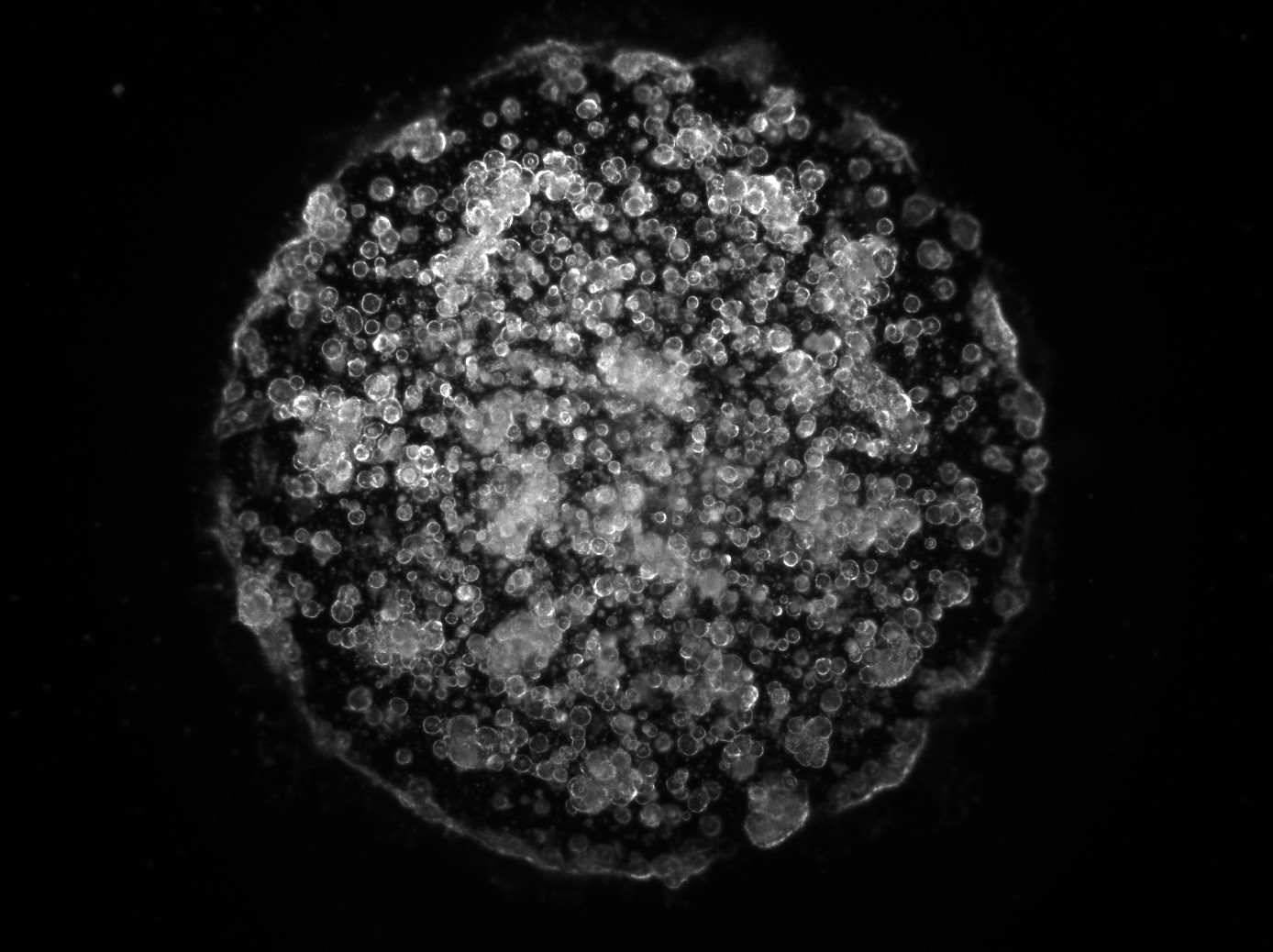

Supplement: Supplementary file 4 — Source Data [file 41467_2024_45605_MOESM4_ESM.zip › Source Data/Figures_Source_Data/figure 2/panel b/B19.jpg]

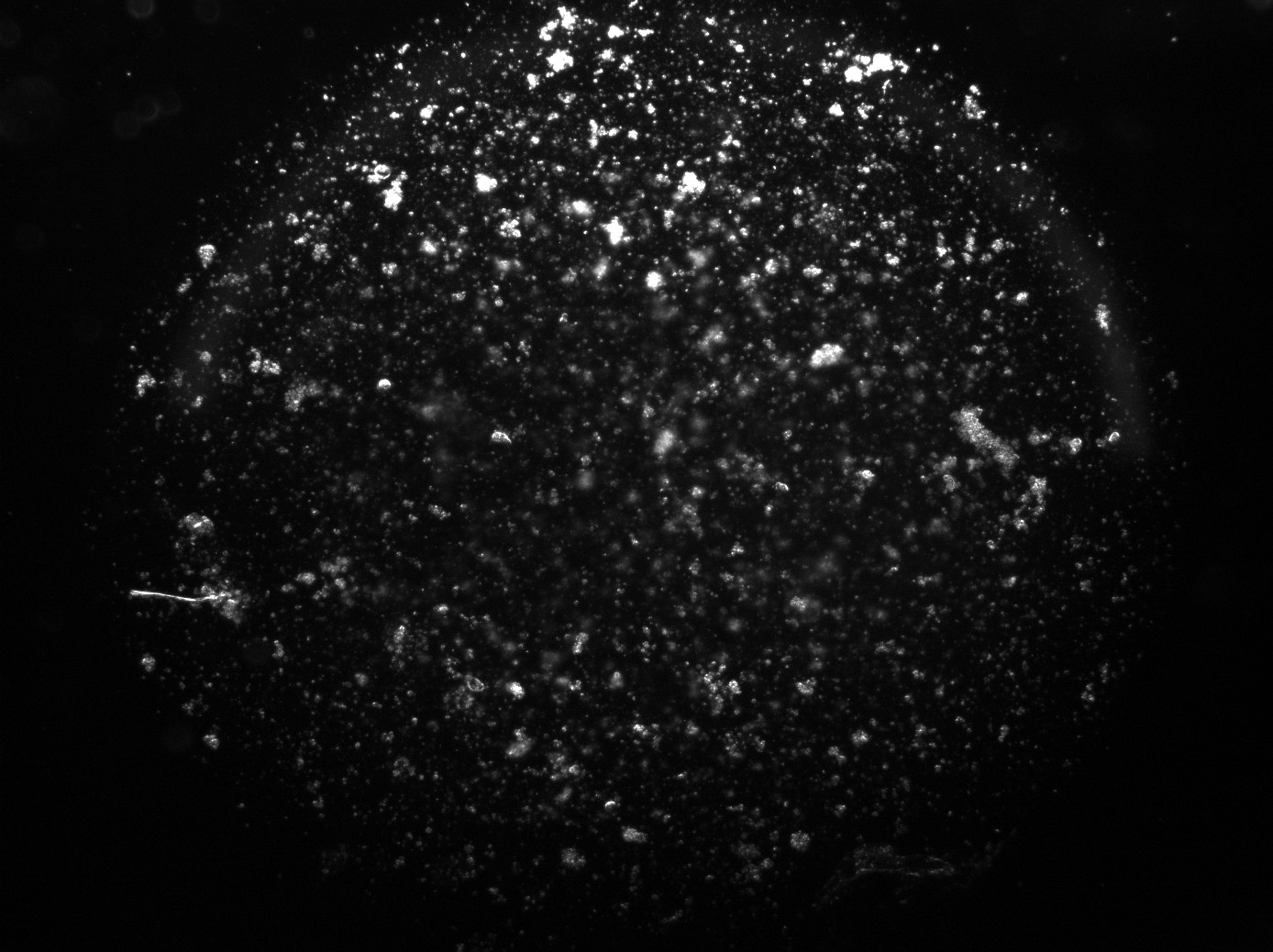

Supplement: Supplementary file 4 — Source Data [file 41467_2024_45605_MOESM4_ESM.zip › Source Data/Figures_Source_Data/figure 2/panel b/B22.jpg]

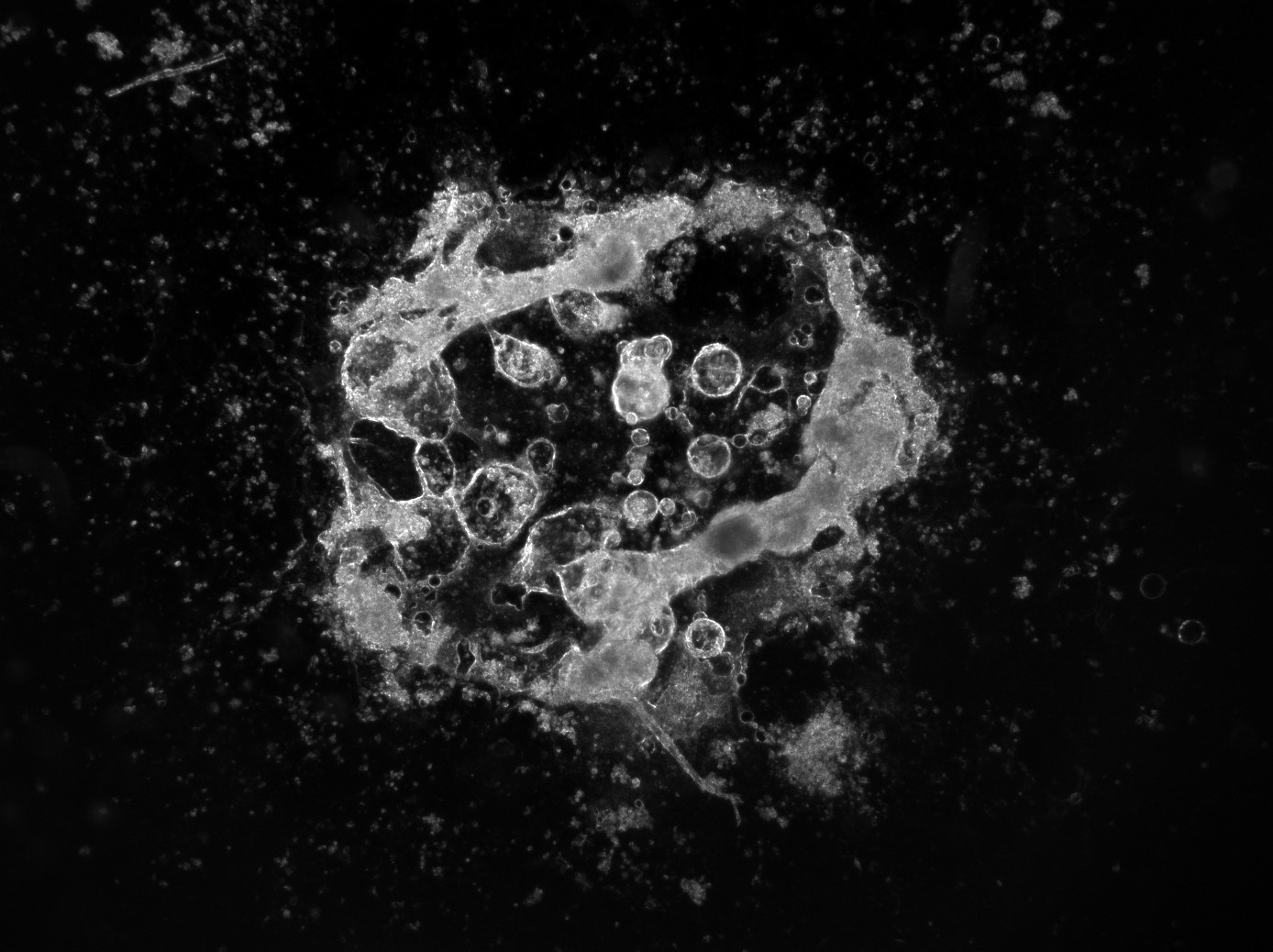

Supplement: Supplementary file 4 — Source Data [file 41467_2024_45605_MOESM4_ESM.zip › Source Data/Figures_Source_Data/figure 2/panel b/B23.jpg]

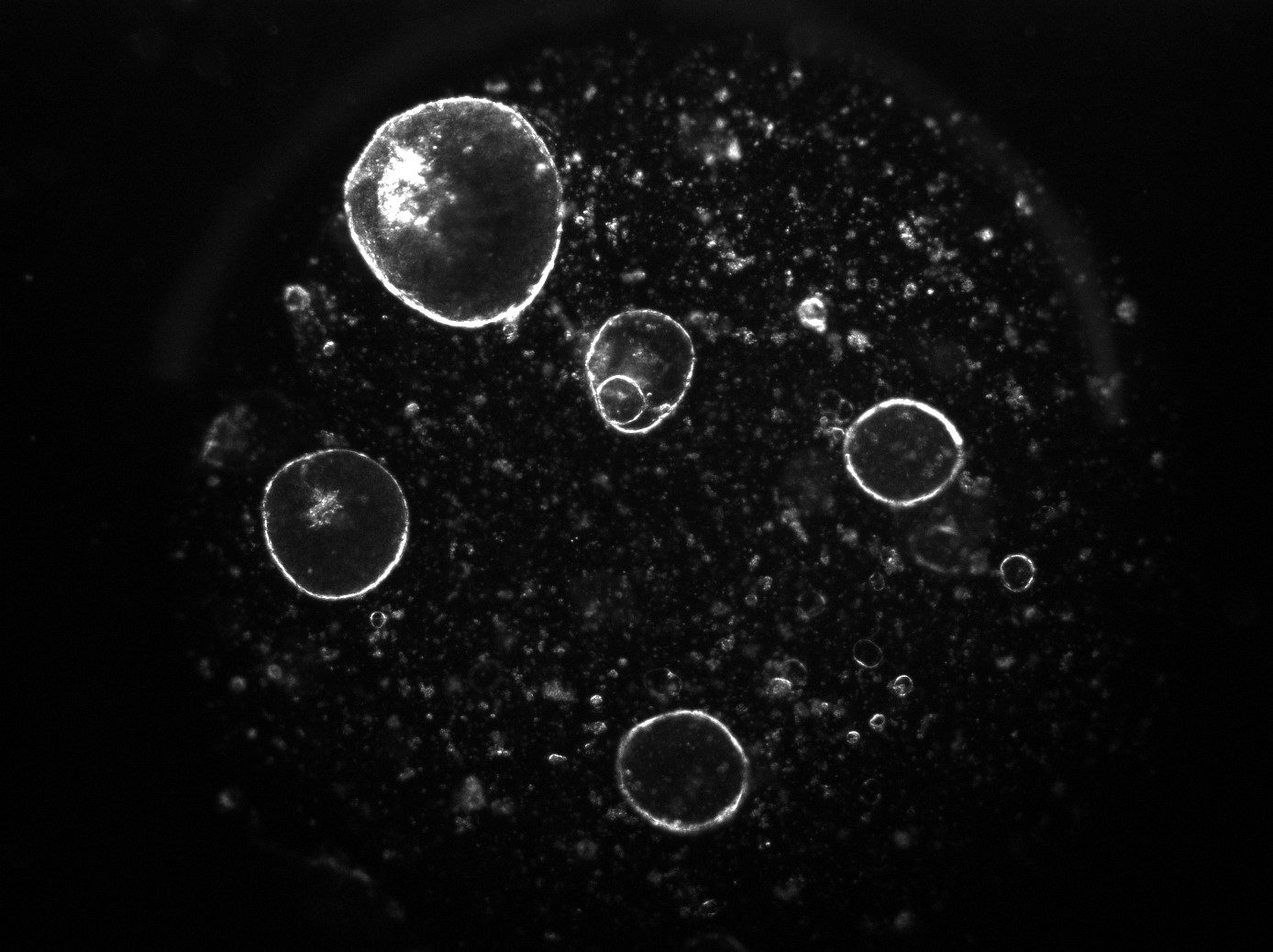

Supplement: Supplementary file 4 — Source Data [file 41467_2024_45605_MOESM4_ESM.zip › Source Data/Figures_Source_Data/figure 2/panel b/B21.jpg]

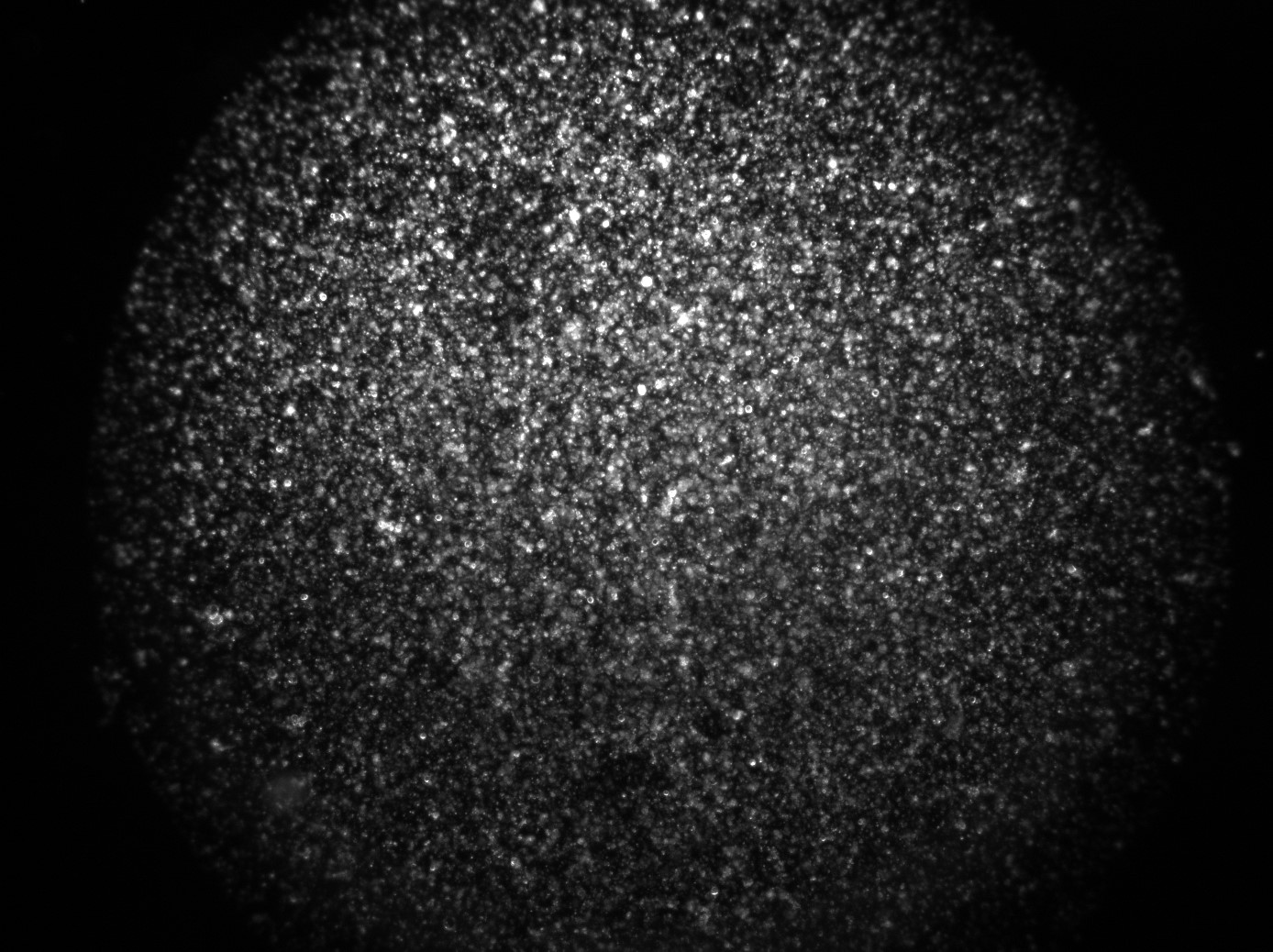

Supplement: Supplementary file 4 — Source Data [file 41467_2024_45605_MOESM4_ESM.zip › Source Data/Figures_Source_Data/figure 2/panel b/B20.jpg]

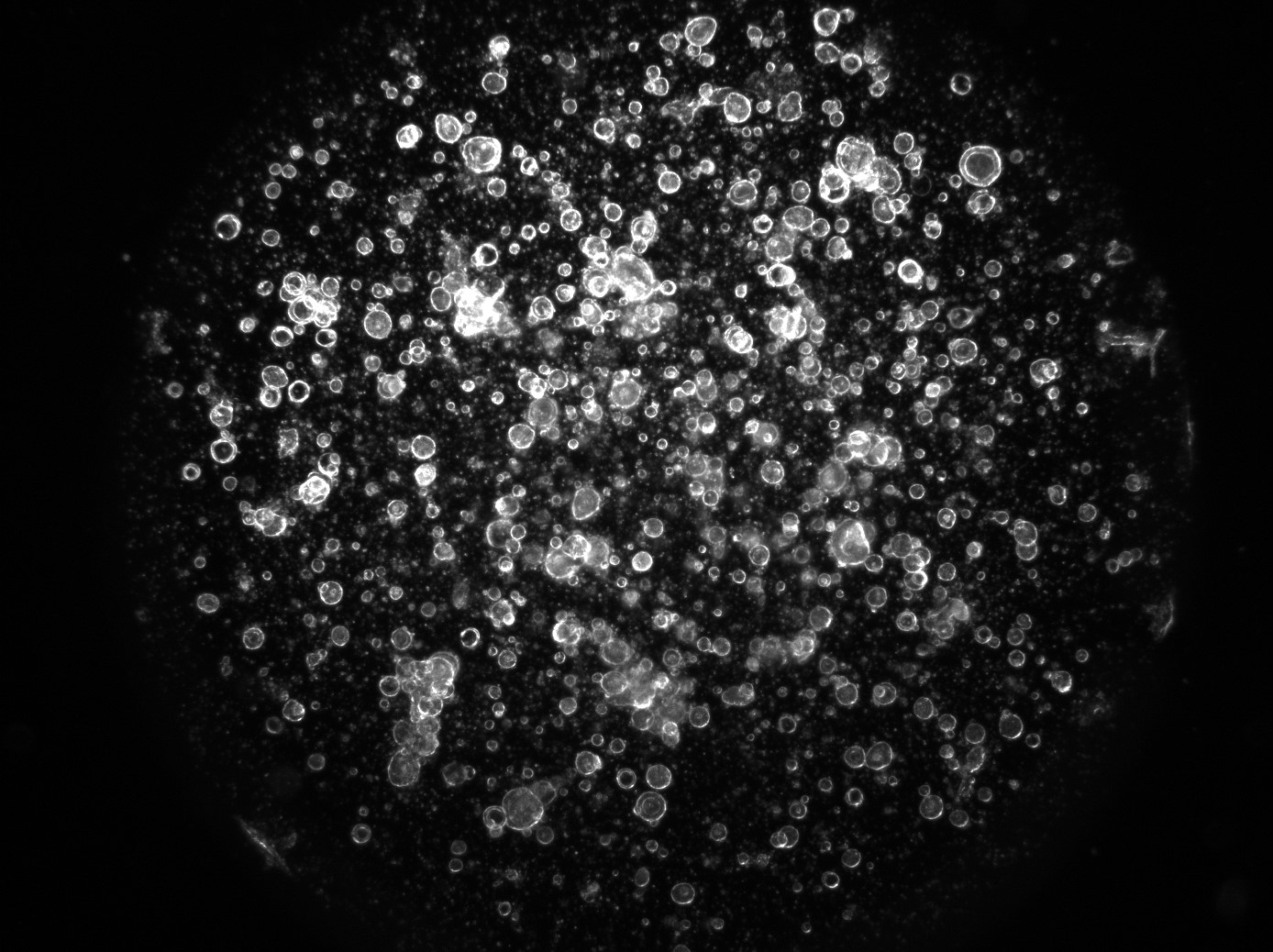

Supplement: Supplementary file 4 — Source Data [file 41467_2024_45605_MOESM4_ESM.zip › Source Data/Figures_Source_Data/figure 2/panel b/B11.jpg]

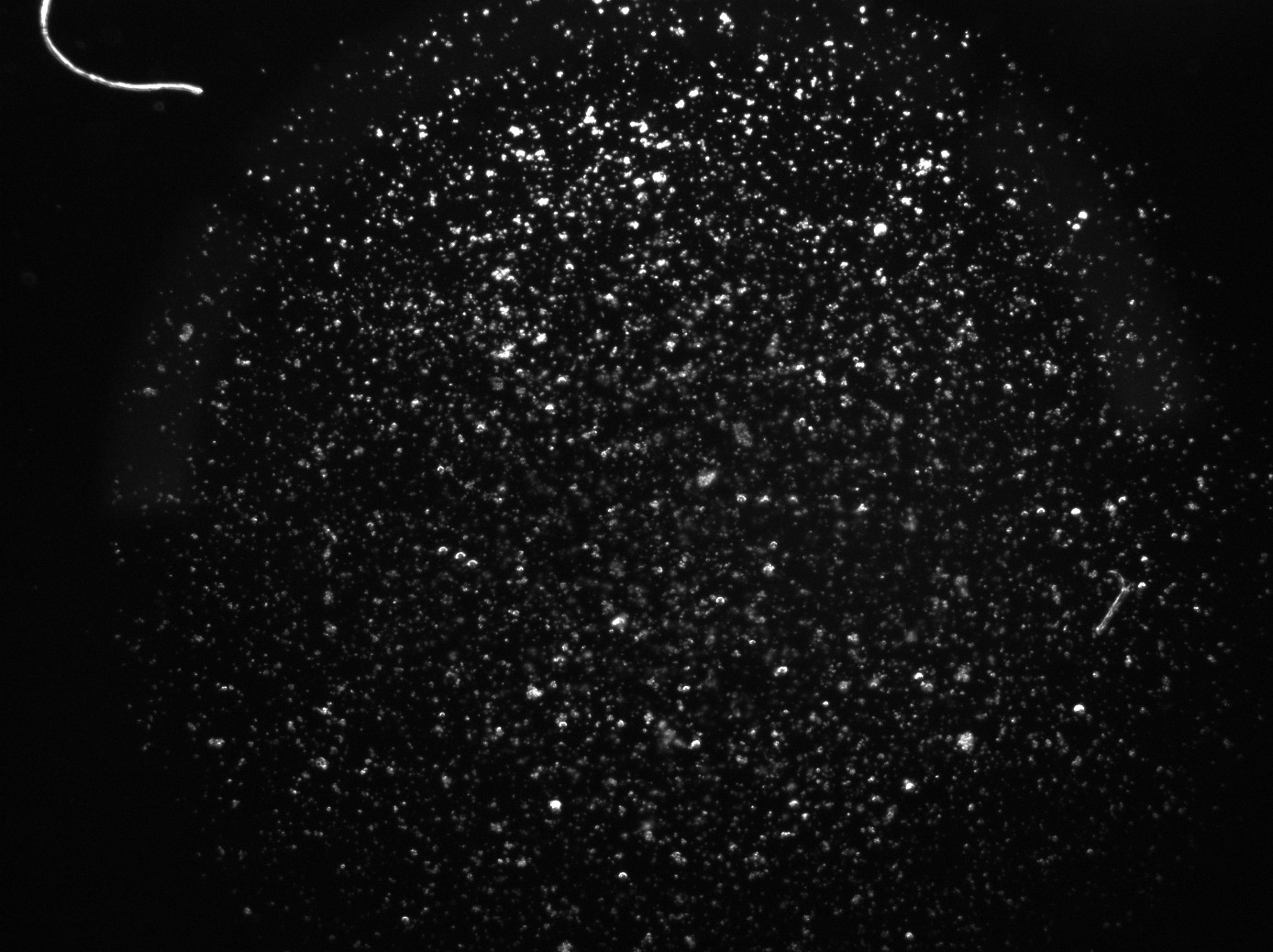

Supplement: Supplementary file 4 — Source Data [file 41467_2024_45605_MOESM4_ESM.zip › Source Data/Figures_Source_Data/figure 2/panel b/B10.jpg]

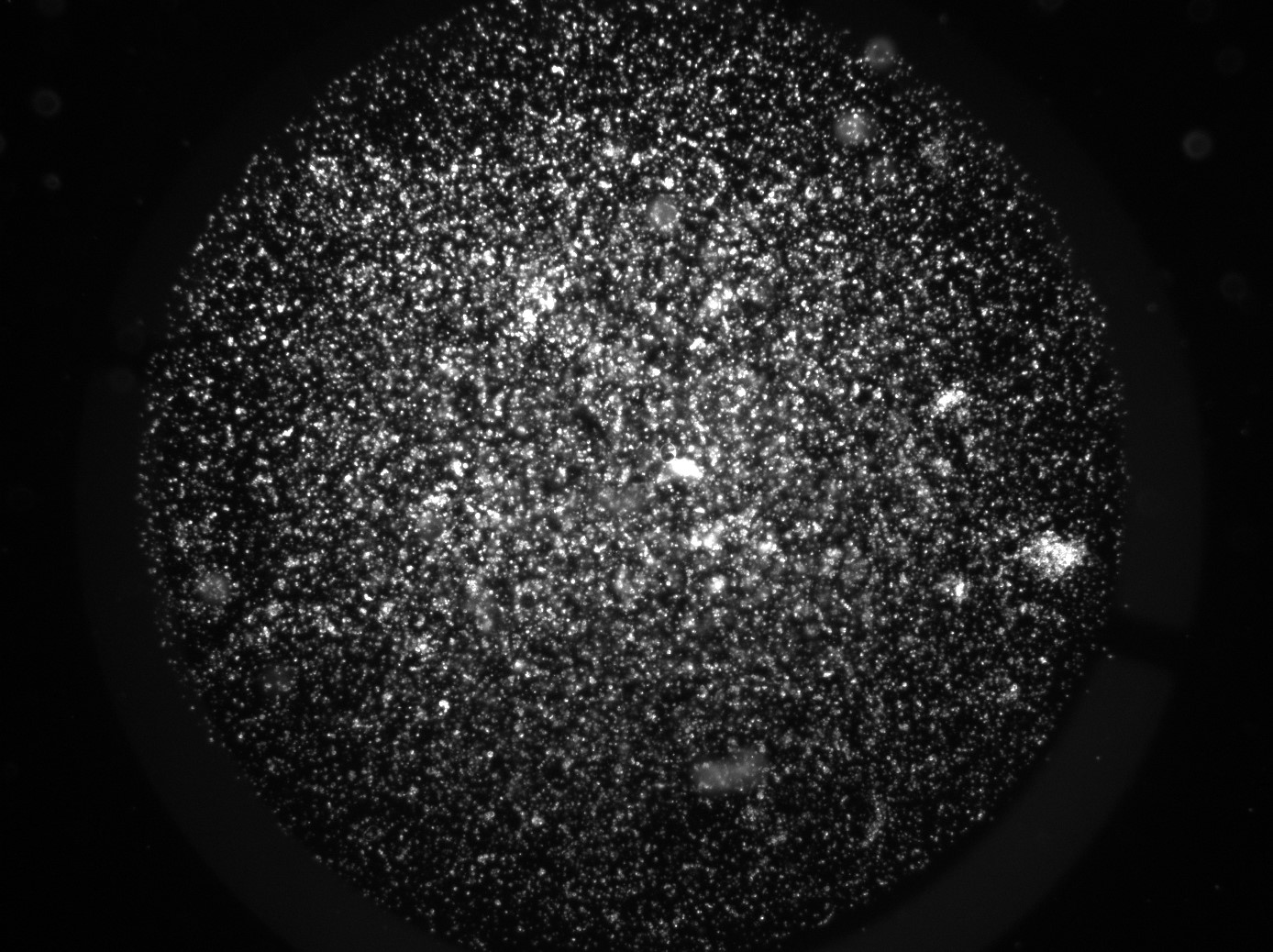

Supplement: Supplementary file 4 — Source Data [file 41467_2024_45605_MOESM4_ESM.zip › Source Data/Figures_Source_Data/figure 2/panel b/B12.jpg]

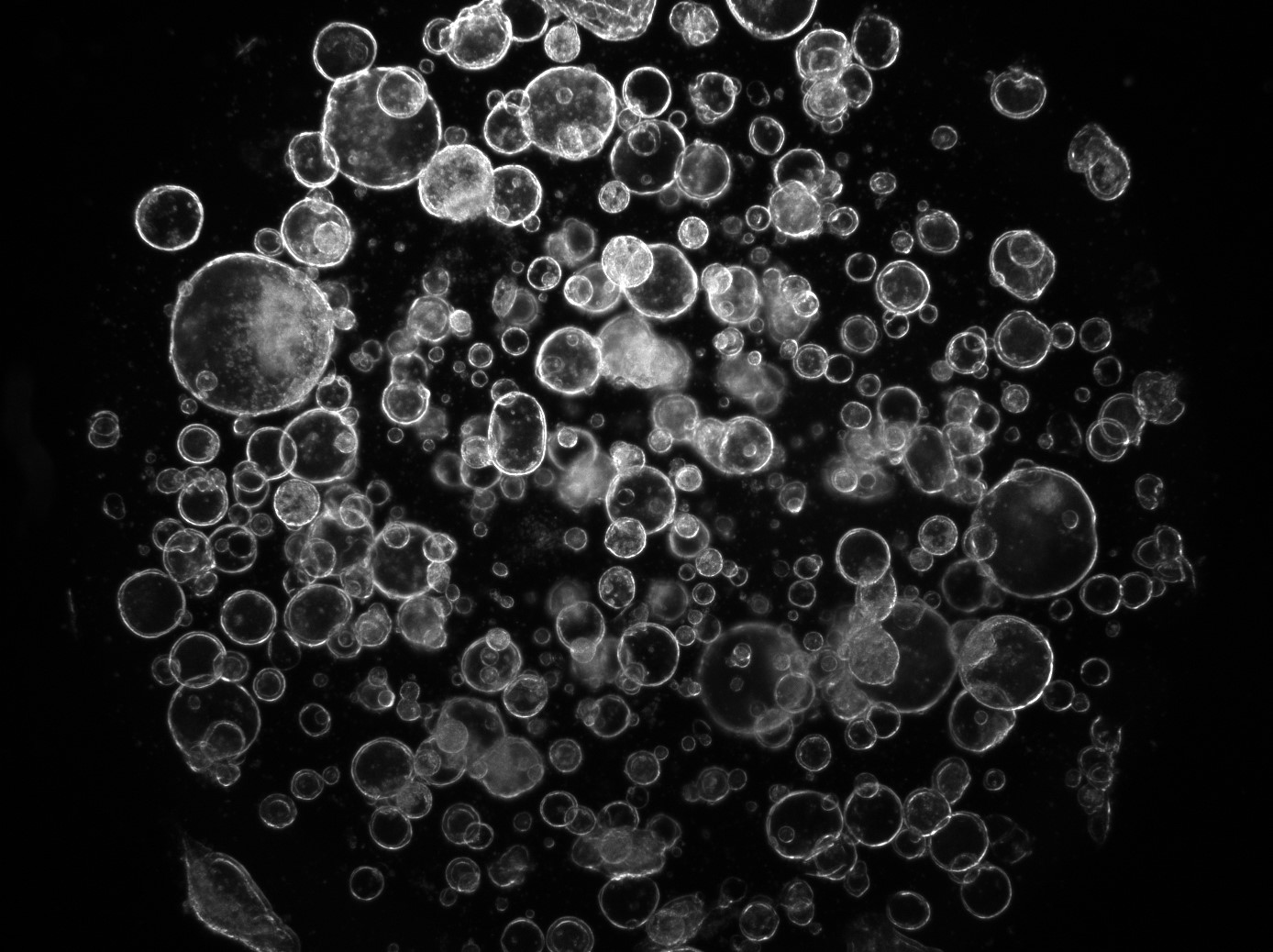

Supplement: Supplementary file 4 — Source Data [file 41467_2024_45605_MOESM4_ESM.zip › Source Data/Figures_Source_Data/figure 2/panel b/B13.jpg]

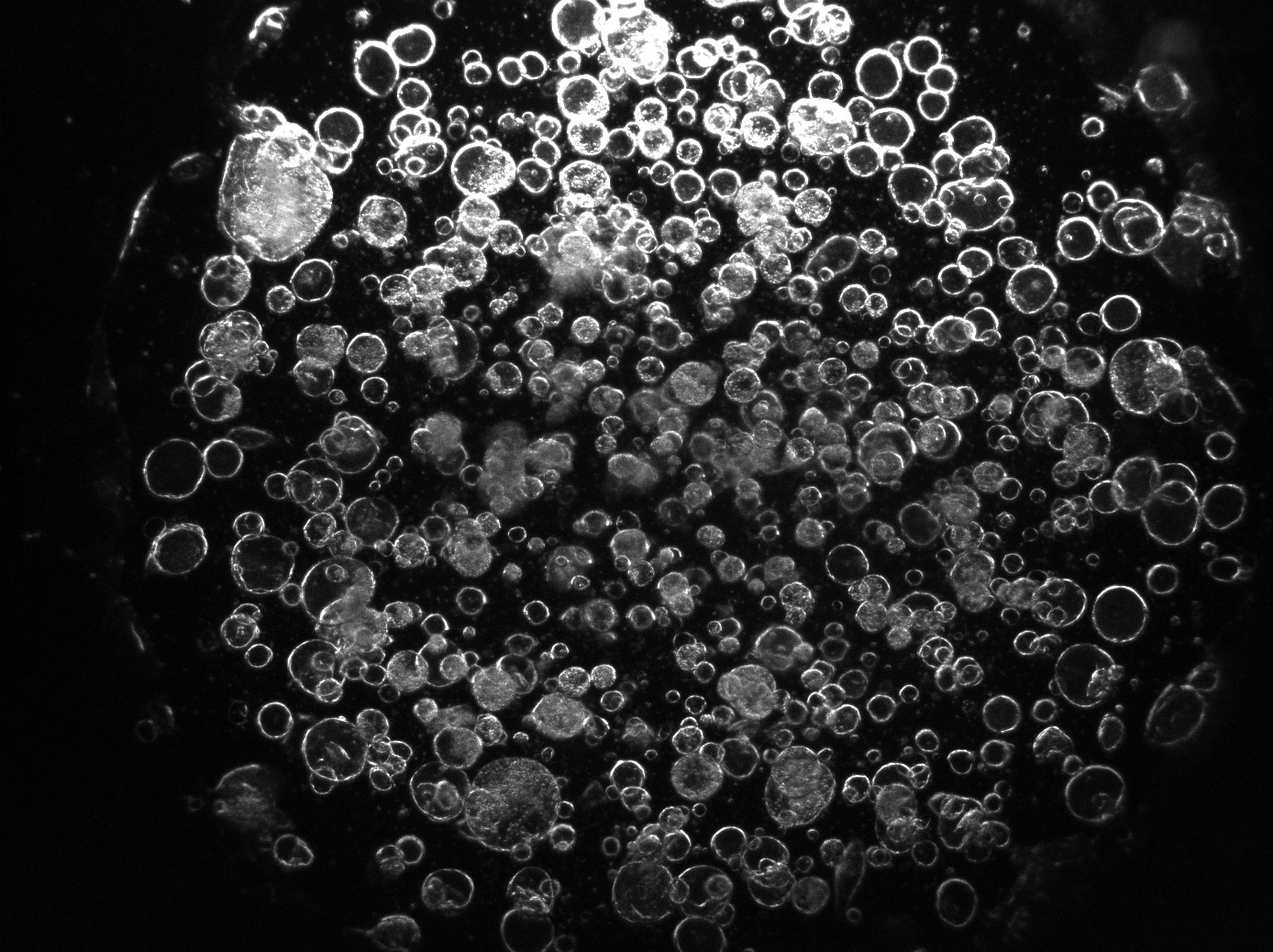

Supplement: Supplementary file 4 — Source Data [file 41467_2024_45605_MOESM4_ESM.zip › Source Data/Figures_Source_Data/figure 2/panel b/B17.jpg]

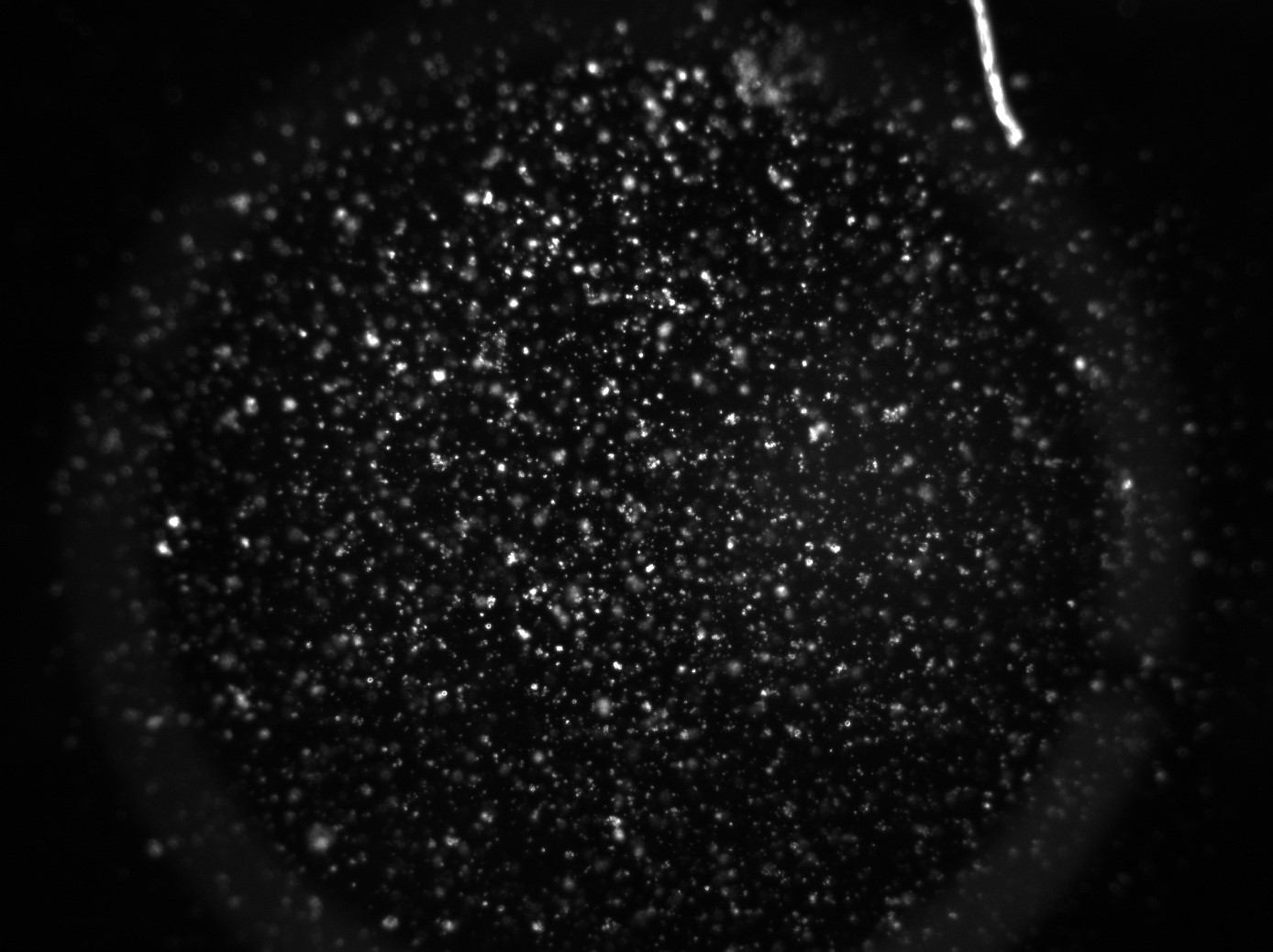

Supplement: Supplementary file 4 — Source Data [file 41467_2024_45605_MOESM4_ESM.zip › Source Data/Figures_Source_Data/figure 2/panel b/B16.jpg]

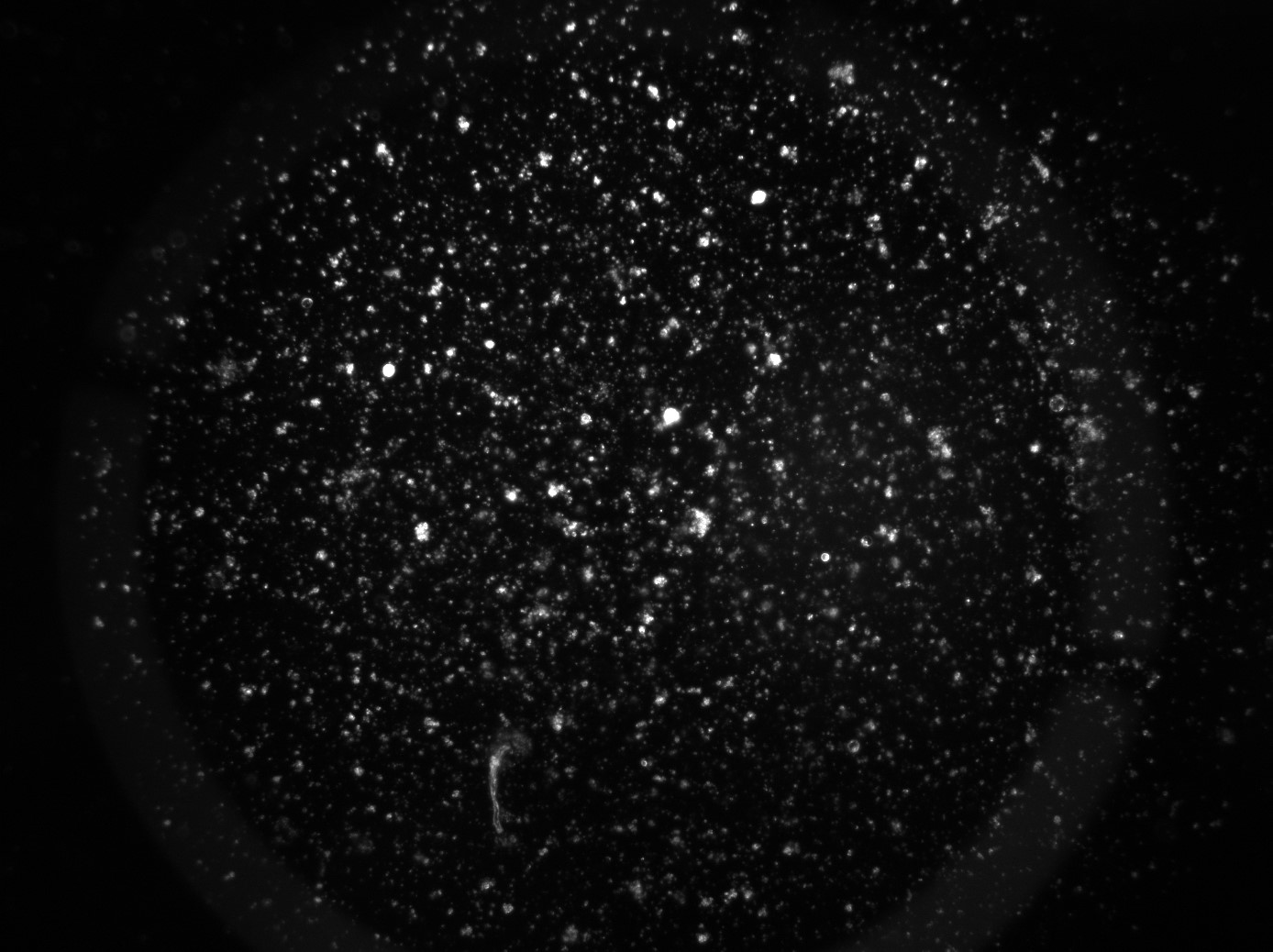

Supplement: Supplementary file 4 — Source Data [file 41467_2024_45605_MOESM4_ESM.zip › Source Data/Figures_Source_Data/figure 2/panel b/B14.jpg]

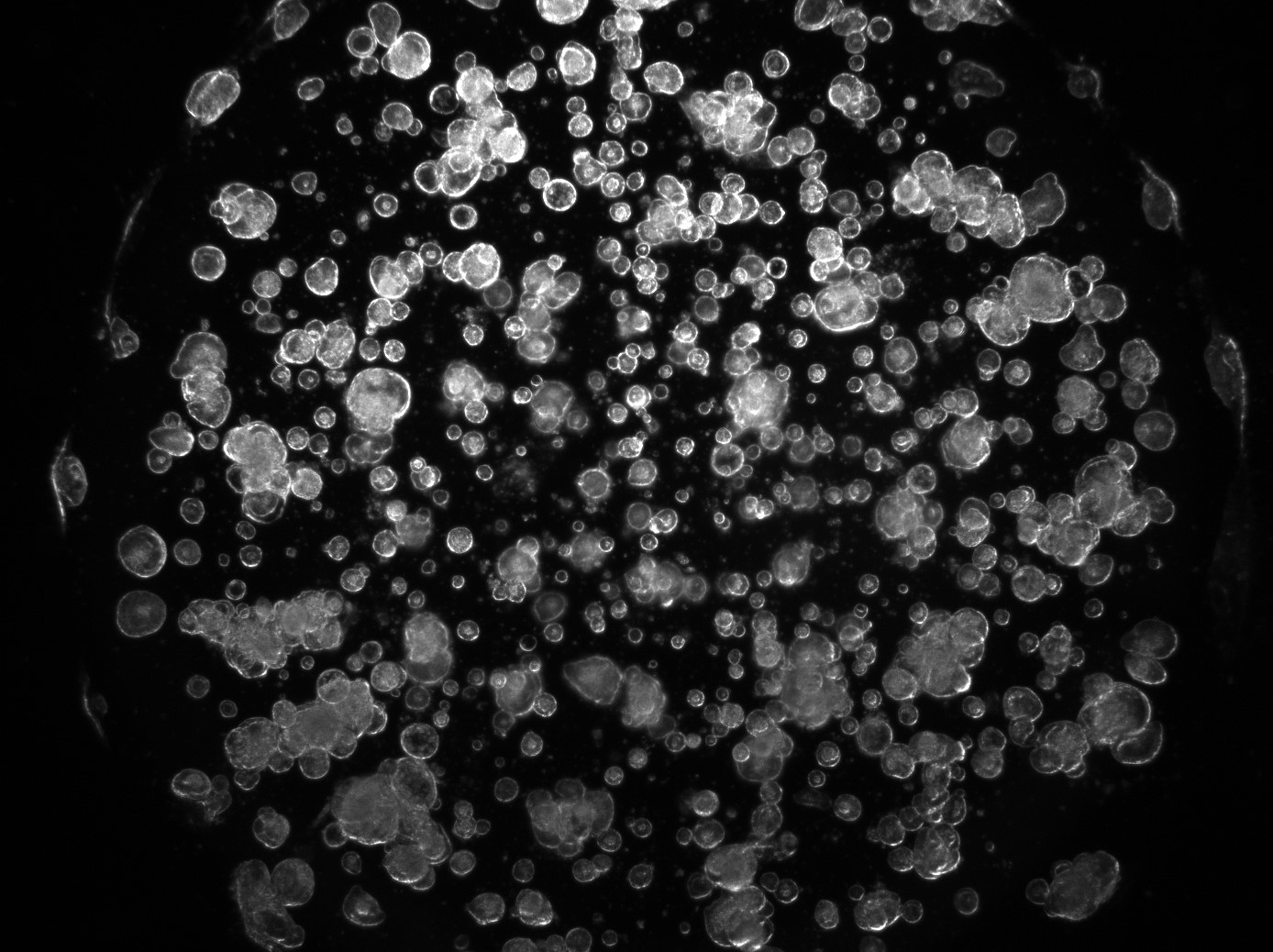

Supplement: Supplementary file 4 — Source Data [file 41467_2024_45605_MOESM4_ESM.zip › Source Data/Figures_Source_Data/figure 2/panel b/B15.jpg]

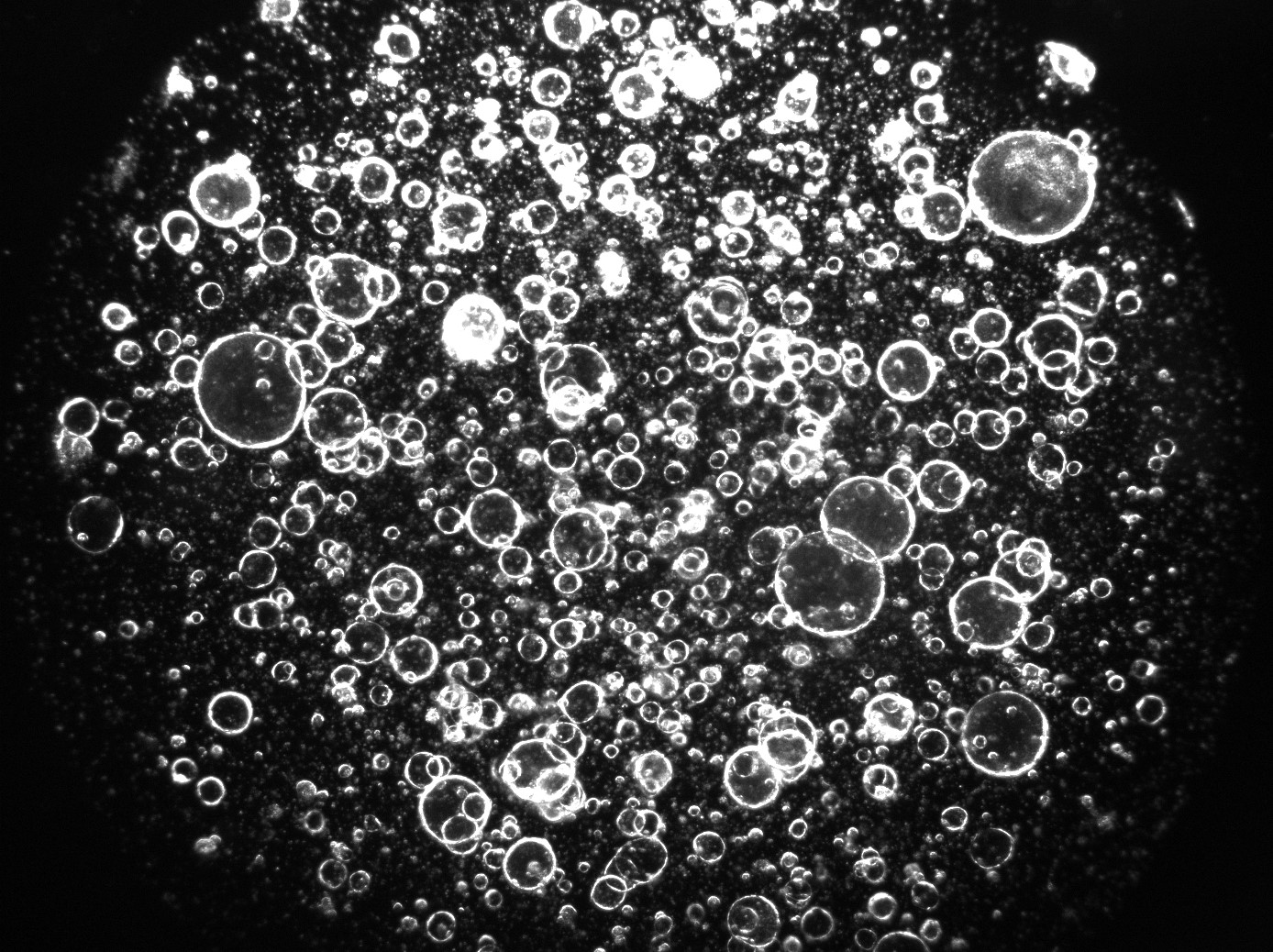

Supplement: Supplementary file 4 — Source Data [file 41467_2024_45605_MOESM4_ESM.zip › Source Data/Figures_Source_Data/figure 2/panel b/B5.jpg]

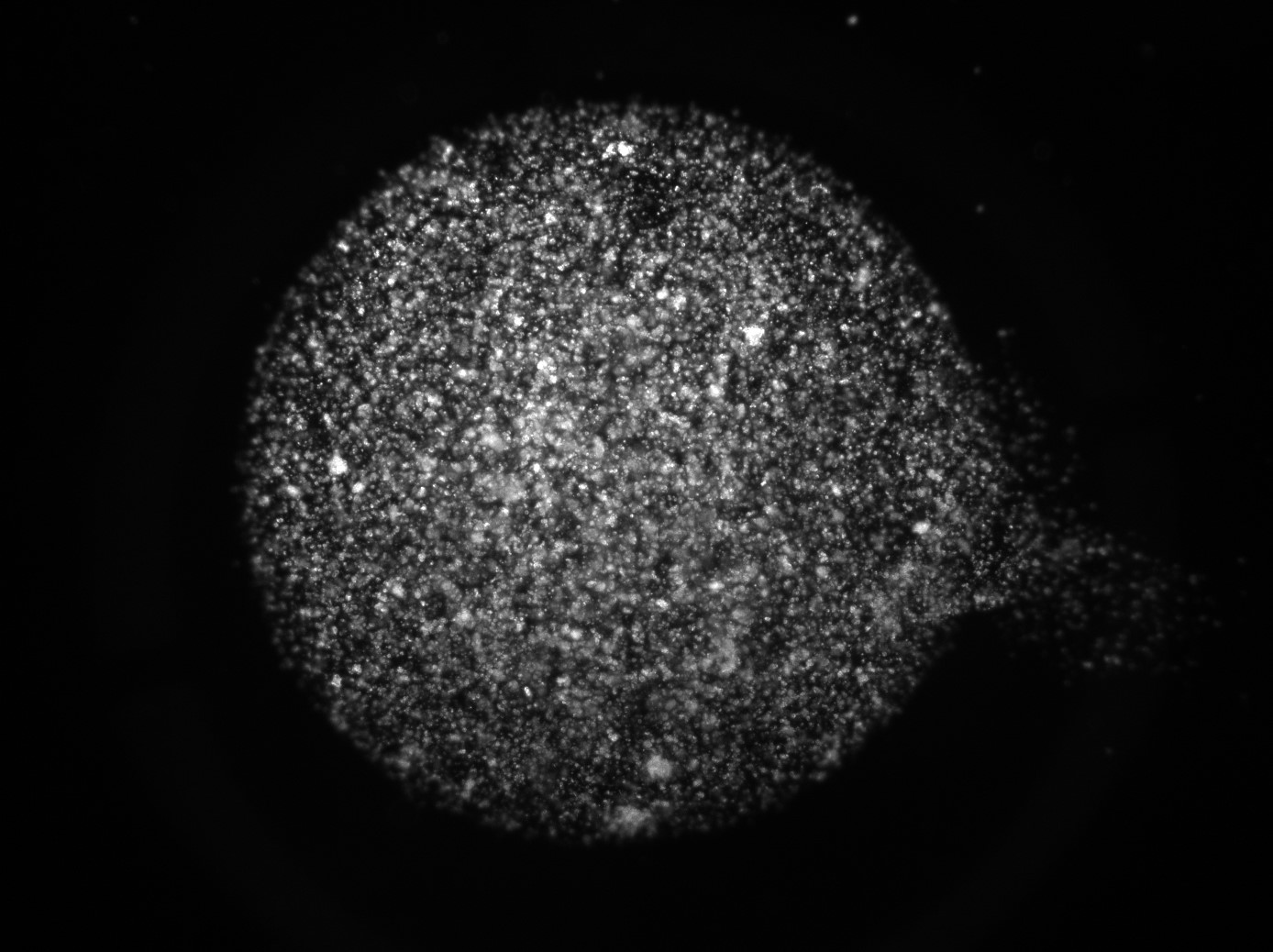

Supplement: Supplementary file 4 — Source Data [file 41467_2024_45605_MOESM4_ESM.zip › Source Data/Figures_Source_Data/figure 2/panel b/B4.jpg]

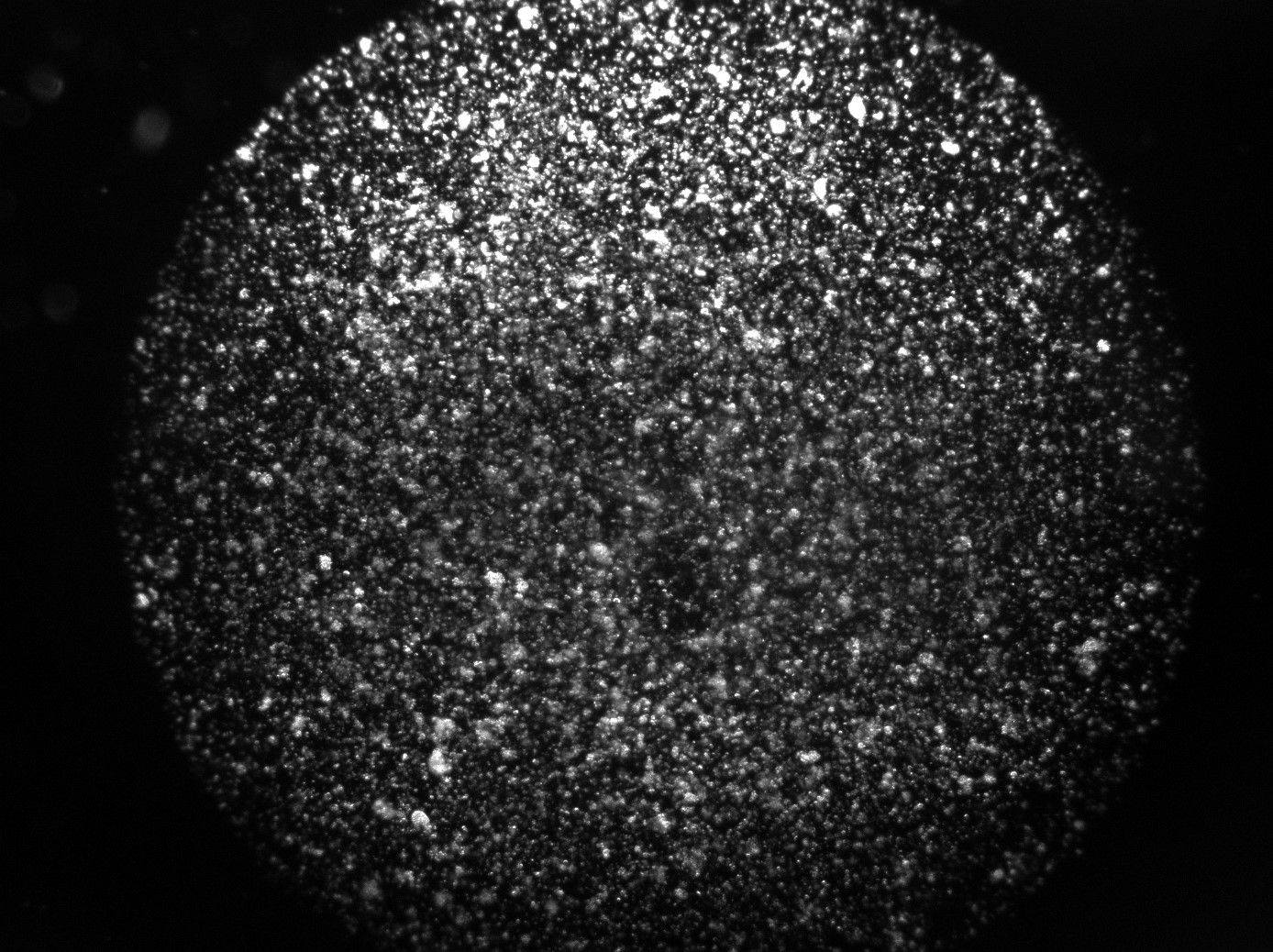

Supplement: Supplementary file 4 — Source Data [file 41467_2024_45605_MOESM4_ESM.zip › Source Data/Figures_Source_Data/figure 2/panel b/B6.jpg]

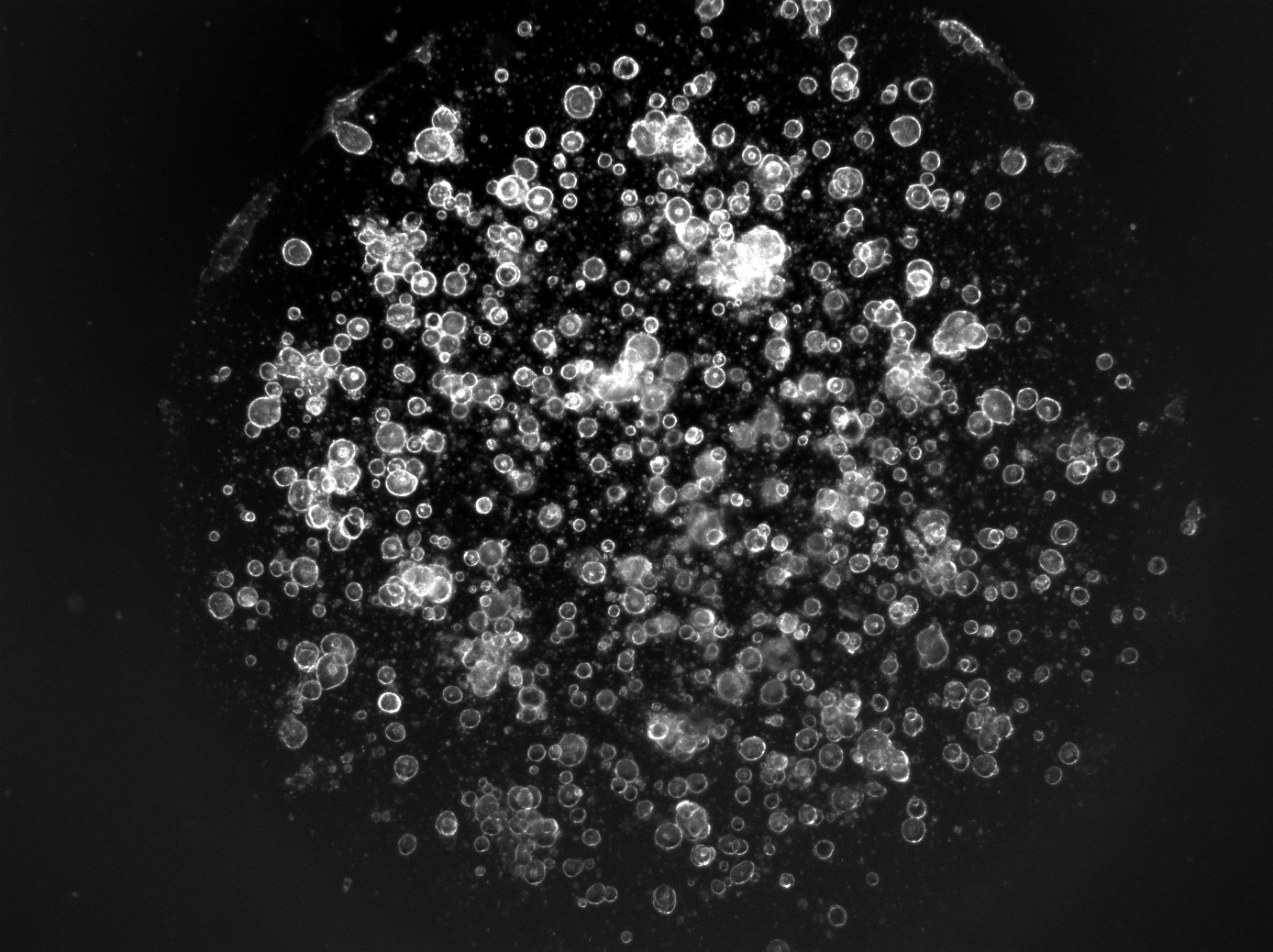

Supplement: Supplementary file 4 — Source Data [file 41467_2024_45605_MOESM4_ESM.zip › Source Data/Figures_Source_Data/figure 2/panel b/B7.jpg]

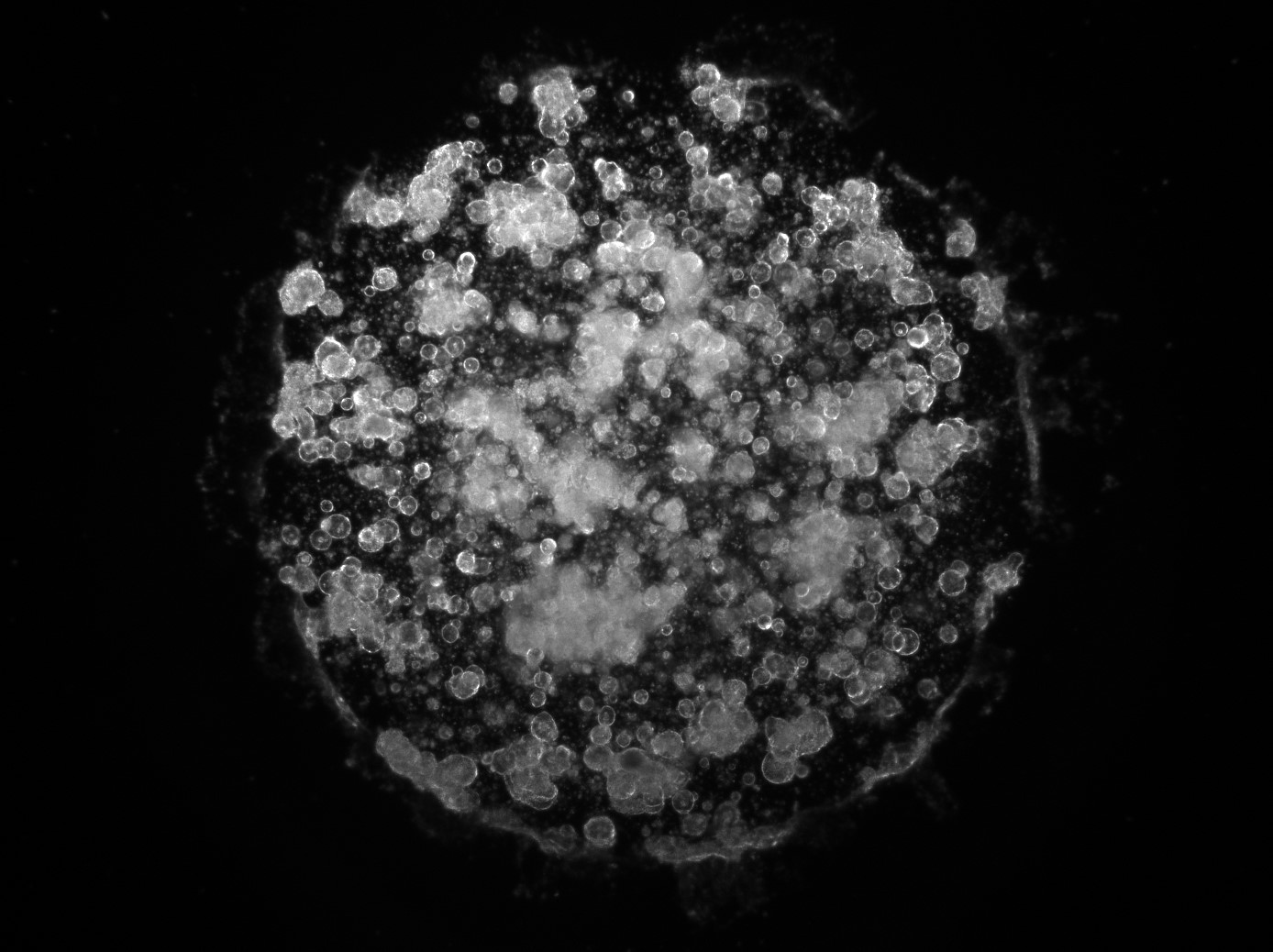

Supplement: Supplementary file 4 — Source Data [file 41467_2024_45605_MOESM4_ESM.zip › Source Data/Figures_Source_Data/figure 2/panel b/B3.jpg]

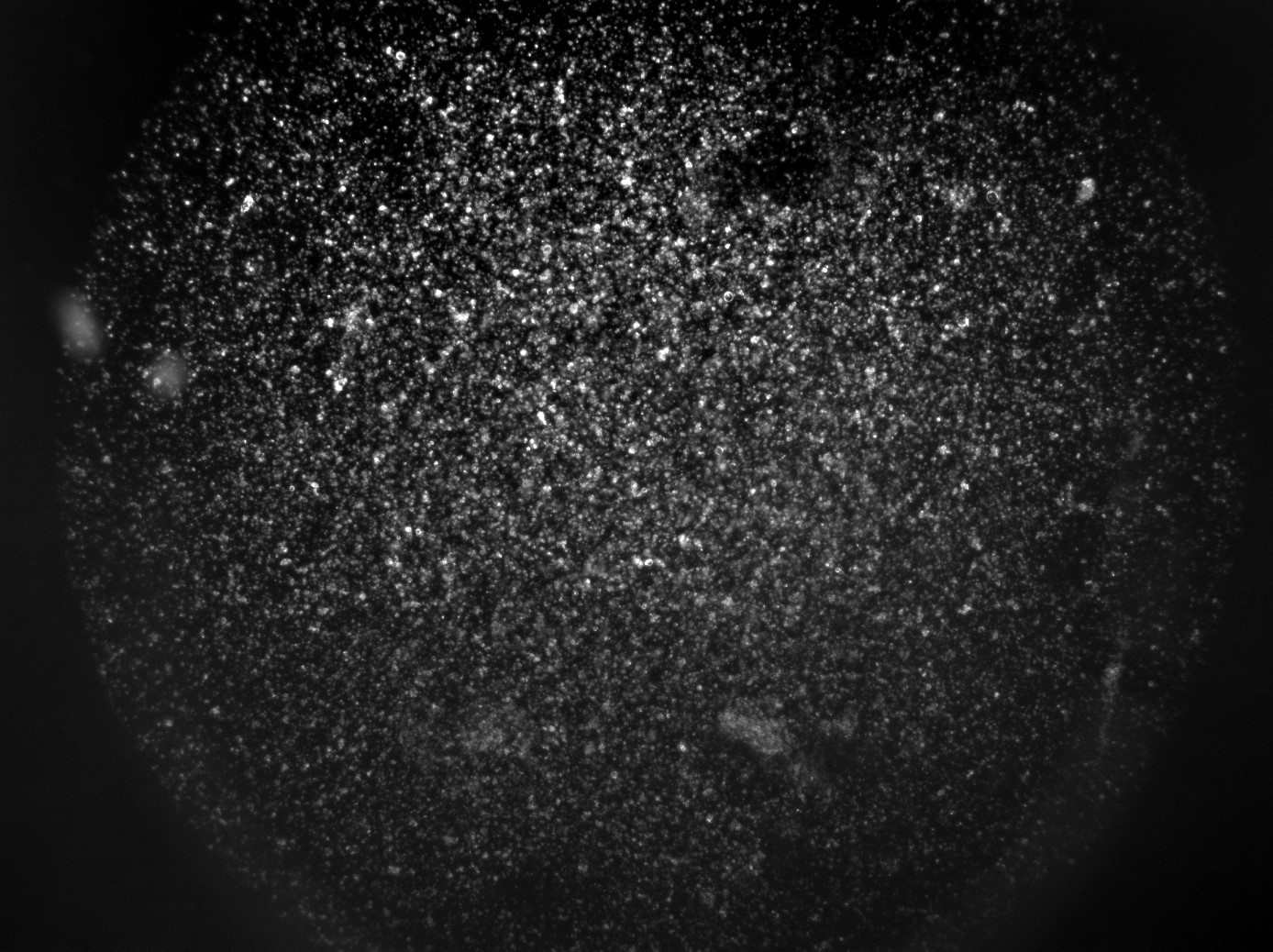

Supplement: Supplementary file 4 — Source Data [file 41467_2024_45605_MOESM4_ESM.zip › Source Data/Figures_Source_Data/figure 2/panel b/B2.jpg]

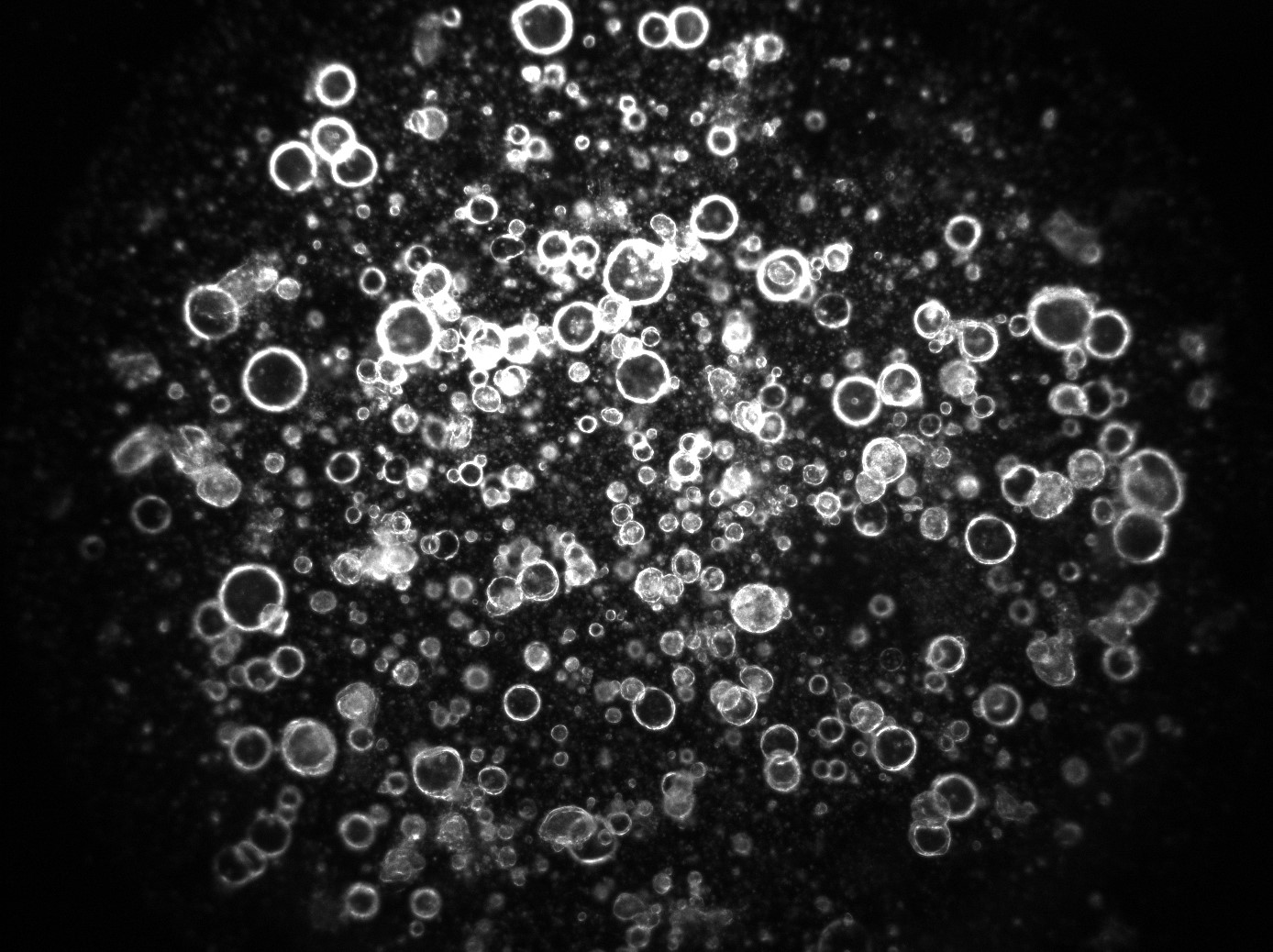

Supplement: Supplementary file 4 — Source Data [file 41467_2024_45605_MOESM4_ESM.zip › Source Data/Figures_Source_Data/figure 2/panel b/B1.jpg]

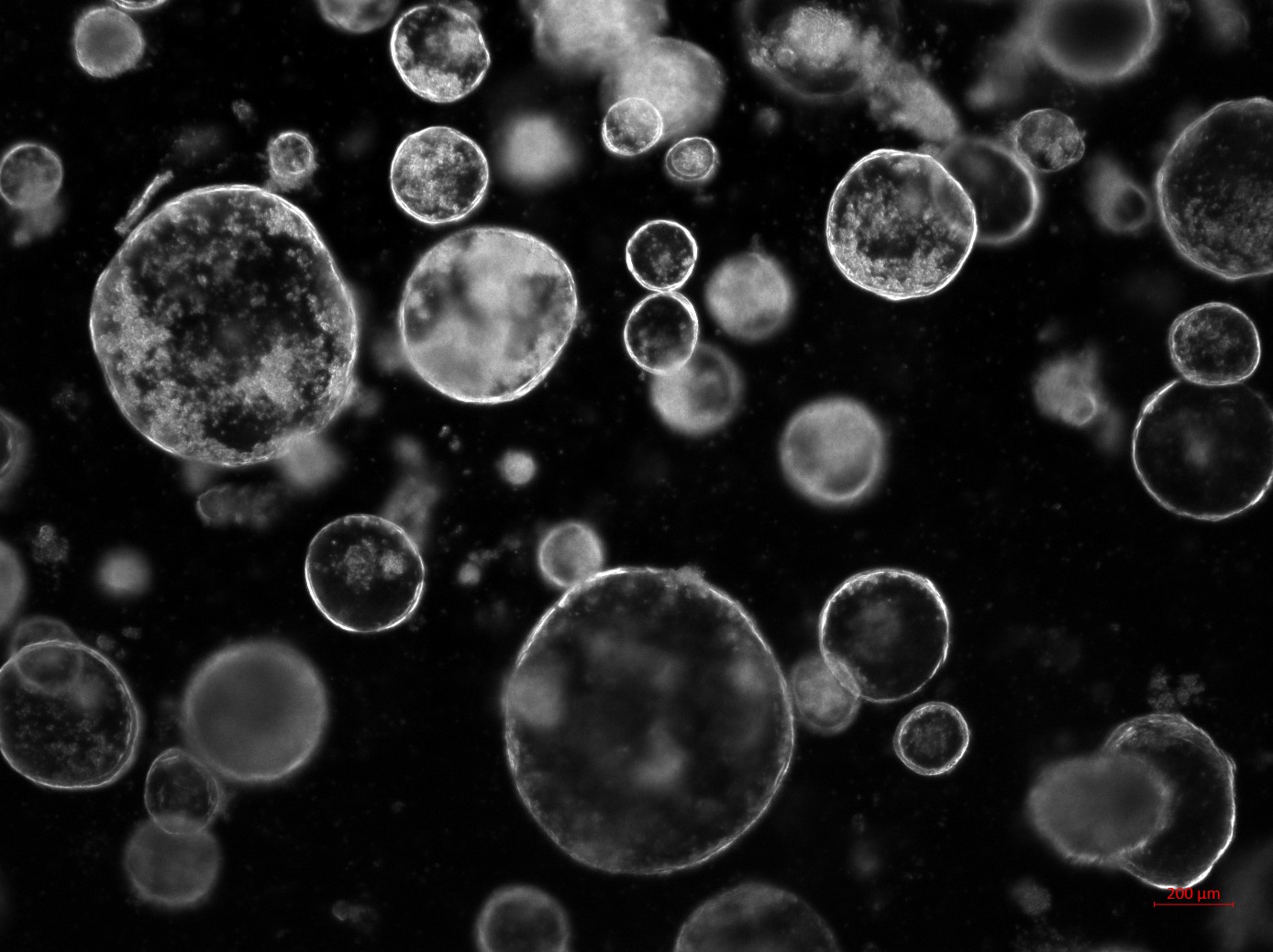

Supplement: Supplementary file 4 — Source Data [file 41467_2024_45605_MOESM4_ESM.zip › Source Data/Figures_Source_Data/supplemental figure 1/panel d,e/D6.jpg]

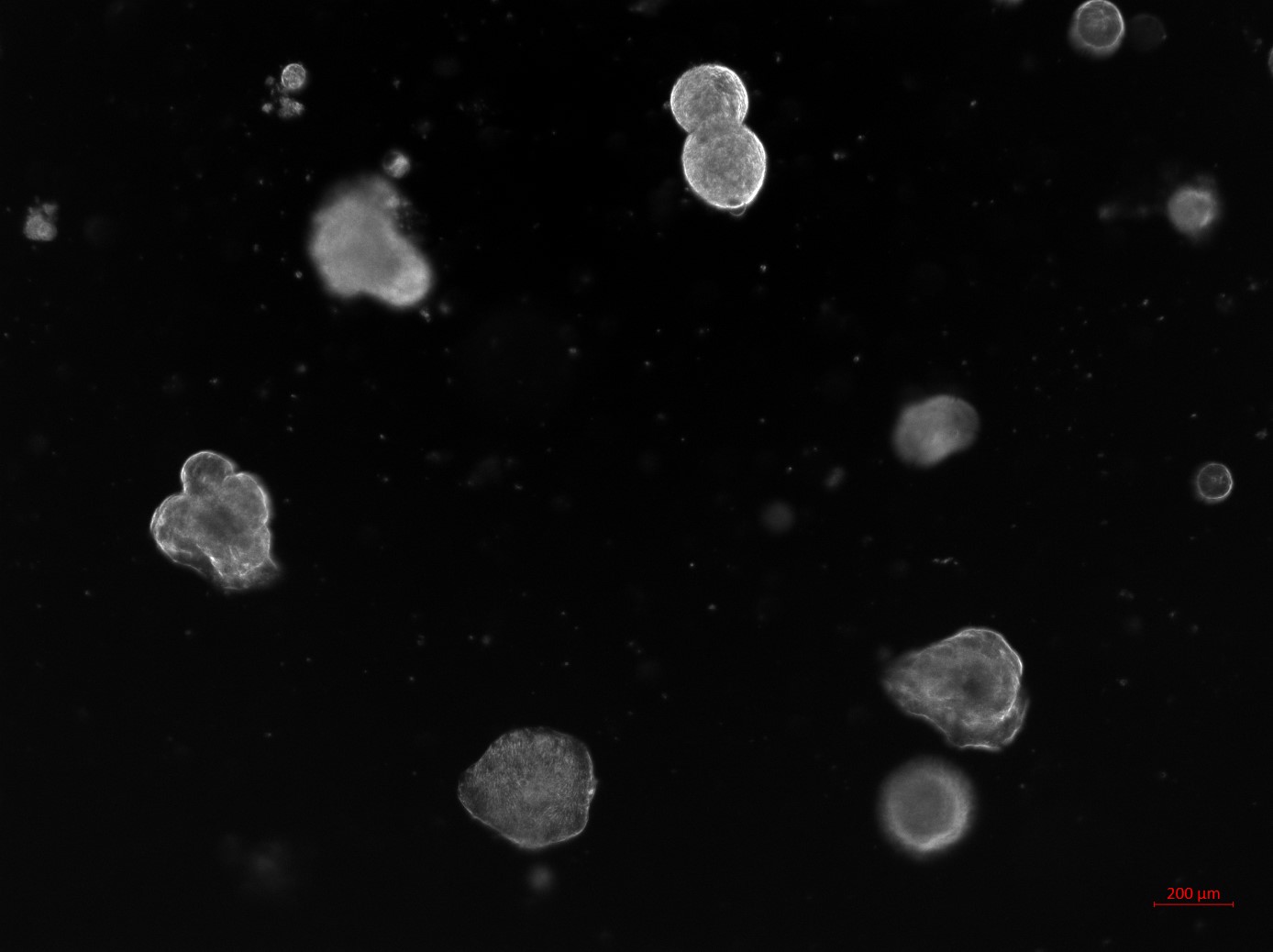

Supplement: Supplementary file 4 — Source Data [file 41467_2024_45605_MOESM4_ESM.zip › Source Data/Figures_Source_Data/supplemental figure 1/panel d,e/E2.jpg]

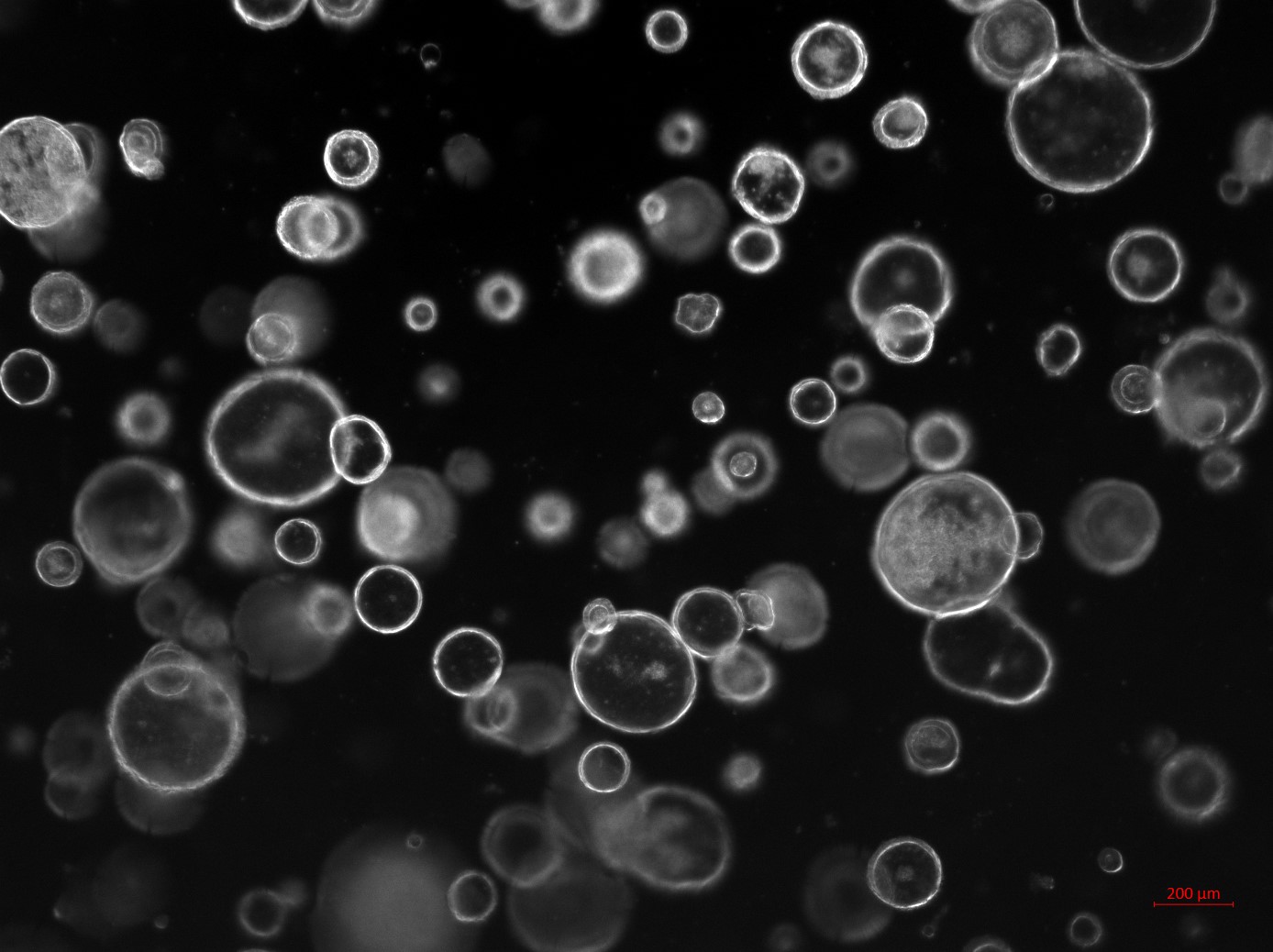

Supplement: Supplementary file 4 — Source Data [file 41467_2024_45605_MOESM4_ESM.zip › Source Data/Figures_Source_Data/supplemental figure 1/panel d,e/E3.jpg]

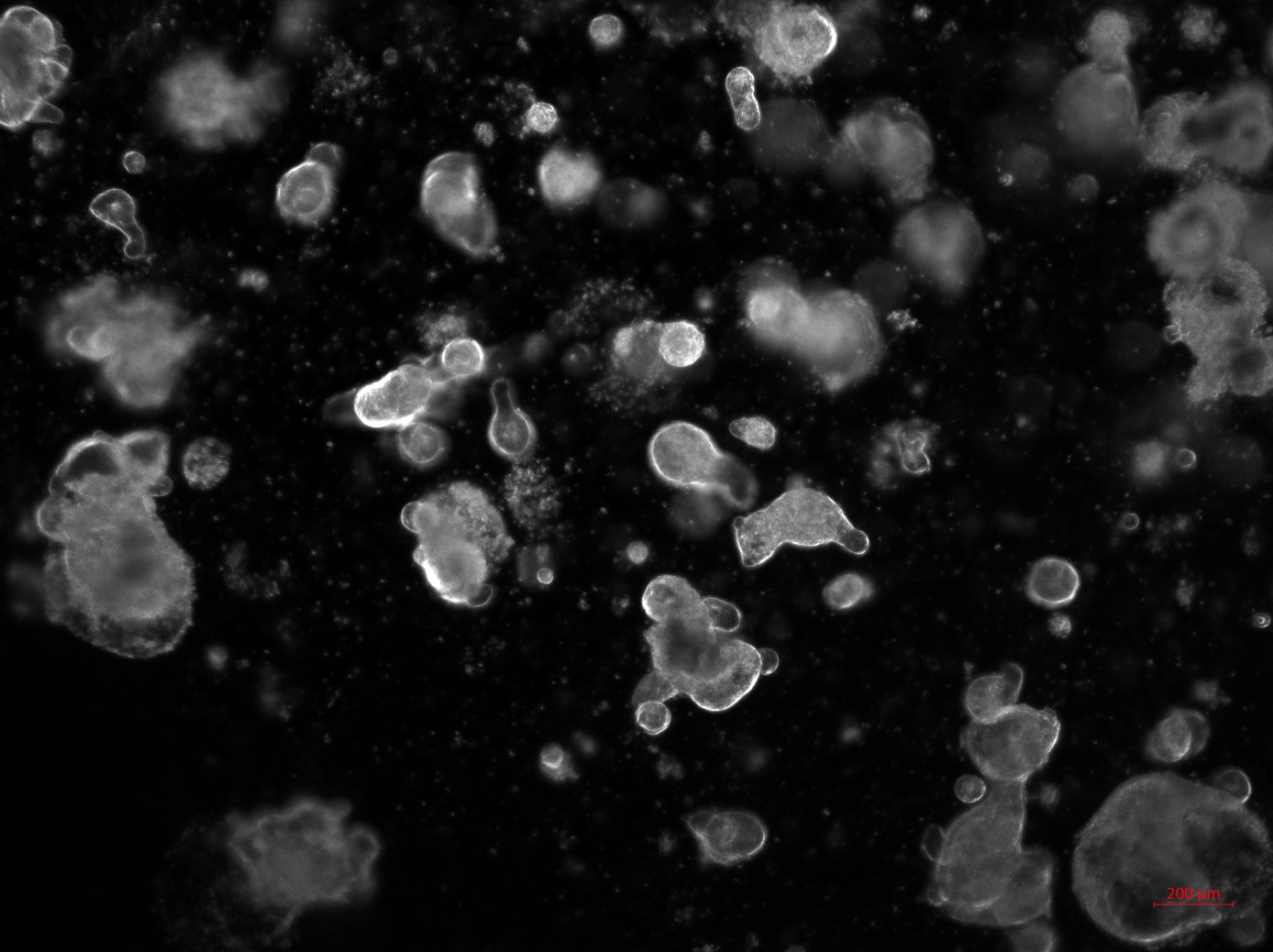

Supplement: Supplementary file 4 — Source Data [file 41467_2024_45605_MOESM4_ESM.zip › Source Data/Figures_Source_Data/supplemental figure 1/panel d,e/E1.jpg]

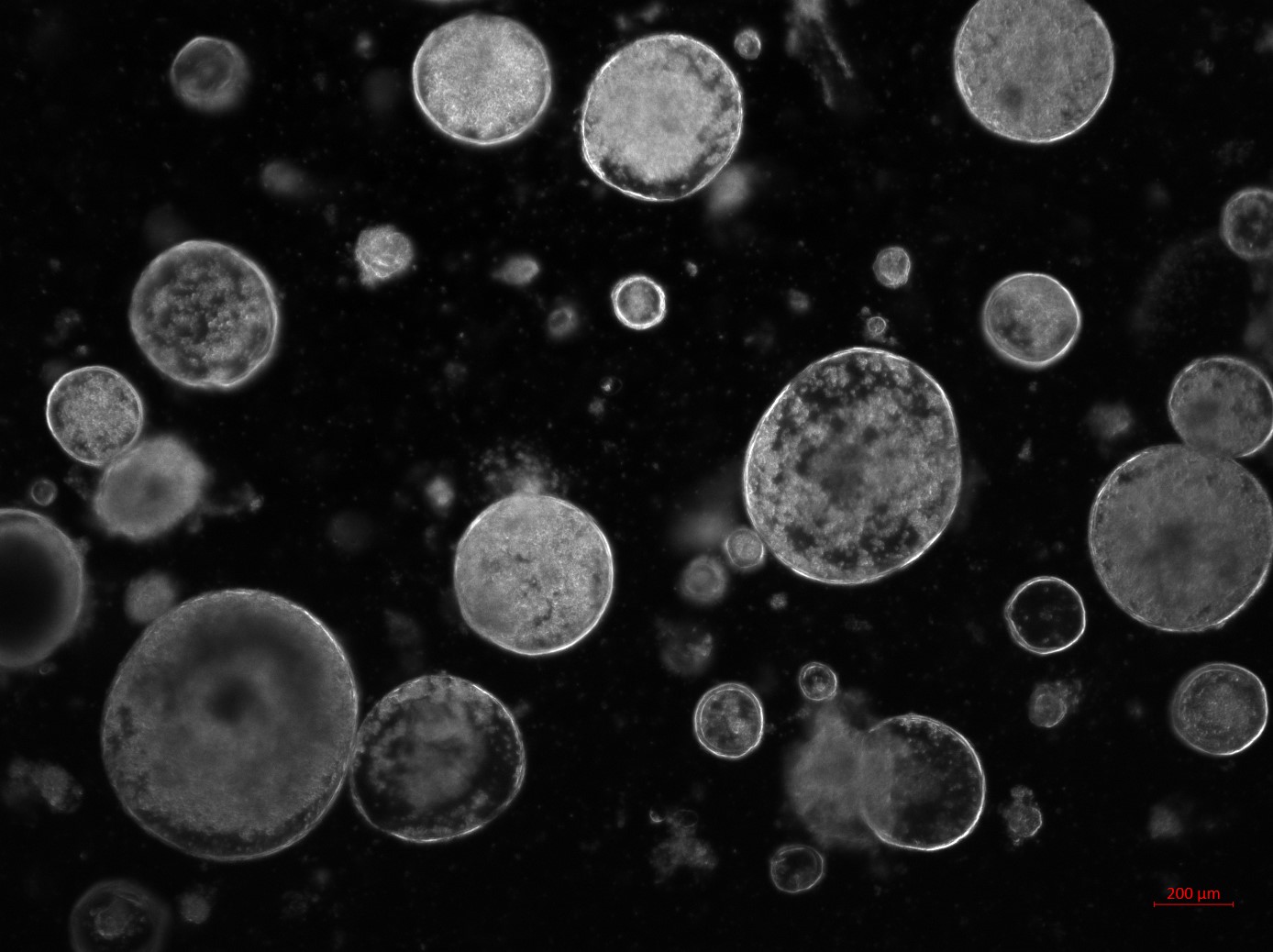

Supplement: Supplementary file 4 — Source Data [file 41467_2024_45605_MOESM4_ESM.zip › Source Data/Figures_Source_Data/supplemental figure 1/panel d,e/D5.jpg]

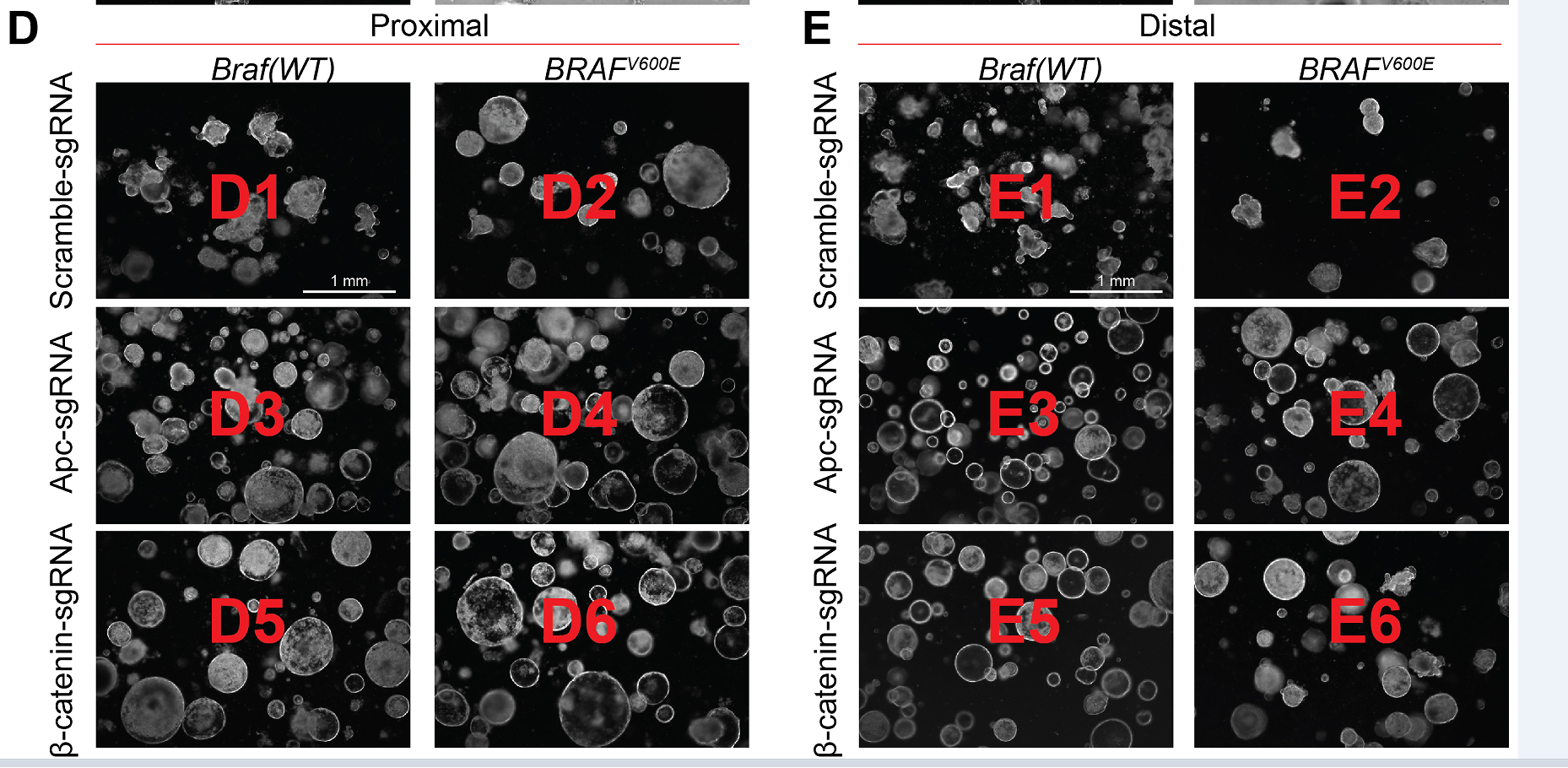

Supplement: Supplementary file 4 — Source Data [file 41467_2024_45605_MOESM4_ESM.zip › Source Data/Figures_Source_Data/supplemental figure 1/panel d,e/panel c,d.png]

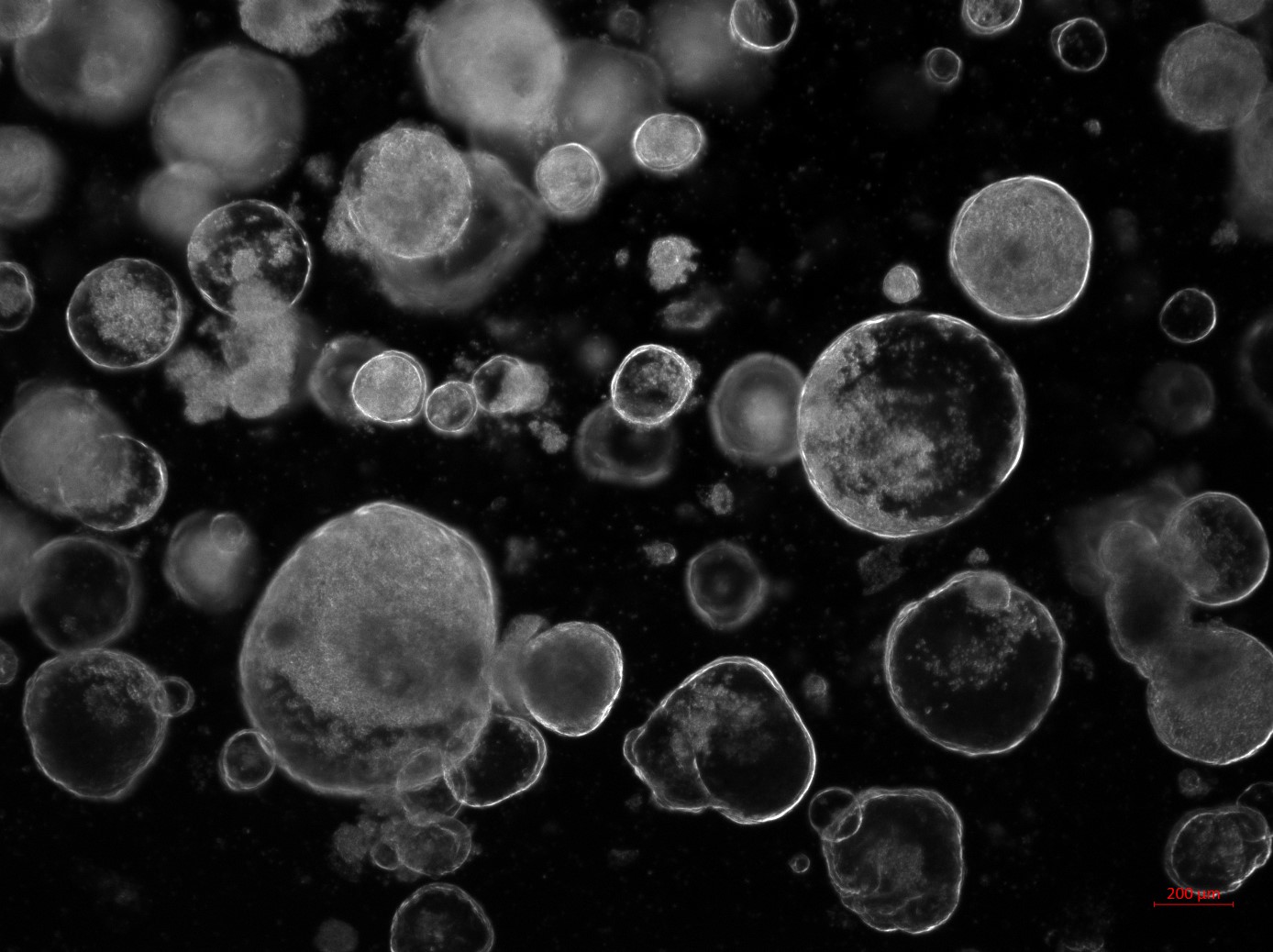

Supplement: Supplementary file 4 — Source Data [file 41467_2024_45605_MOESM4_ESM.zip › Source Data/Figures_Source_Data/supplemental figure 1/panel d,e/D4.jpg]

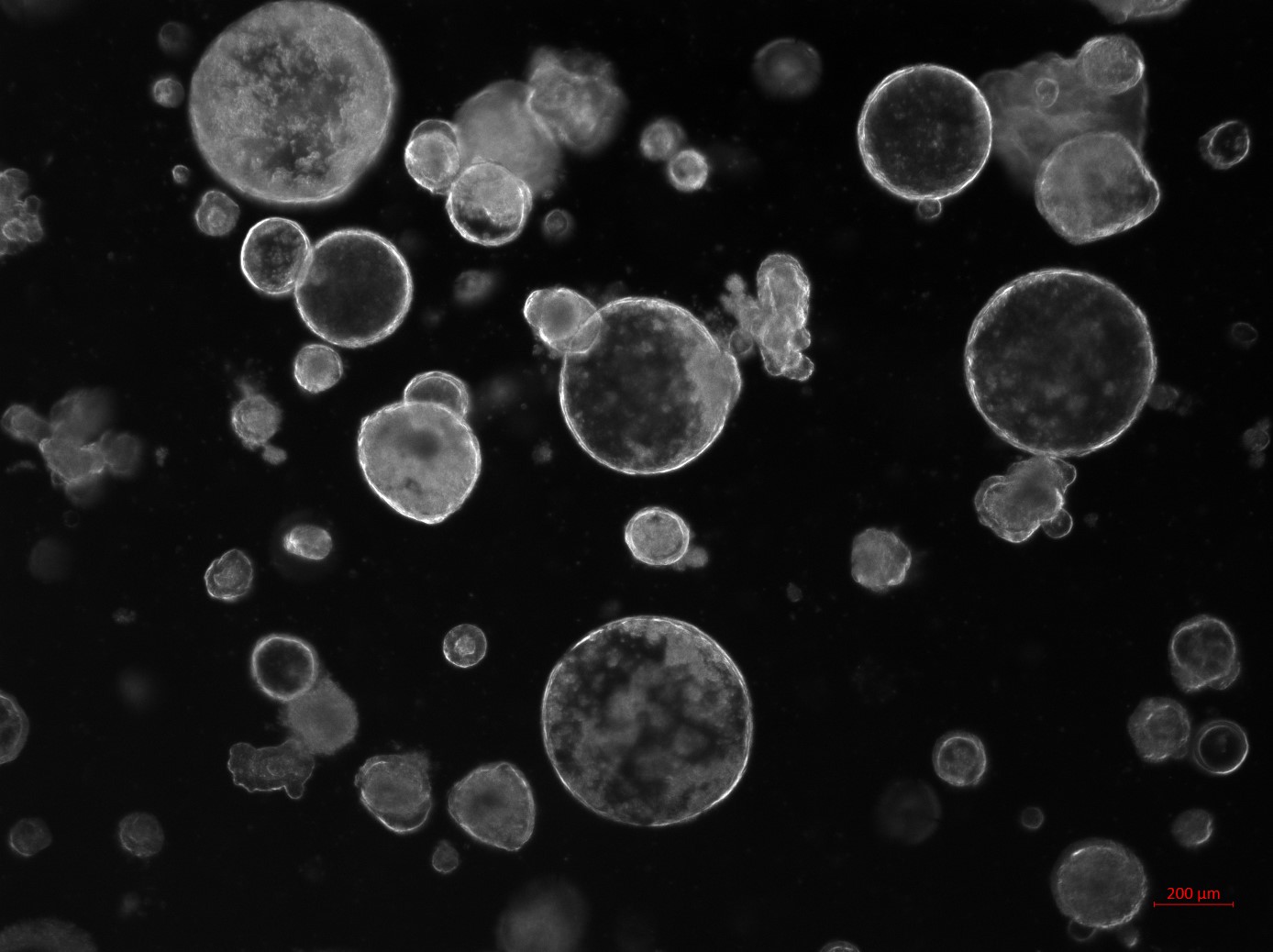

Supplement: Supplementary file 4 — Source Data [file 41467_2024_45605_MOESM4_ESM.zip › Source Data/Figures_Source_Data/supplemental figure 1/panel d,e/E4.jpg]

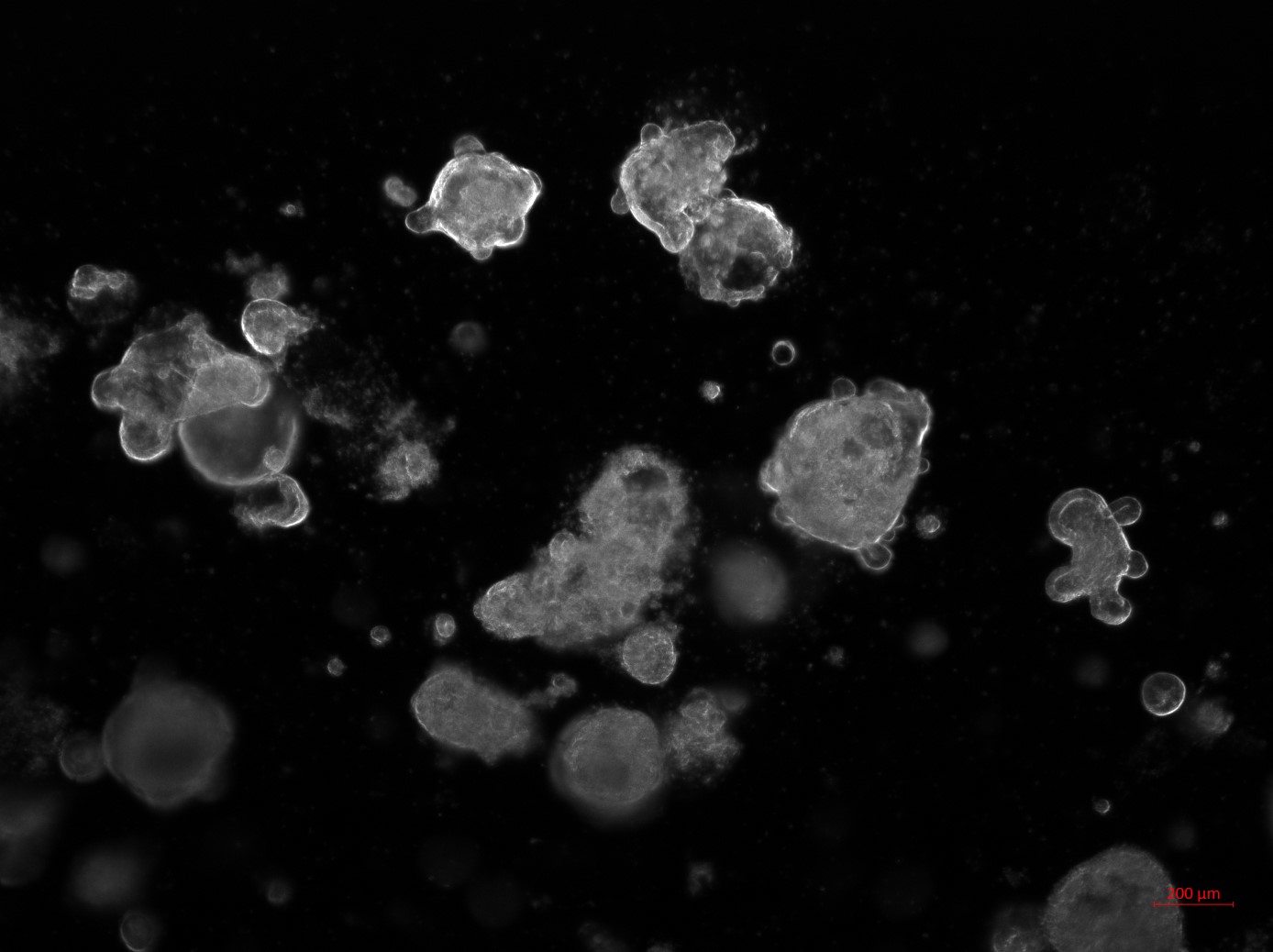

Supplement: Supplementary file 4 — Source Data [file 41467_2024_45605_MOESM4_ESM.zip › Source Data/Figures_Source_Data/supplemental figure 1/panel d,e/D1.jpg]

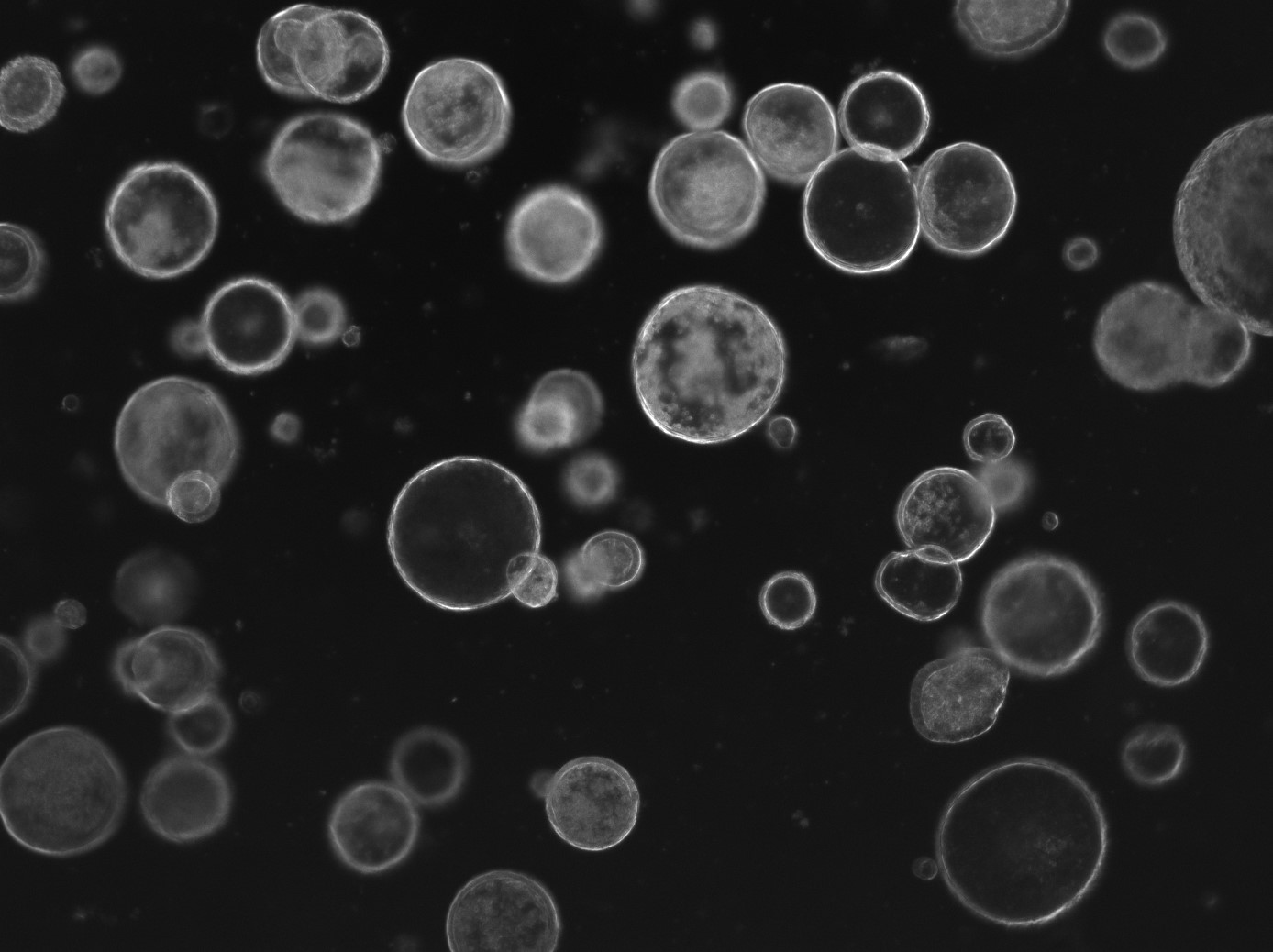

Supplement: Supplementary file 4 — Source Data [file 41467_2024_45605_MOESM4_ESM.zip › Source Data/Figures_Source_Data/supplemental figure 1/panel d,e/E5.jpg]

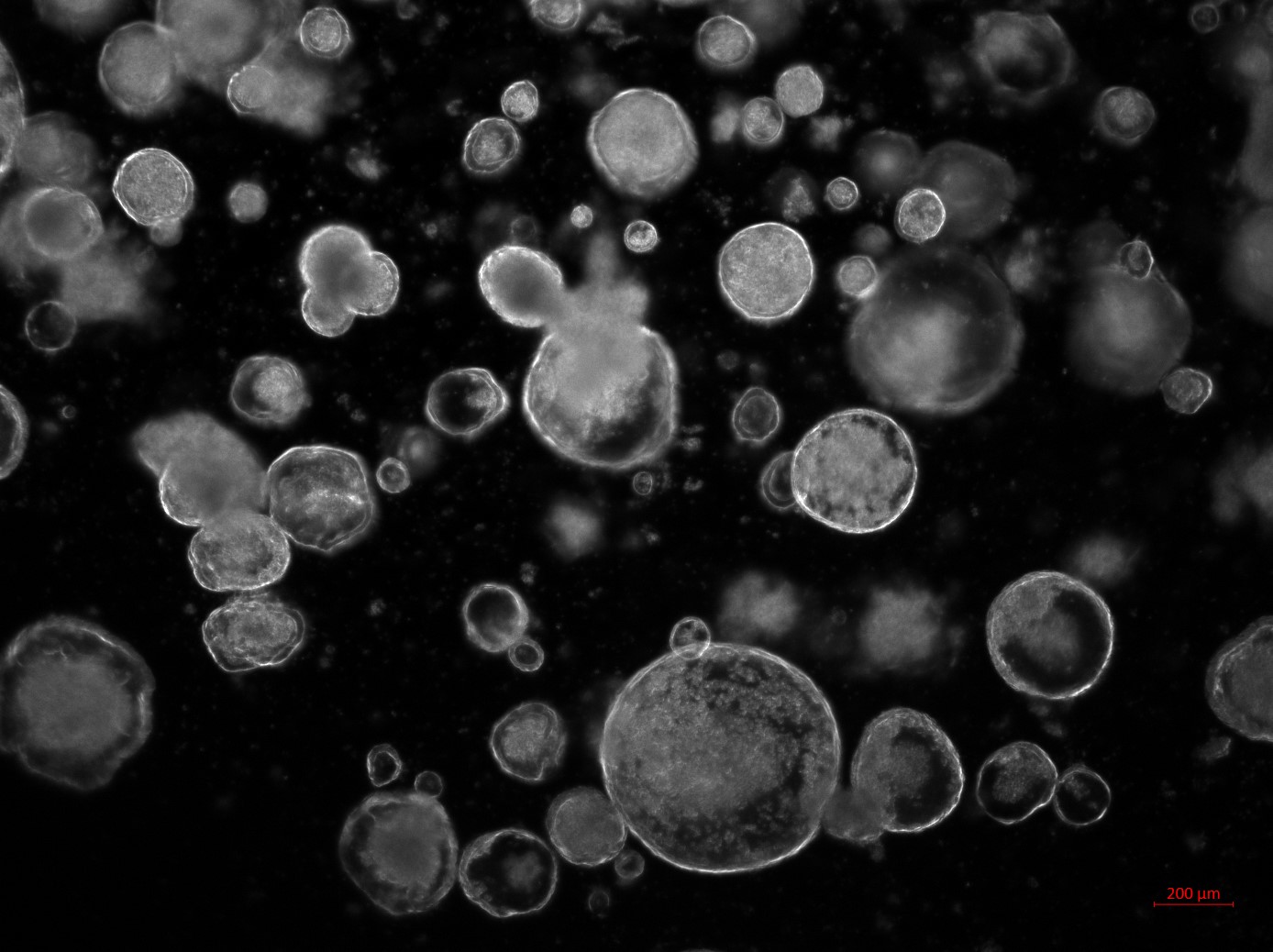

Supplement: Supplementary file 4 — Source Data [file 41467_2024_45605_MOESM4_ESM.zip › Source Data/Figures_Source_Data/supplemental figure 1/panel d,e/D3.jpg]
