# Supplementary figures and images for: Tissue-location-specific transcription programs drive tumor dependencies in colon cancer (part 1 of 2)
Source: Nat Commun. 2024 Feb 15;15:1384. doi: 10.1038/s41467-024-45605-4 (PMC10869357; doi:10.1038/s41467-024-45605-4)

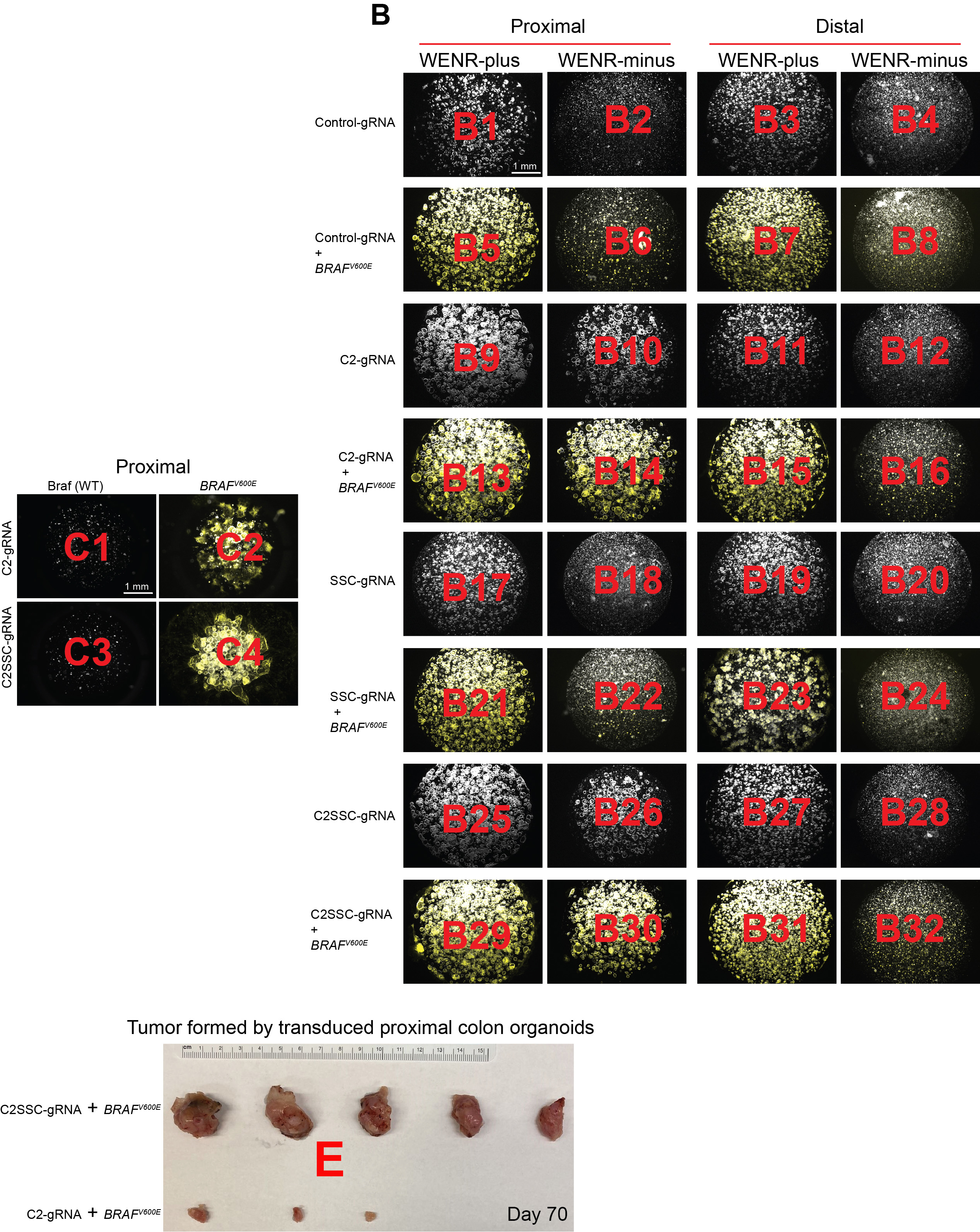

Supplement: Supplementary file 4 — Source Data [file 41467_2024_45605_MOESM4_ESM.zip › Source Data/Figures_Source_Data/figure 3/figure 3.jpg]

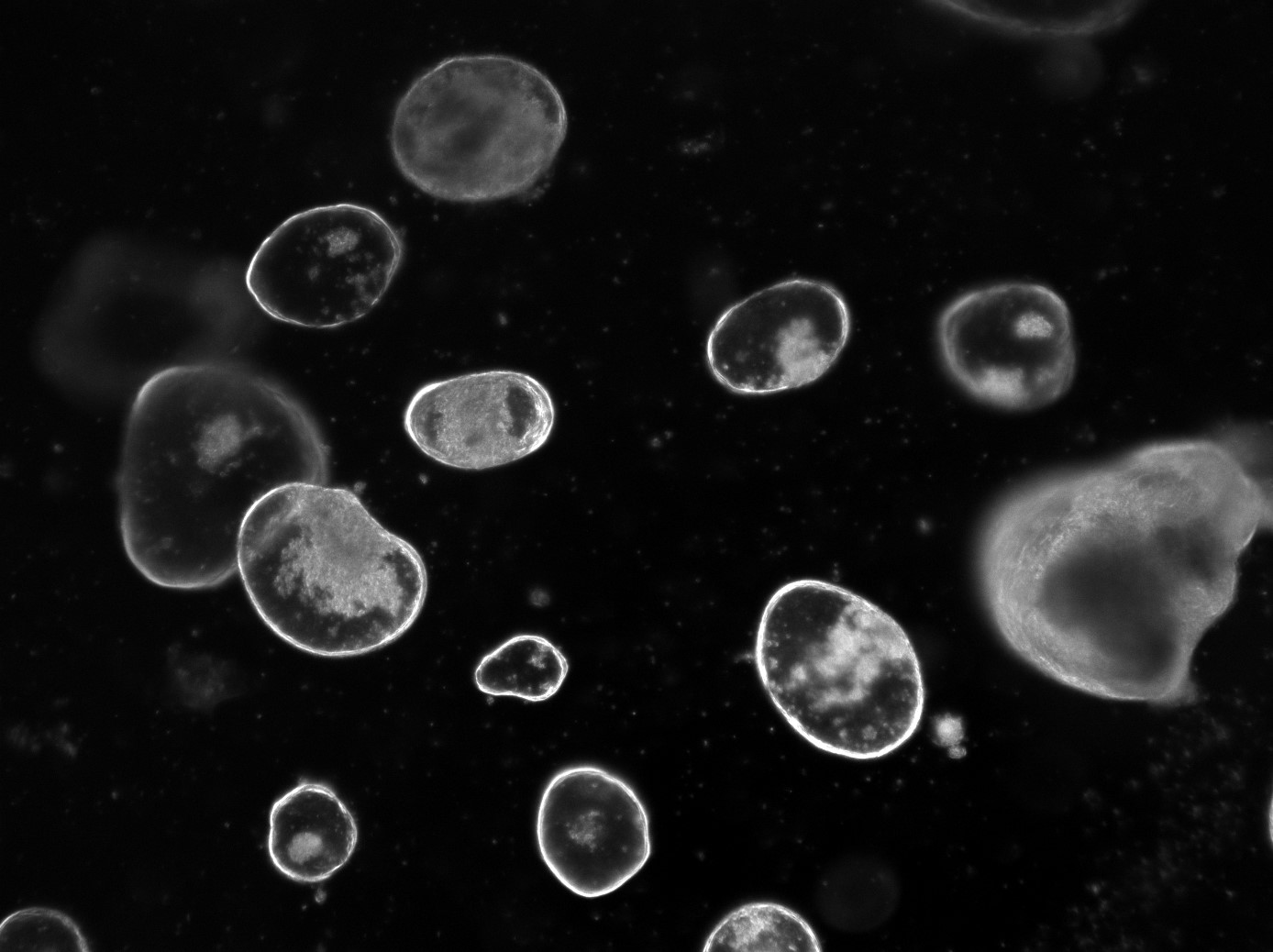

Supplement: Supplementary file 4 — Source Data [file 41467_2024_45605_MOESM4_ESM.zip › Source Data/Figures_Source_Data/supplemental figure 3/panel e/e2.jpg]

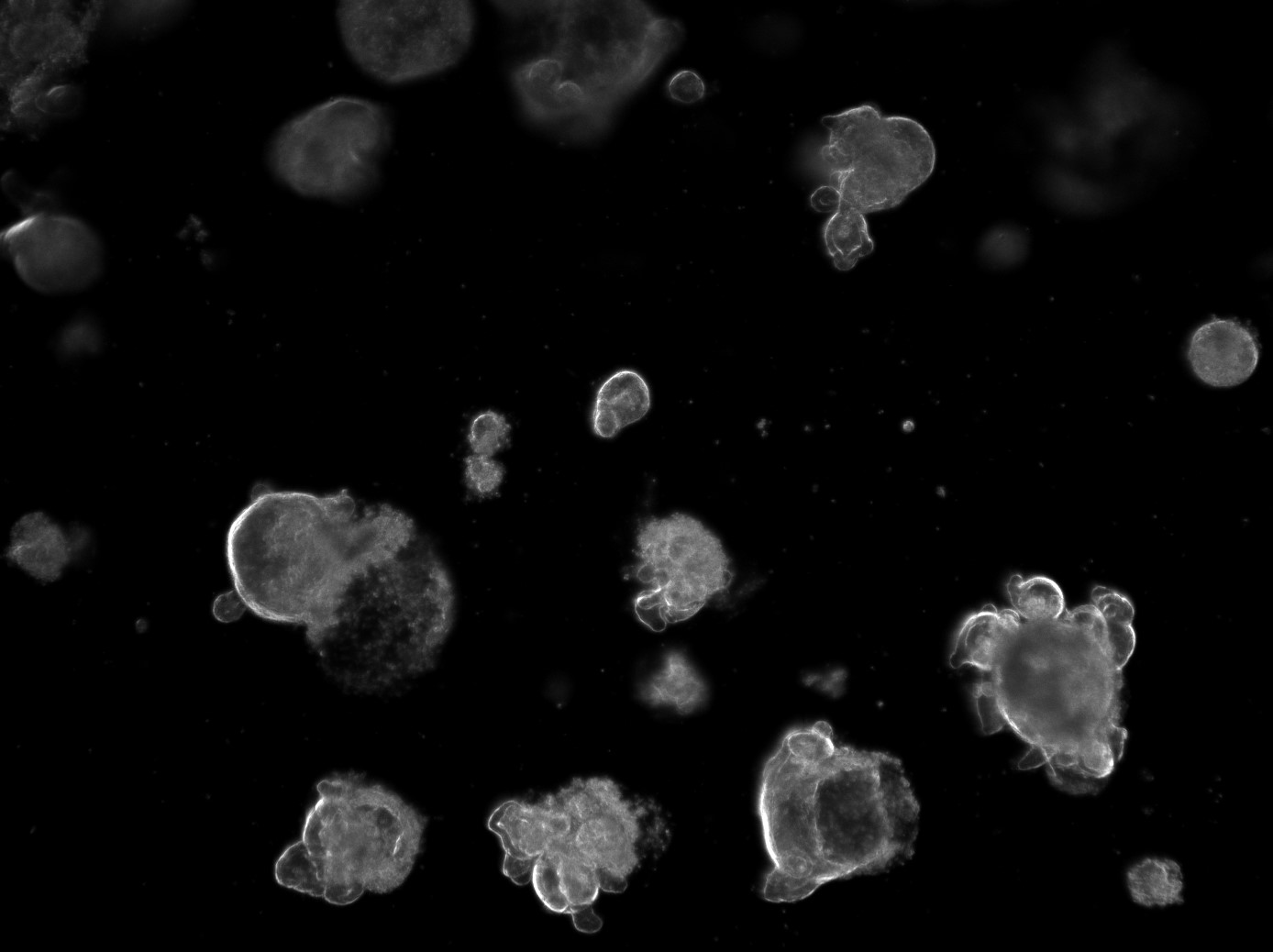

Supplement: Supplementary file 4 — Source Data [file 41467_2024_45605_MOESM4_ESM.zip › Source Data/Figures_Source_Data/supplemental figure 3/panel e/e4.jpg]

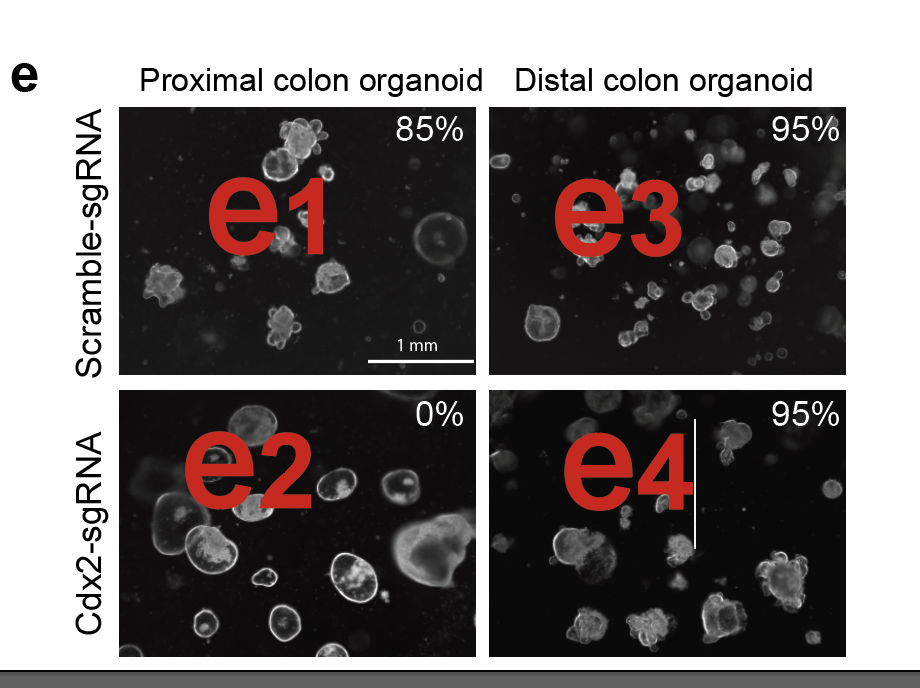

Supplement: Supplementary file 4 — Source Data [file 41467_2024_45605_MOESM4_ESM.zip › Source Data/Figures_Source_Data/supplemental figure 3/panel e/panel e.png]

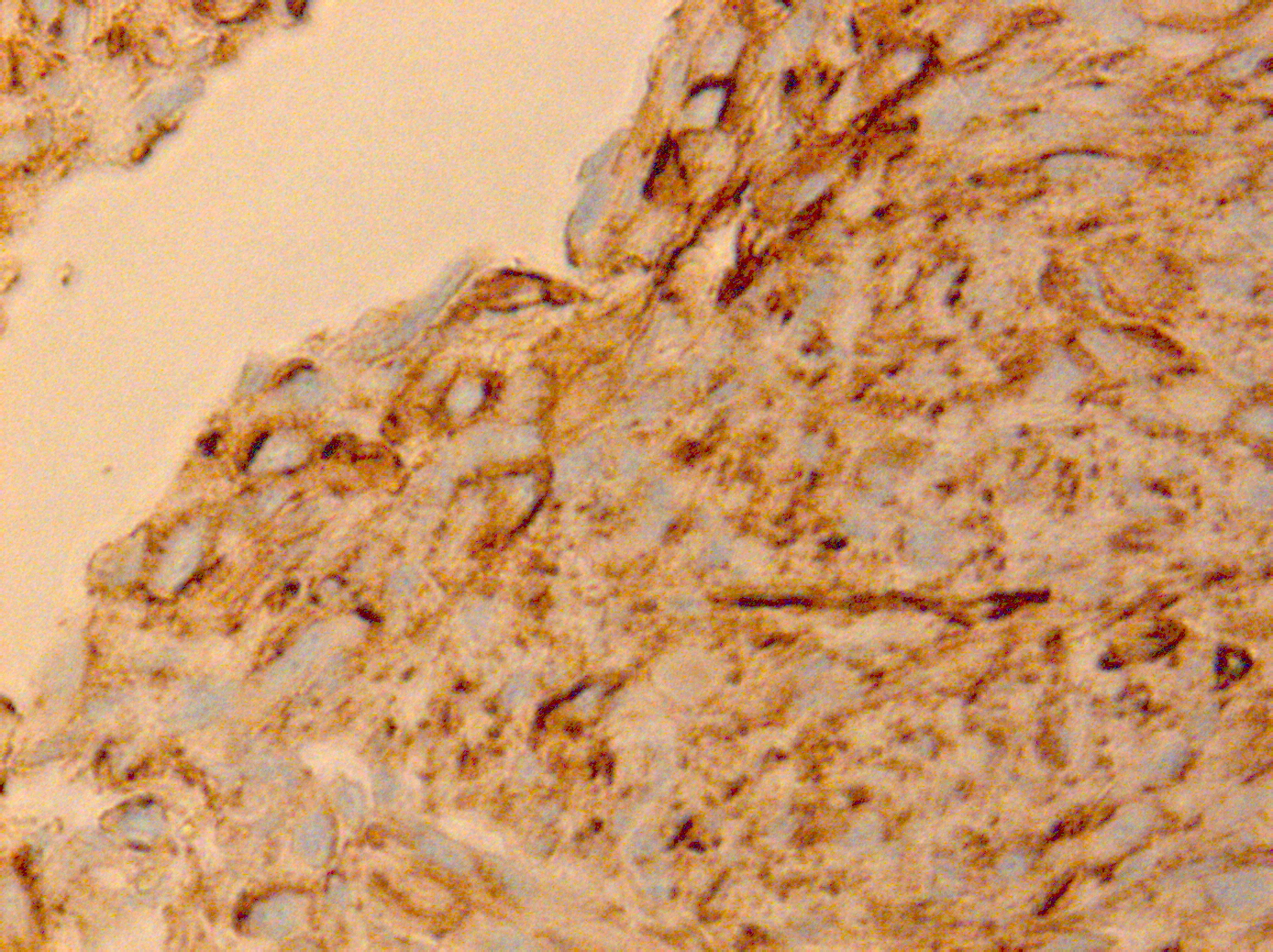

Supplement: Supplementary file 4 — Source Data [file 41467_2024_45605_MOESM4_ESM.zip › Source Data/Figures_Source_Data/supplemental figure 4/Panel d,e,f/F6..jpg]

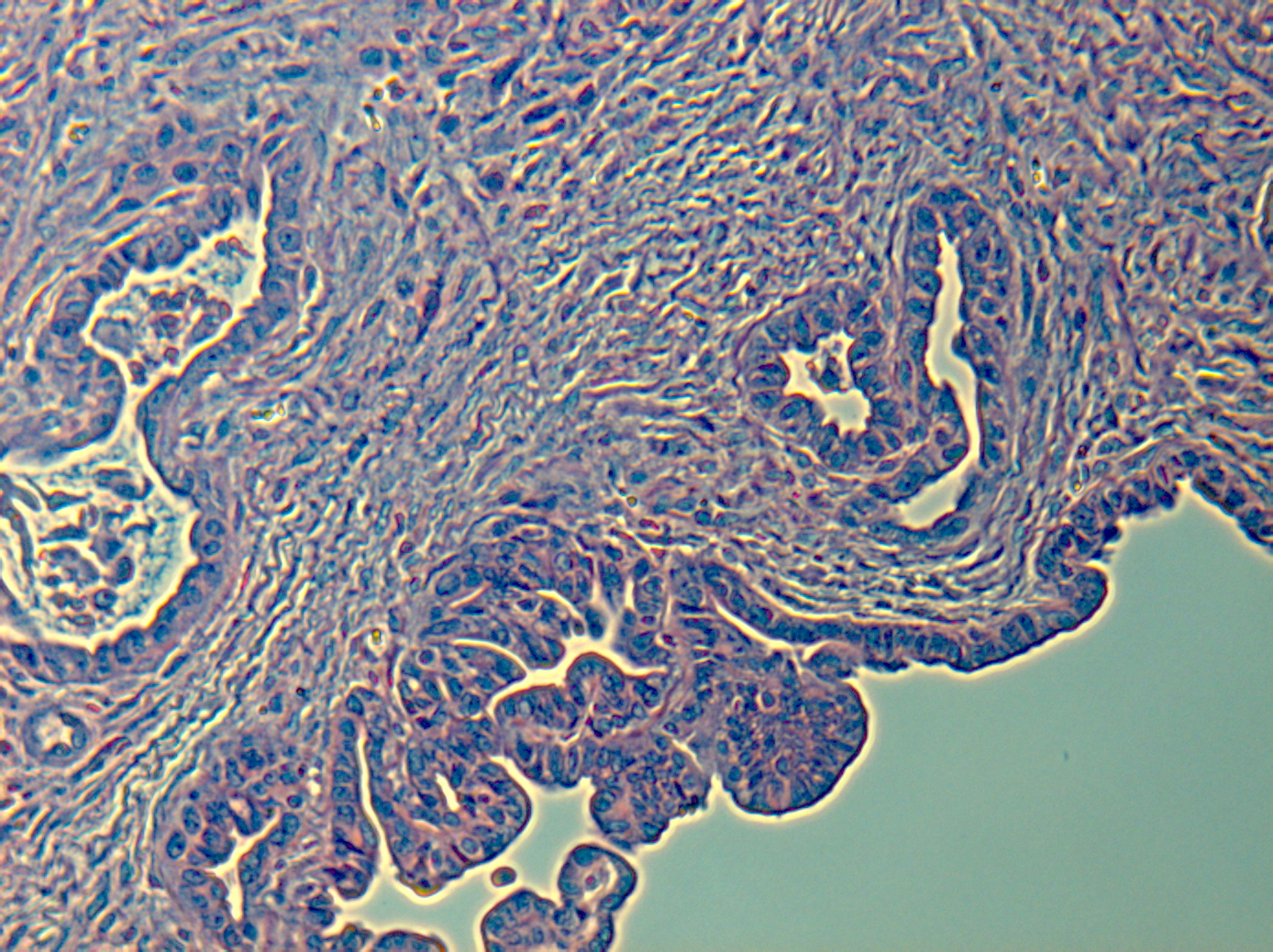

Supplement: Supplementary file 4 — Source Data [file 41467_2024_45605_MOESM4_ESM.zip › Source Data/Figures_Source_Data/supplemental figure 4/Panel d,e,f/F7..jpg]

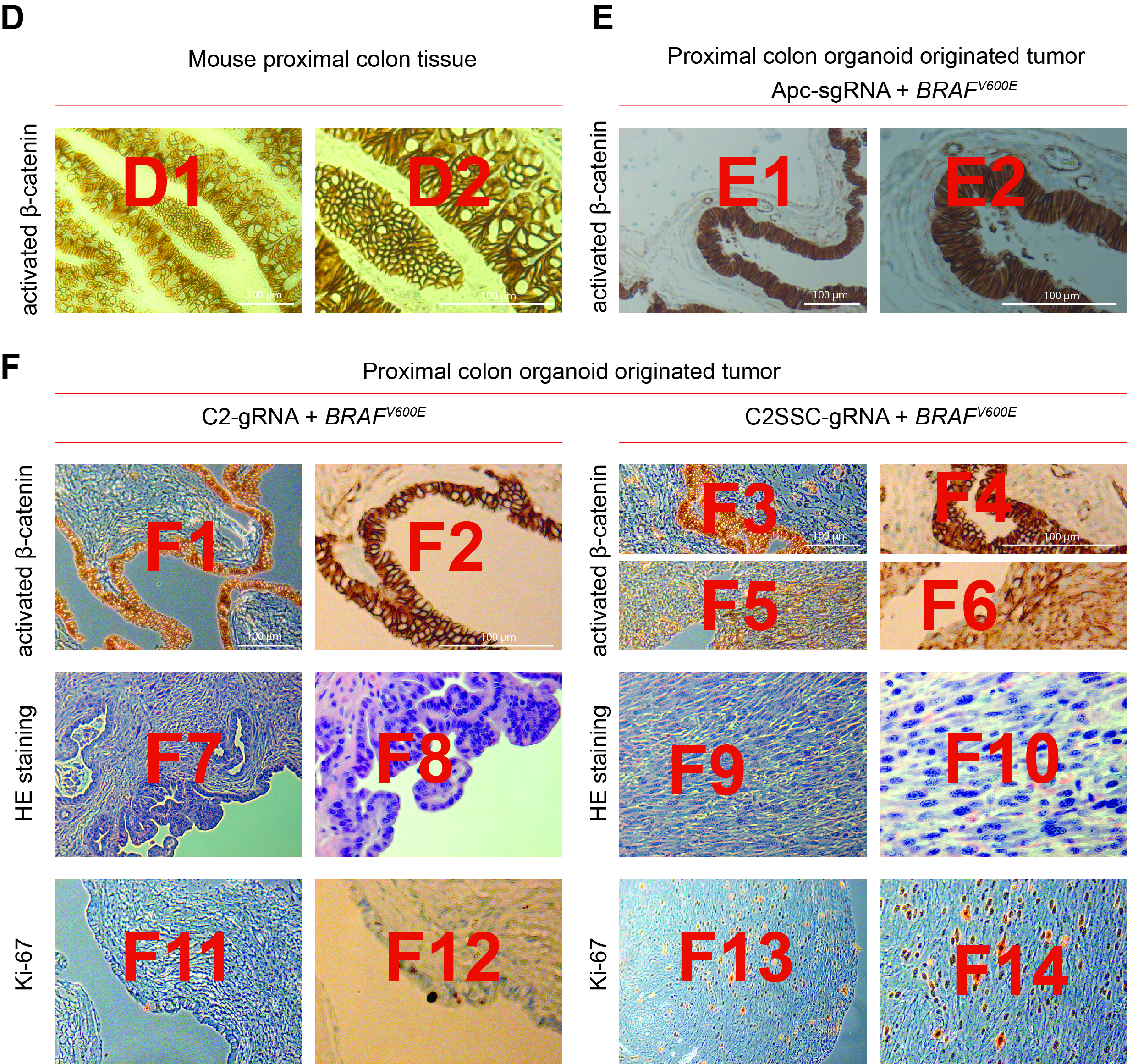

Supplement: Supplementary file 4 — Source Data [file 41467_2024_45605_MOESM4_ESM.zip › Source Data/Figures_Source_Data/supplemental figure 4/Panel d,e,f/sup_figure_4-2_20221028.jpg]

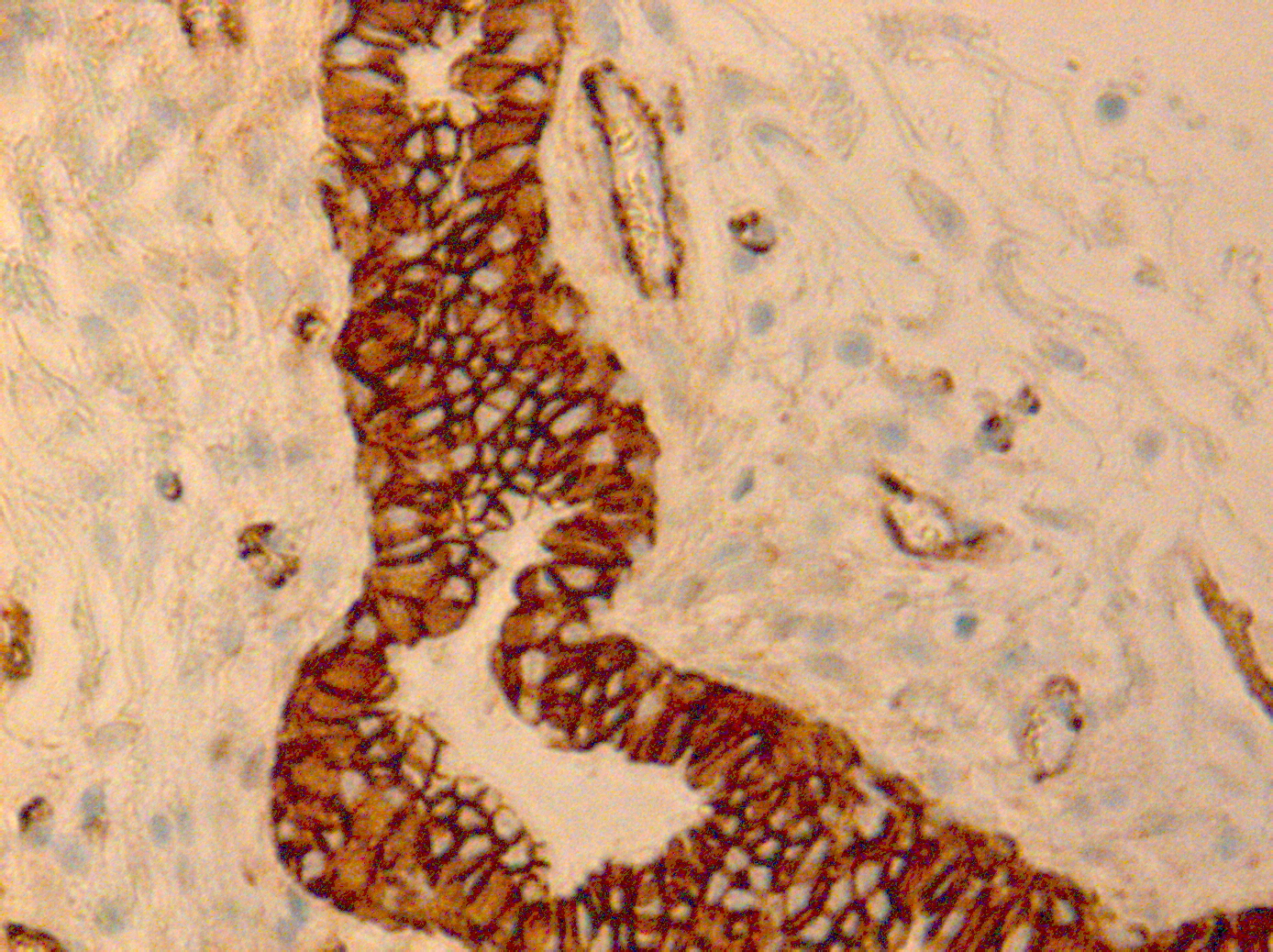

Supplement: Supplementary file 4 — Source Data [file 41467_2024_45605_MOESM4_ESM.zip › Source Data/Figures_Source_Data/supplemental figure 4/Panel d,e,f/F4.jpg]

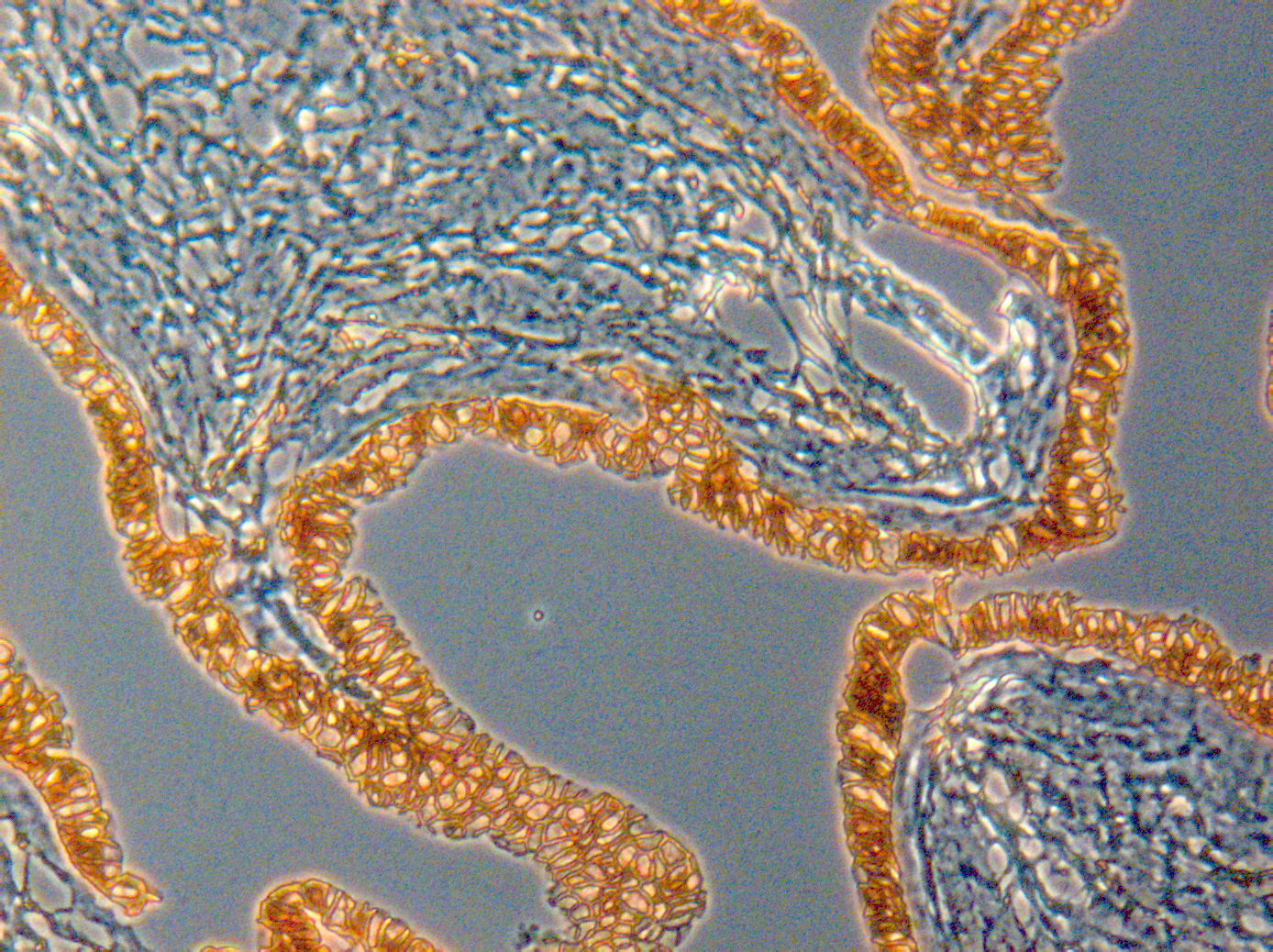

Supplement: Supplementary file 4 — Source Data [file 41467_2024_45605_MOESM4_ESM.zip › Source Data/Figures_Source_Data/supplemental figure 4/Panel d,e,f/F1.jpg]

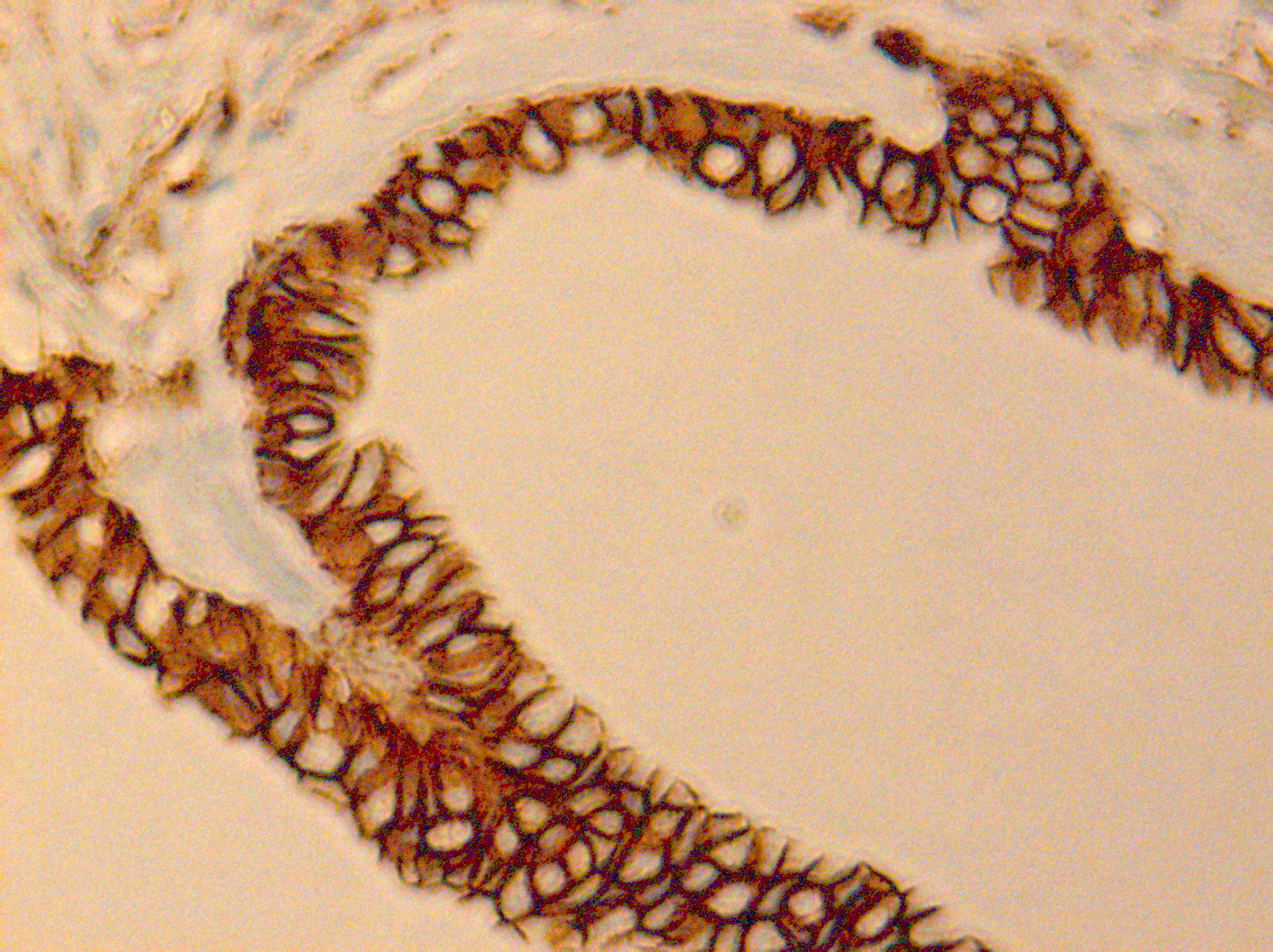

Supplement: Supplementary file 4 — Source Data [file 41467_2024_45605_MOESM4_ESM.zip › Source Data/Figures_Source_Data/supplemental figure 4/Panel d,e,f/F2.jpg]

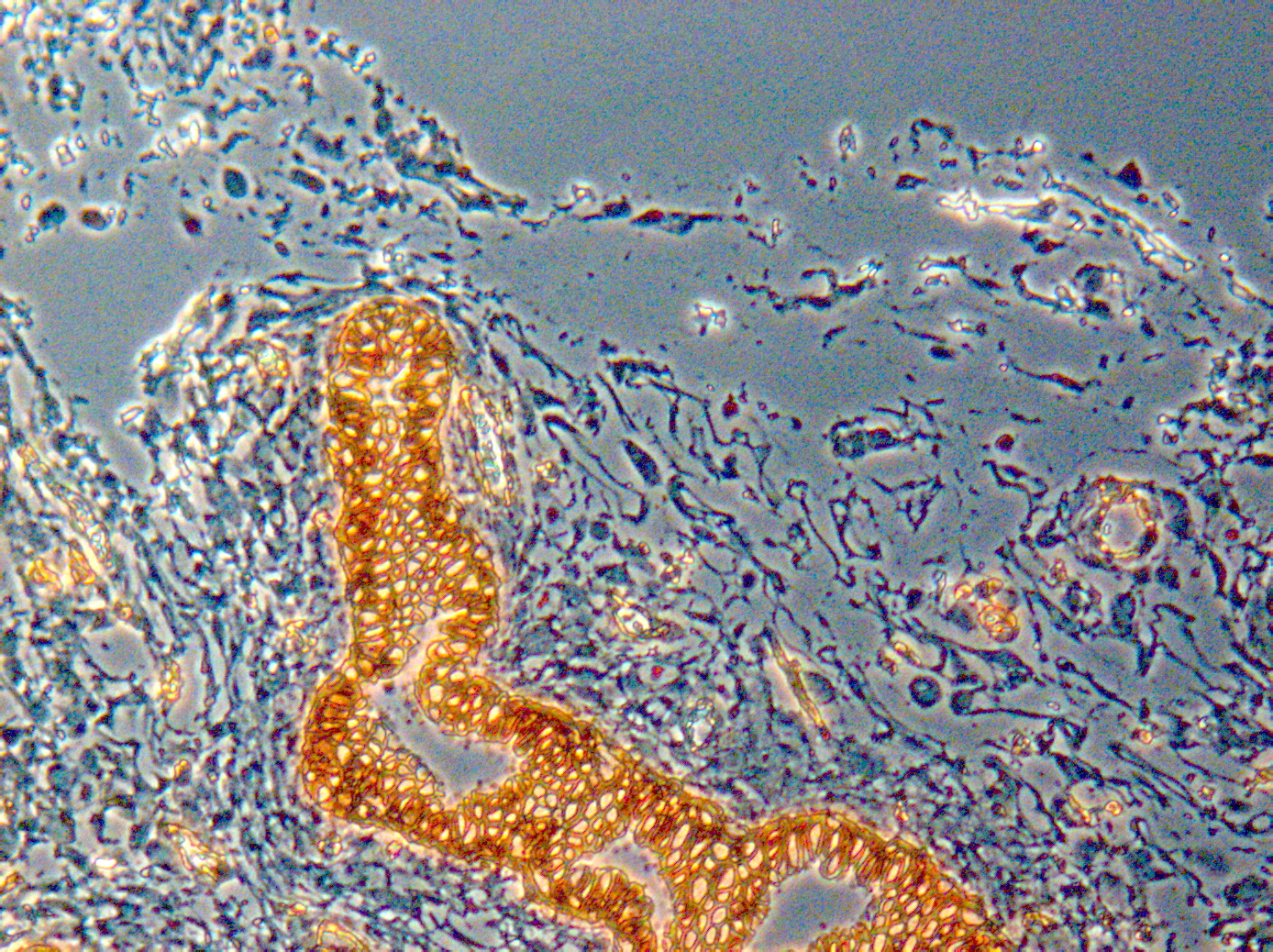

Supplement: Supplementary file 4 — Source Data [file 41467_2024_45605_MOESM4_ESM.zip › Source Data/Figures_Source_Data/supplemental figure 4/Panel d,e,f/F3.jpg]

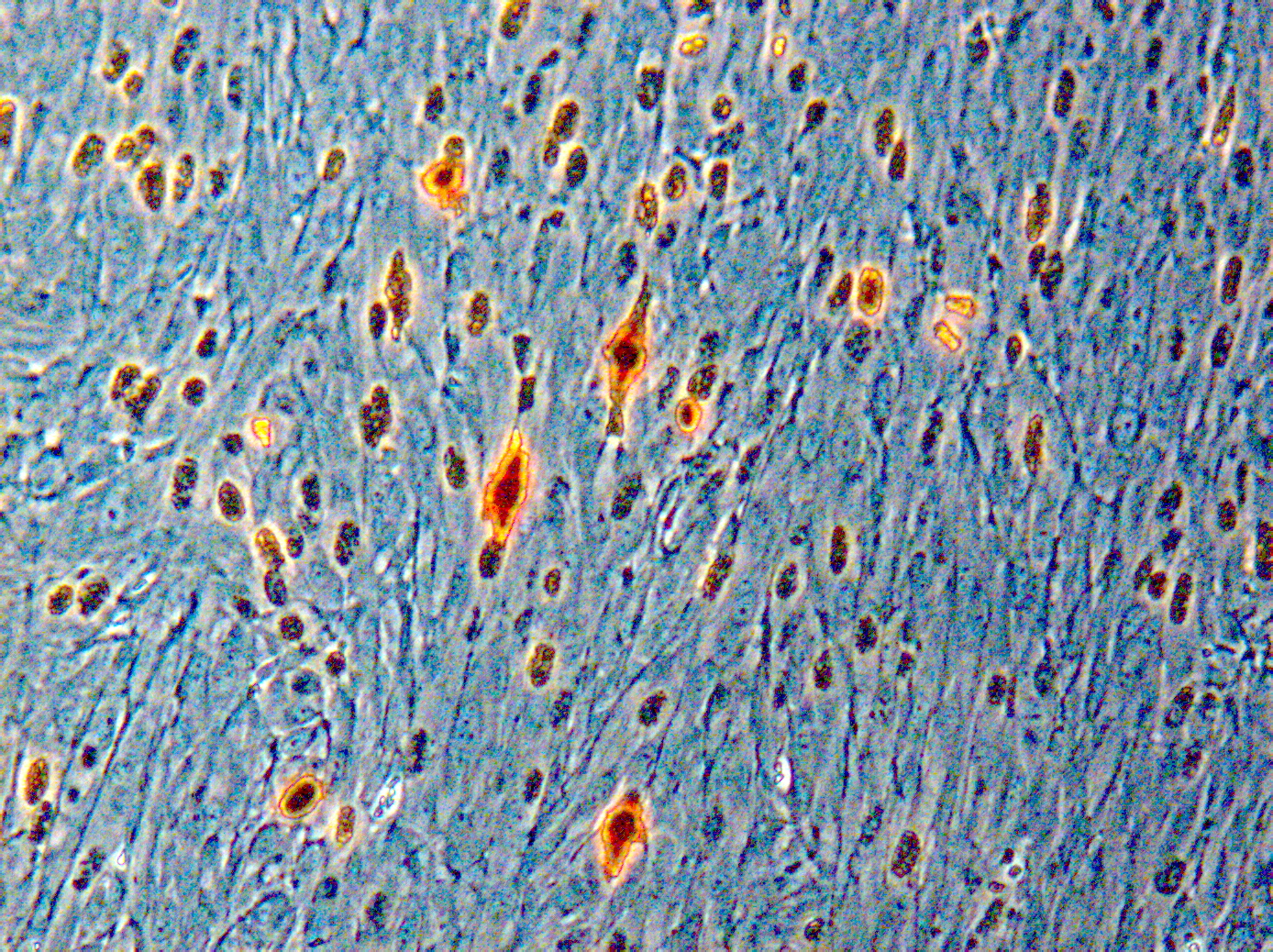

Supplement: Supplementary file 4 — Source Data [file 41467_2024_45605_MOESM4_ESM.zip › Source Data/Figures_Source_Data/supplemental figure 4/Panel d,e,f/F14.jpg]

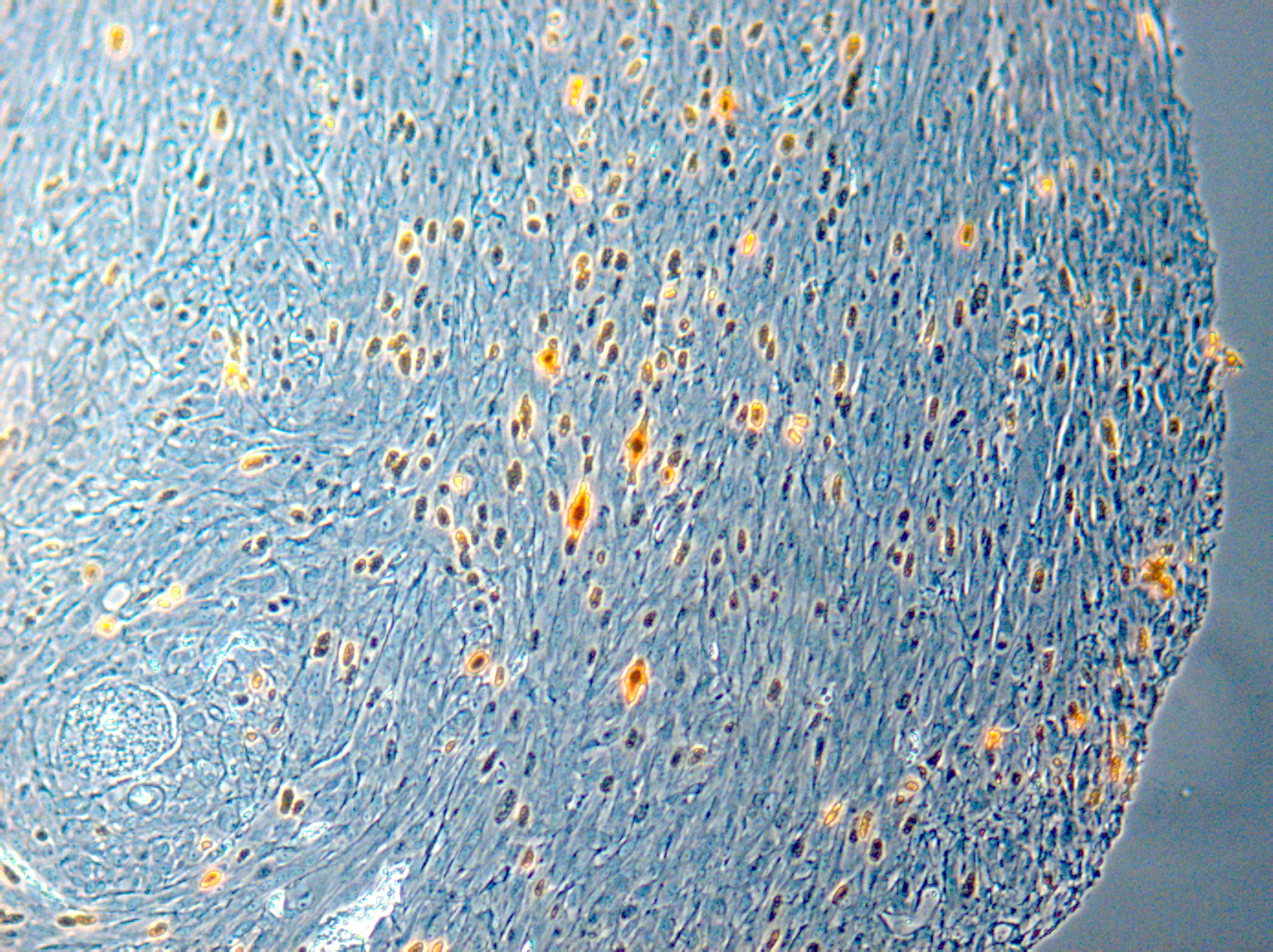

Supplement: Supplementary file 4 — Source Data [file 41467_2024_45605_MOESM4_ESM.zip › Source Data/Figures_Source_Data/supplemental figure 4/Panel d,e,f/F13.jpg]

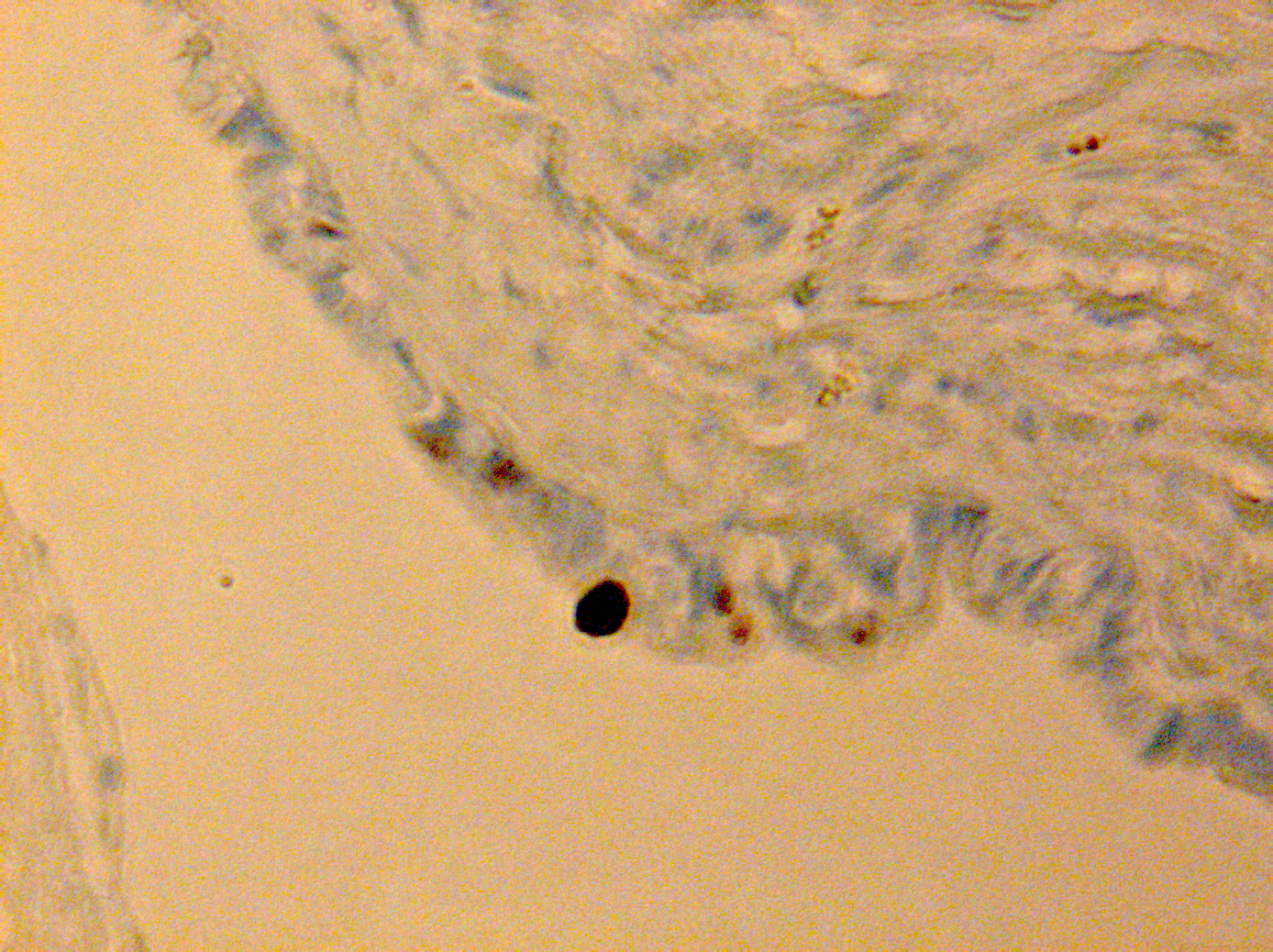

Supplement: Supplementary file 4 — Source Data [file 41467_2024_45605_MOESM4_ESM.zip › Source Data/Figures_Source_Data/supplemental figure 4/Panel d,e,f/F12.jpg]

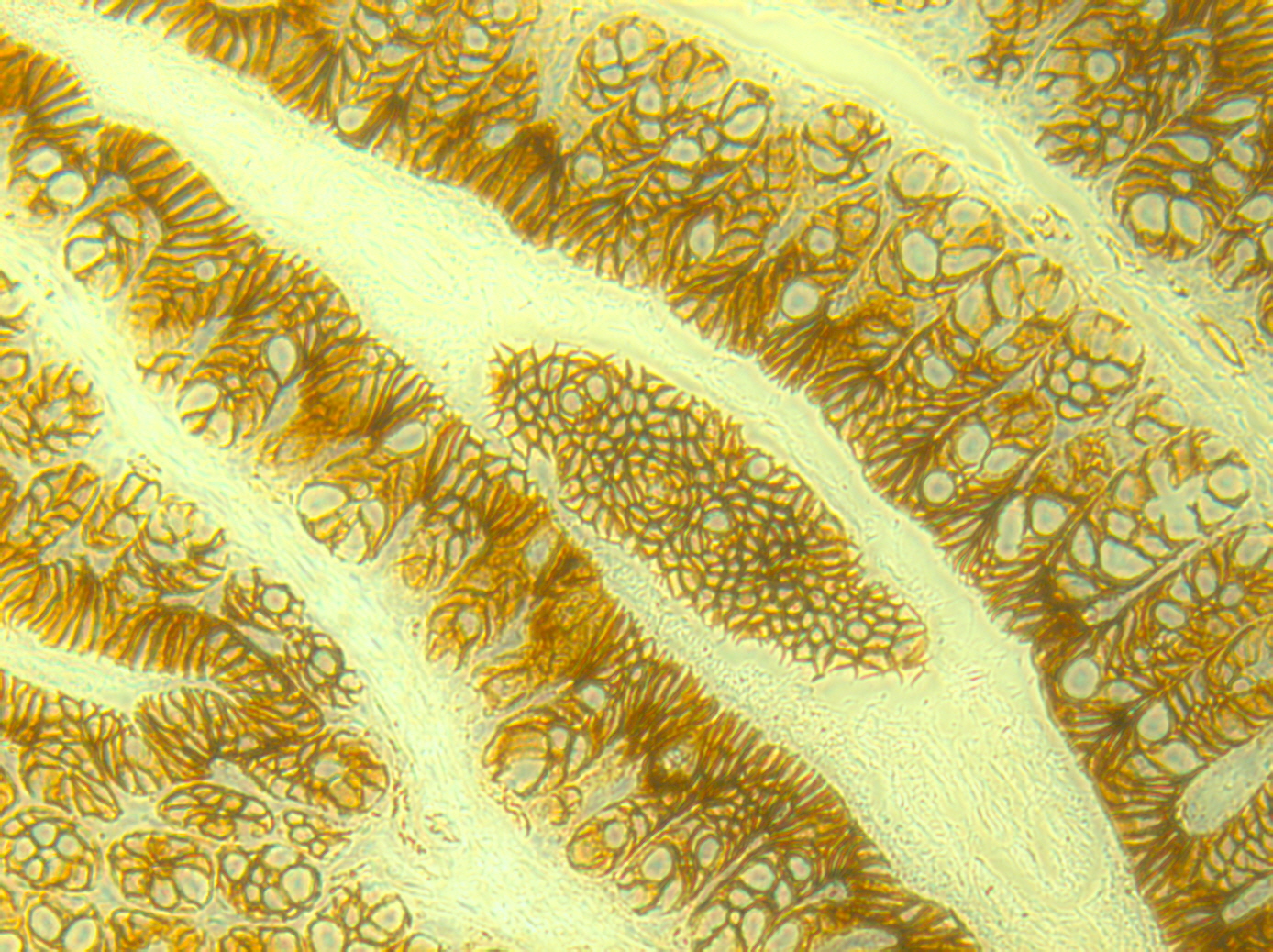

Supplement: Supplementary file 4 — Source Data [file 41467_2024_45605_MOESM4_ESM.zip › Source Data/Figures_Source_Data/supplemental figure 4/Panel d,e,f/D1.jpg]

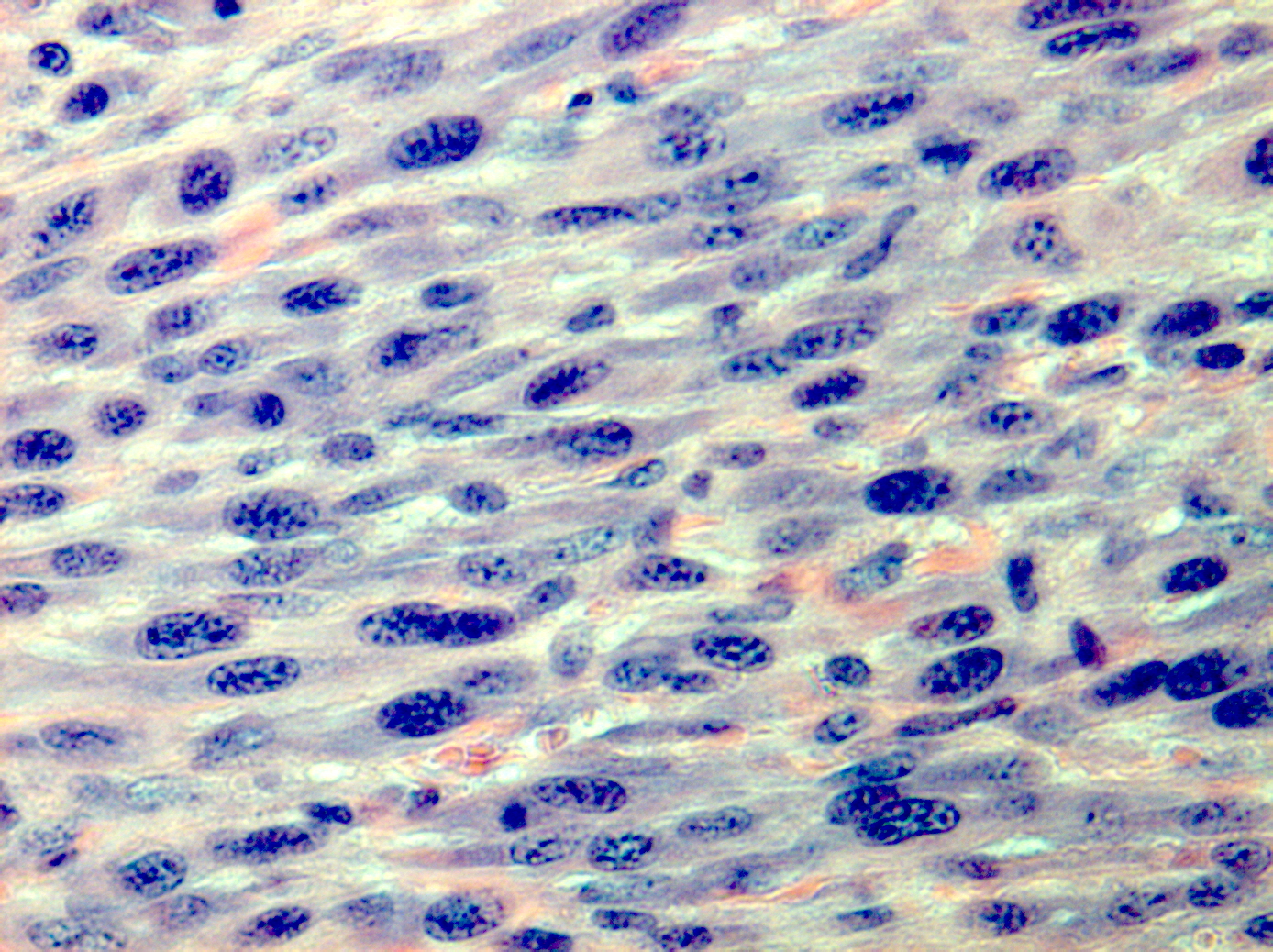

Supplement: Supplementary file 4 — Source Data [file 41467_2024_45605_MOESM4_ESM.zip › Source Data/Figures_Source_Data/supplemental figure 4/Panel d,e,f/F10.jpg]

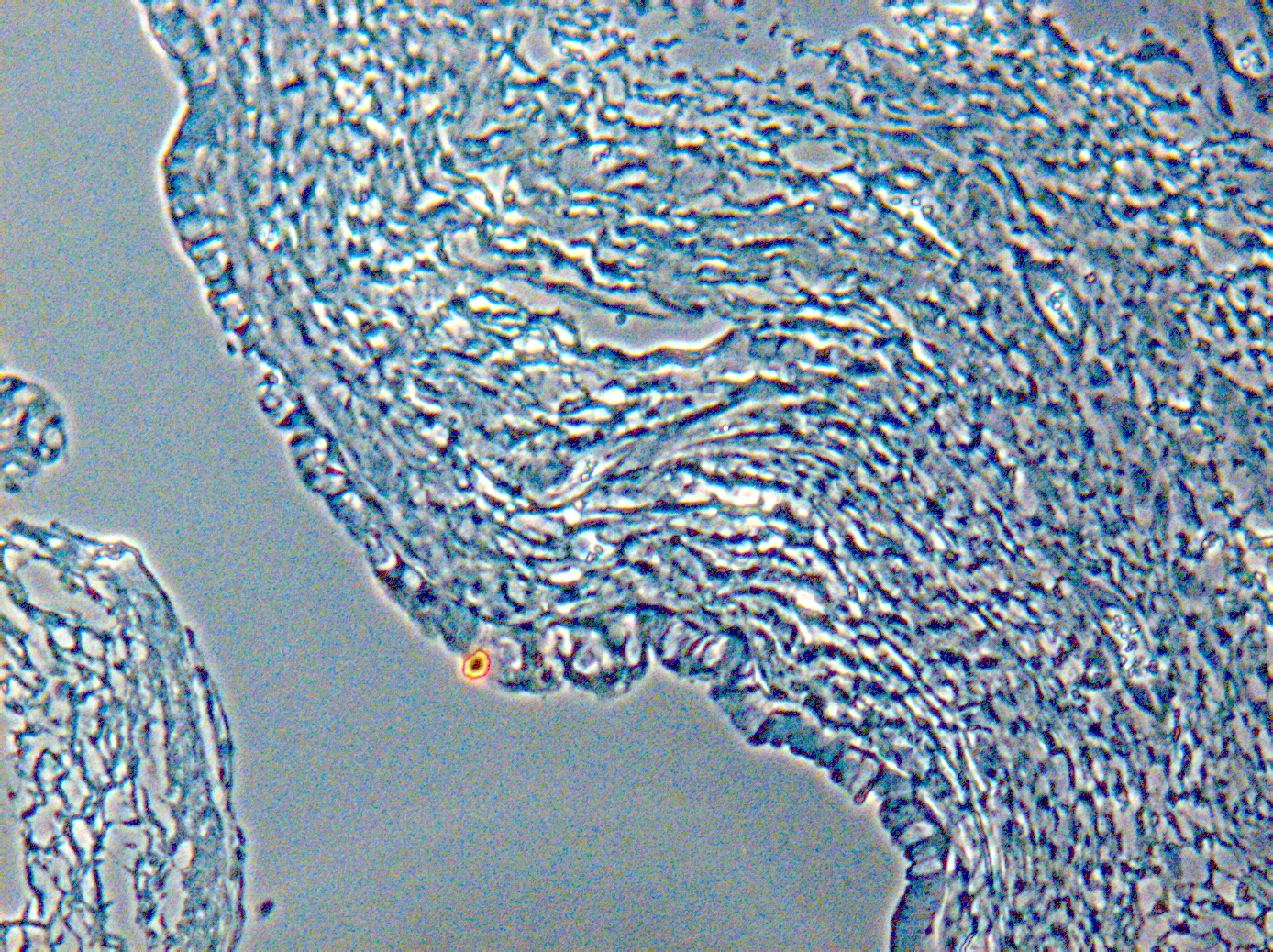

Supplement: Supplementary file 4 — Source Data [file 41467_2024_45605_MOESM4_ESM.zip › Source Data/Figures_Source_Data/supplemental figure 4/Panel d,e,f/F11.jpg]

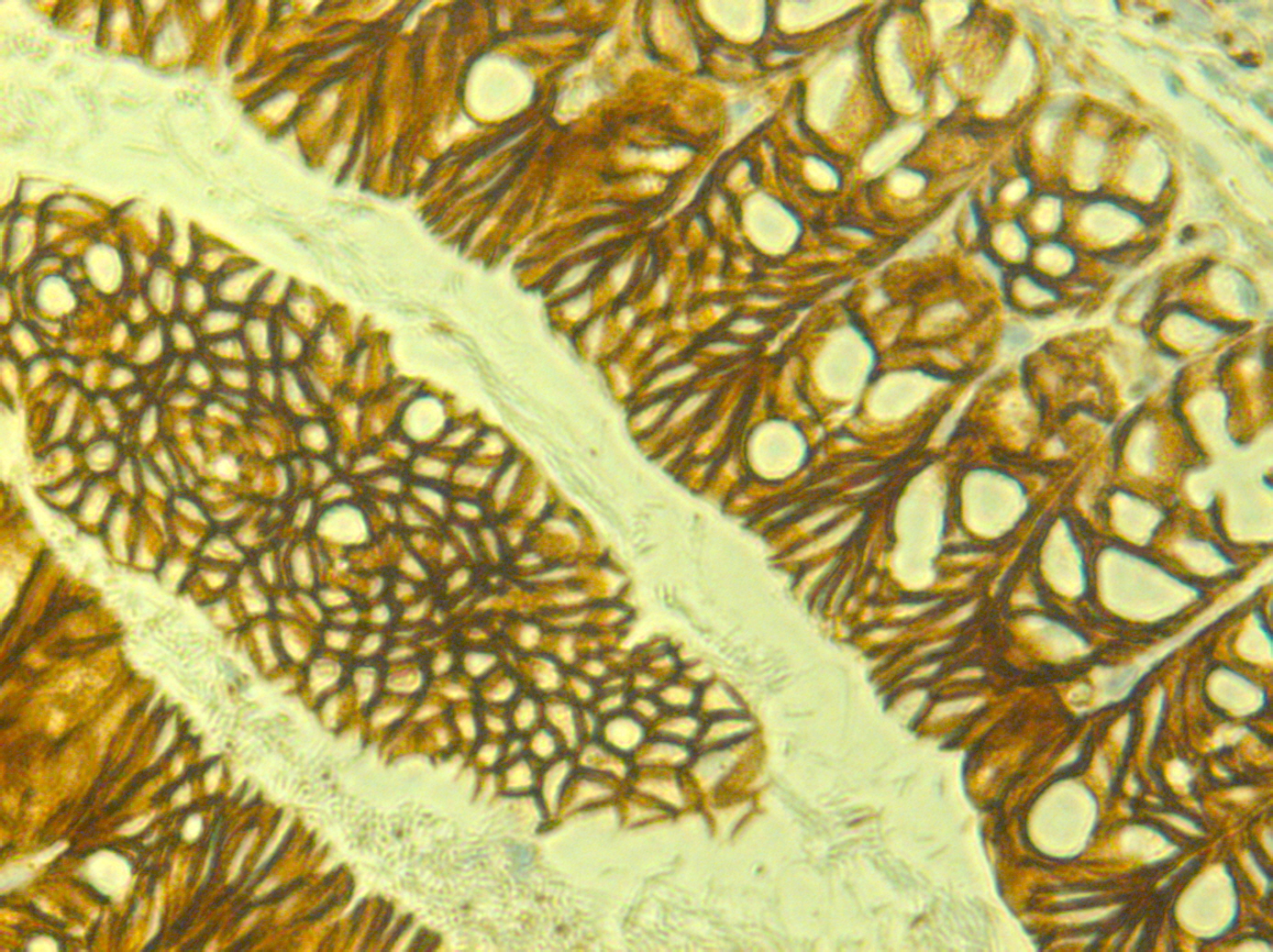

Supplement: Supplementary file 4 — Source Data [file 41467_2024_45605_MOESM4_ESM.zip › Source Data/Figures_Source_Data/supplemental figure 4/Panel d,e,f/D2.jpg]

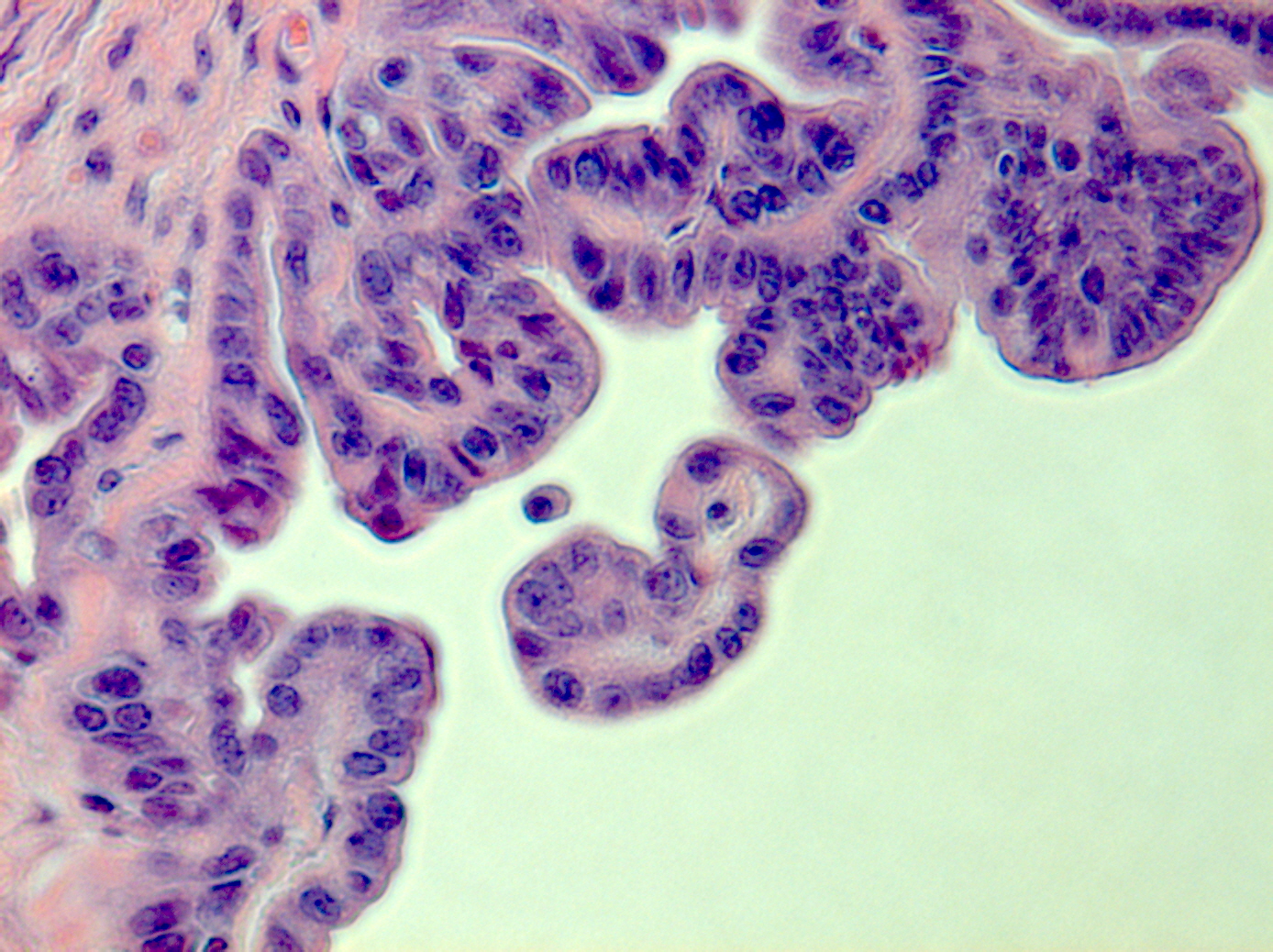

Supplement: Supplementary file 4 — Source Data [file 41467_2024_45605_MOESM4_ESM.zip › Source Data/Figures_Source_Data/supplemental figure 4/Panel d,e,f/F8..jpg]

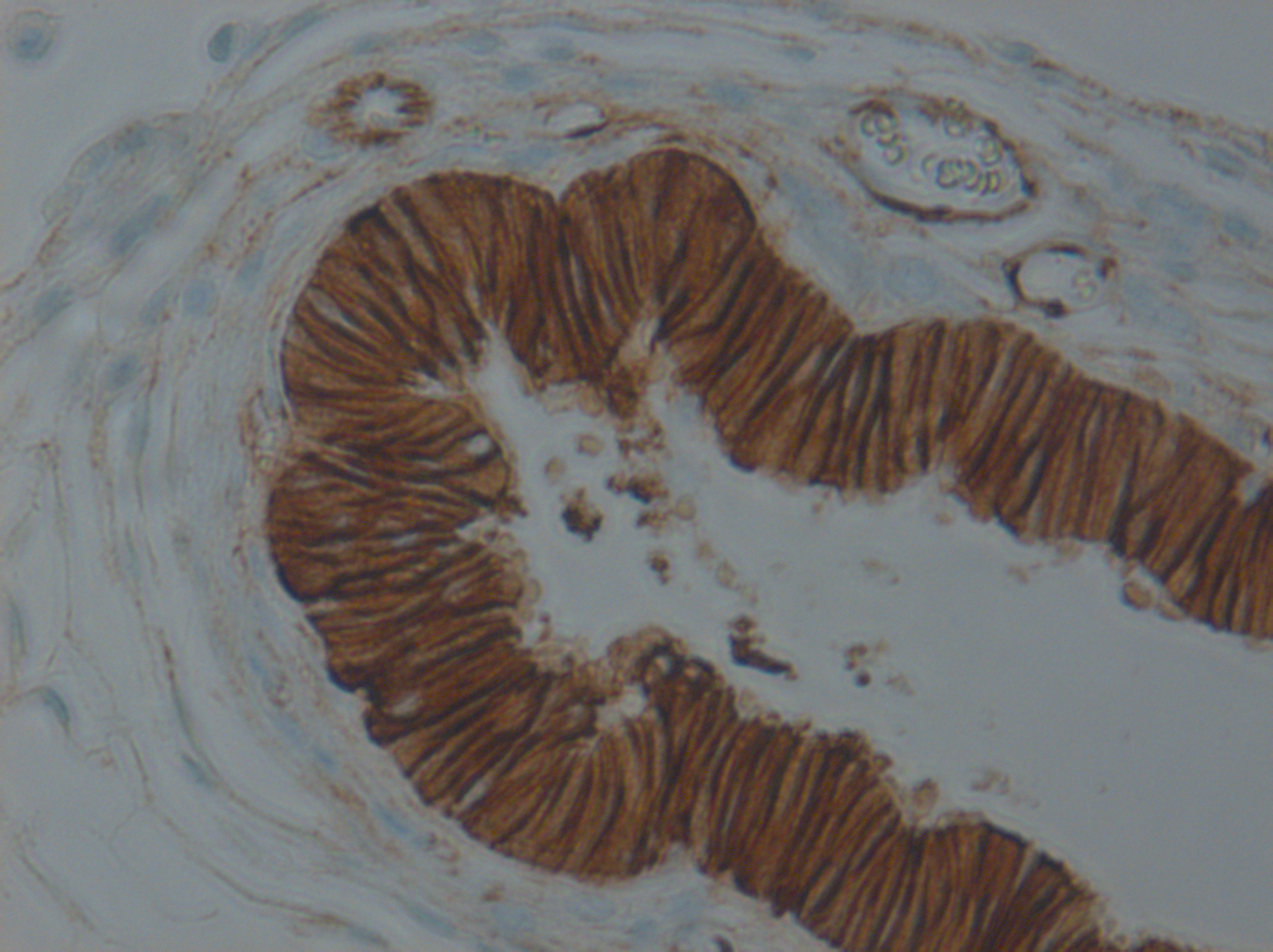

Supplement: Supplementary file 4 — Source Data [file 41467_2024_45605_MOESM4_ESM.zip › Source Data/Figures_Source_Data/supplemental figure 4/Panel d,e,f/E2.tif]

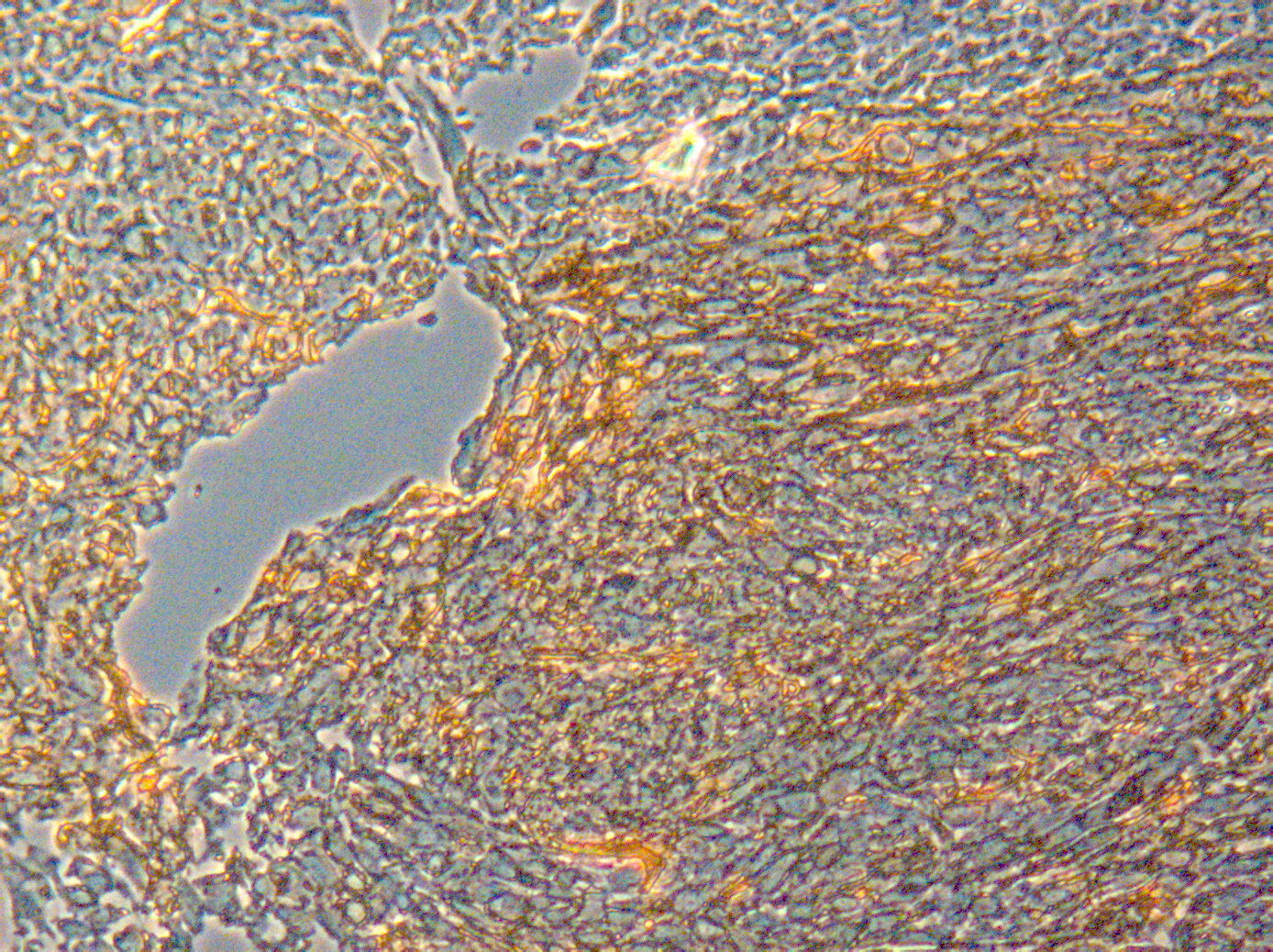

Supplement: Supplementary file 4 — Source Data [file 41467_2024_45605_MOESM4_ESM.zip › Source Data/Figures_Source_Data/supplemental figure 4/Panel d,e,f/F5..jpg]

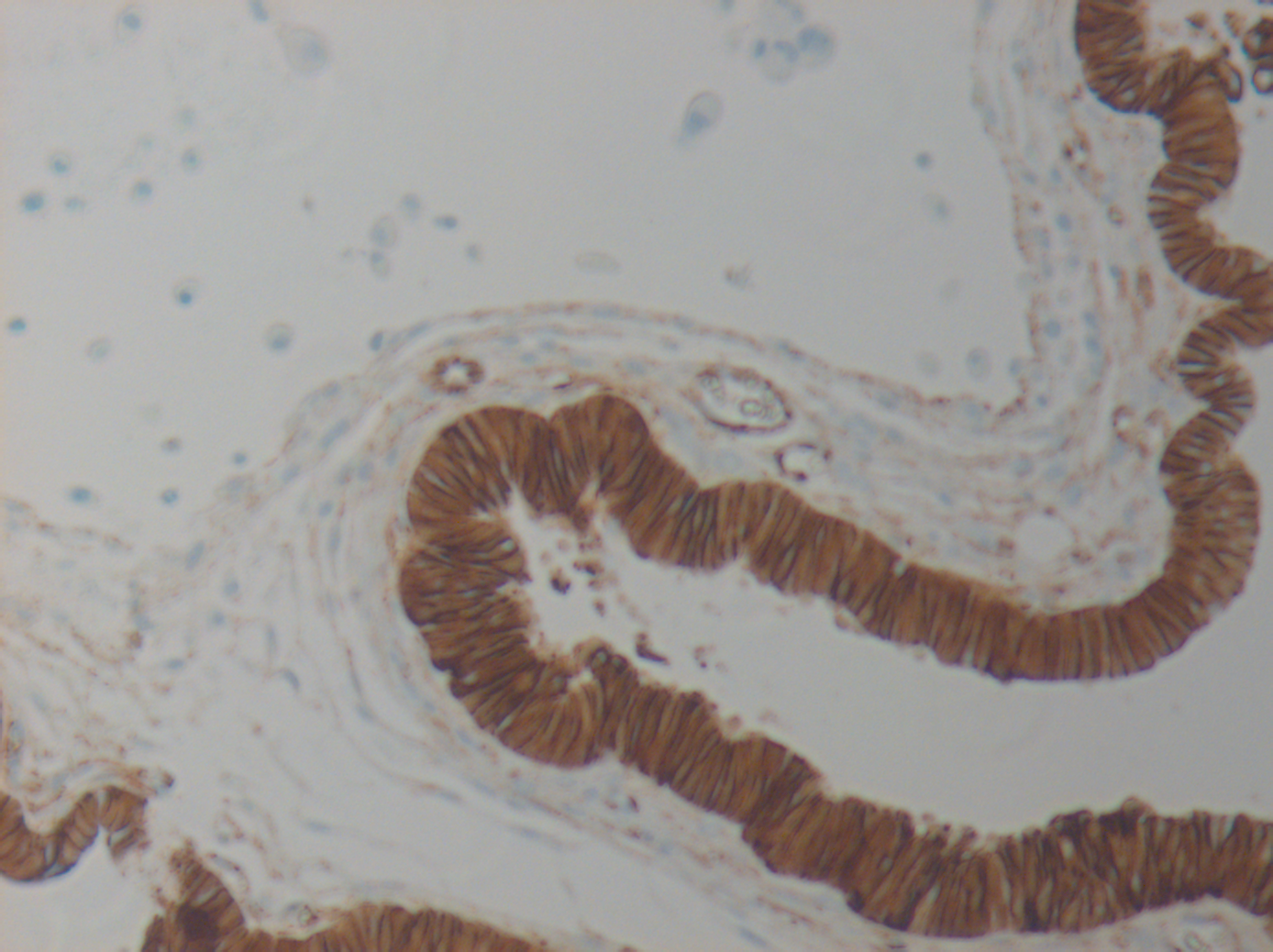

Supplement: Supplementary file 4 — Source Data [file 41467_2024_45605_MOESM4_ESM.zip › Source Data/Figures_Source_Data/supplemental figure 4/Panel d,e,f/E1.tif]

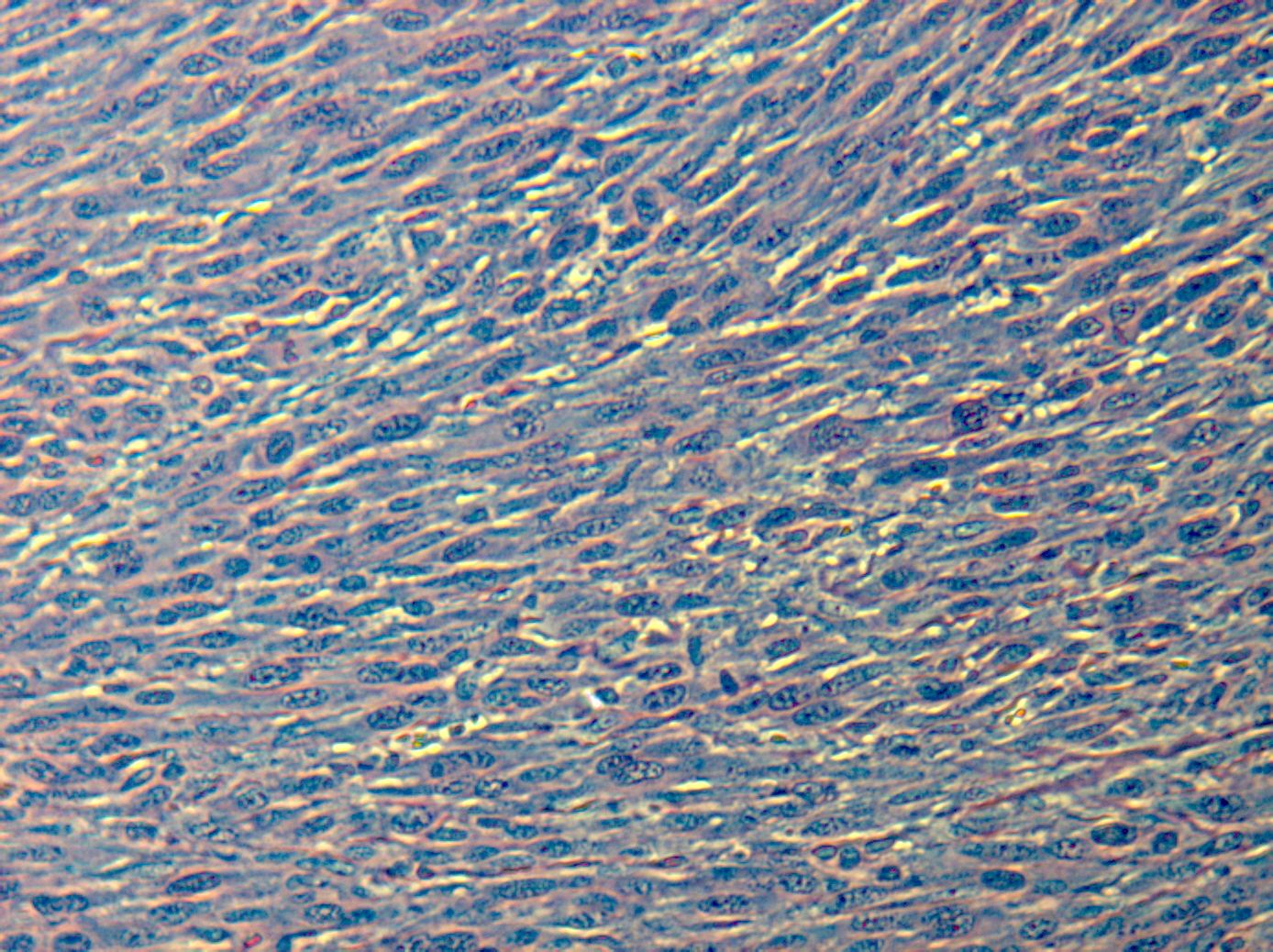

Supplement: Supplementary file 4 — Source Data [file 41467_2024_45605_MOESM4_ESM.zip › Source Data/Figures_Source_Data/supplemental figure 4/Panel d,e,f/F9..jpg]

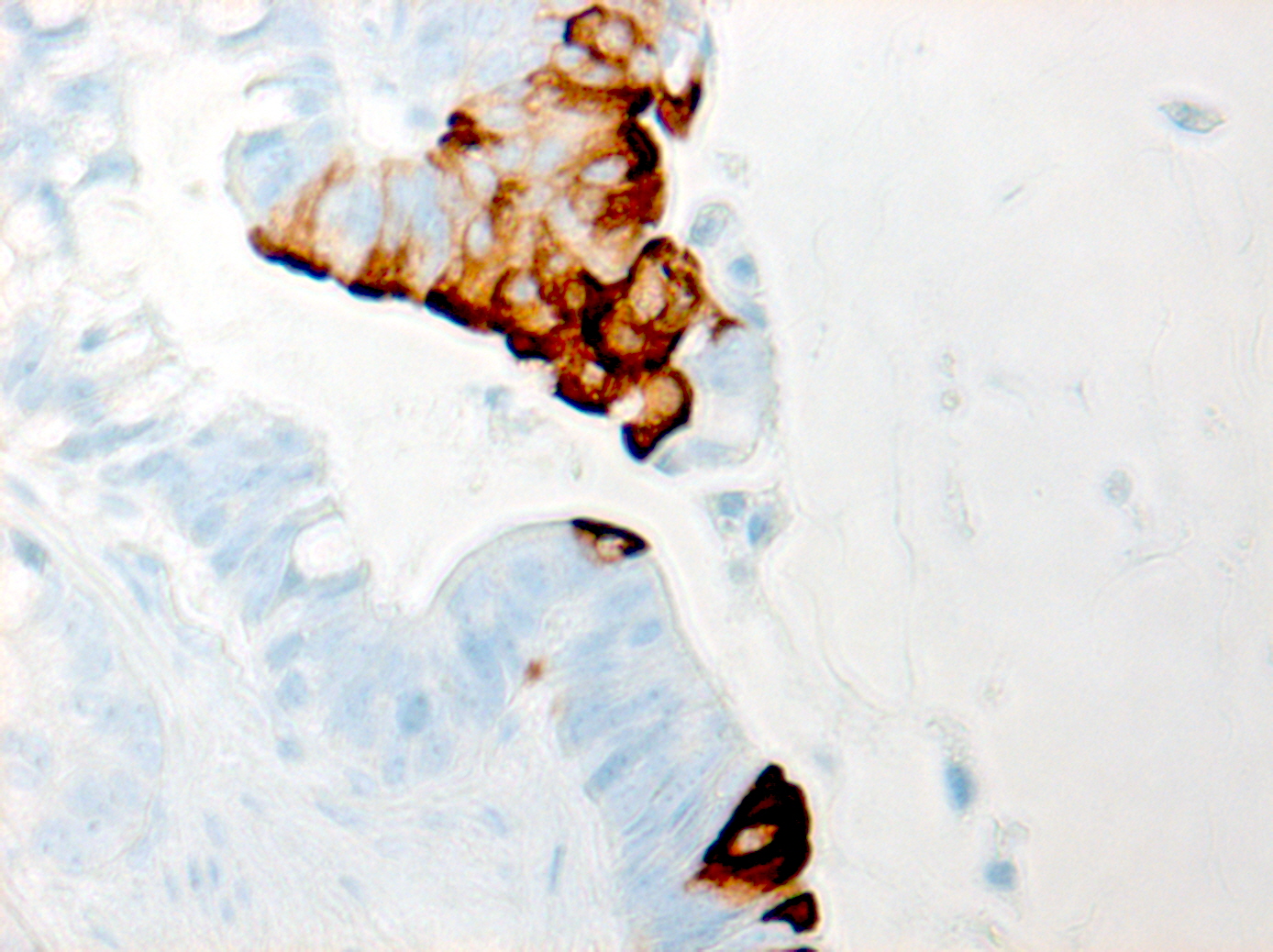

Supplement: Supplementary file 4 — Source Data [file 41467_2024_45605_MOESM4_ESM.zip › Source Data/Figures_Source_Data/supplemental figure 2/panel b/b8.jpg]

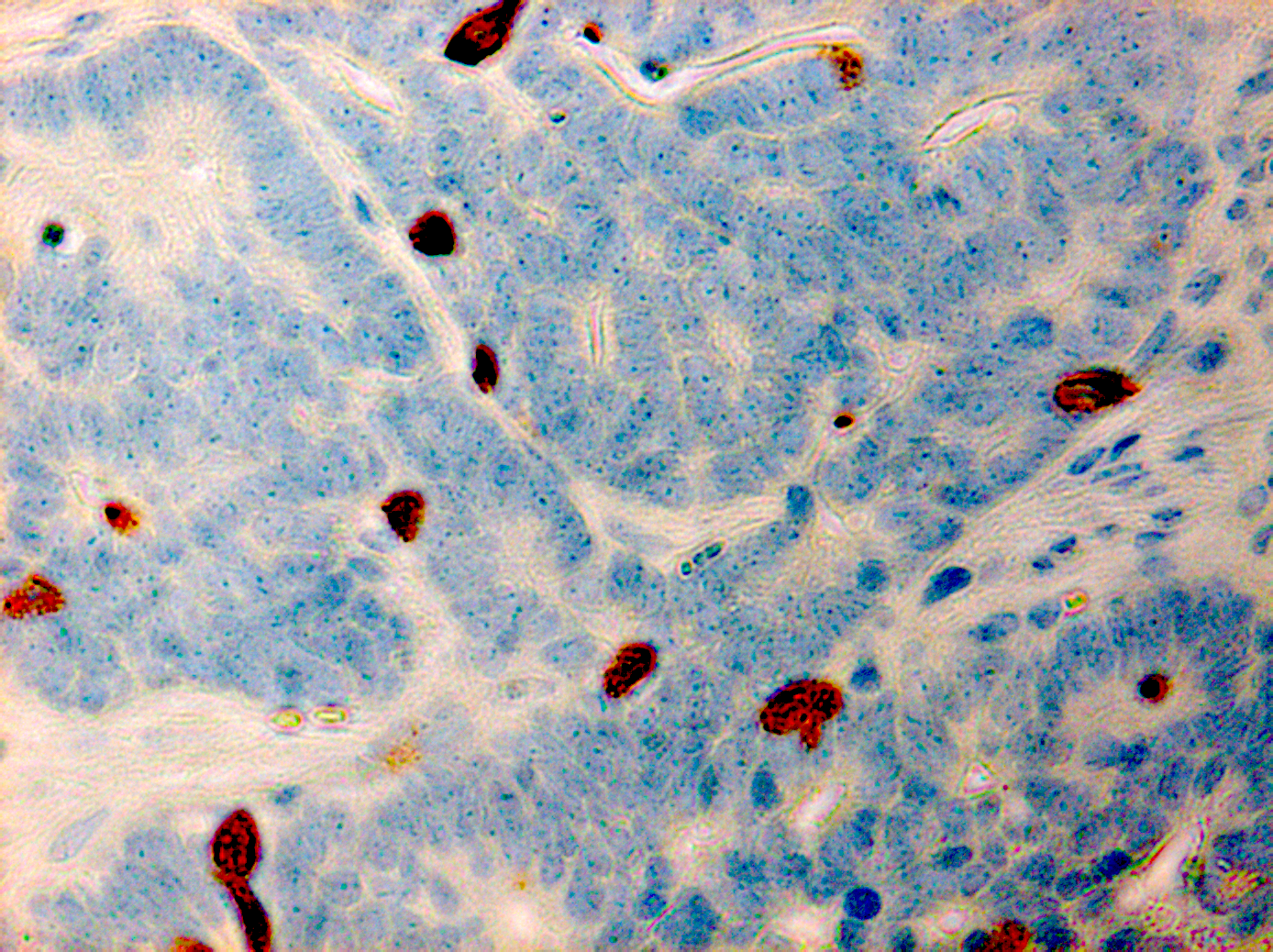

Supplement: Supplementary file 4 — Source Data [file 41467_2024_45605_MOESM4_ESM.zip › Source Data/Figures_Source_Data/supplemental figure 2/panel b/b2.tif]

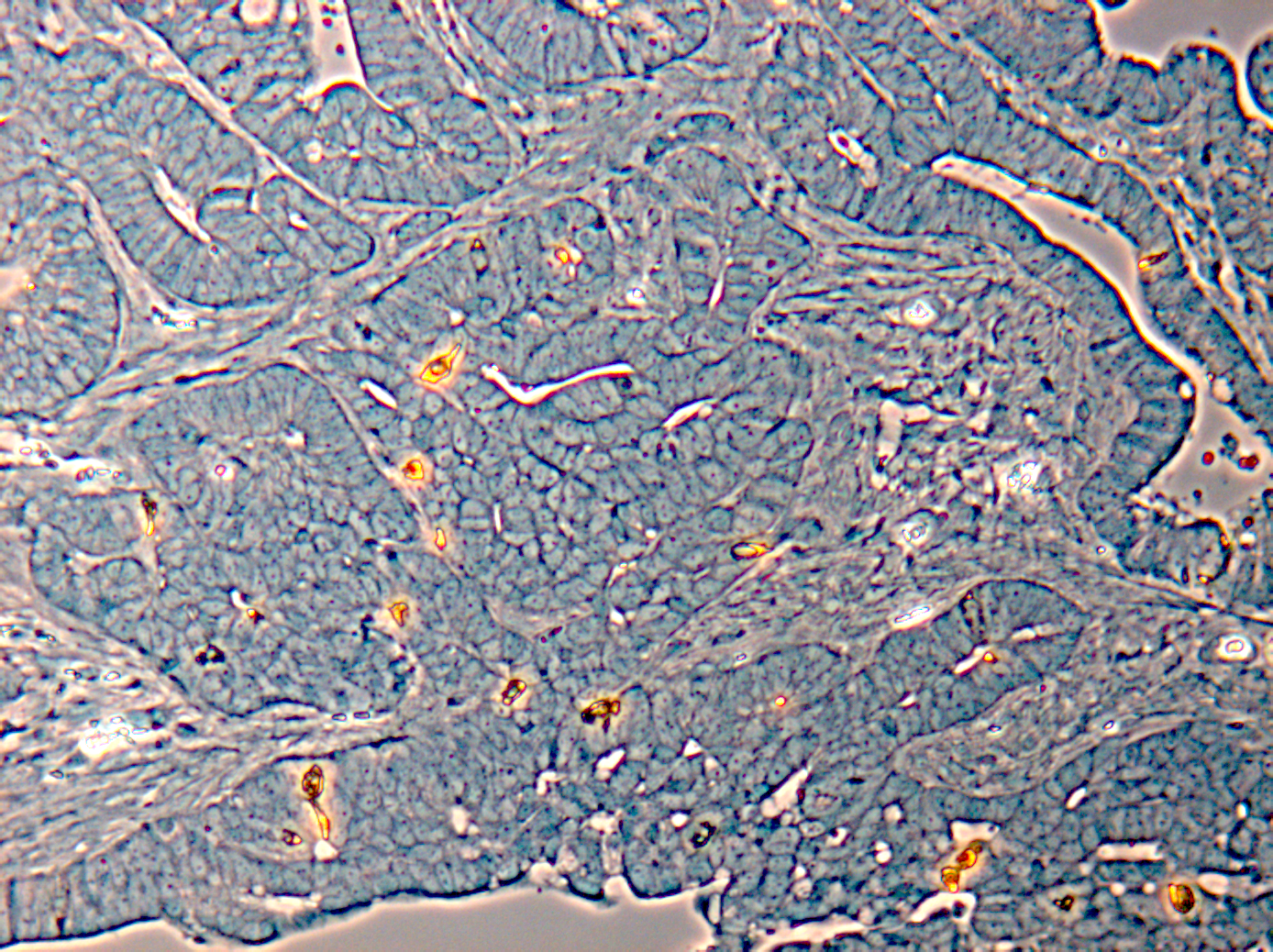

Supplement: Supplementary file 4 — Source Data [file 41467_2024_45605_MOESM4_ESM.zip › Source Data/Figures_Source_Data/supplemental figure 2/panel b/b1.tif]

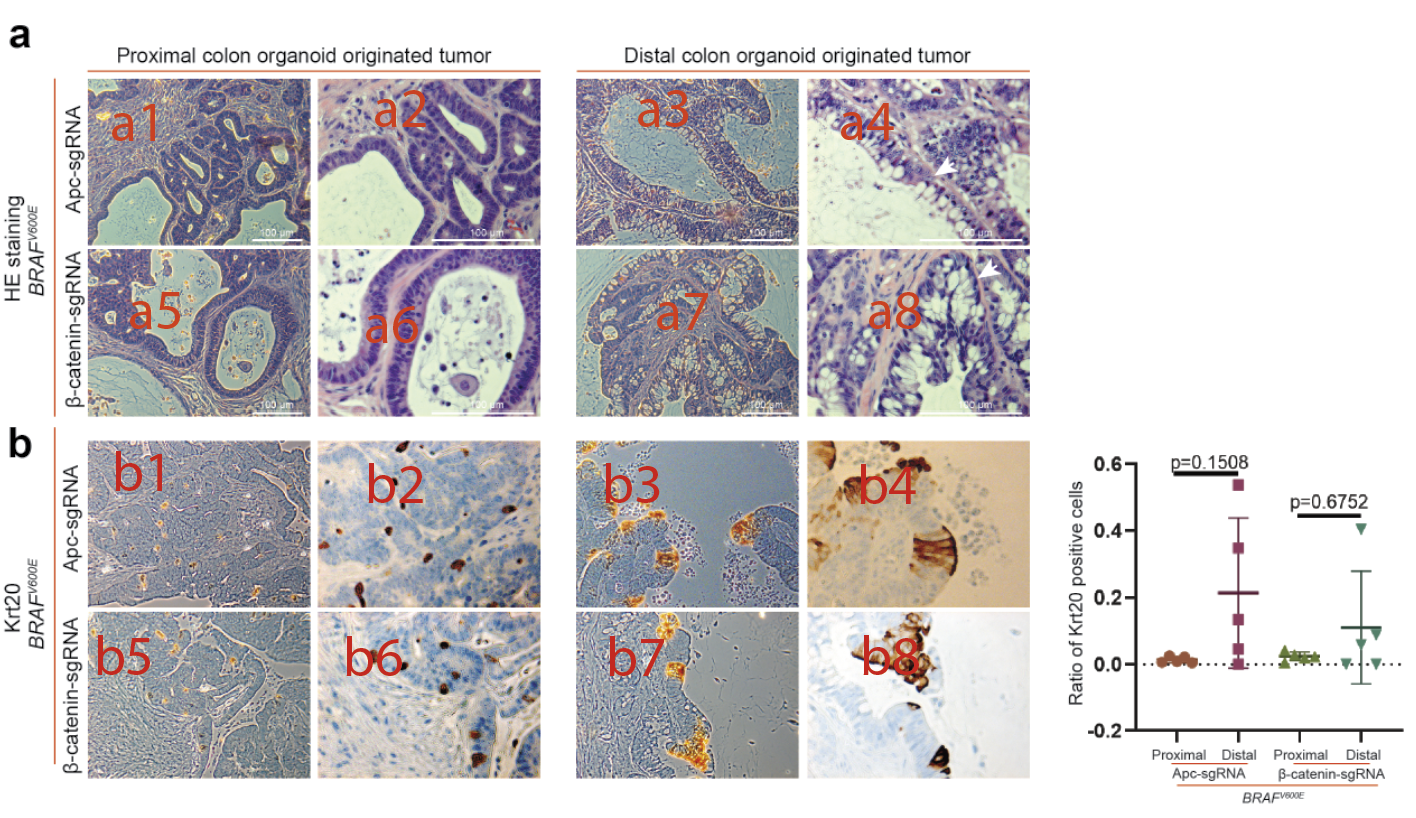

Supplement: Supplementary file 4 — Source Data [file 41467_2024_45605_MOESM4_ESM.zip › Source Data/Figures_Source_Data/supplemental figure 2/panel b/panel a,b.png]

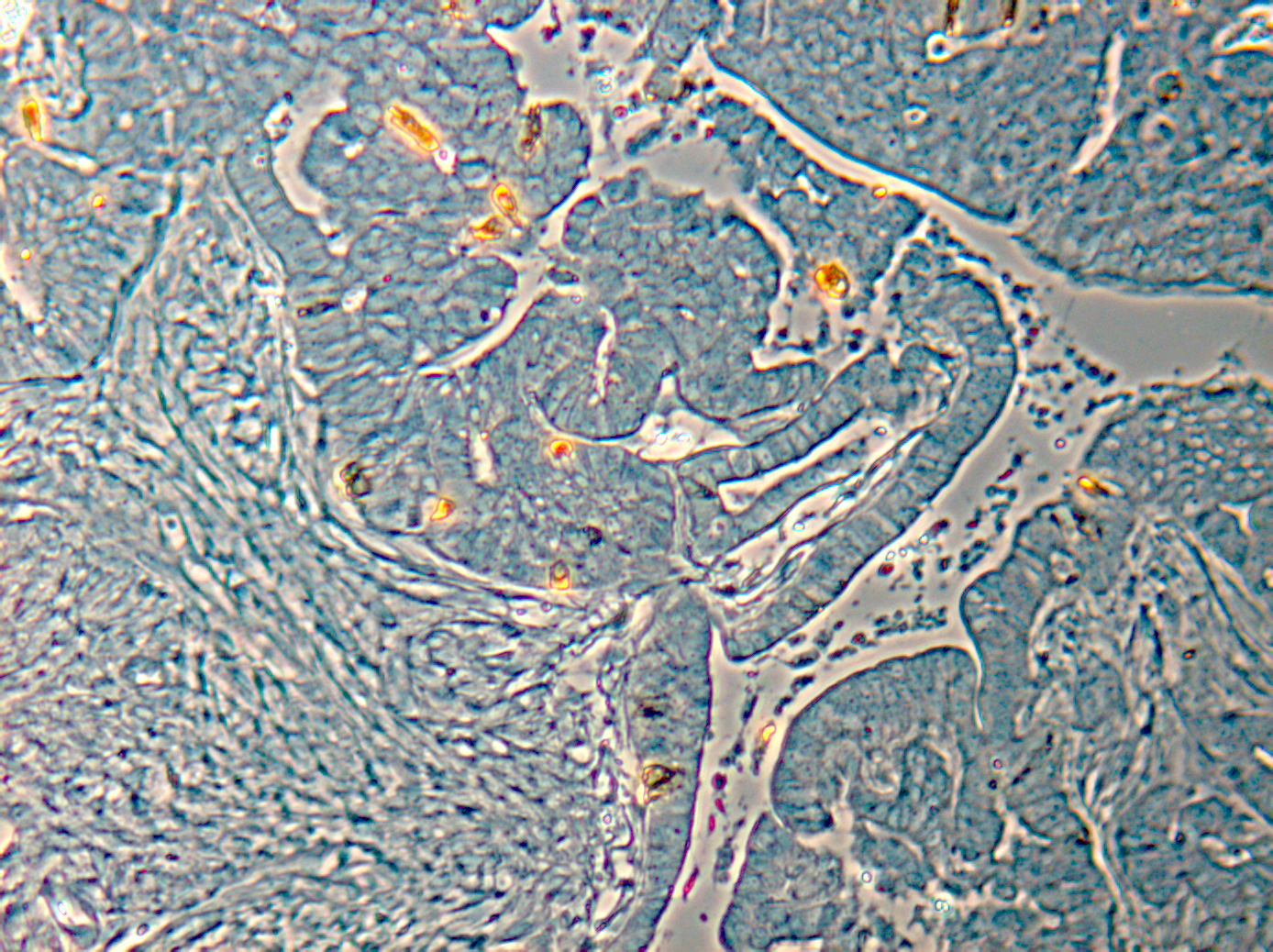

Supplement: Supplementary file 4 — Source Data [file 41467_2024_45605_MOESM4_ESM.zip › Source Data/Figures_Source_Data/supplemental figure 2/panel b/b5.tif]

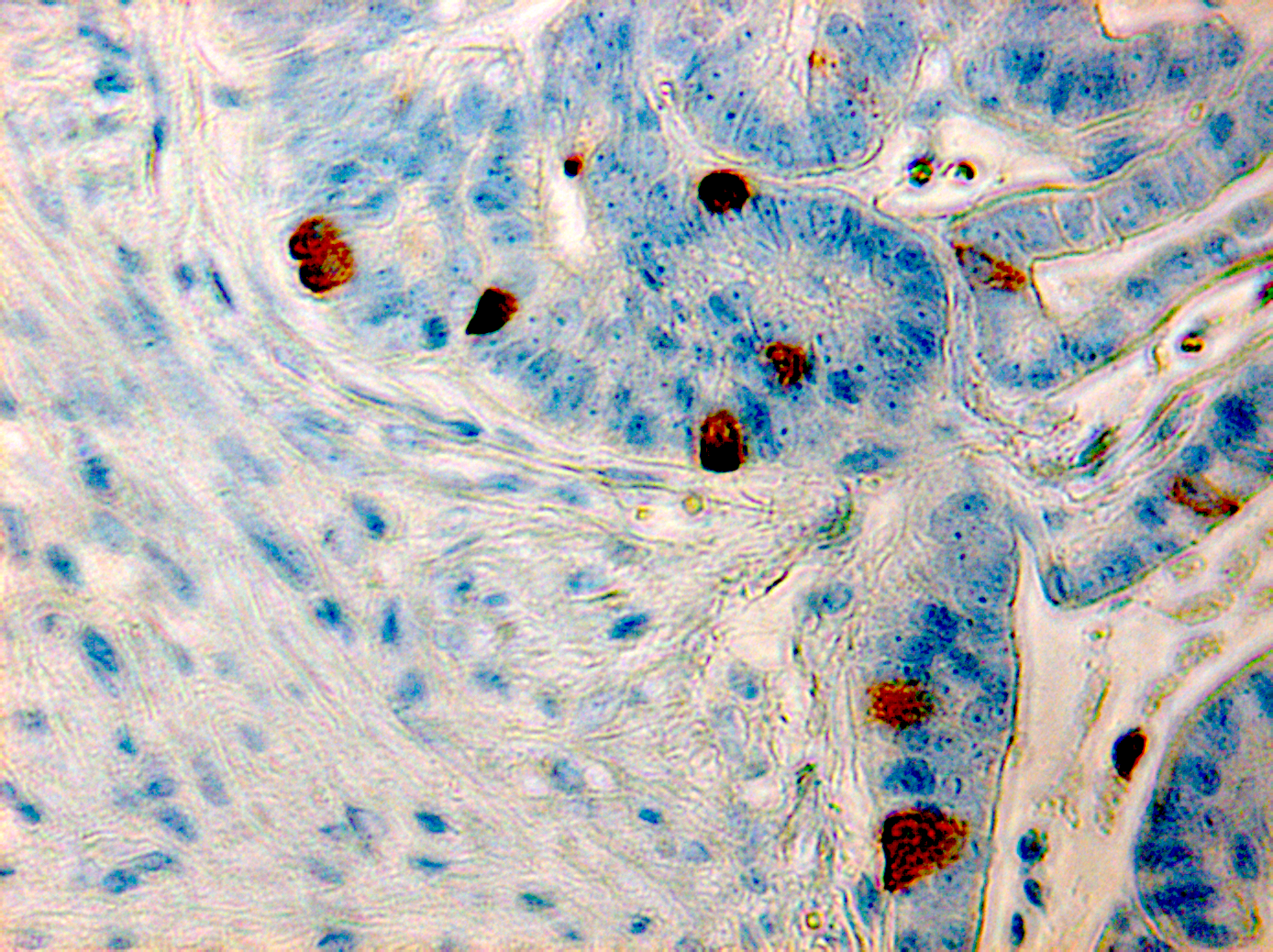

Supplement: Supplementary file 4 — Source Data [file 41467_2024_45605_MOESM4_ESM.zip › Source Data/Figures_Source_Data/supplemental figure 2/panel b/b6.tif]

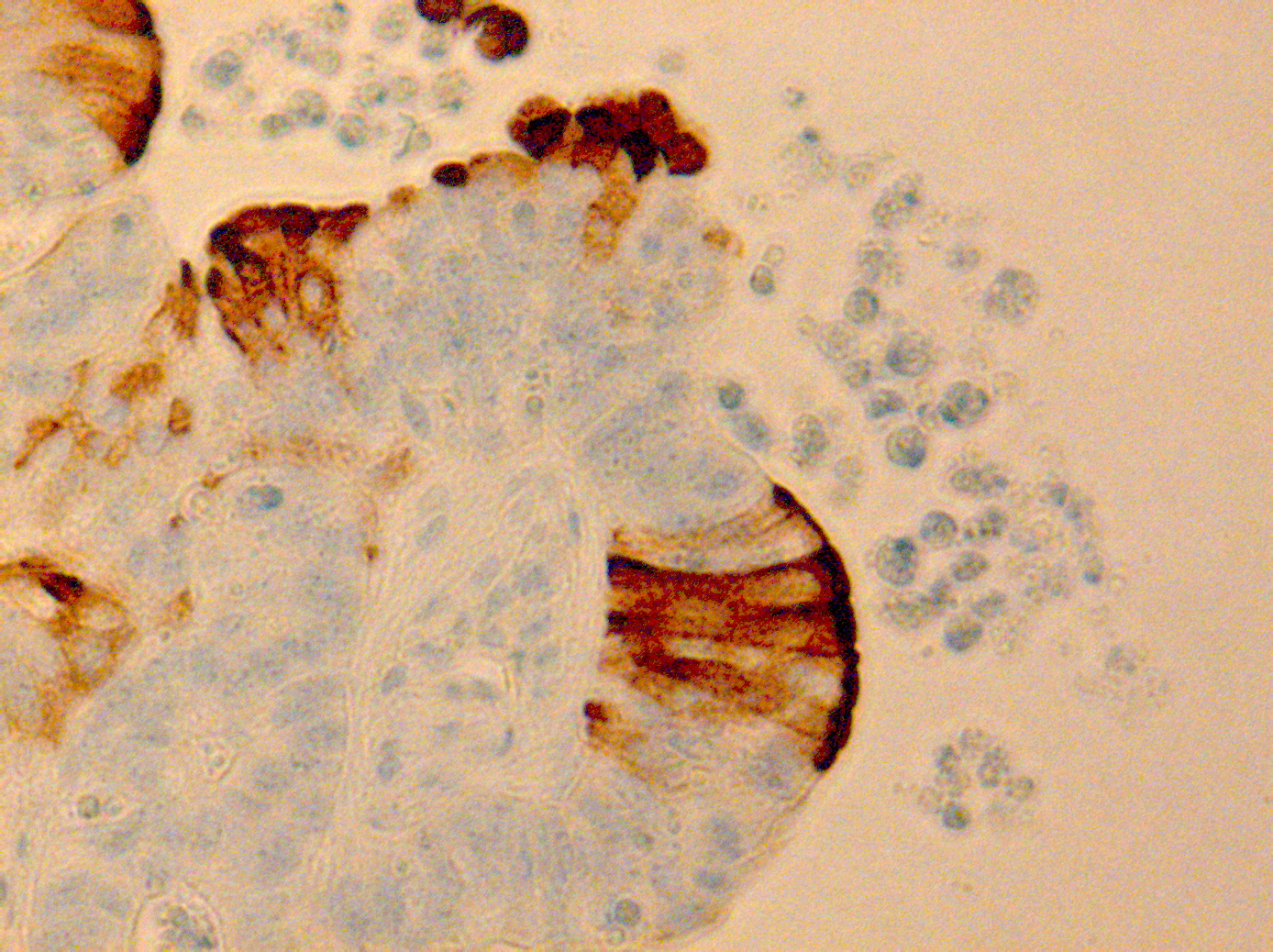

Supplement: Supplementary file 4 — Source Data [file 41467_2024_45605_MOESM4_ESM.zip › Source Data/Figures_Source_Data/supplemental figure 2/panel b/b4.jpg]

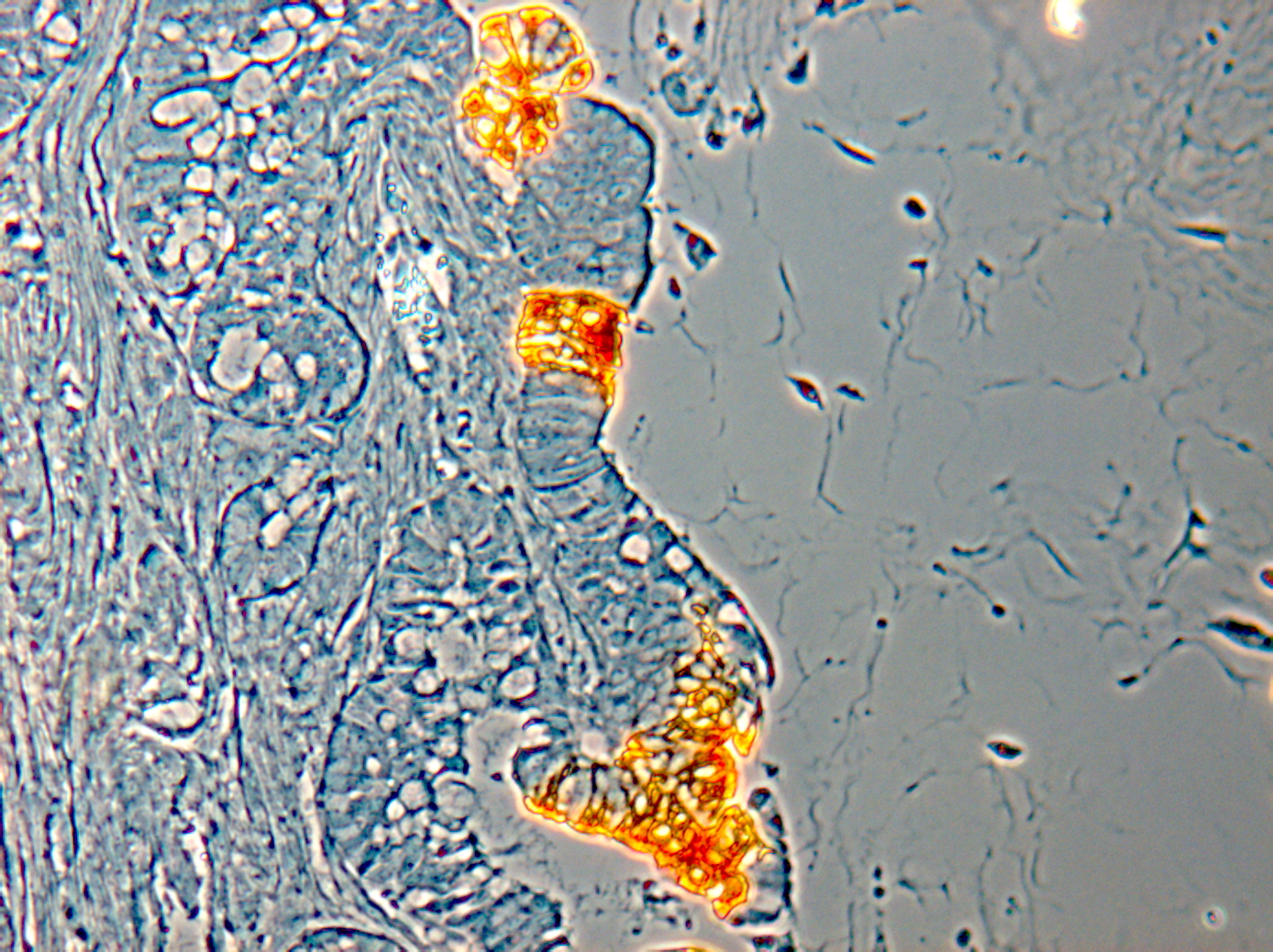

Supplement: Supplementary file 4 — Source Data [file 41467_2024_45605_MOESM4_ESM.zip › Source Data/Figures_Source_Data/supplemental figure 2/panel b/b7.jpg]

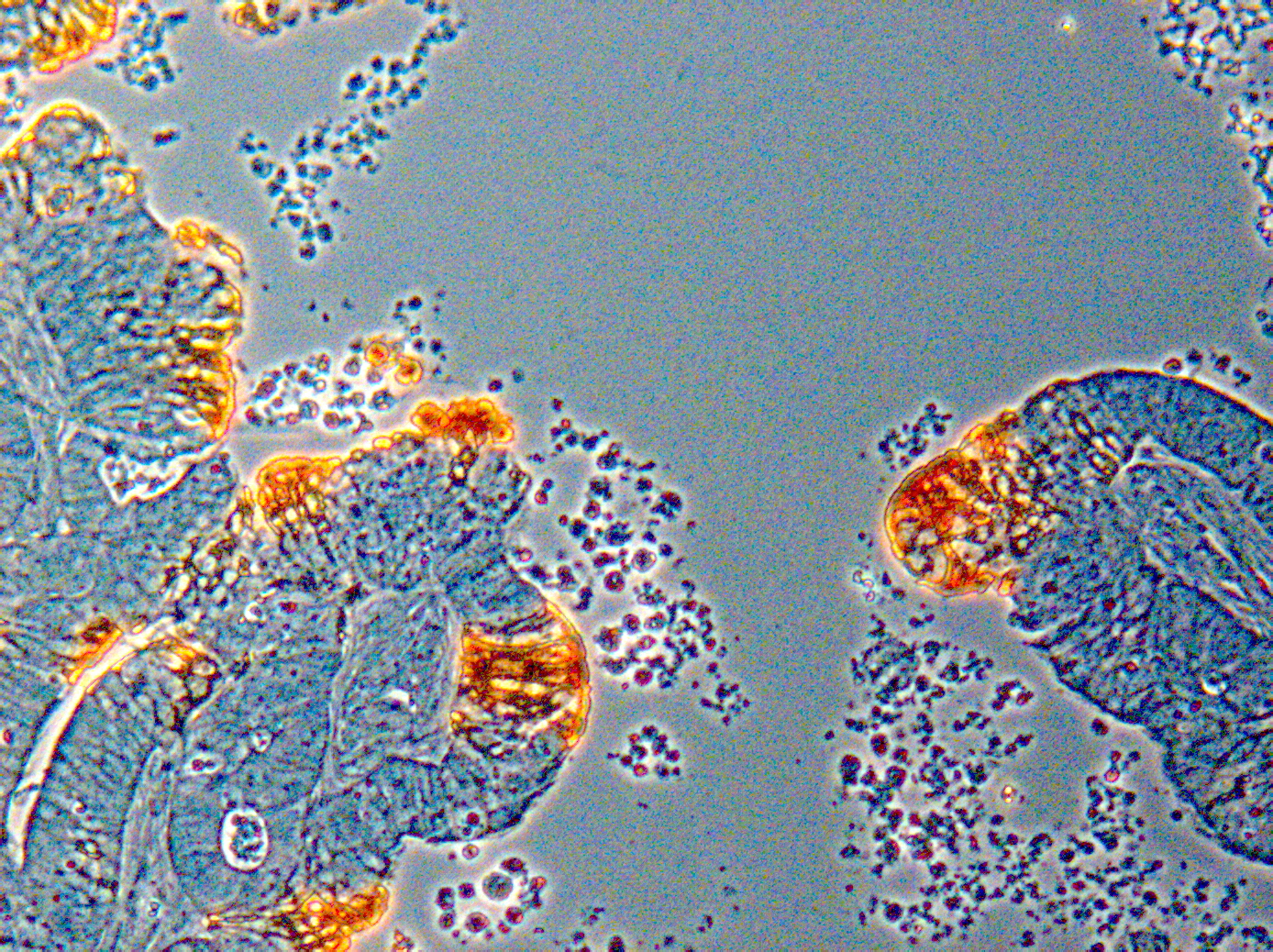

Supplement: Supplementary file 4 — Source Data [file 41467_2024_45605_MOESM4_ESM.zip › Source Data/Figures_Source_Data/supplemental figure 2/panel b/b3.jpg]

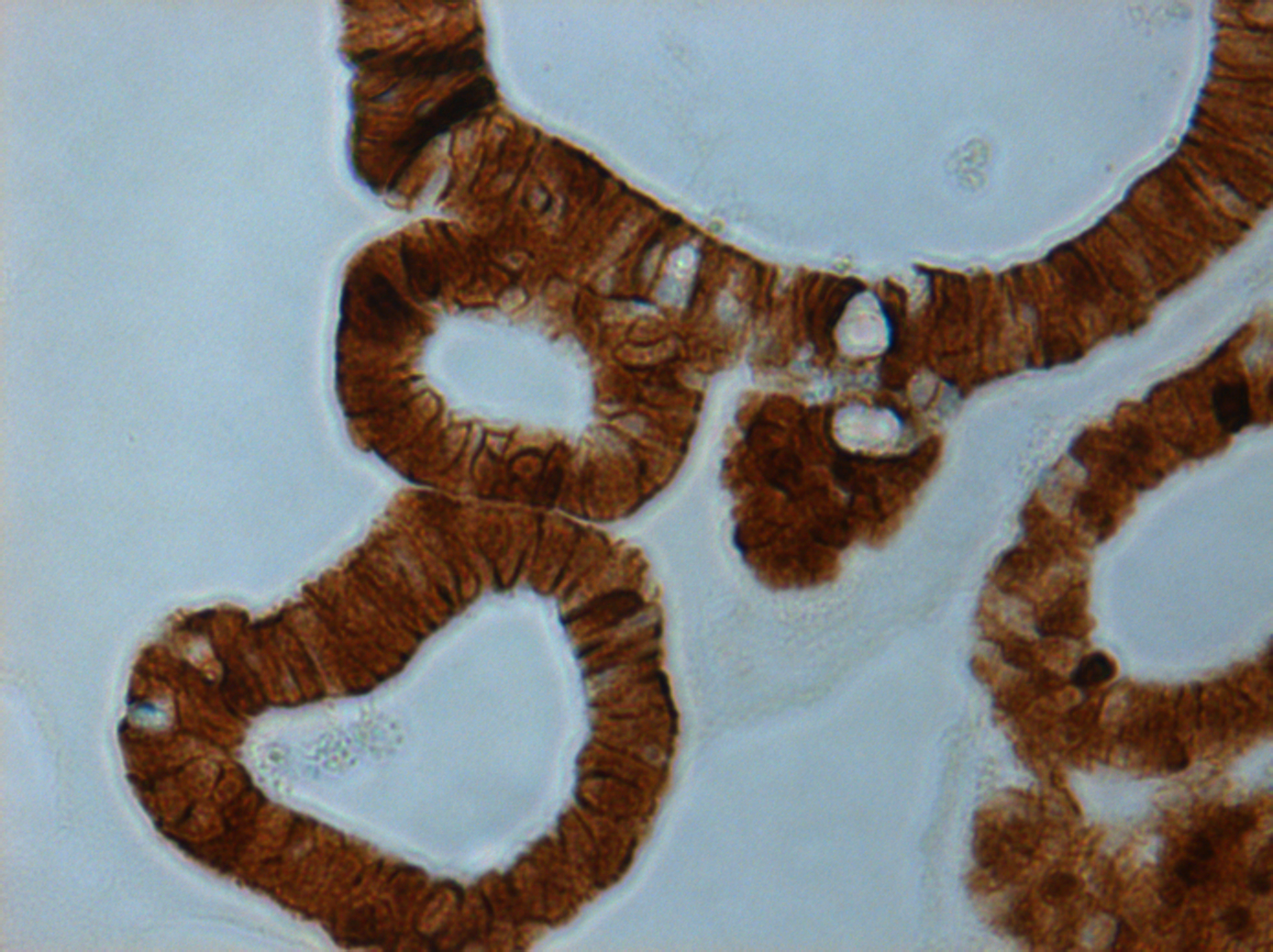

Supplement: Supplementary file 4 — Source Data [file 41467_2024_45605_MOESM4_ESM.zip › Source Data/Figures_Source_Data/supplemental figure 2/panel d/D8.tif]

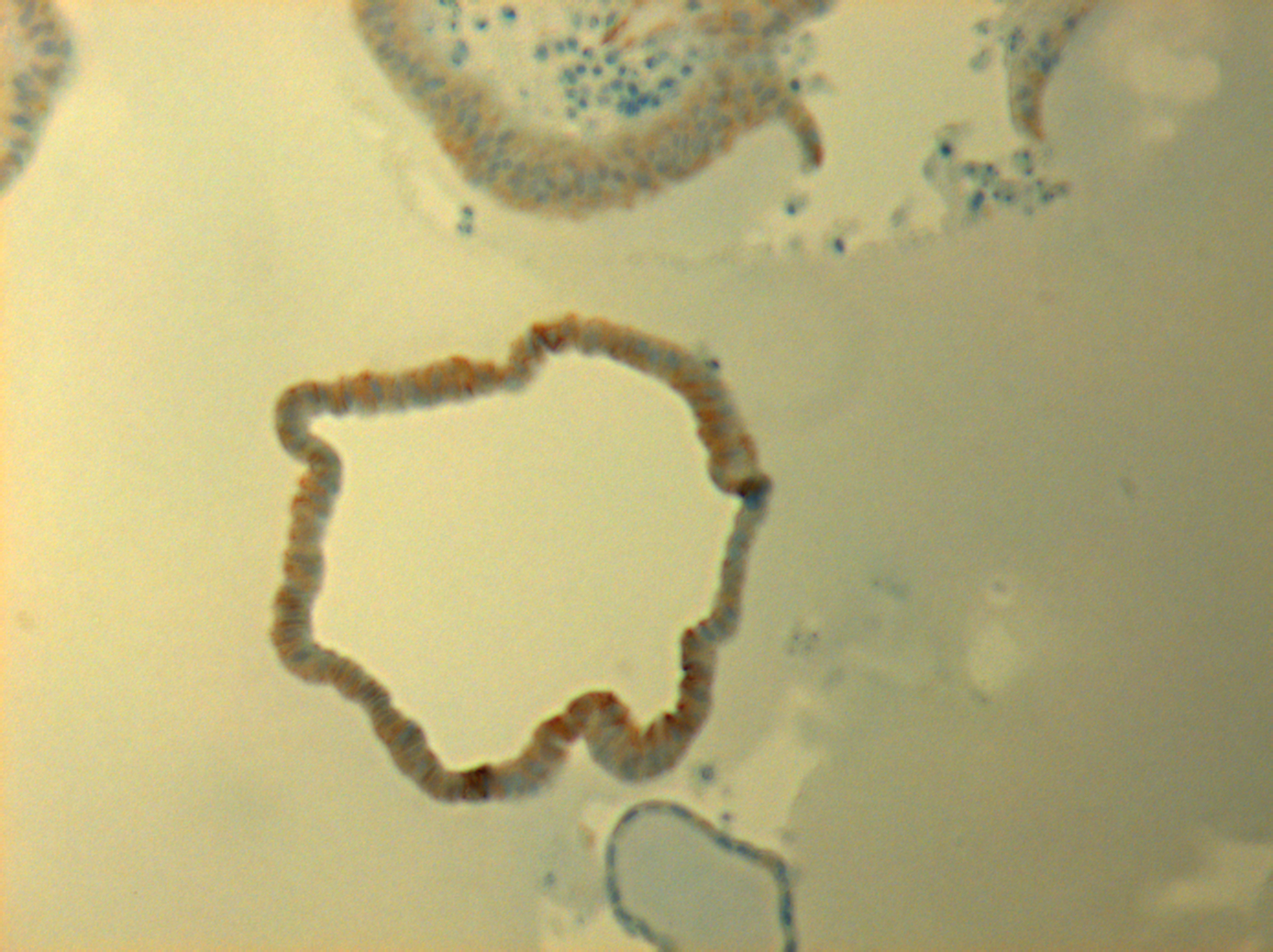

Supplement: Supplementary file 4 — Source Data [file 41467_2024_45605_MOESM4_ESM.zip › Source Data/Figures_Source_Data/supplemental figure 2/panel d/D9.tif]

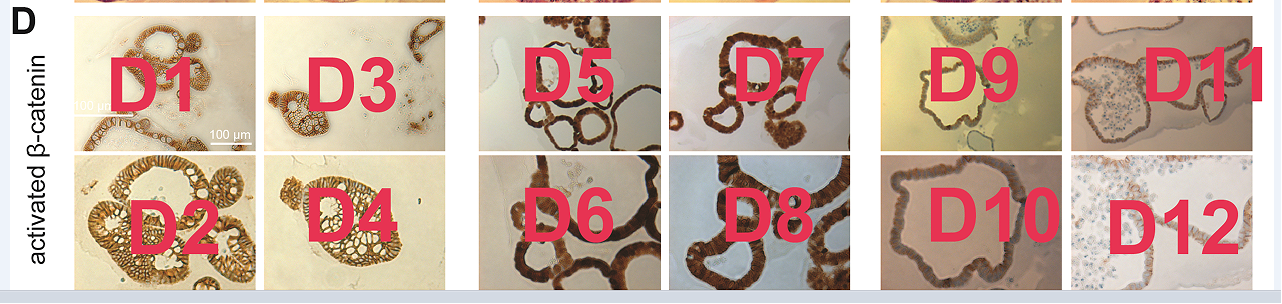

Supplement: Supplementary file 4 — Source Data [file 41467_2024_45605_MOESM4_ESM.zip › Source Data/Figures_Source_Data/supplemental figure 2/panel d/panel d.png]

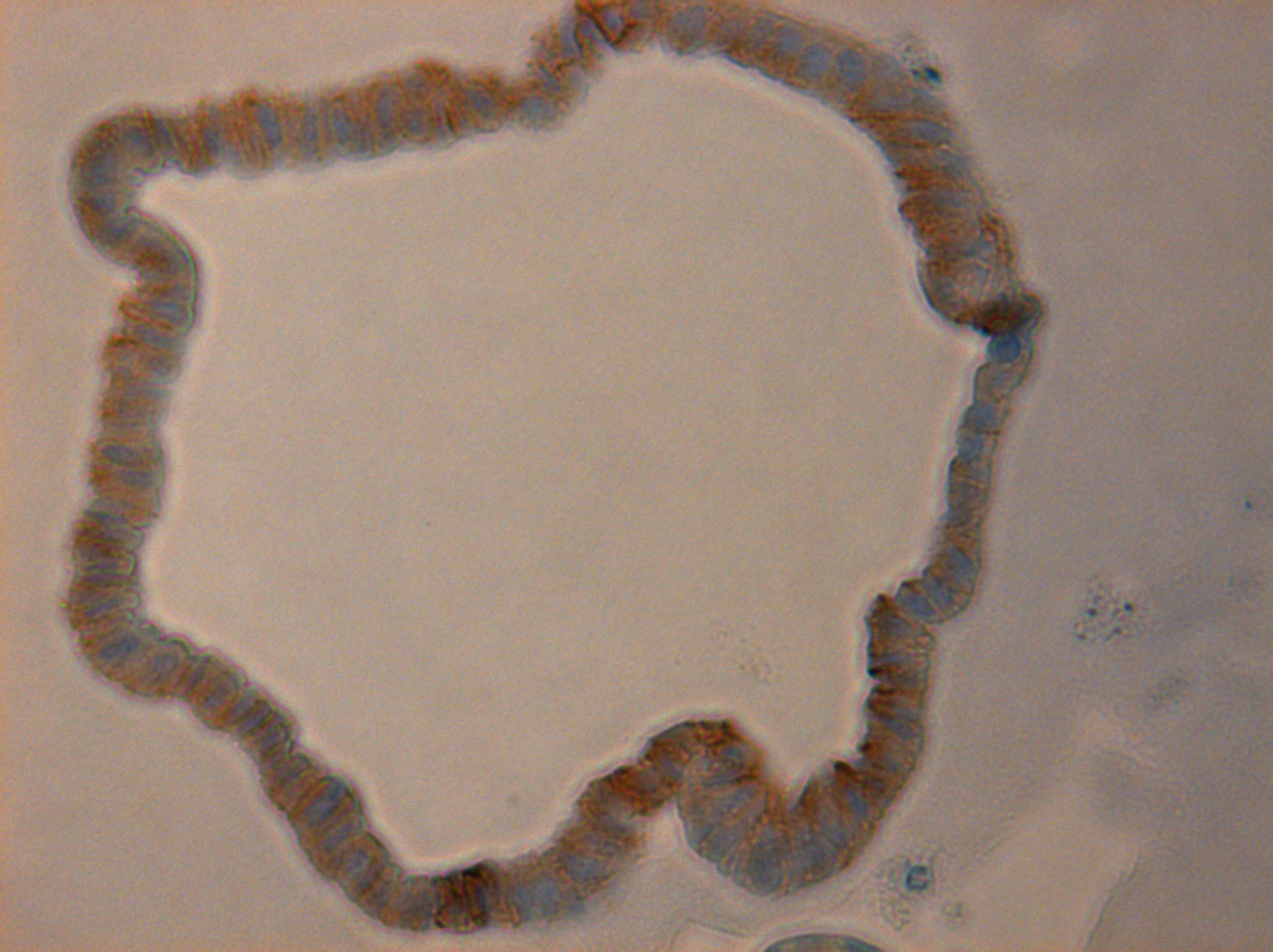

Supplement: Supplementary file 4 — Source Data [file 41467_2024_45605_MOESM4_ESM.zip › Source Data/Figures_Source_Data/supplemental figure 2/panel d/D10.tif]

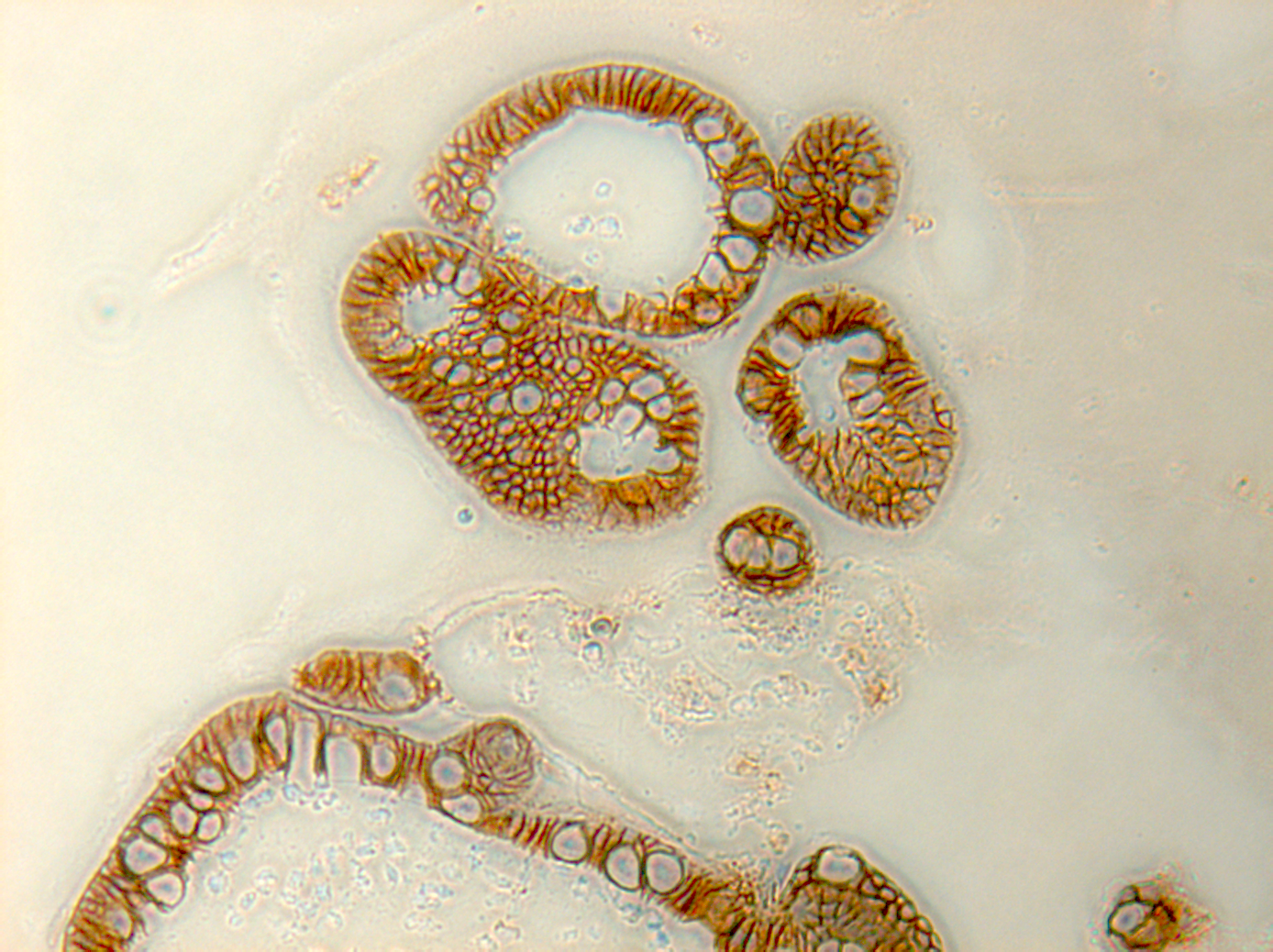

Supplement: Supplementary file 4 — Source Data [file 41467_2024_45605_MOESM4_ESM.zip › Source Data/Figures_Source_Data/supplemental figure 2/panel d/D1.tif]

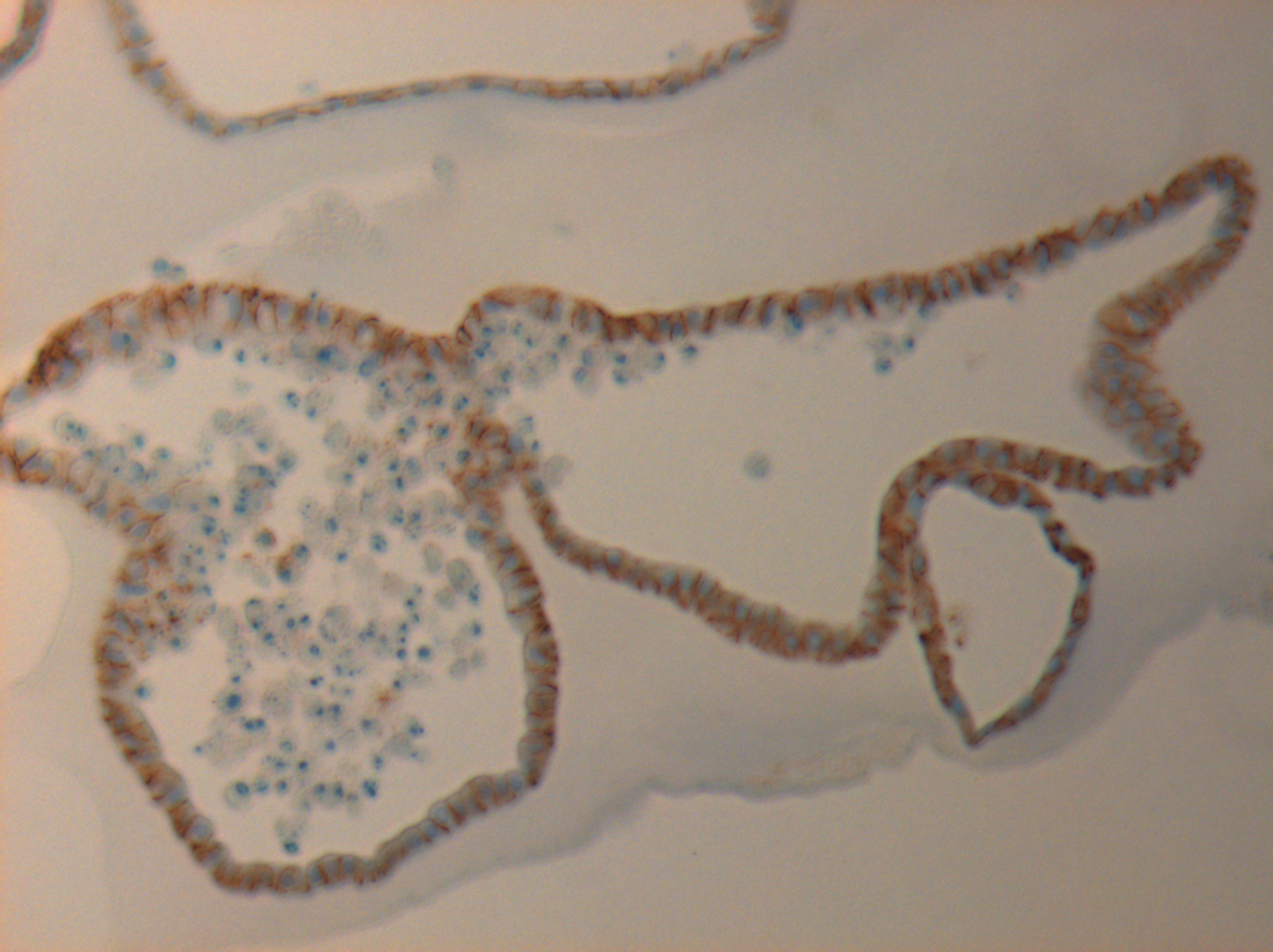

Supplement: Supplementary file 4 — Source Data [file 41467_2024_45605_MOESM4_ESM.zip › Source Data/Figures_Source_Data/supplemental figure 2/panel d/D11.tif]

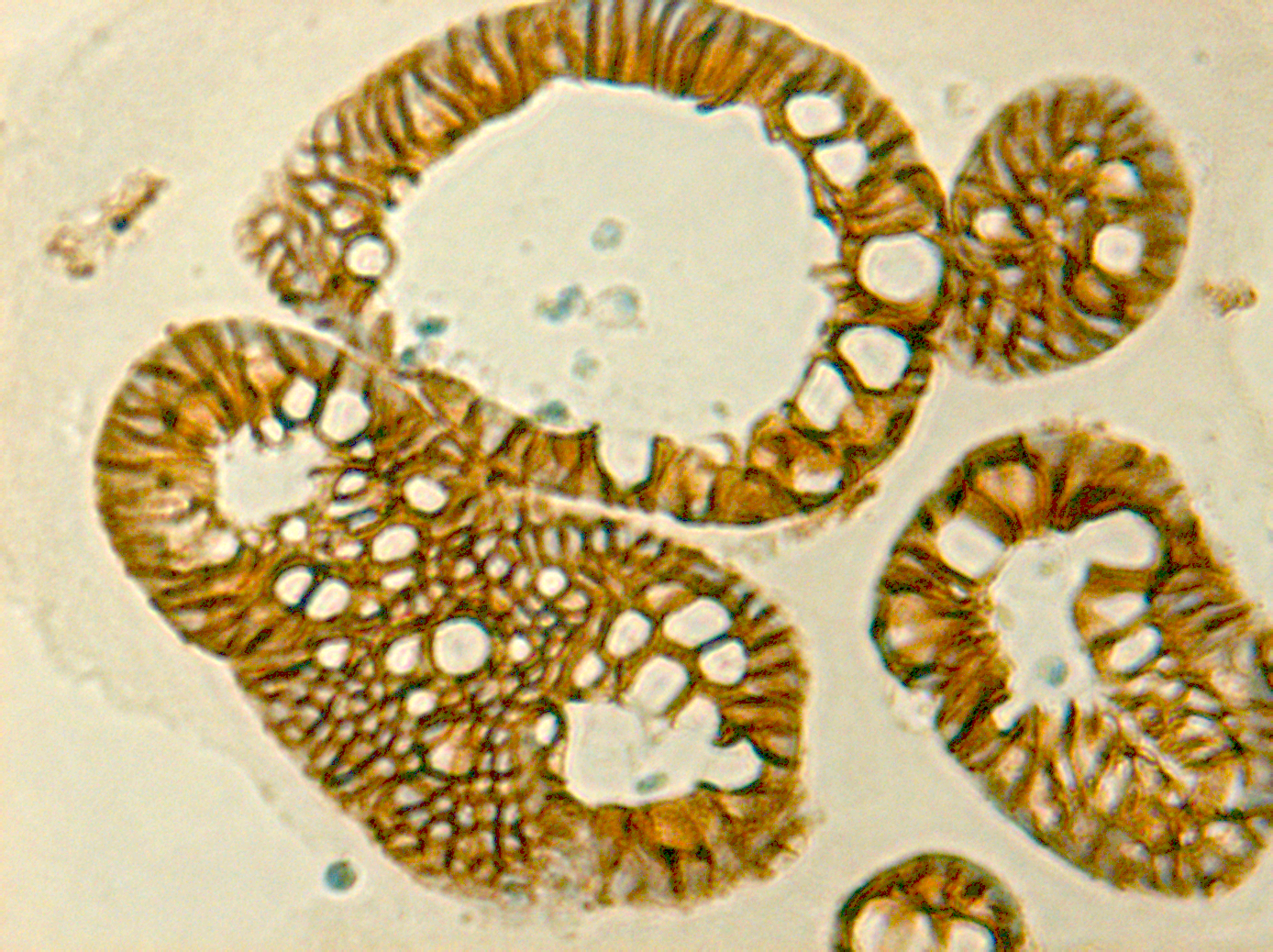

Supplement: Supplementary file 4 — Source Data [file 41467_2024_45605_MOESM4_ESM.zip › Source Data/Figures_Source_Data/supplemental figure 2/panel d/D2.tif]

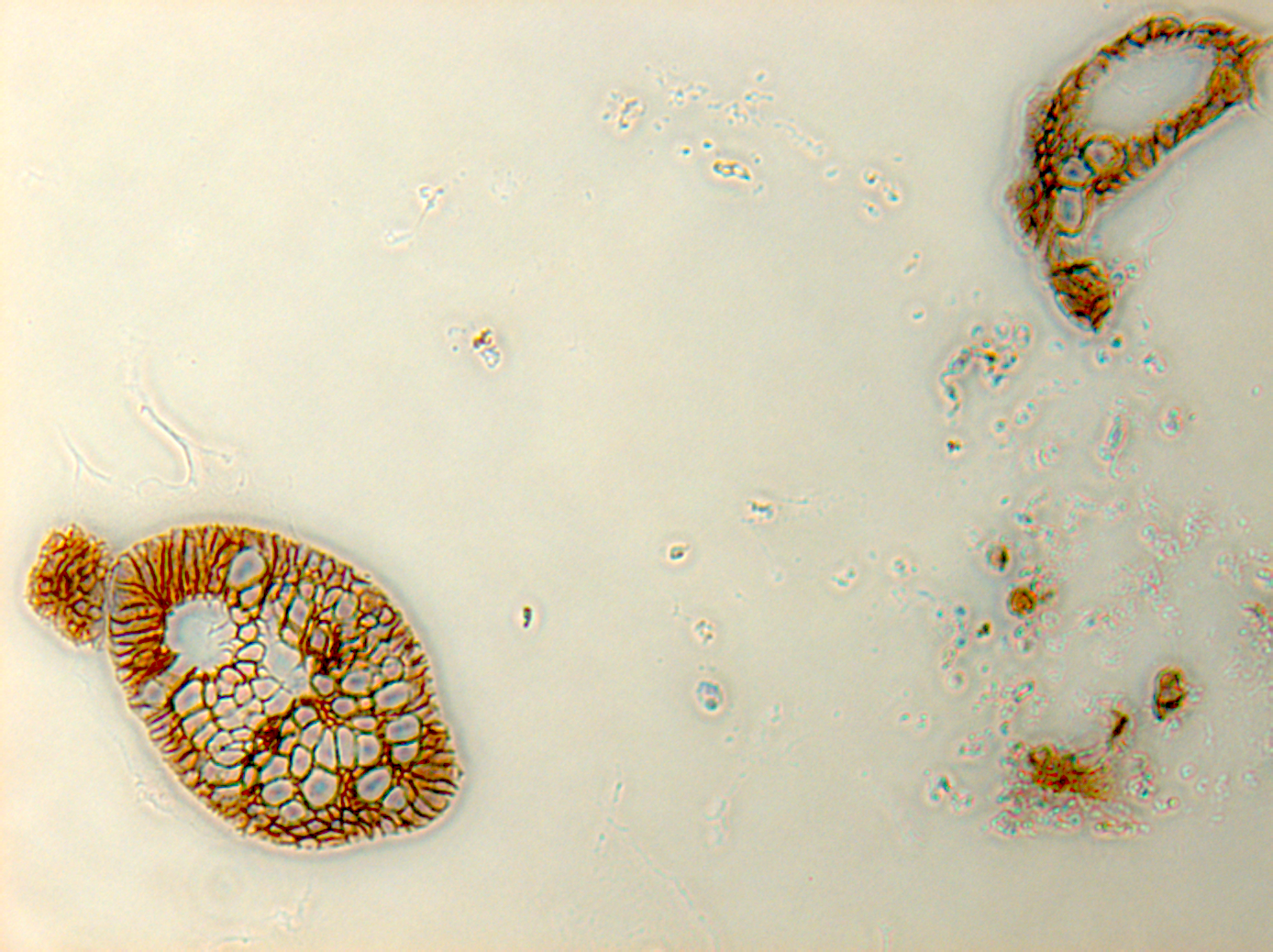

Supplement: Supplementary file 4 — Source Data [file 41467_2024_45605_MOESM4_ESM.zip › Source Data/Figures_Source_Data/supplemental figure 2/panel d/D3.tif]

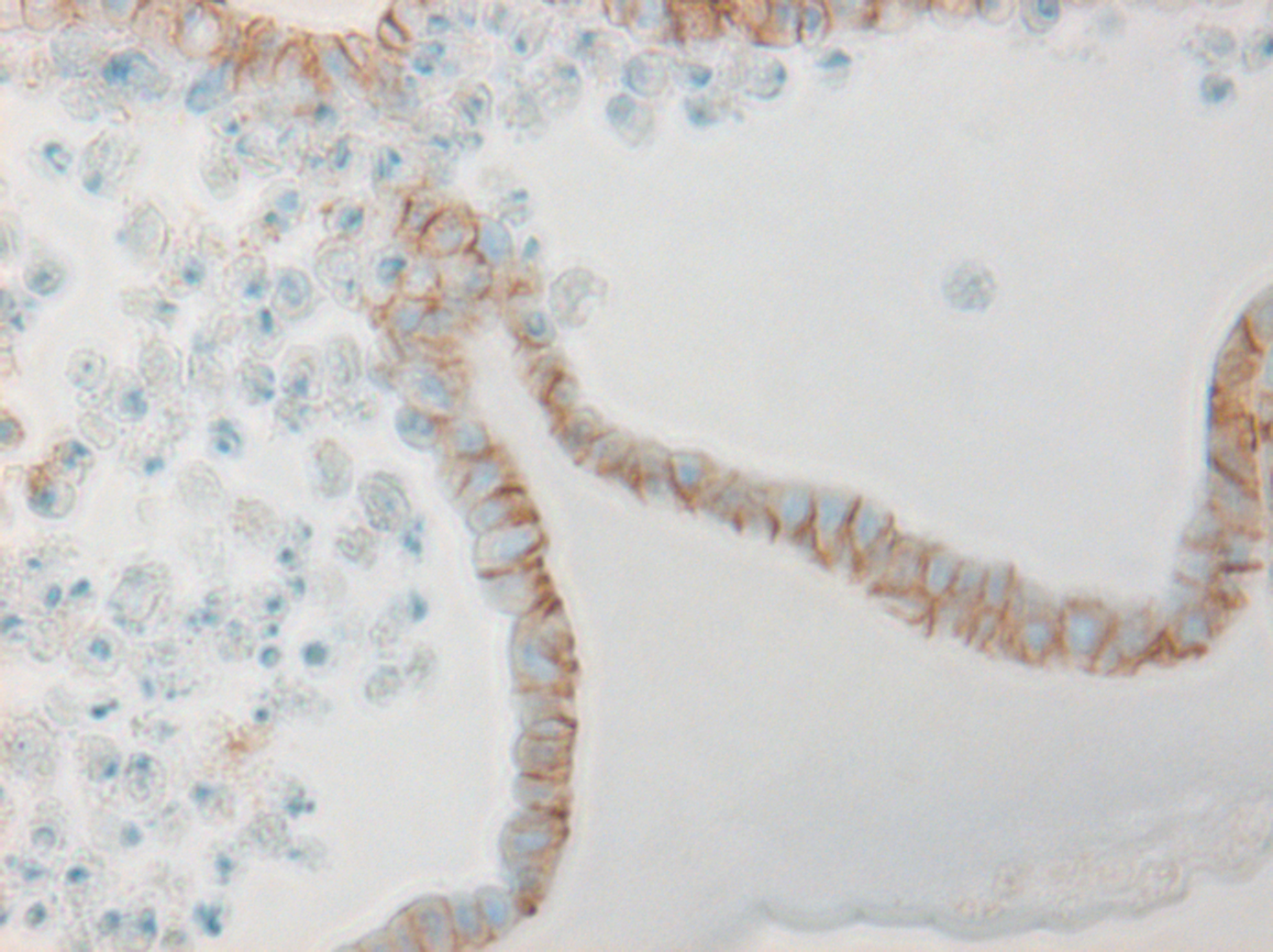

Supplement: Supplementary file 4 — Source Data [file 41467_2024_45605_MOESM4_ESM.zip › Source Data/Figures_Source_Data/supplemental figure 2/panel d/D12.tif]

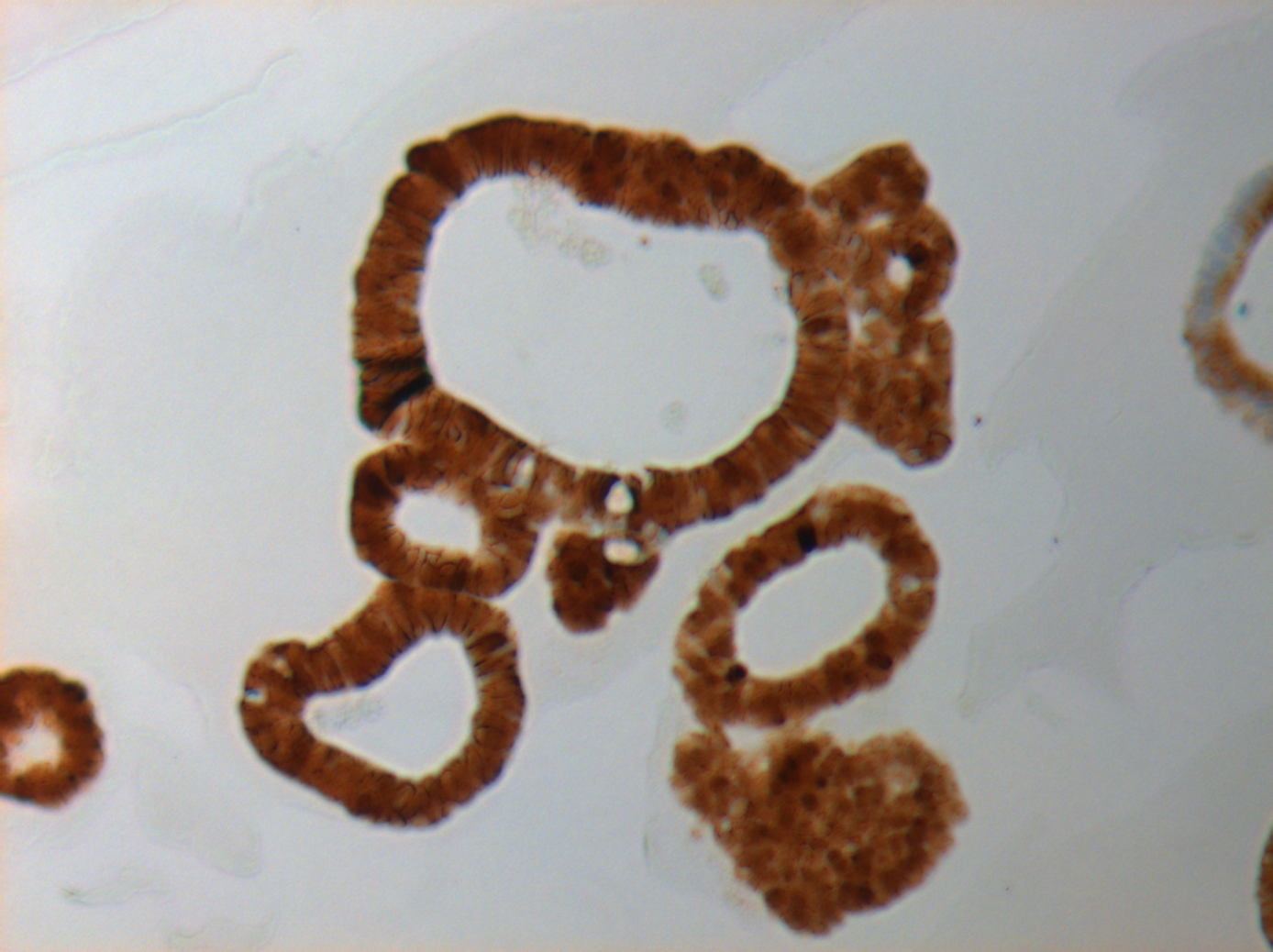

Supplement: Supplementary file 4 — Source Data [file 41467_2024_45605_MOESM4_ESM.zip › Source Data/Figures_Source_Data/supplemental figure 2/panel d/D7.tif]

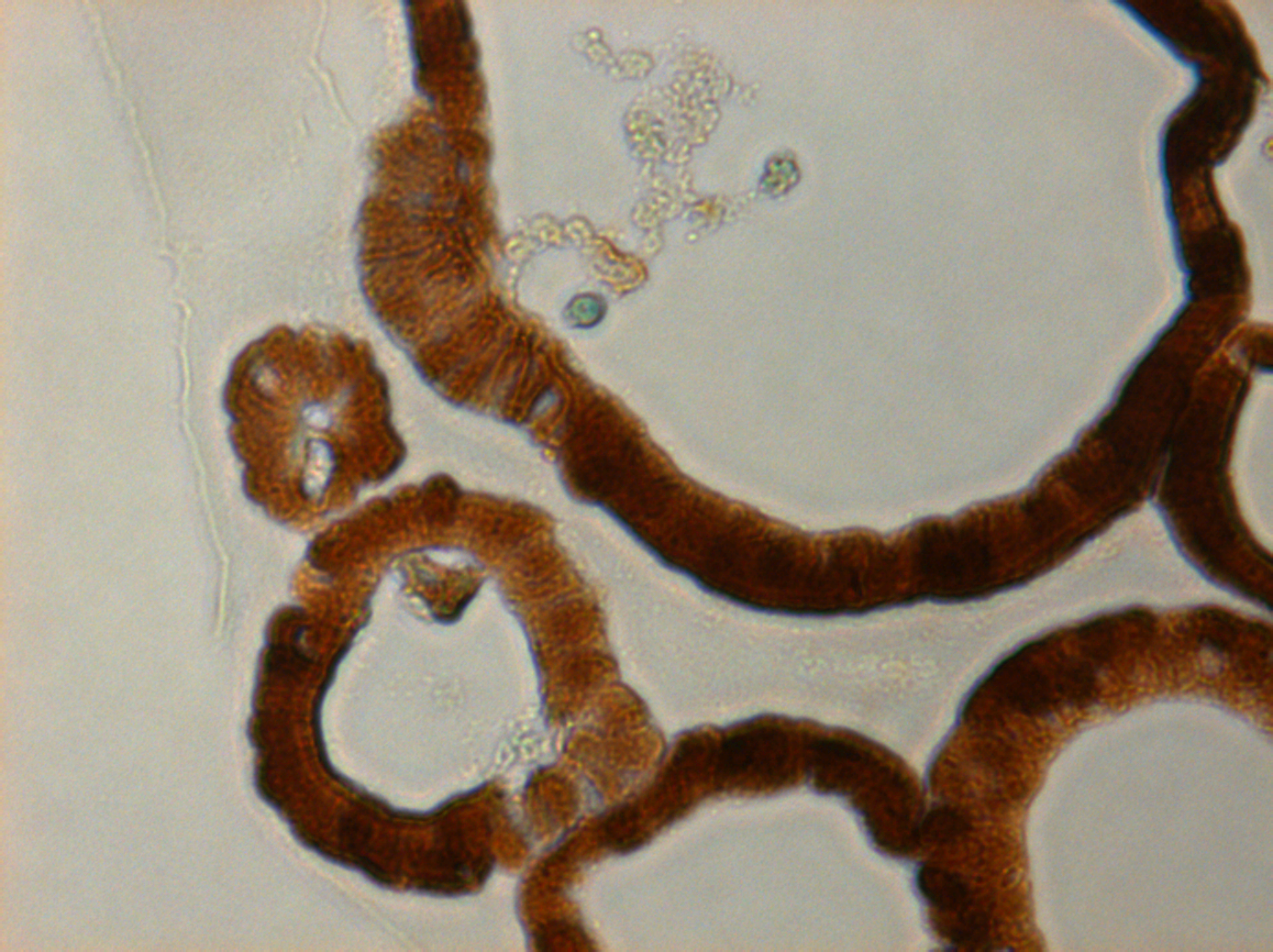

Supplement: Supplementary file 4 — Source Data [file 41467_2024_45605_MOESM4_ESM.zip › Source Data/Figures_Source_Data/supplemental figure 2/panel d/D6.tif]

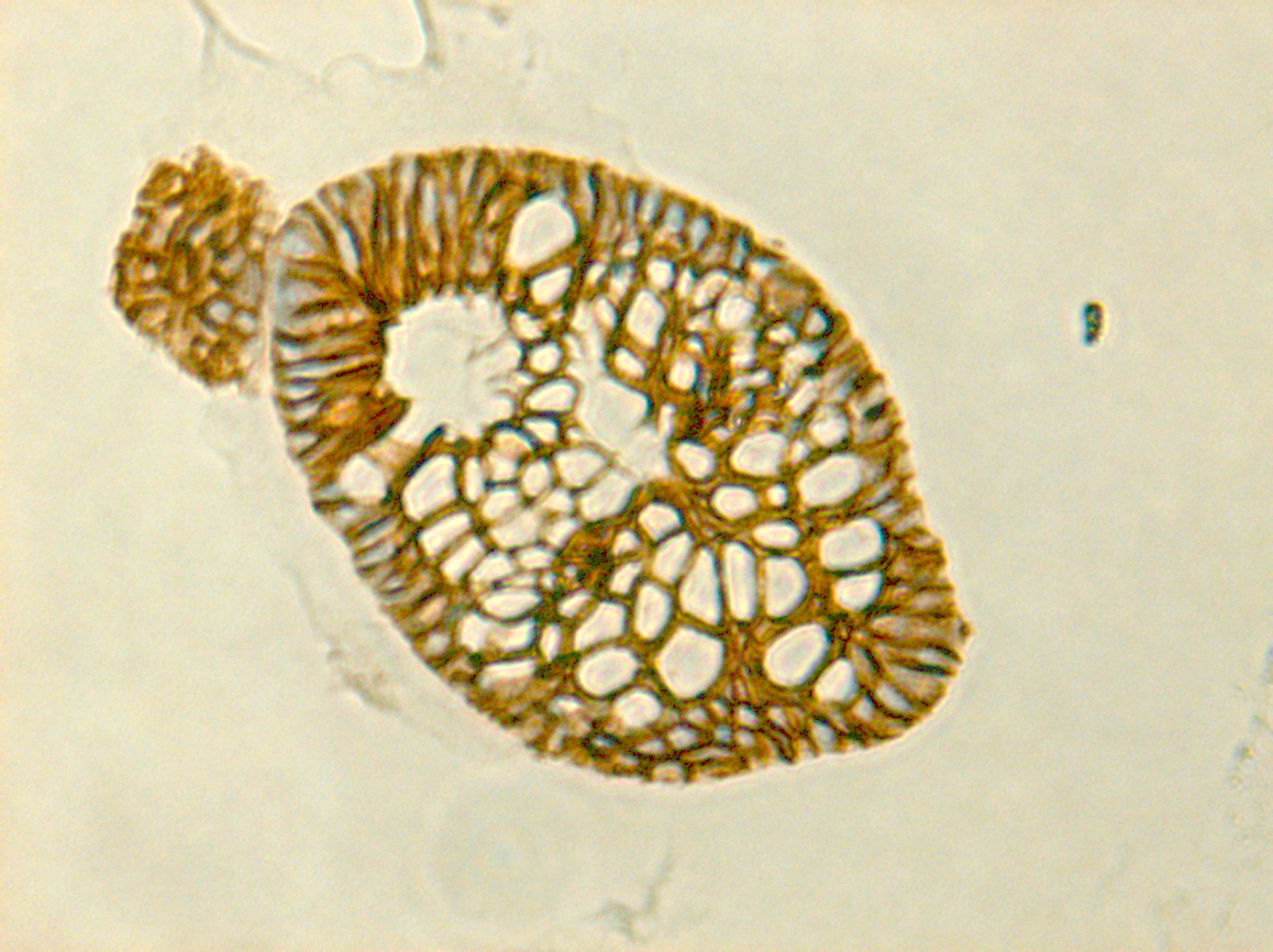

Supplement: Supplementary file 4 — Source Data [file 41467_2024_45605_MOESM4_ESM.zip › Source Data/Figures_Source_Data/supplemental figure 2/panel d/D4.tif]

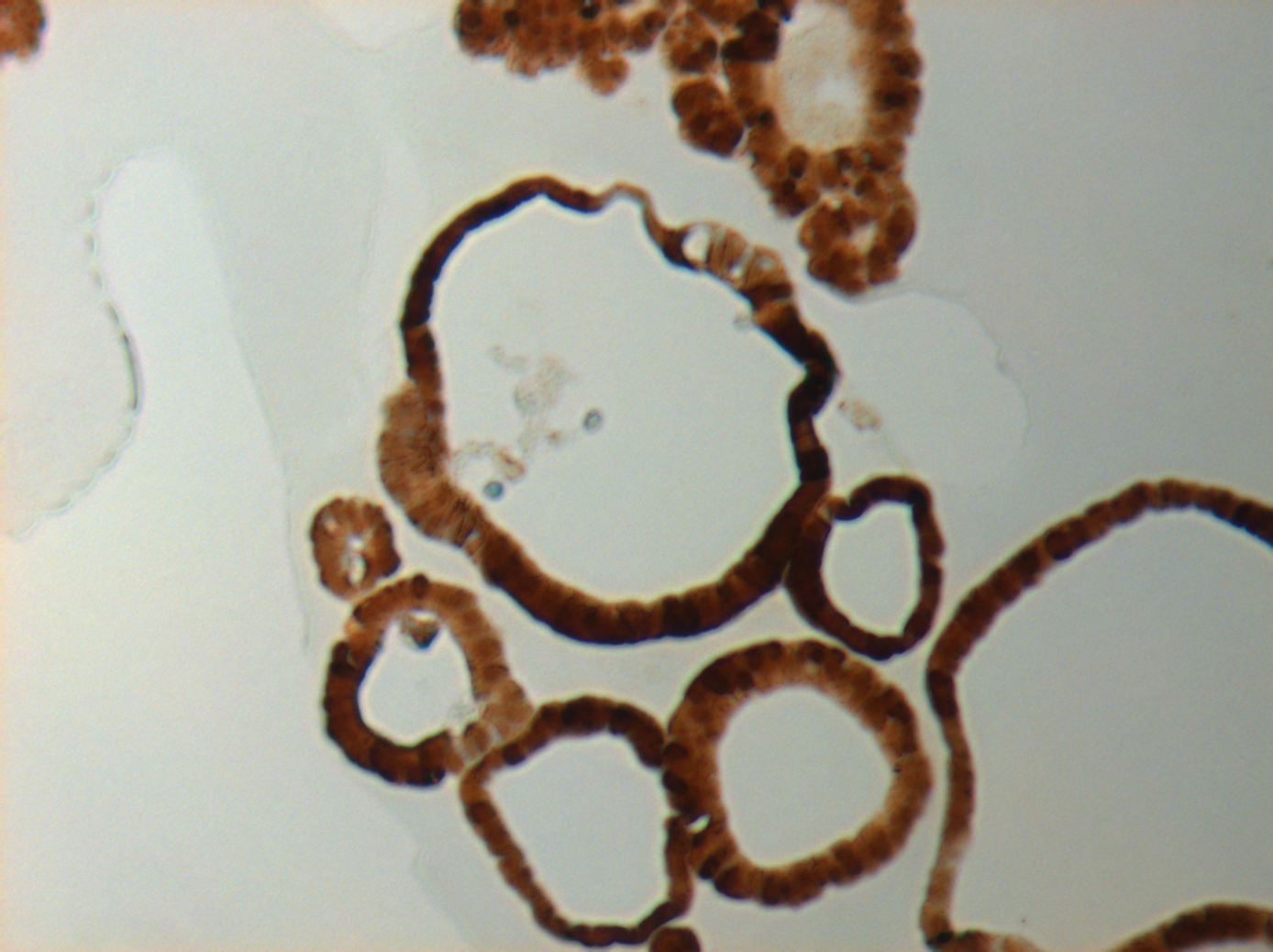

Supplement: Supplementary file 4 — Source Data [file 41467_2024_45605_MOESM4_ESM.zip › Source Data/Figures_Source_Data/supplemental figure 2/panel d/D5.tif]

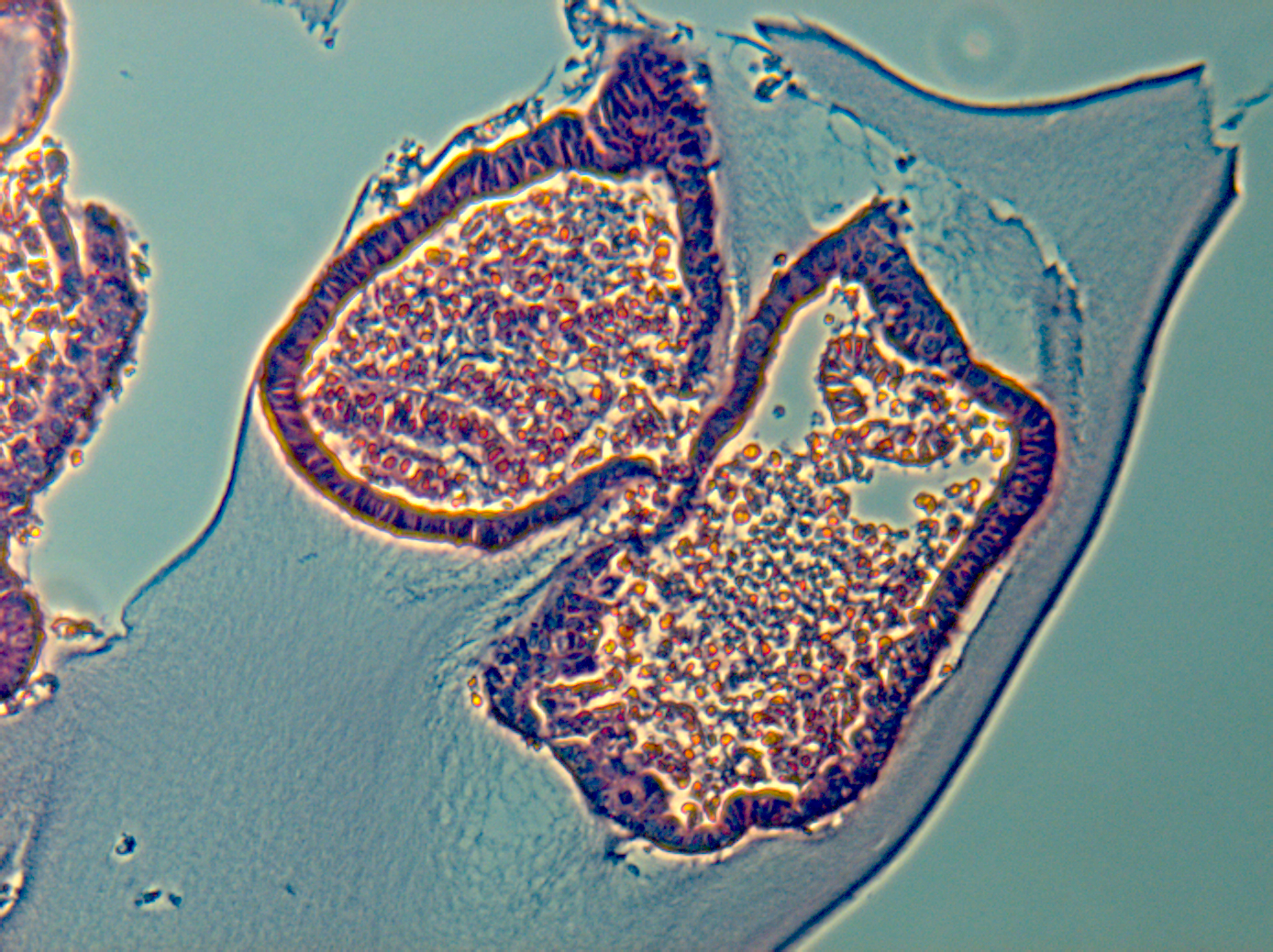

Supplement: Supplementary file 4 — Source Data [file 41467_2024_45605_MOESM4_ESM.zip › Source Data/Figures_Source_Data/supplemental figure 2/panel c/C9.tif]

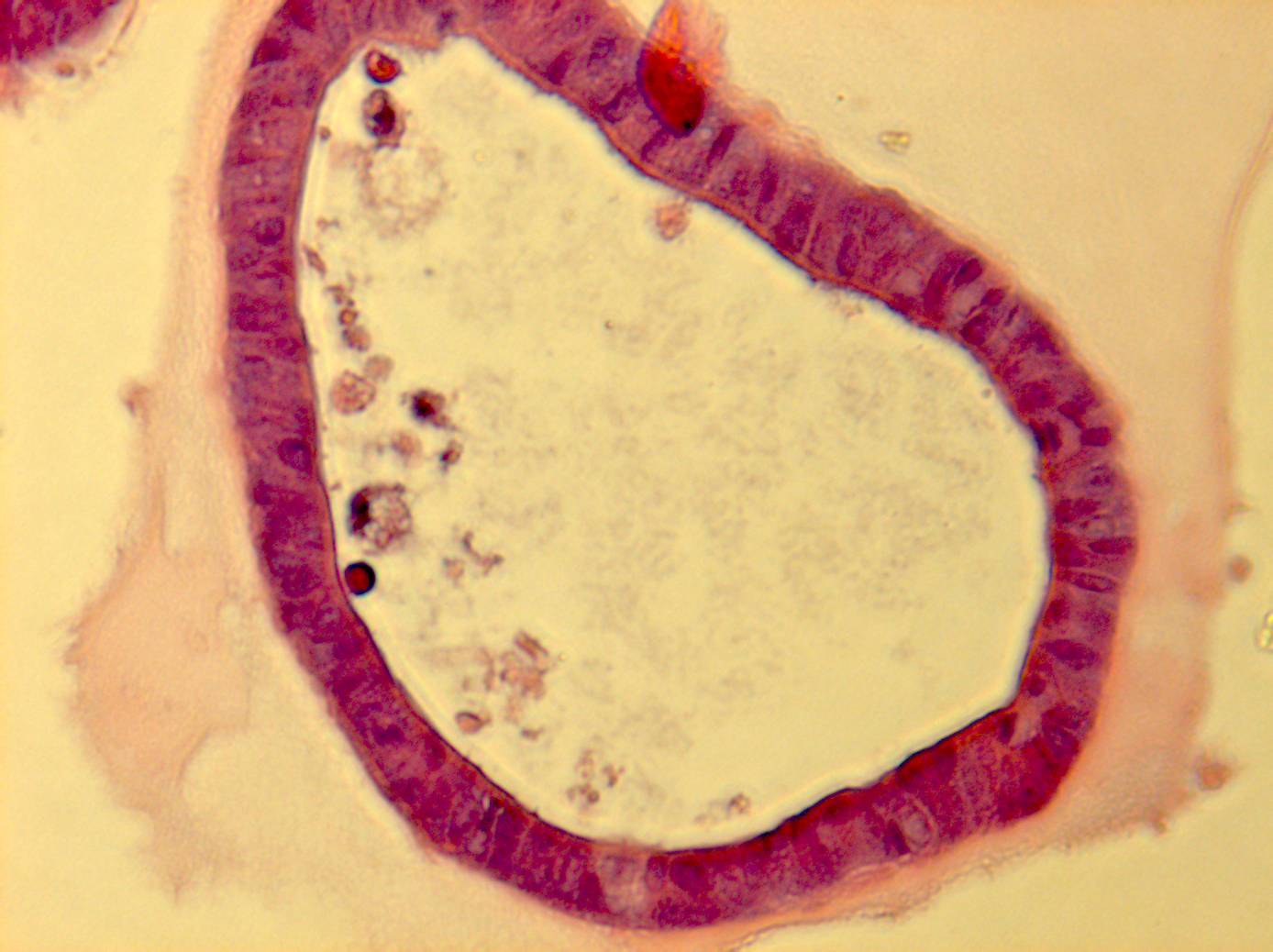

Supplement: Supplementary file 4 — Source Data [file 41467_2024_45605_MOESM4_ESM.zip › Source Data/Figures_Source_Data/supplemental figure 2/panel c/C8.tif]

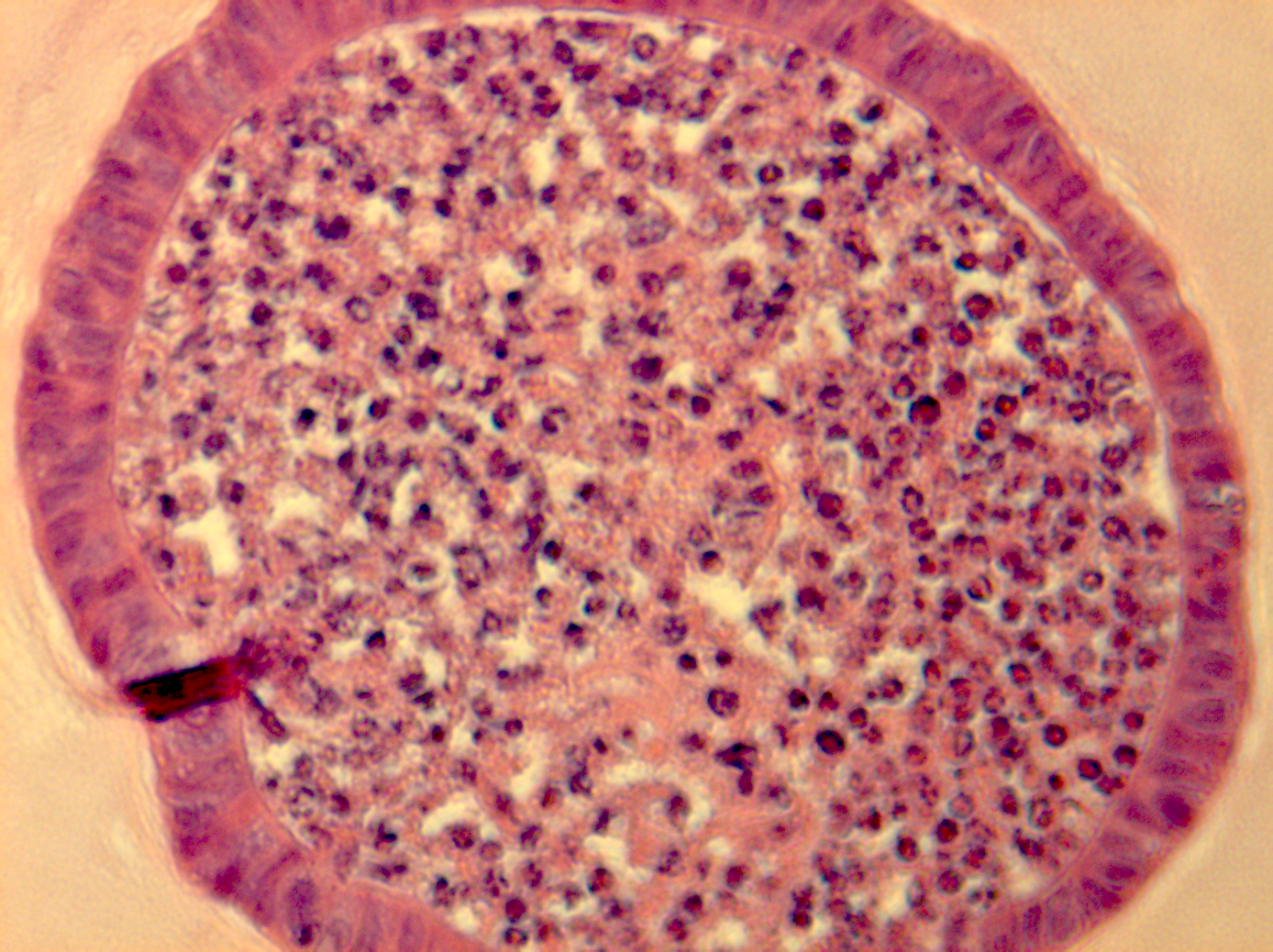

Supplement: Supplementary file 4 — Source Data [file 41467_2024_45605_MOESM4_ESM.zip › Source Data/Figures_Source_Data/supplemental figure 2/panel c/C6.tif]

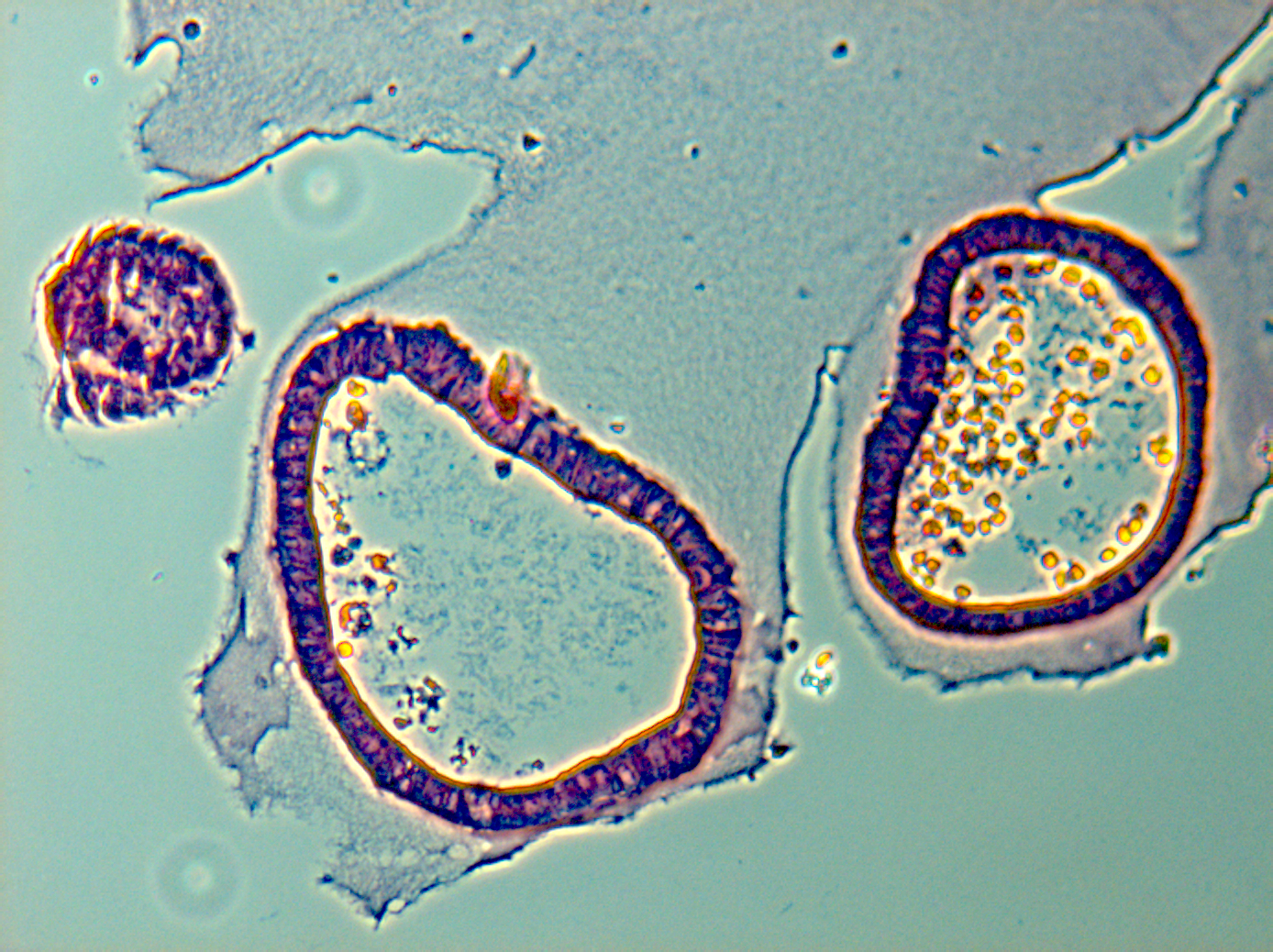

Supplement: Supplementary file 4 — Source Data [file 41467_2024_45605_MOESM4_ESM.zip › Source Data/Figures_Source_Data/supplemental figure 2/panel c/C7.tif]

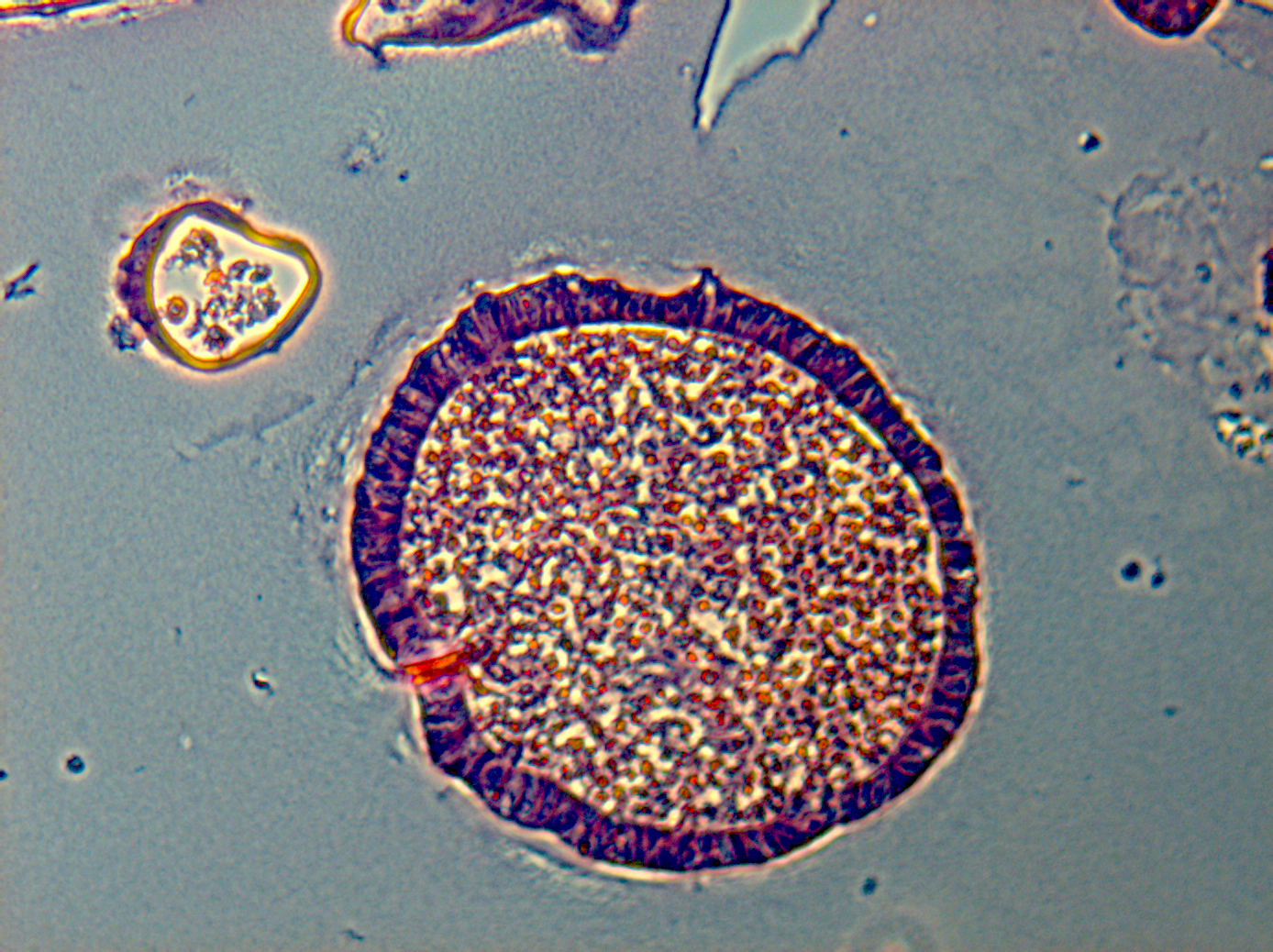

Supplement: Supplementary file 4 — Source Data [file 41467_2024_45605_MOESM4_ESM.zip › Source Data/Figures_Source_Data/supplemental figure 2/panel c/C5.tif]

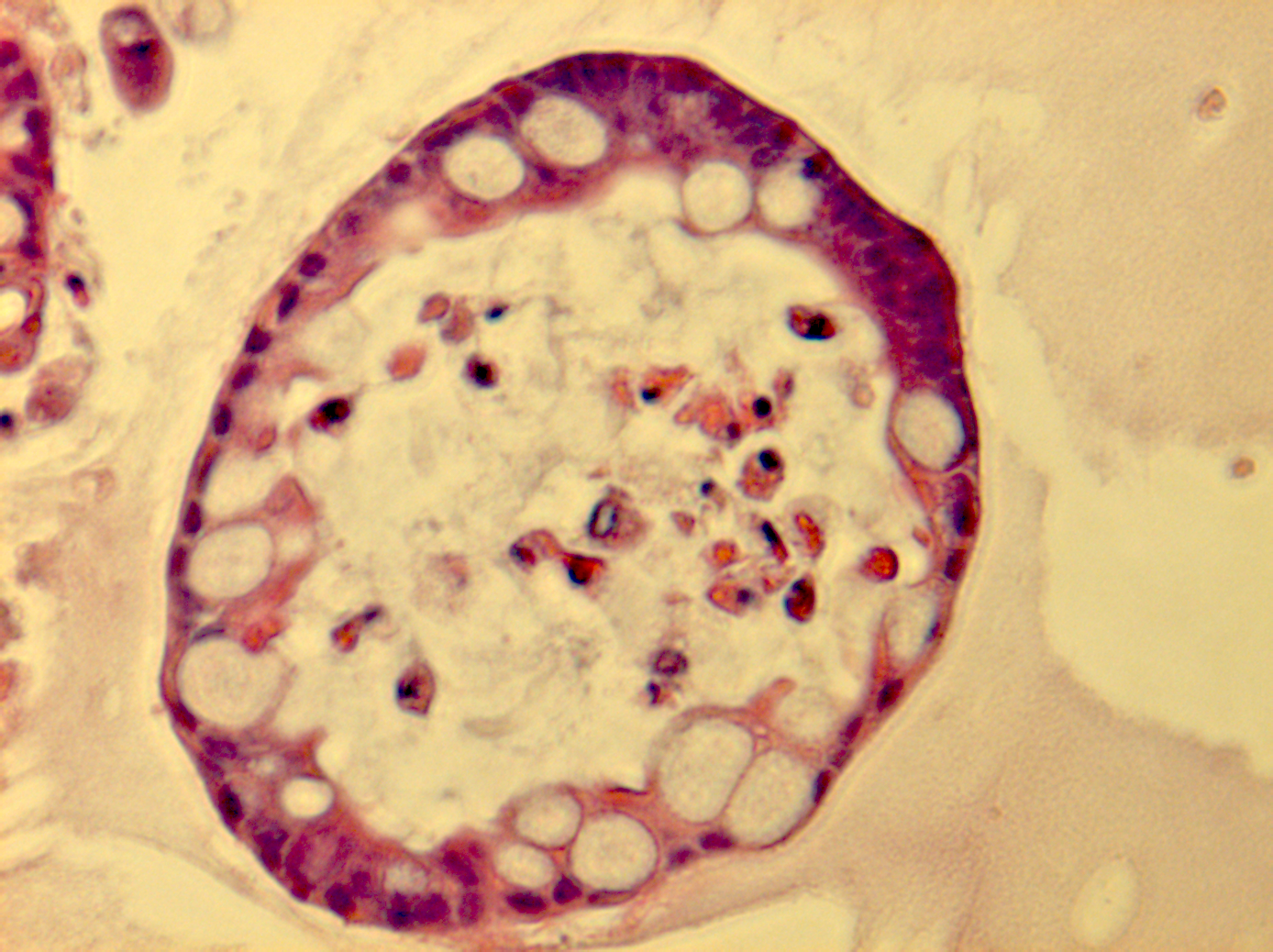

Supplement: Supplementary file 4 — Source Data [file 41467_2024_45605_MOESM4_ESM.zip › Source Data/Figures_Source_Data/supplemental figure 2/panel c/C4.tif]

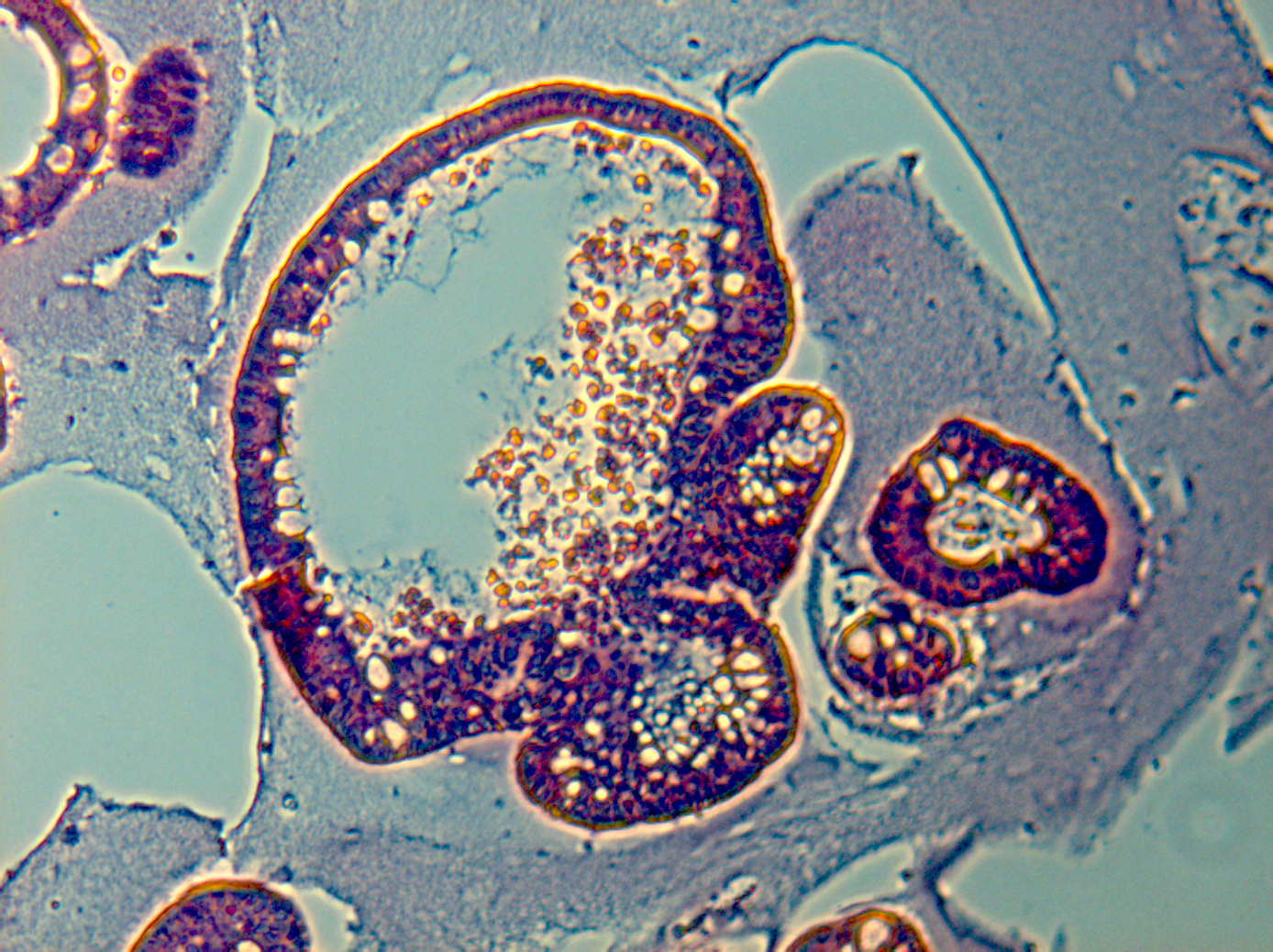

Supplement: Supplementary file 4 — Source Data [file 41467_2024_45605_MOESM4_ESM.zip › Source Data/Figures_Source_Data/supplemental figure 2/panel c/C1.tif]

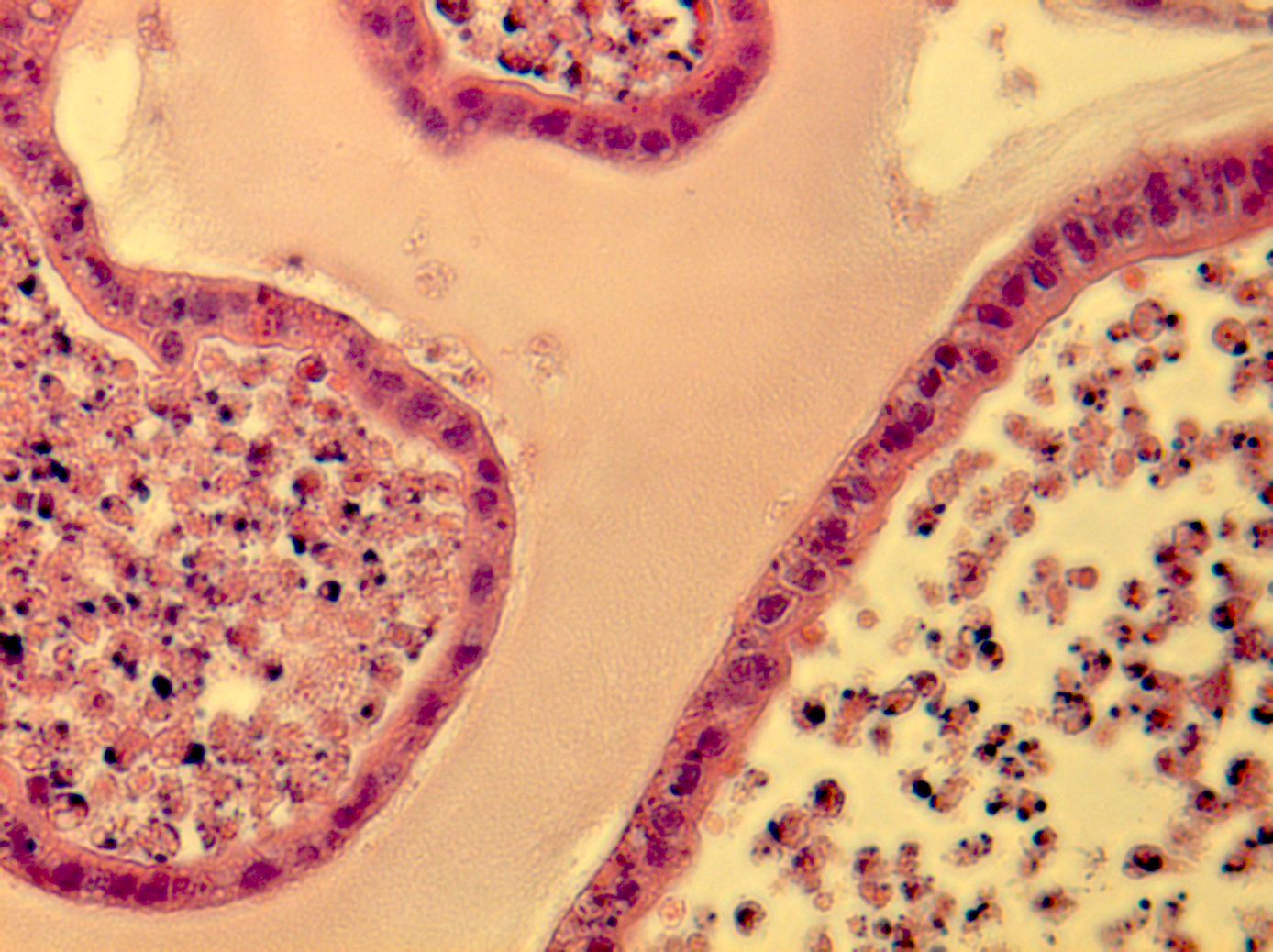

Supplement: Supplementary file 4 — Source Data [file 41467_2024_45605_MOESM4_ESM.zip › Source Data/Figures_Source_Data/supplemental figure 2/panel c/C12.tif]

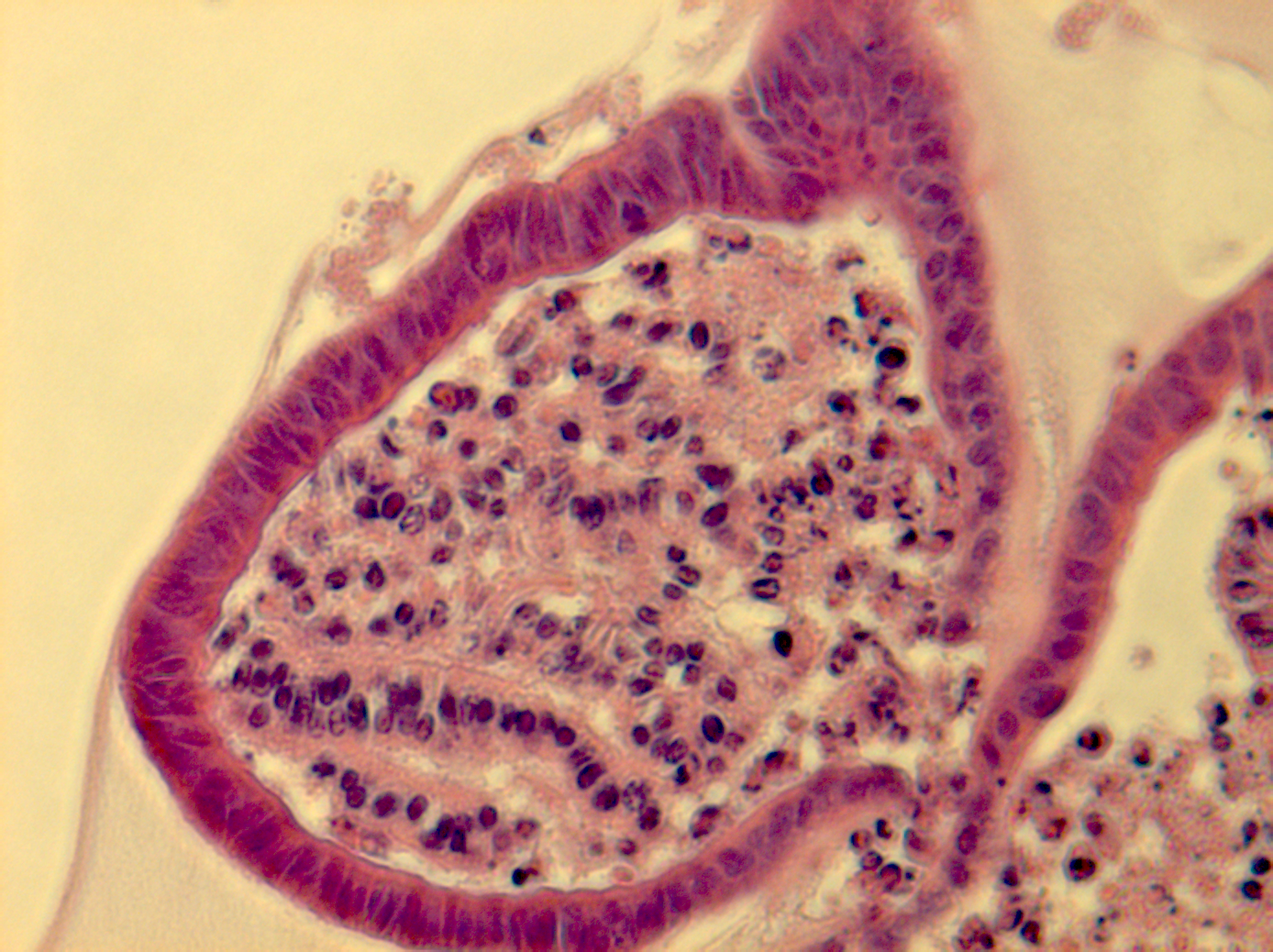

Supplement: Supplementary file 4 — Source Data [file 41467_2024_45605_MOESM4_ESM.zip › Source Data/Figures_Source_Data/supplemental figure 2/panel c/C10.tif]

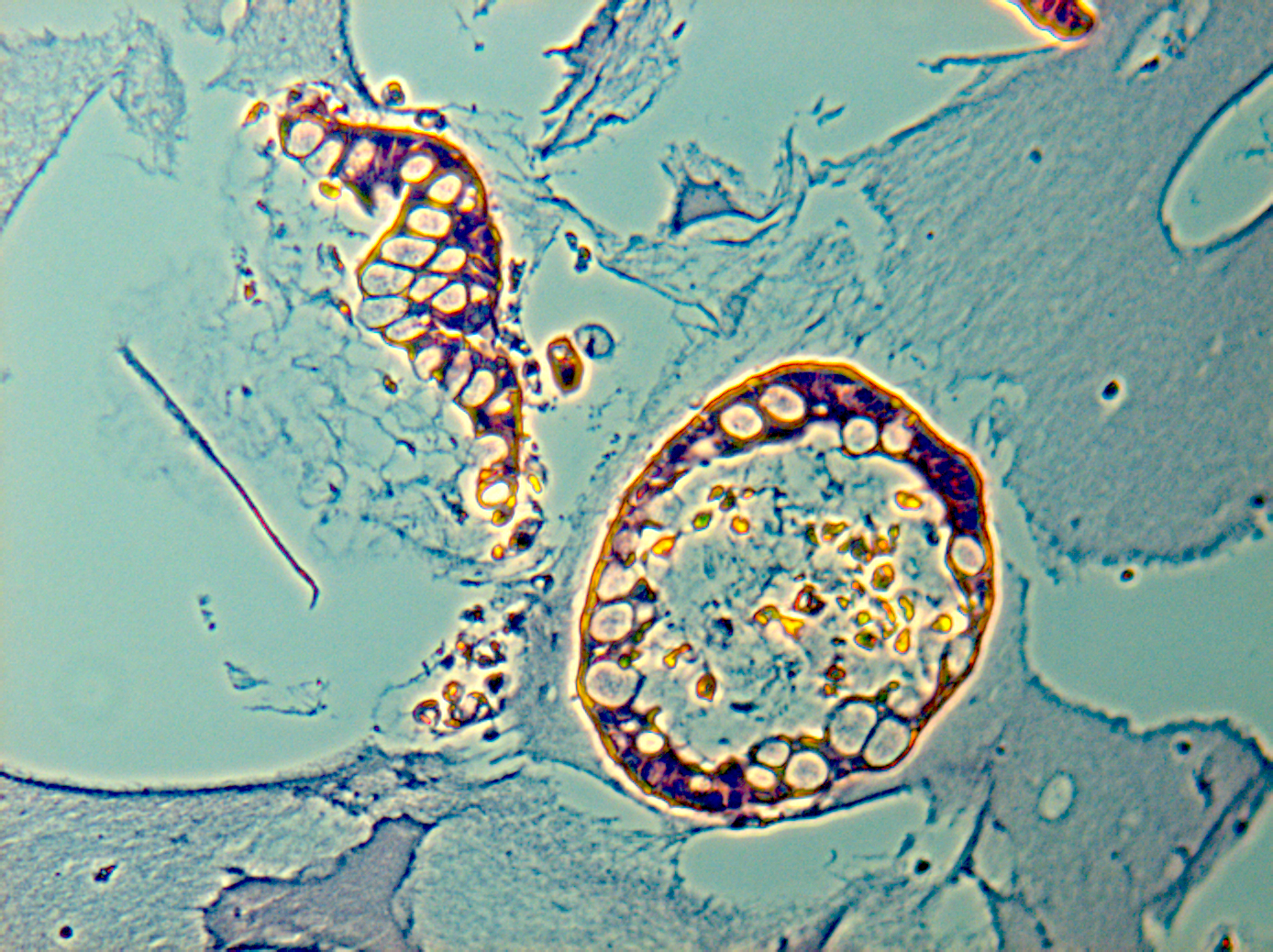

Supplement: Supplementary file 4 — Source Data [file 41467_2024_45605_MOESM4_ESM.zip › Source Data/Figures_Source_Data/supplemental figure 2/panel c/C3.tif]

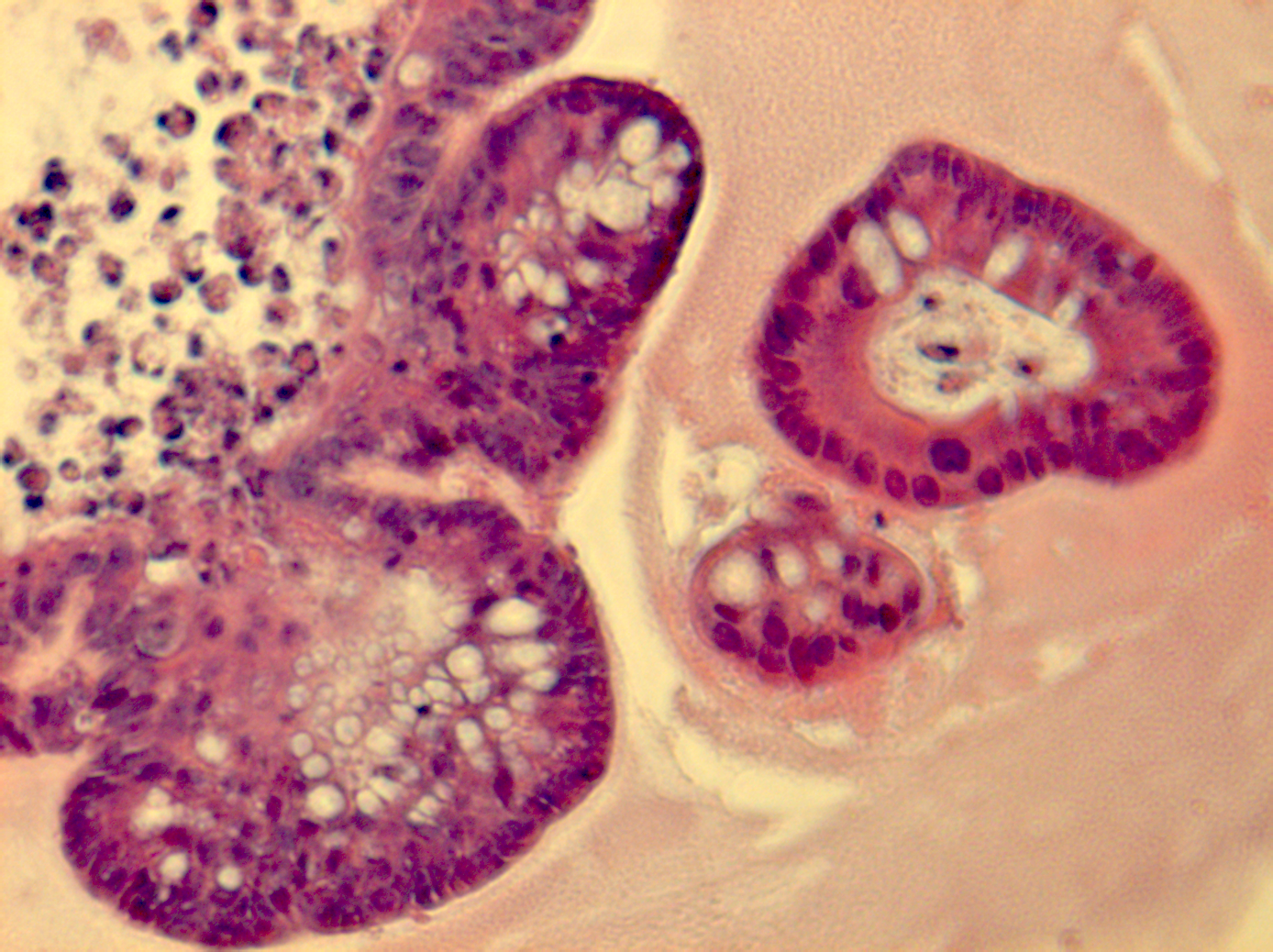

Supplement: Supplementary file 4 — Source Data [file 41467_2024_45605_MOESM4_ESM.zip › Source Data/Figures_Source_Data/supplemental figure 2/panel c/C2.tif]

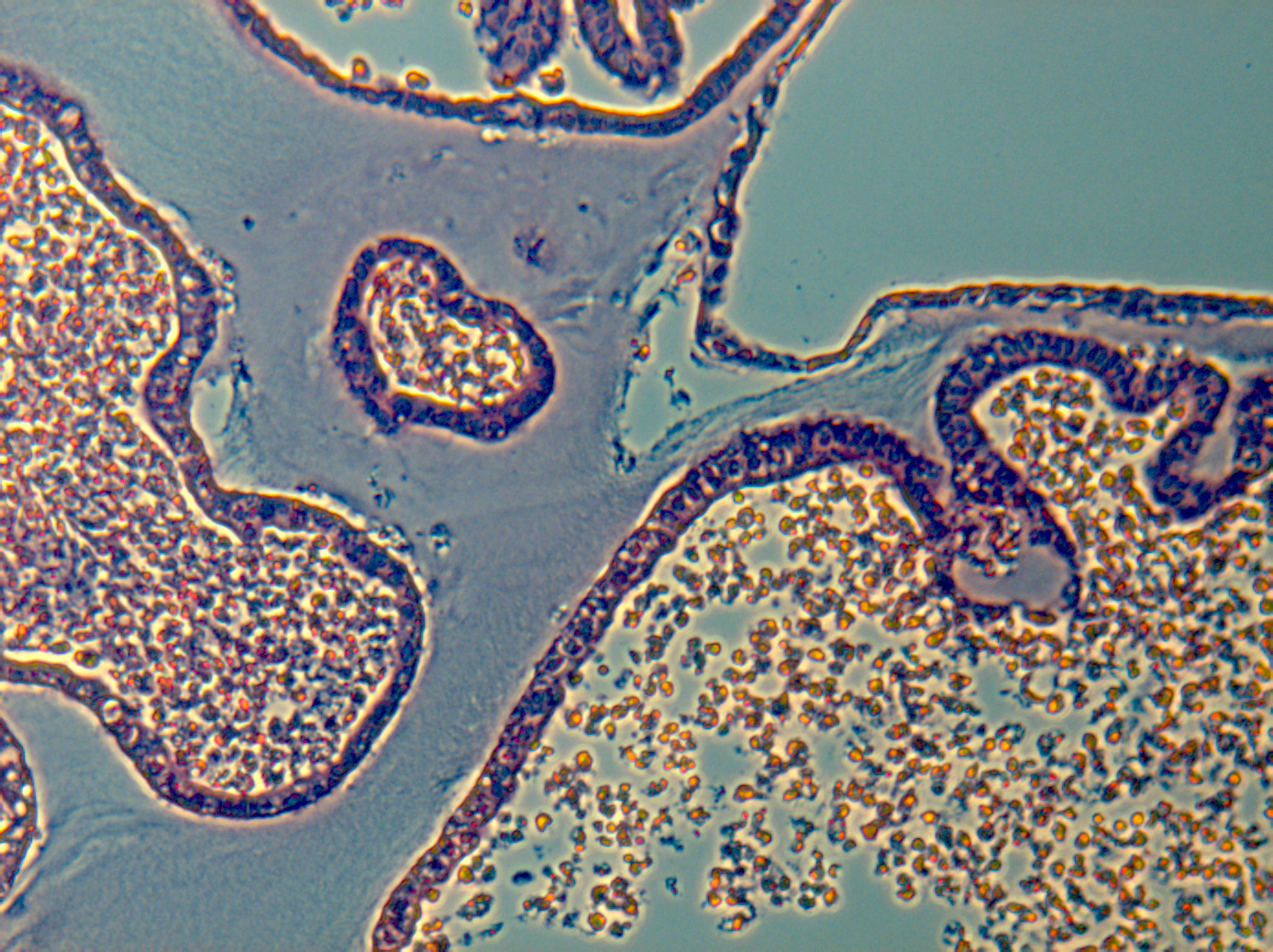

Supplement: Supplementary file 4 — Source Data [file 41467_2024_45605_MOESM4_ESM.zip › Source Data/Figures_Source_Data/supplemental figure 2/panel c/C11.tif]

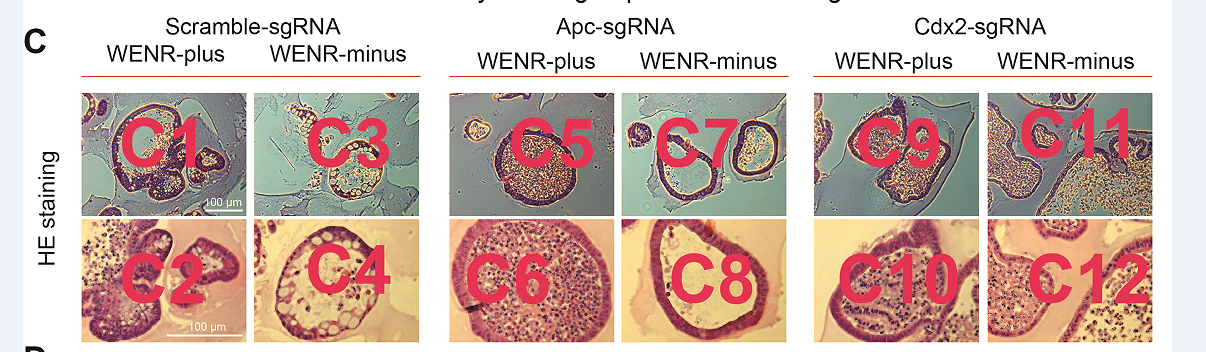

Supplement: Supplementary file 4 — Source Data [file 41467_2024_45605_MOESM4_ESM.zip › Source Data/Figures_Source_Data/supplemental figure 2/panel c/panel c.png]

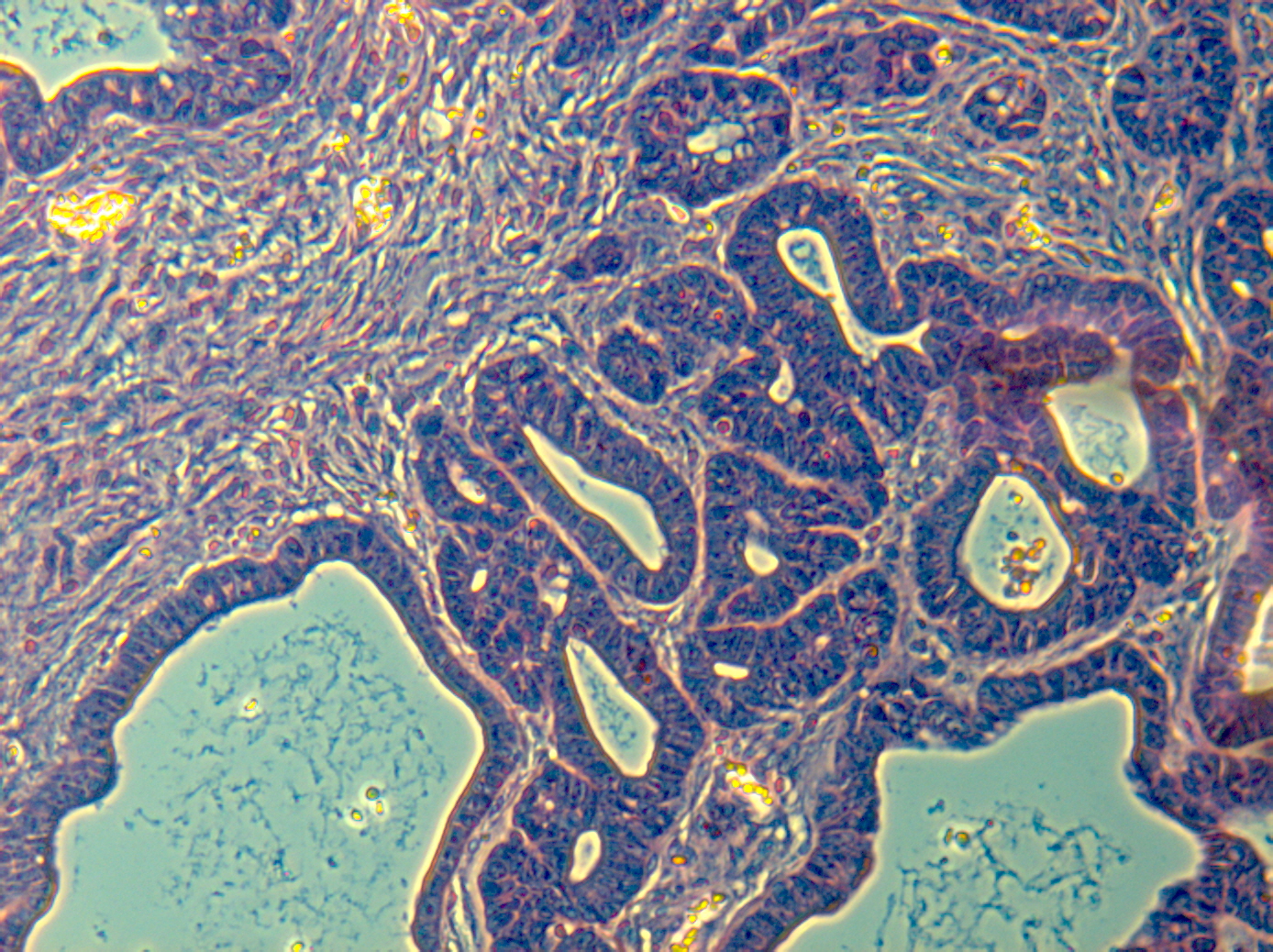

Supplement: Supplementary file 4 — Source Data [file 41467_2024_45605_MOESM4_ESM.zip › Source Data/Figures_Source_Data/supplemental figure 2/panel a/a1.jpg]

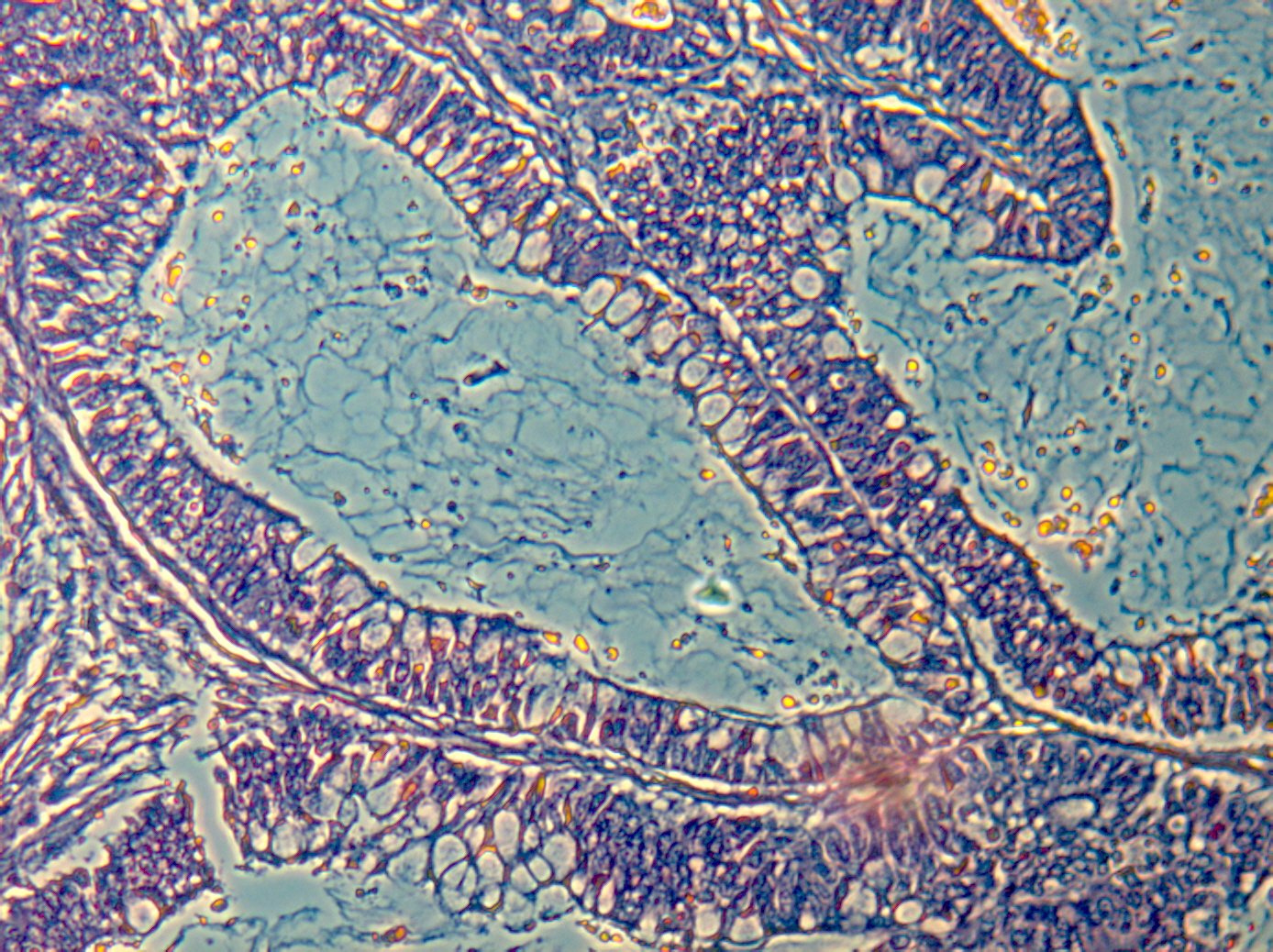

Supplement: Supplementary file 4 — Source Data [file 41467_2024_45605_MOESM4_ESM.zip › Source Data/Figures_Source_Data/supplemental figure 2/panel a/a3.jpg]

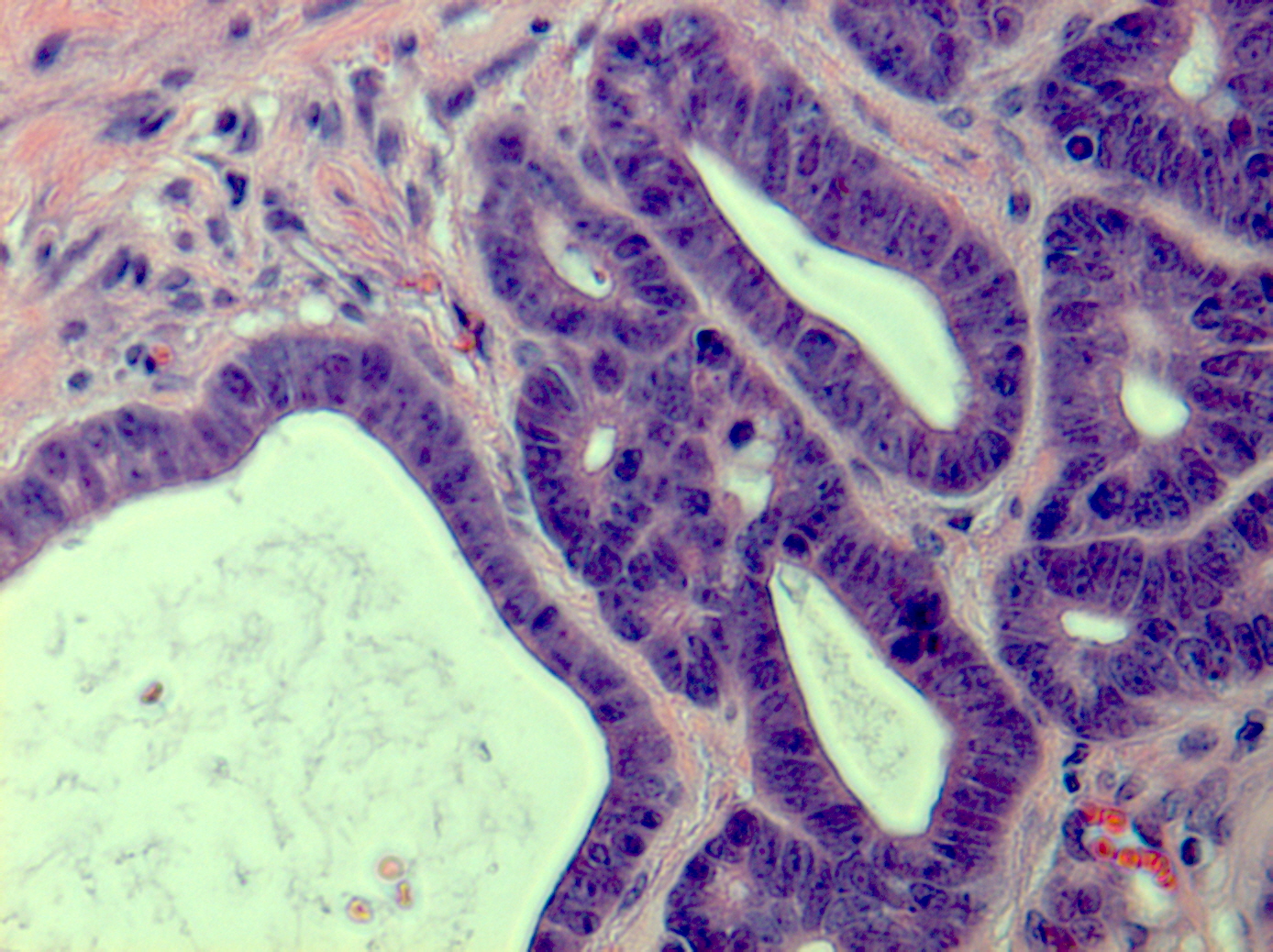

Supplement: Supplementary file 4 — Source Data [file 41467_2024_45605_MOESM4_ESM.zip › Source Data/Figures_Source_Data/supplemental figure 2/panel a/a2.jpg]

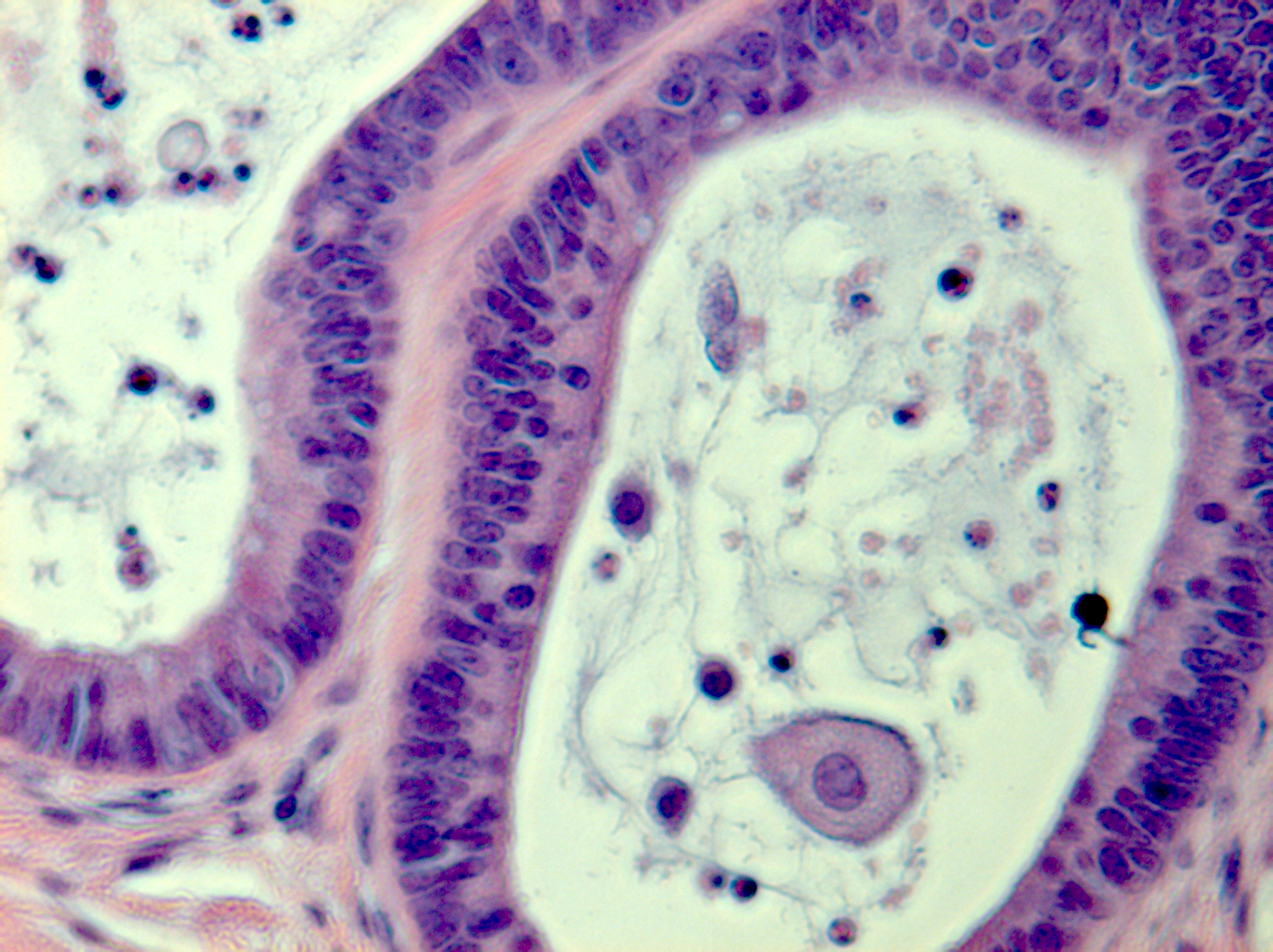

Supplement: Supplementary file 4 — Source Data [file 41467_2024_45605_MOESM4_ESM.zip › Source Data/Figures_Source_Data/supplemental figure 2/panel a/a6.jpg]

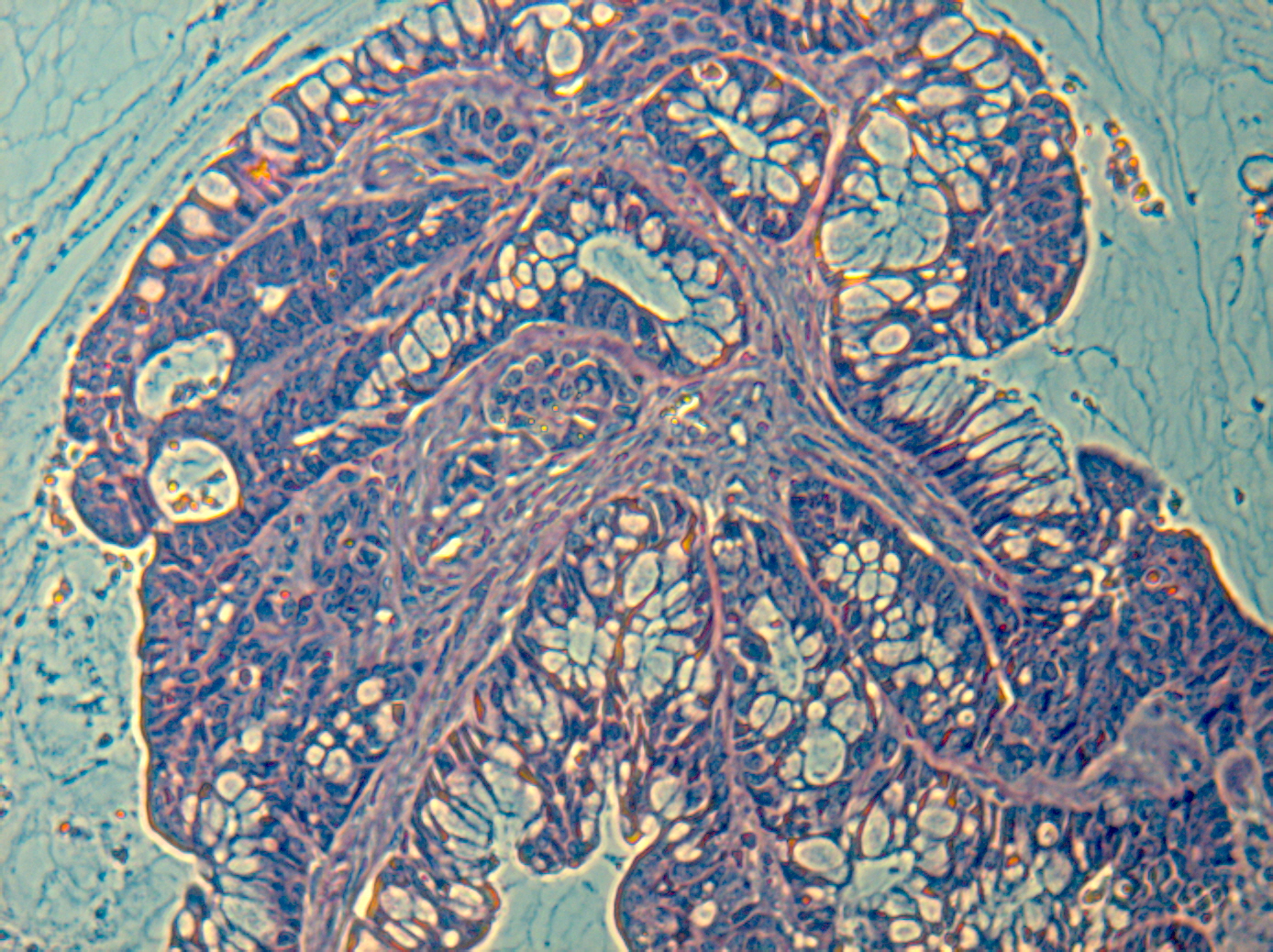

Supplement: Supplementary file 4 — Source Data [file 41467_2024_45605_MOESM4_ESM.zip › Source Data/Figures_Source_Data/supplemental figure 2/panel a/a7.jpg]

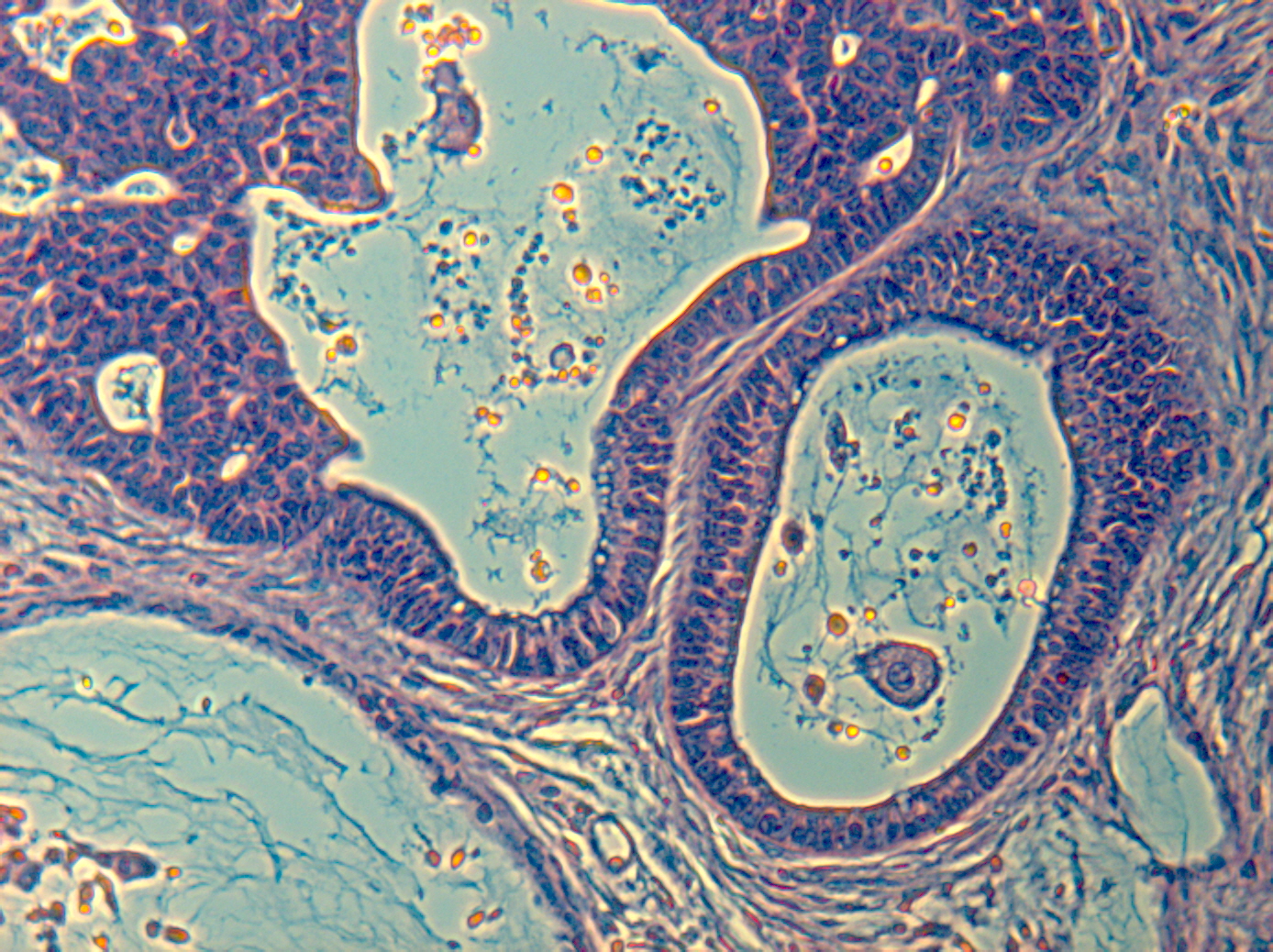

Supplement: Supplementary file 4 — Source Data [file 41467_2024_45605_MOESM4_ESM.zip › Source Data/Figures_Source_Data/supplemental figure 2/panel a/a5.jpg]

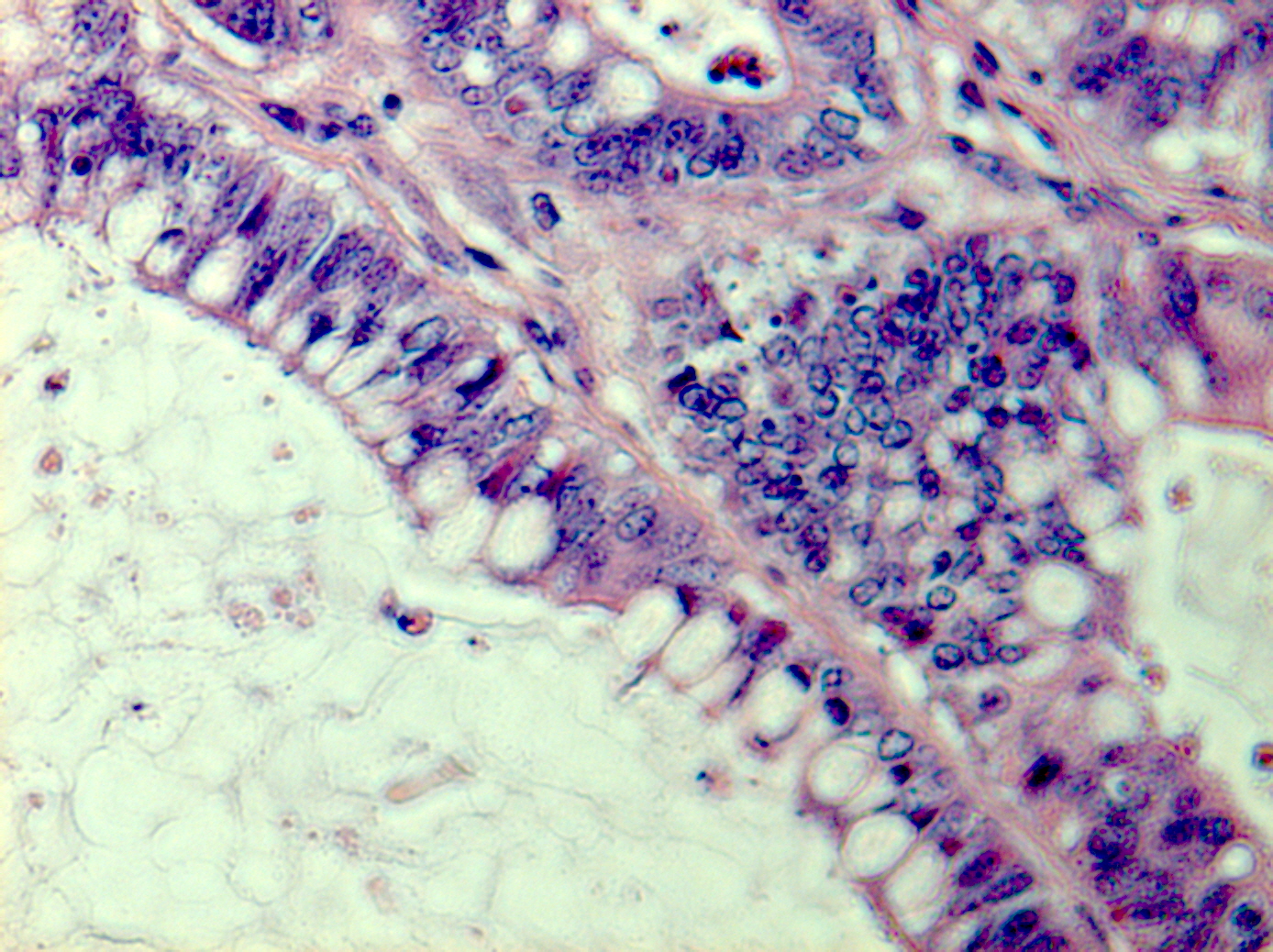

Supplement: Supplementary file 4 — Source Data [file 41467_2024_45605_MOESM4_ESM.zip › Source Data/Figures_Source_Data/supplemental figure 2/panel a/a4.jpg]

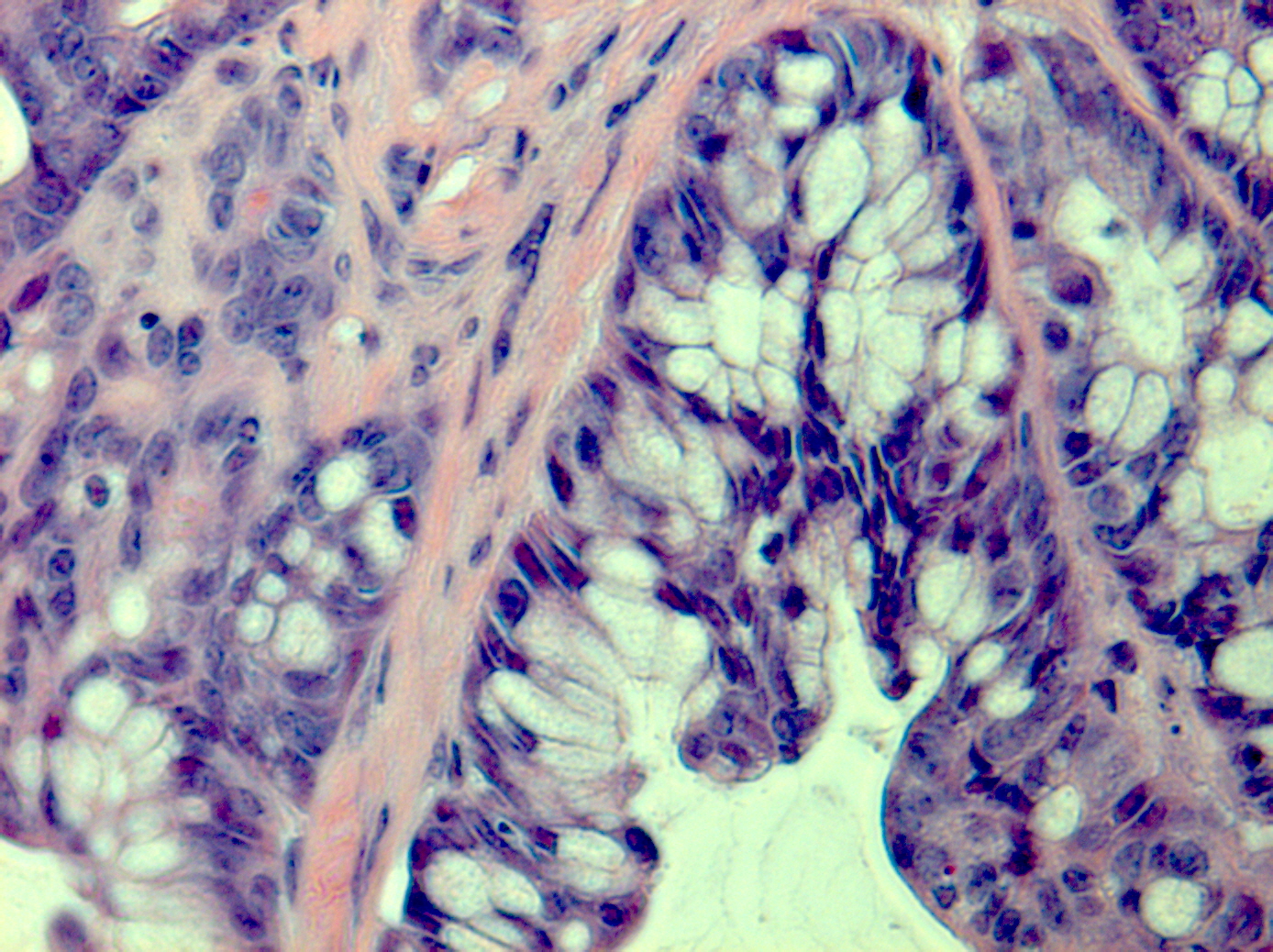

Supplement: Supplementary file 4 — Source Data [file 41467_2024_45605_MOESM4_ESM.zip › Source Data/Figures_Source_Data/supplemental figure 2/panel a/a8.jpg]

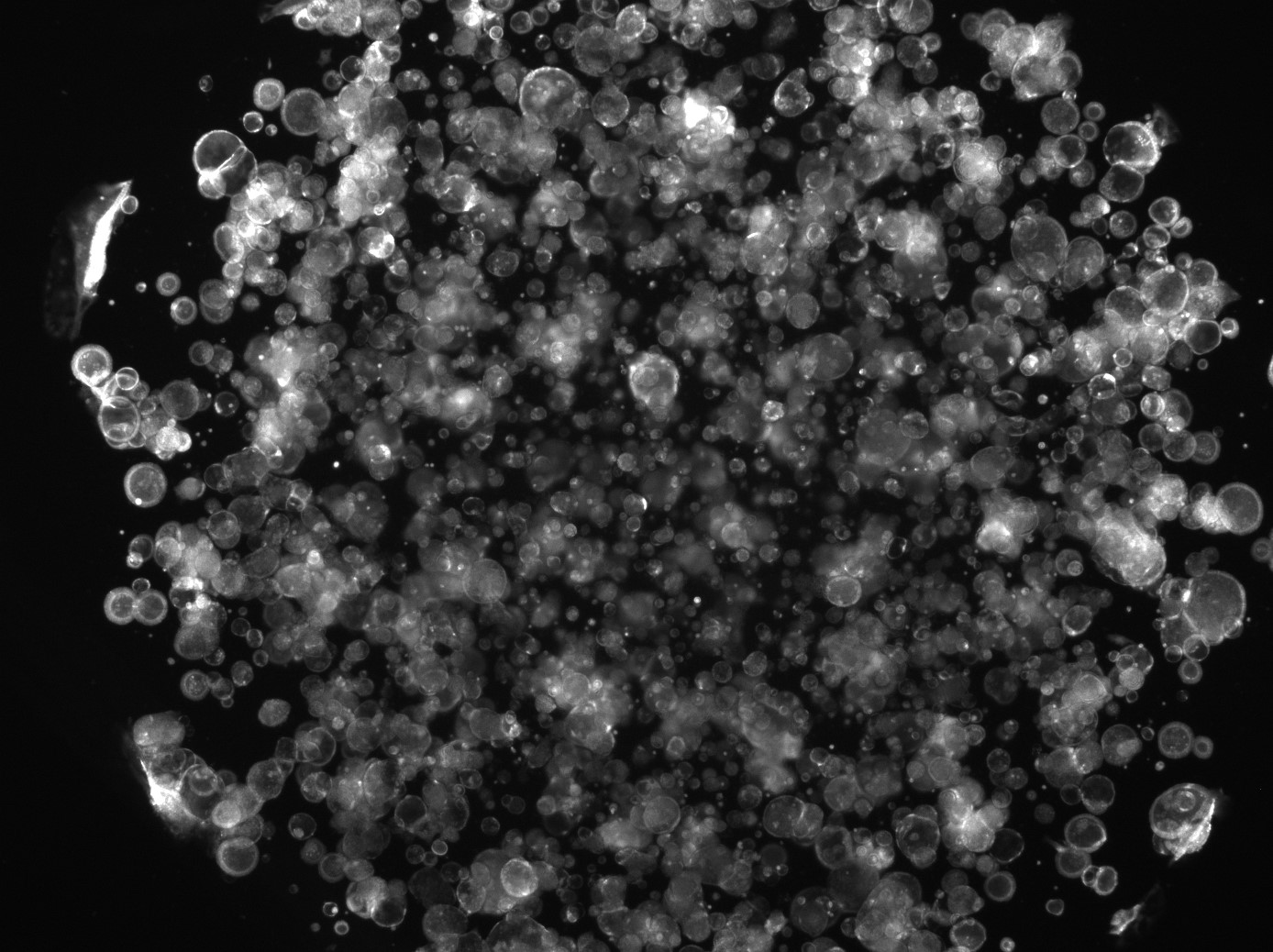

Supplement: Supplementary file 4 — Source Data [file 41467_2024_45605_MOESM4_ESM.zip › Source Data/Figures_Source_Data/figure 1/panel b/B13_TdTomato.jpg]

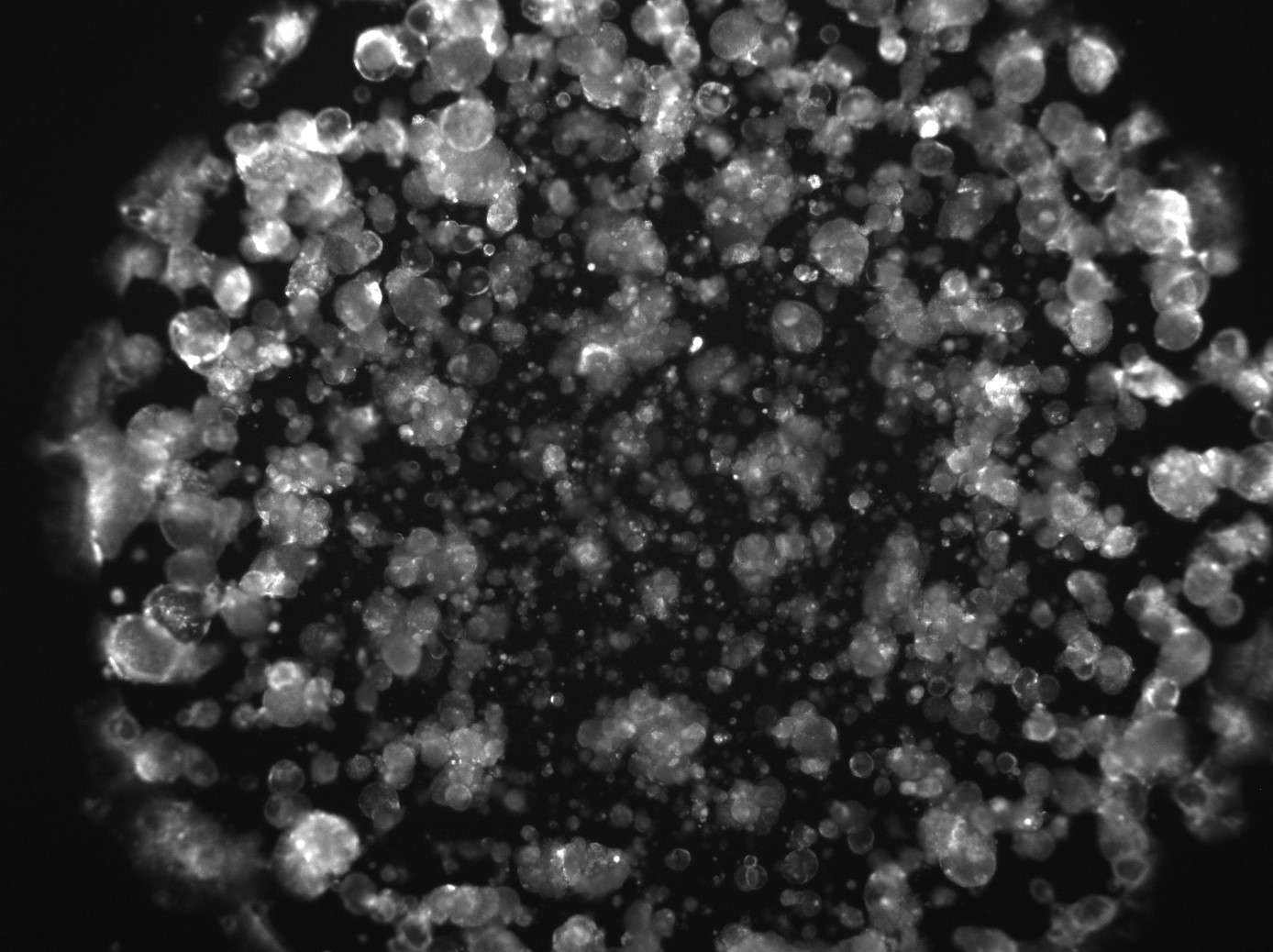

Supplement: Supplementary file 4 — Source Data [file 41467_2024_45605_MOESM4_ESM.zip › Source Data/Figures_Source_Data/figure 1/panel b/B14_TdTomato.jpg]

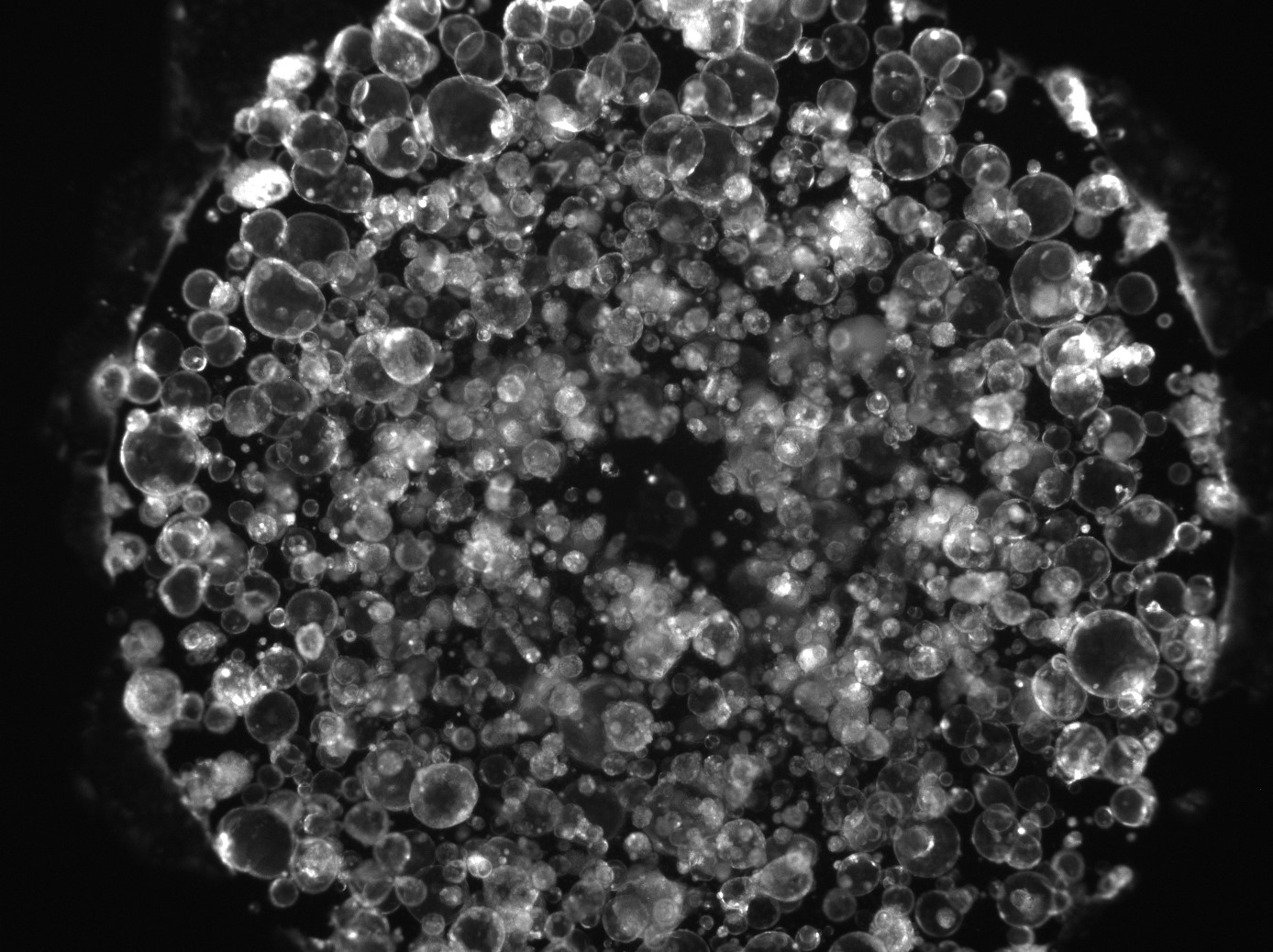

Supplement: Supplementary file 4 — Source Data [file 41467_2024_45605_MOESM4_ESM.zip › Source Data/Figures_Source_Data/figure 1/panel b/B22_Tdtomato.jpg]

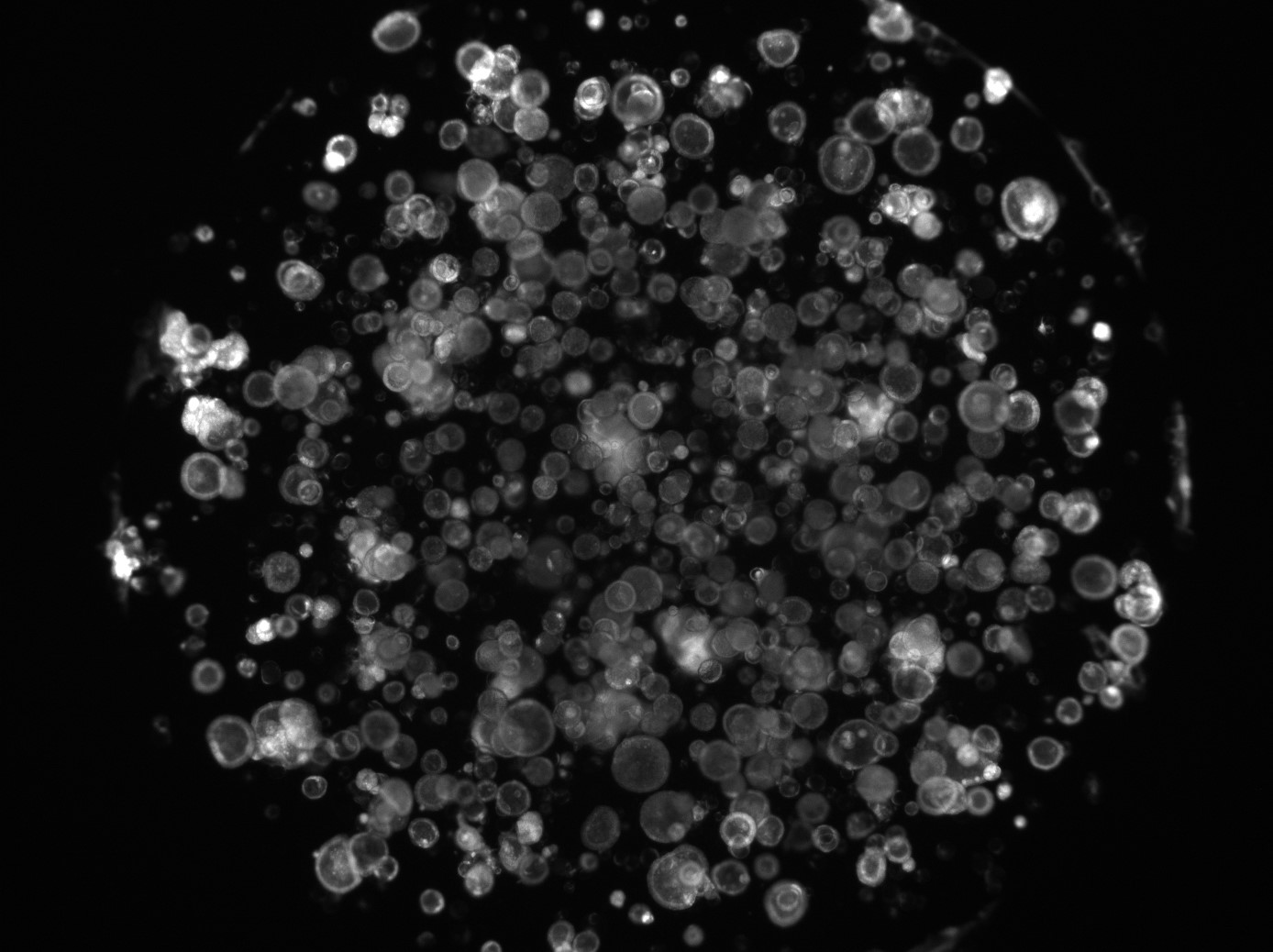

Supplement: Supplementary file 4 — Source Data [file 41467_2024_45605_MOESM4_ESM.zip › Source Data/Figures_Source_Data/figure 1/panel b/B15_TdTomato.jpg]

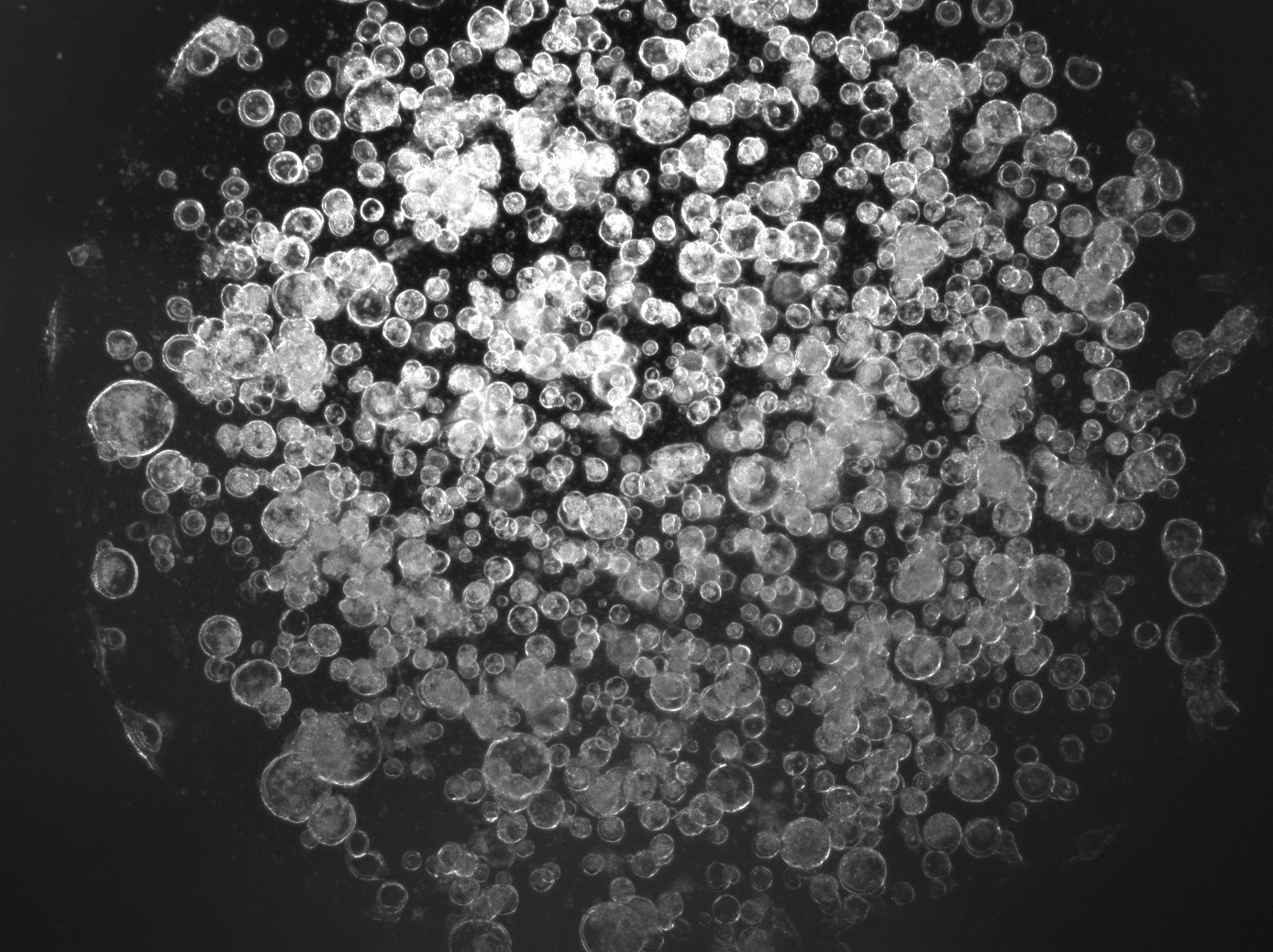

Supplement: Supplementary file 4 — Source Data [file 41467_2024_45605_MOESM4_ESM.zip › Source Data/Figures_Source_Data/figure 1/panel b/B9.jpg]

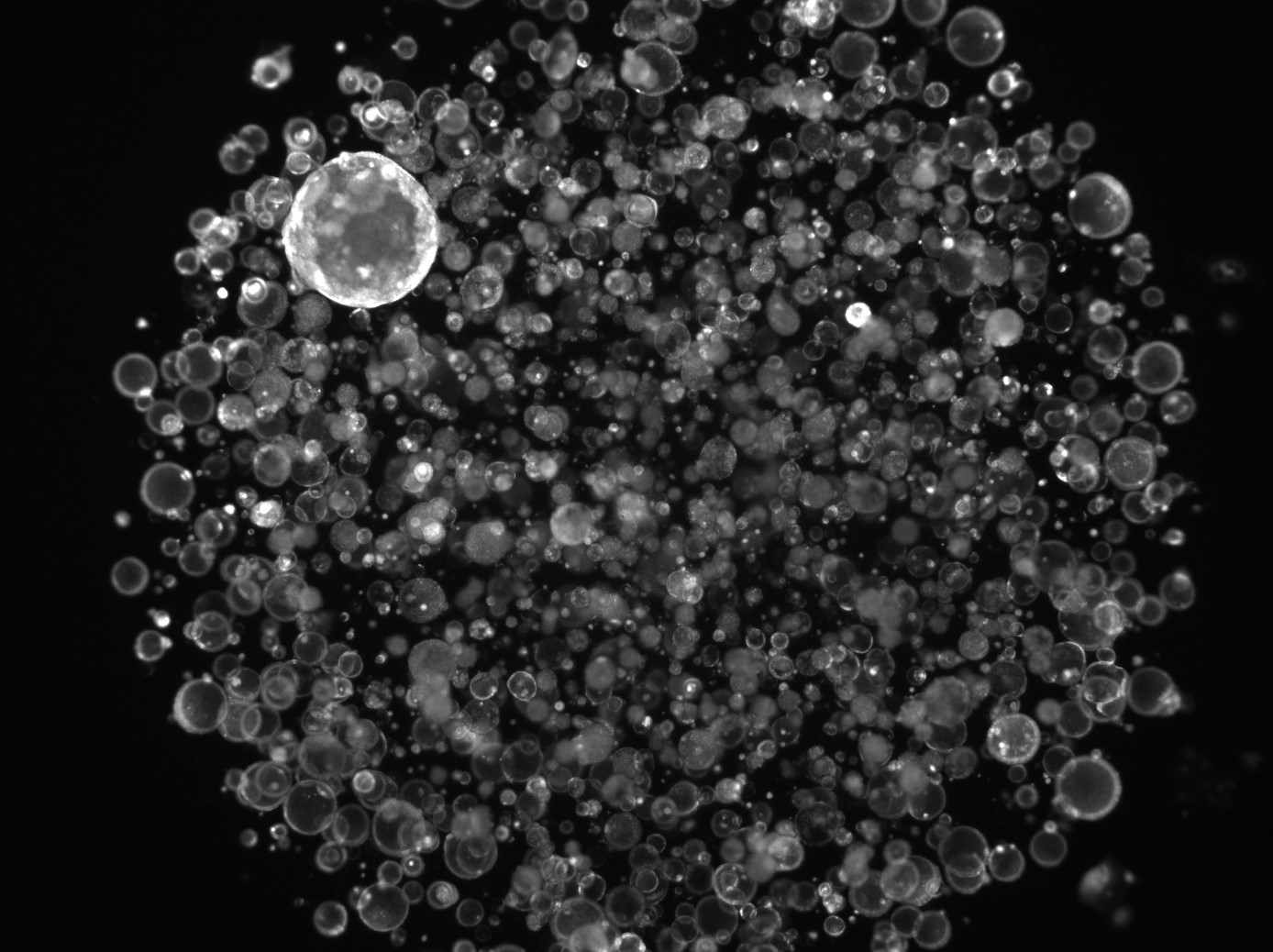

Supplement: Supplementary file 4 — Source Data [file 41467_2024_45605_MOESM4_ESM.zip › Source Data/Figures_Source_Data/figure 1/panel b/B24_TdTomato.jpg]

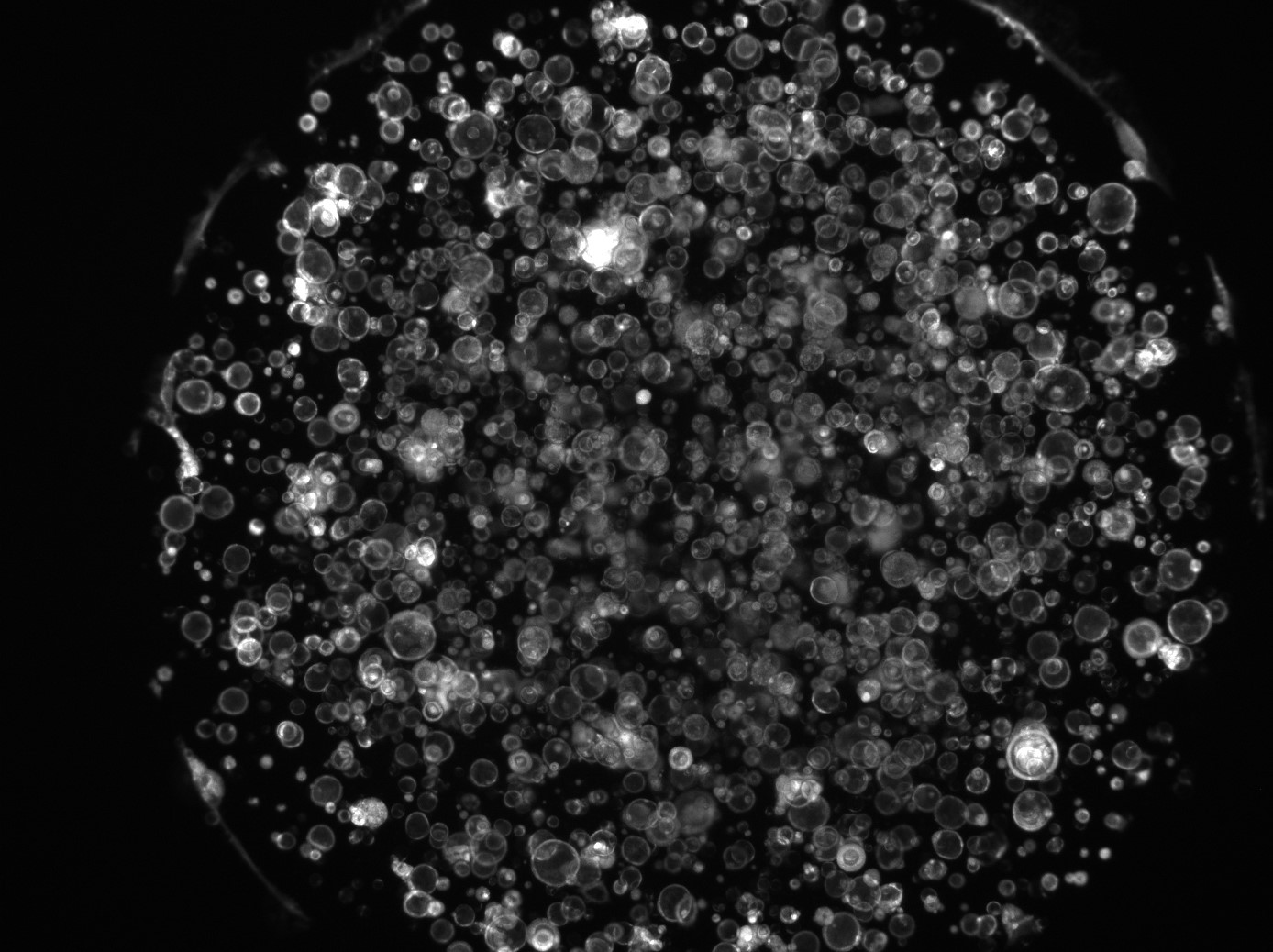

Supplement: Supplementary file 4 — Source Data [file 41467_2024_45605_MOESM4_ESM.zip › Source Data/Figures_Source_Data/figure 1/panel b/B23_TdTomato.jpg]

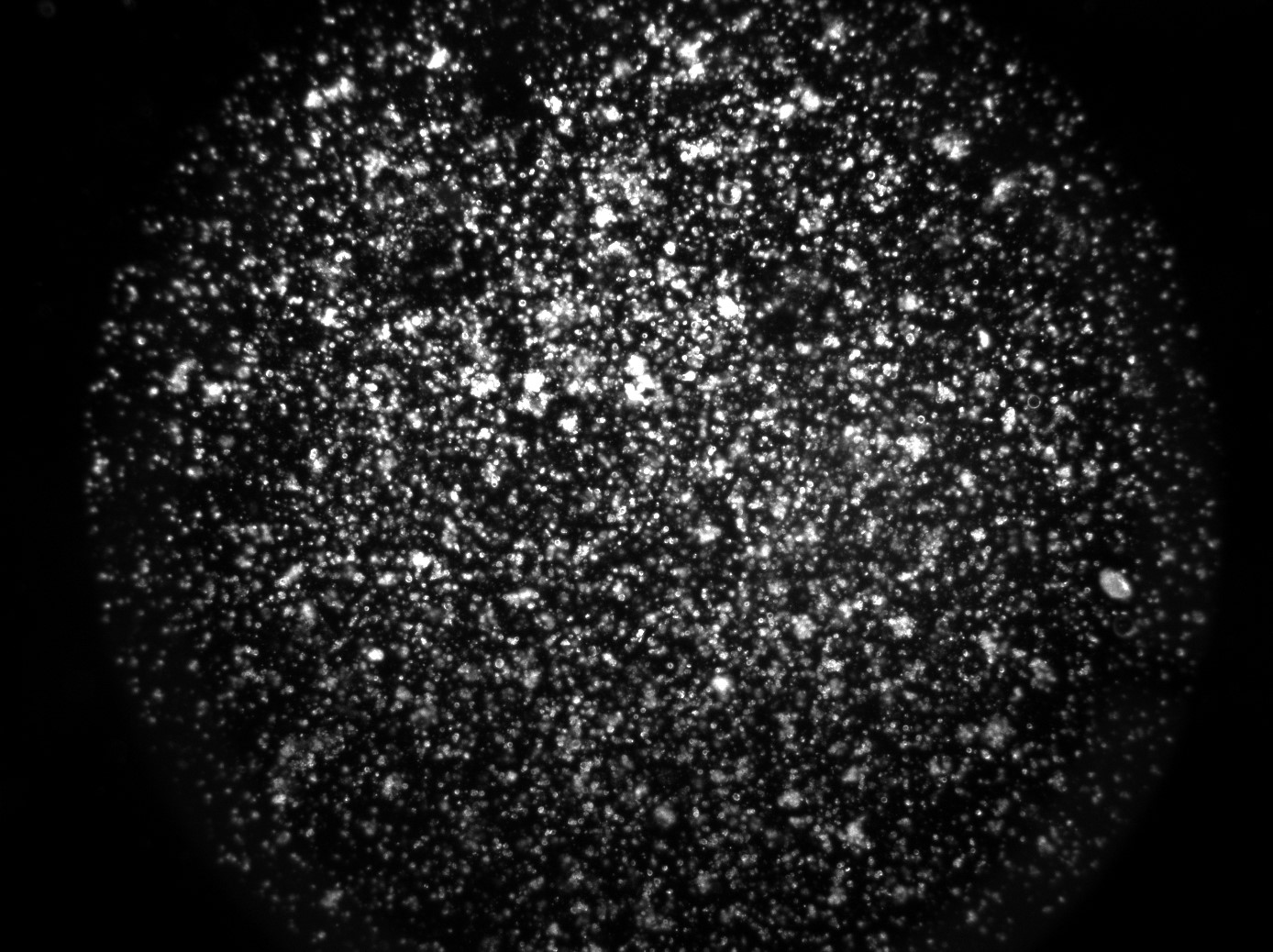

Supplement: Supplementary file 4 — Source Data [file 41467_2024_45605_MOESM4_ESM.zip › Source Data/Figures_Source_Data/figure 1/panel b/B8.jpg]

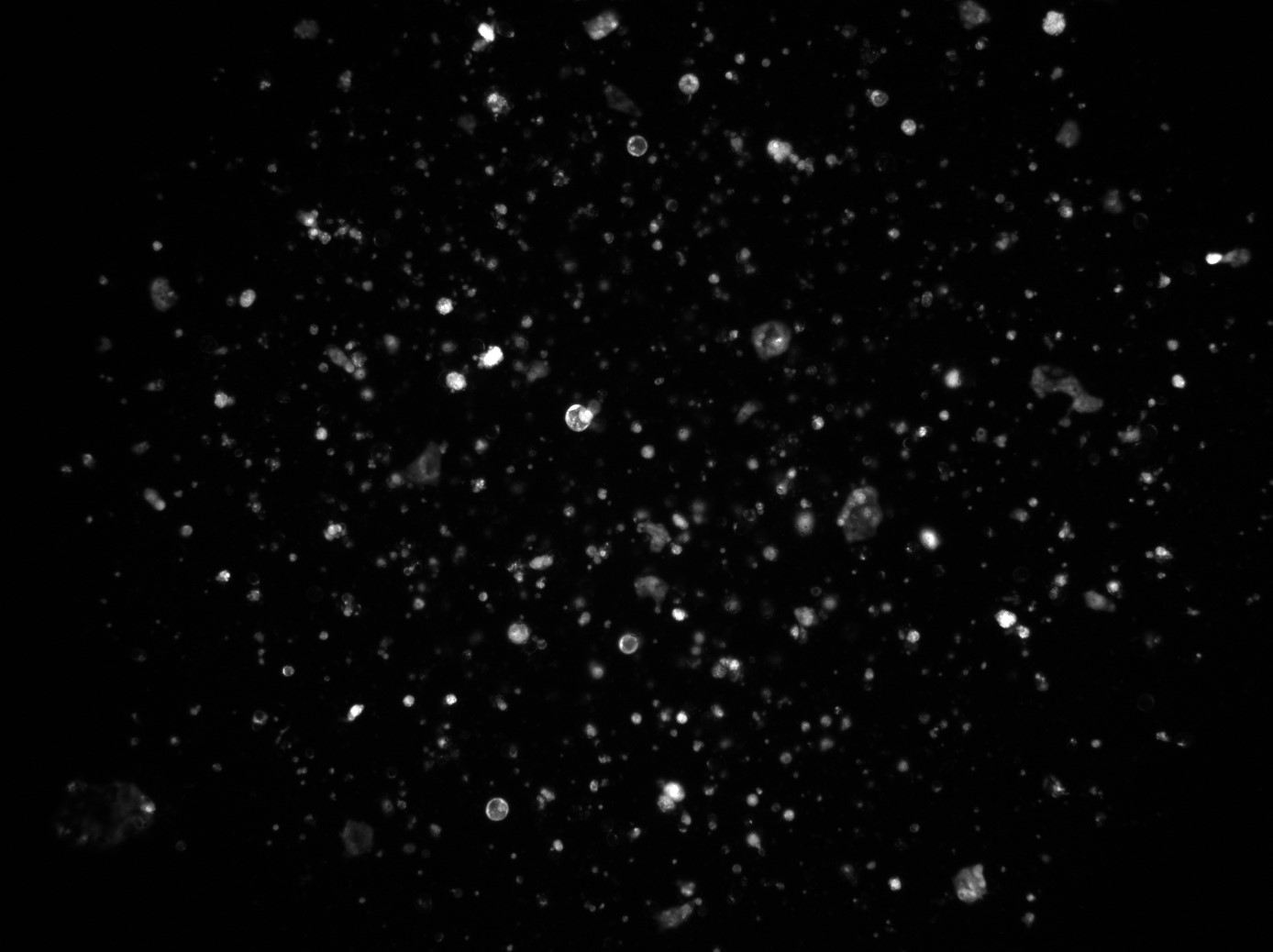

Supplement: Supplementary file 4 — Source Data [file 41467_2024_45605_MOESM4_ESM.zip › Source Data/Figures_Source_Data/figure 1/panel b/B6_TdTomato.jpg]

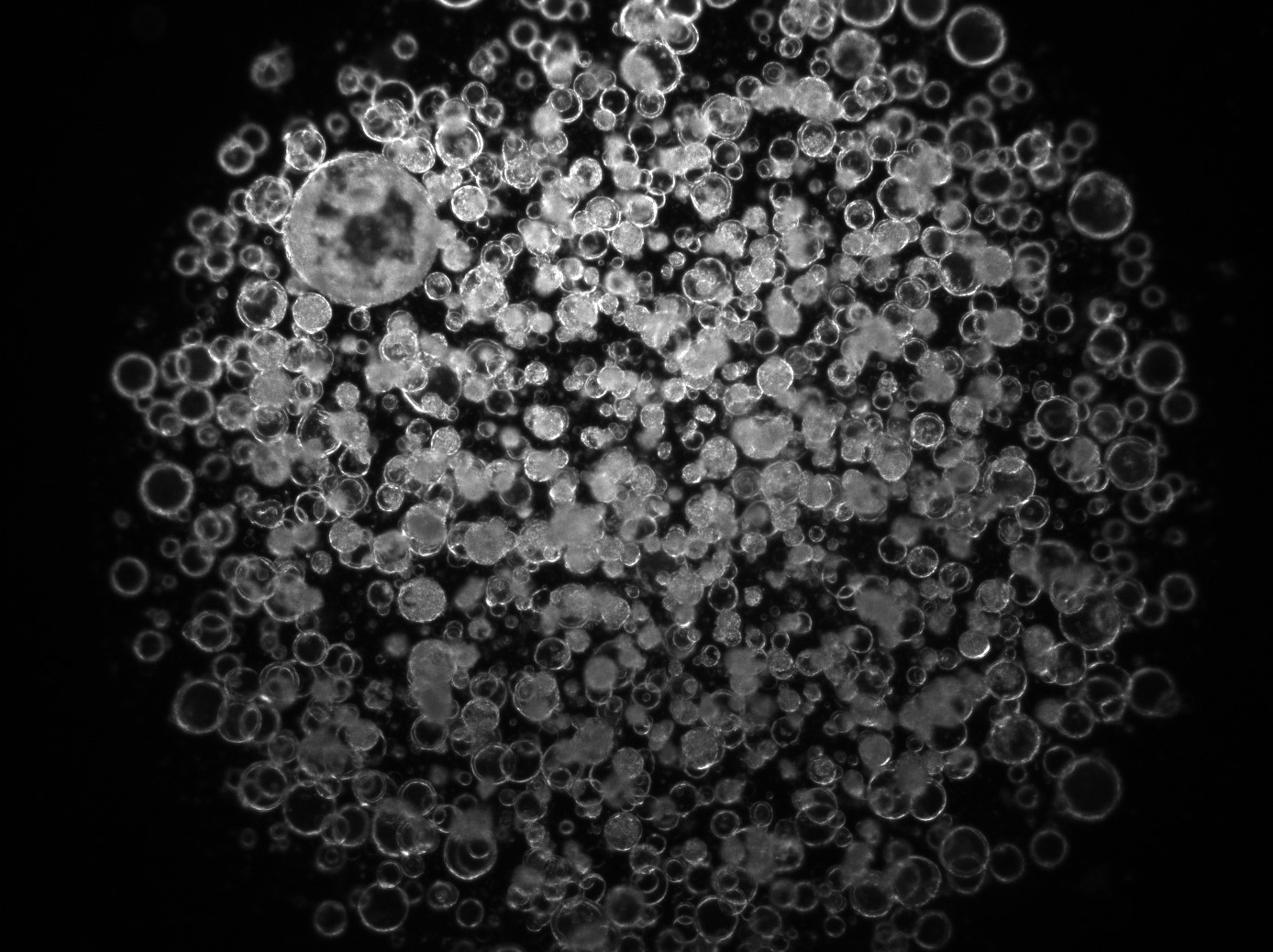

Supplement: Supplementary file 4 — Source Data [file 41467_2024_45605_MOESM4_ESM.zip › Source Data/Figures_Source_Data/figure 1/panel b/B24.jpg]

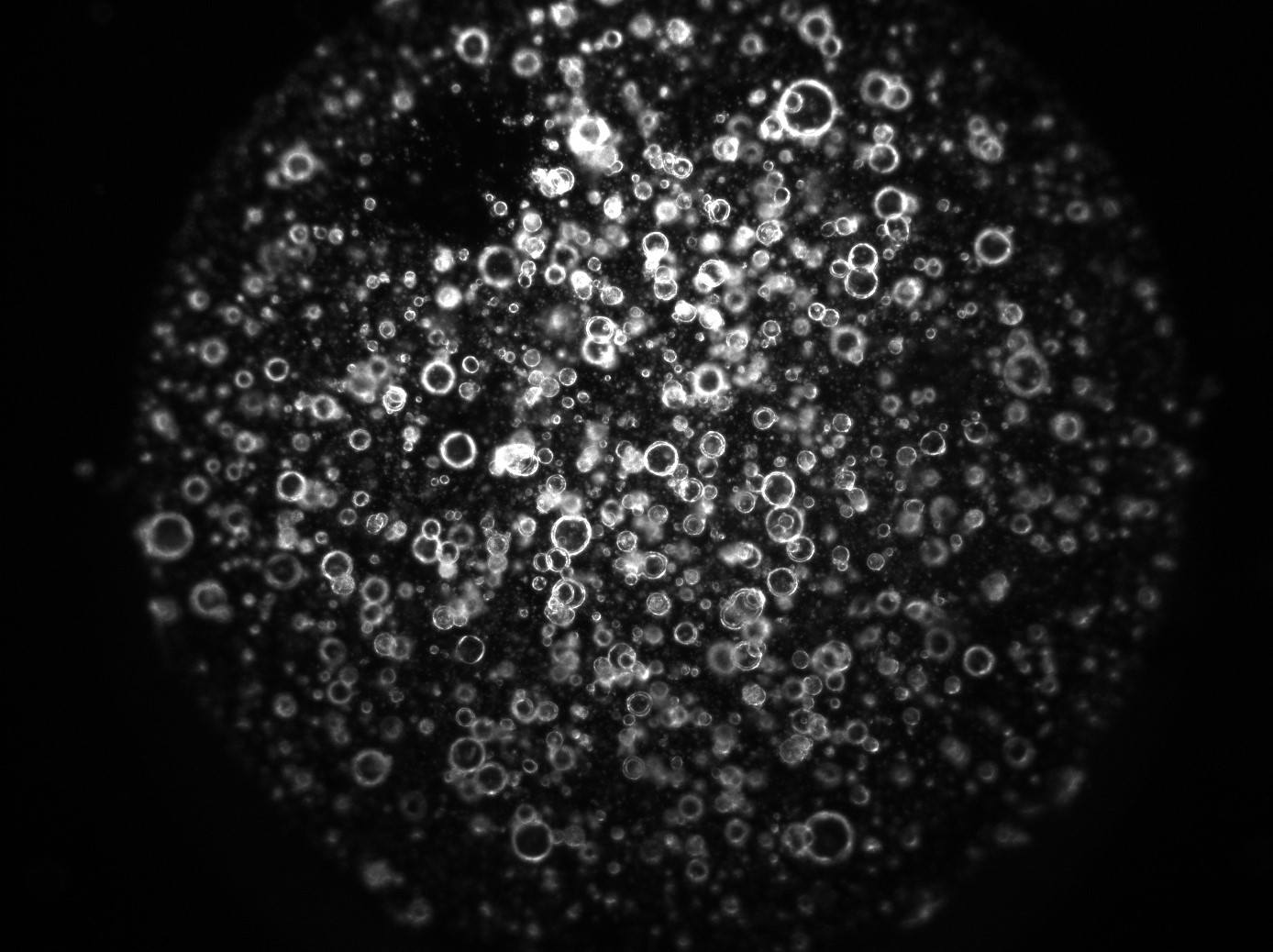

Supplement: Supplementary file 4 — Source Data [file 41467_2024_45605_MOESM4_ESM.zip › Source Data/Figures_Source_Data/figure 1/panel b/B18.jpg]

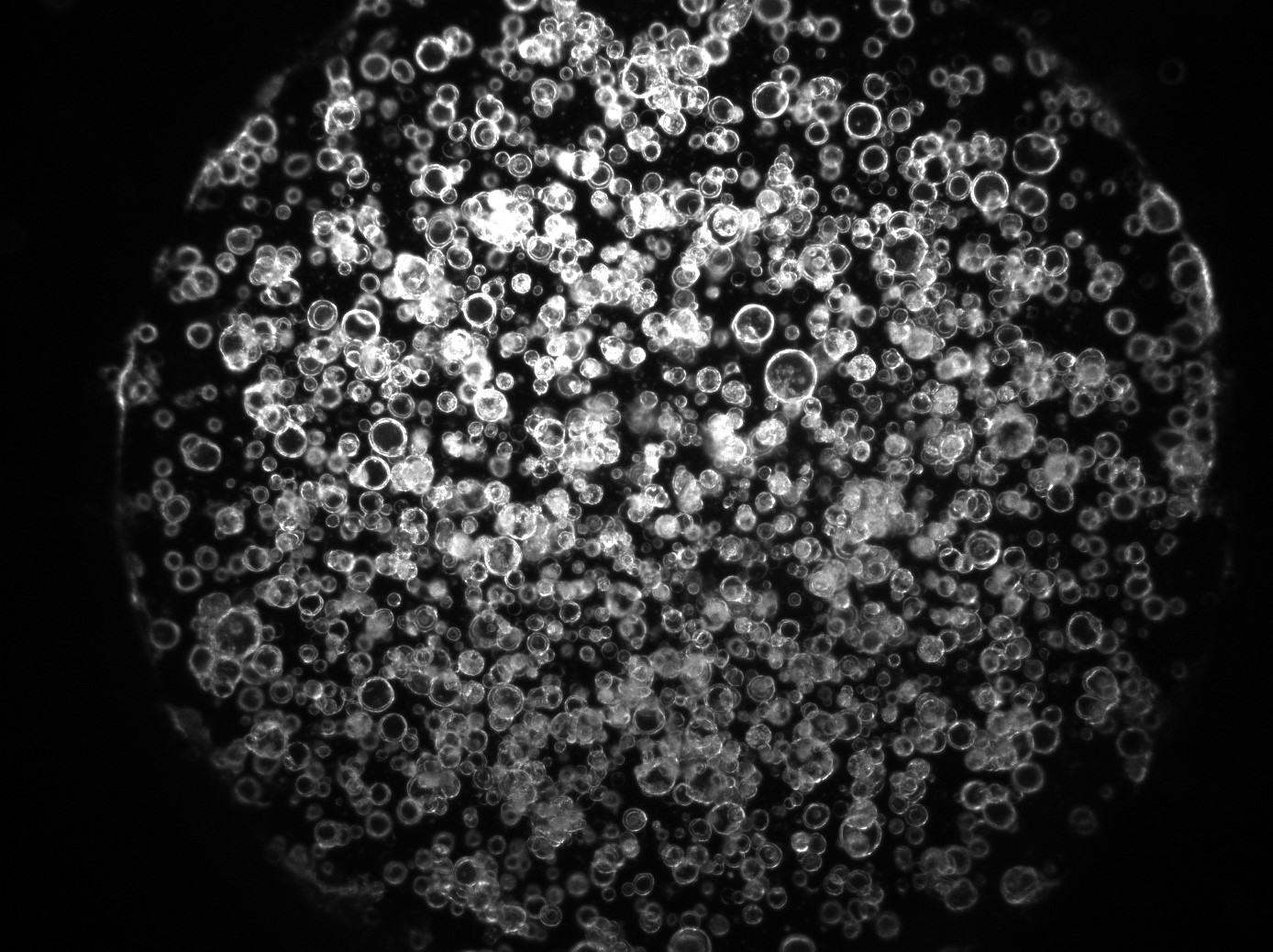

Supplement: Supplementary file 4 — Source Data [file 41467_2024_45605_MOESM4_ESM.zip › Source Data/Figures_Source_Data/figure 1/panel b/B19.jpg]

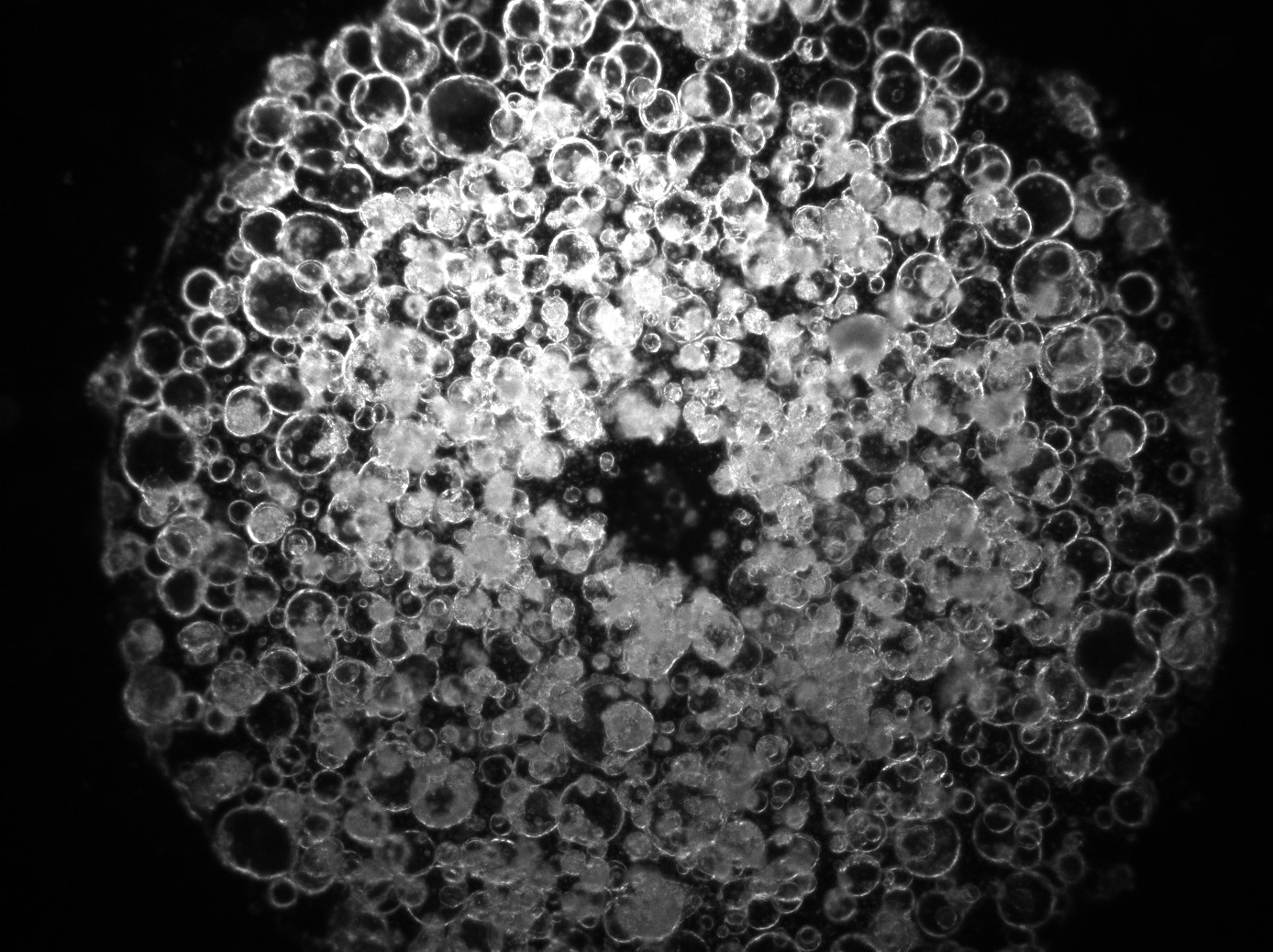

Supplement: Supplementary file 4 — Source Data [file 41467_2024_45605_MOESM4_ESM.zip › Source Data/Figures_Source_Data/figure 1/panel b/B22.jpg]

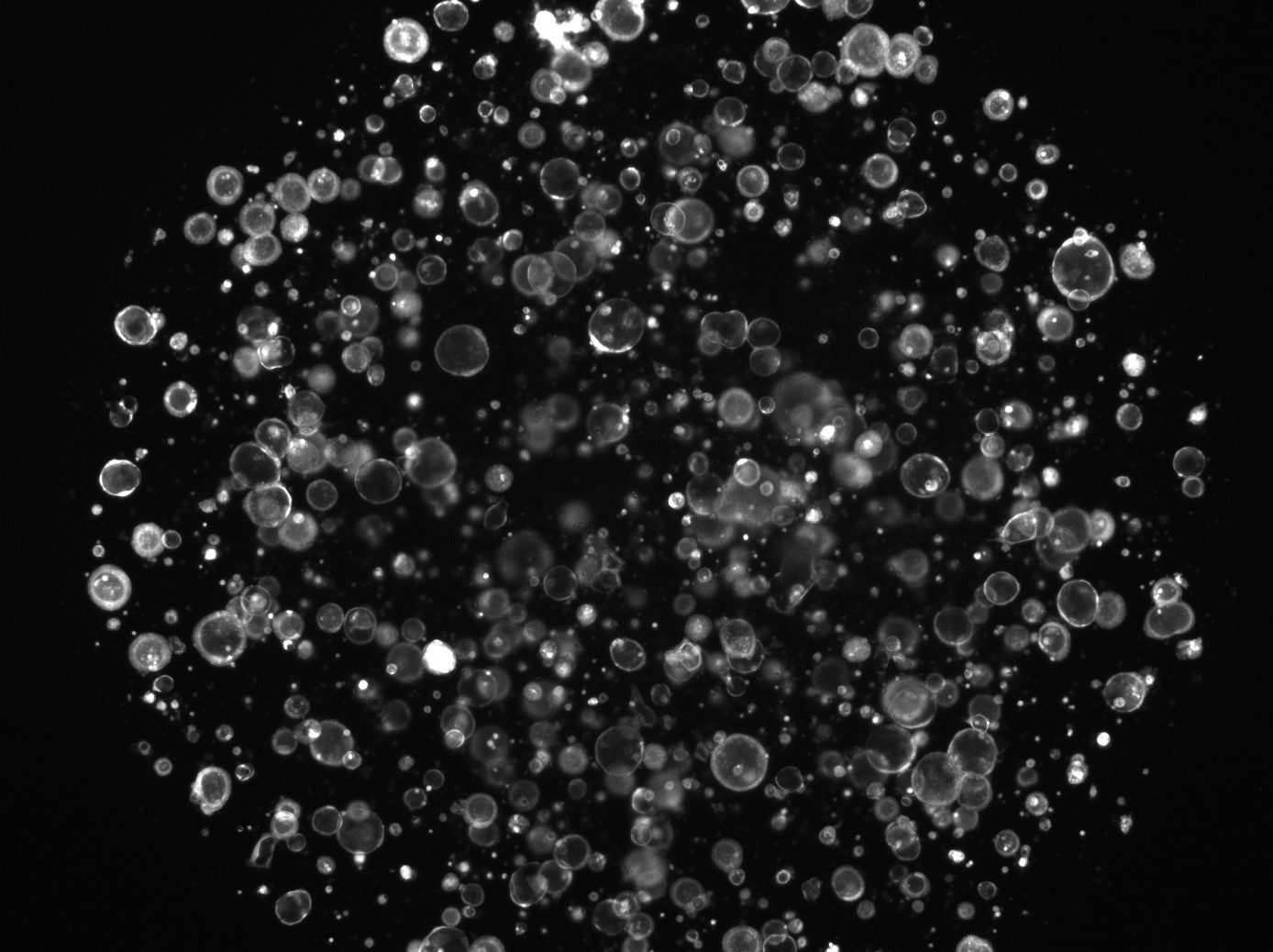

Supplement: Supplementary file 4 — Source Data [file 41467_2024_45605_MOESM4_ESM.zip › Source Data/Figures_Source_Data/figure 1/panel b/B7_Tdtomato.jpg]

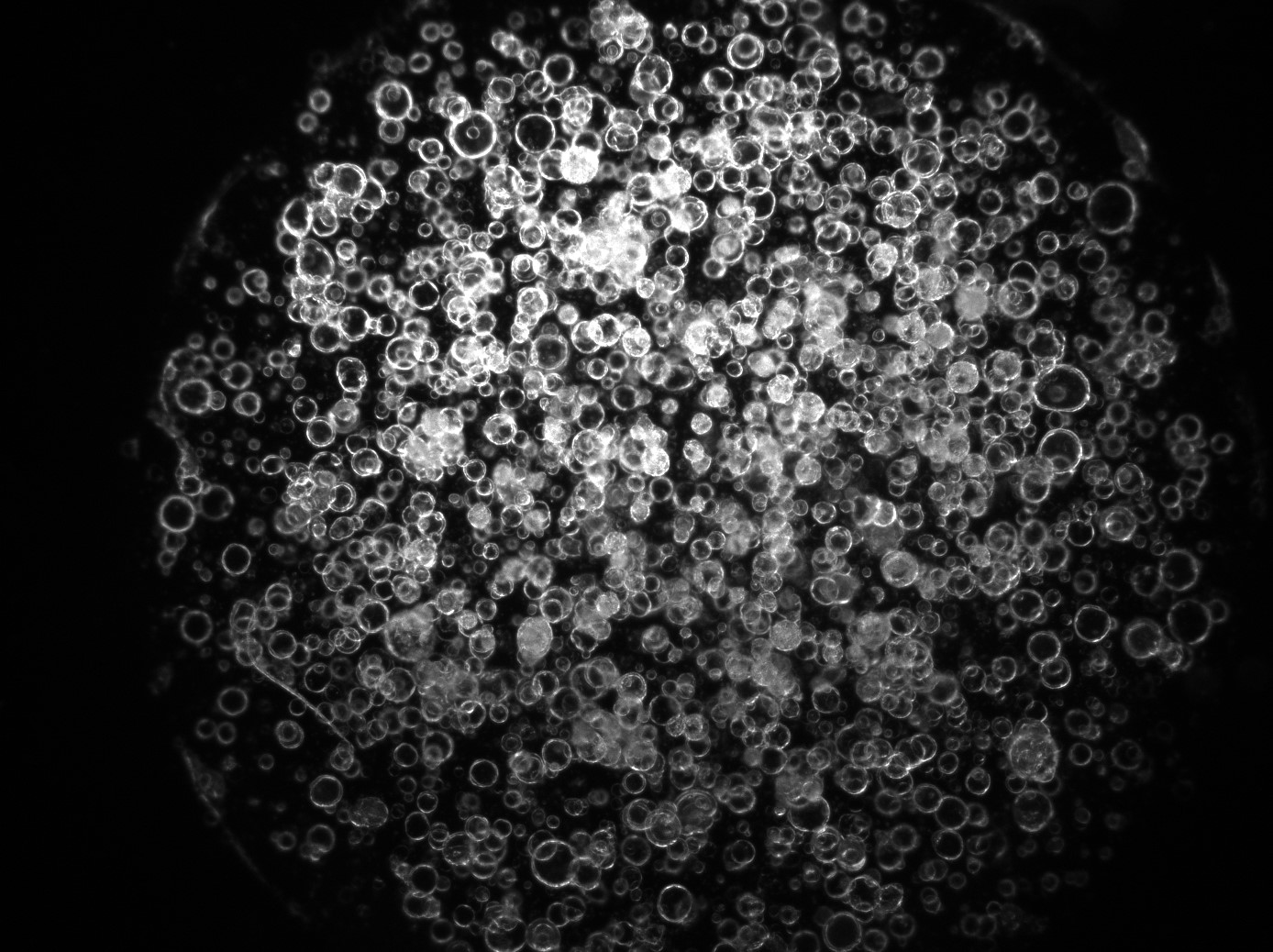

Supplement: Supplementary file 4 — Source Data [file 41467_2024_45605_MOESM4_ESM.zip › Source Data/Figures_Source_Data/figure 1/panel b/B23.jpg]

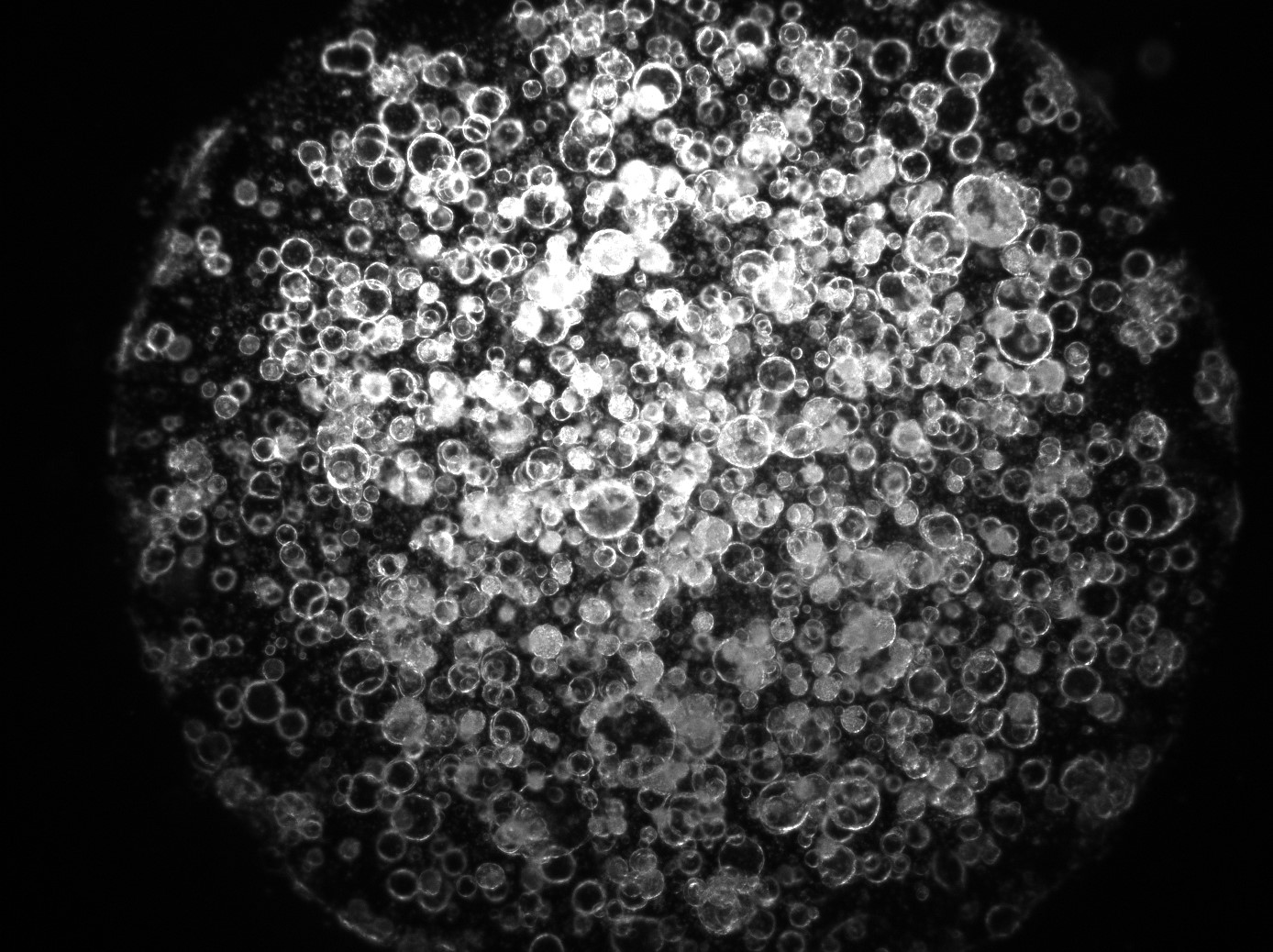

Supplement: Supplementary file 4 — Source Data [file 41467_2024_45605_MOESM4_ESM.zip › Source Data/Figures_Source_Data/figure 1/panel b/B21.jpg]

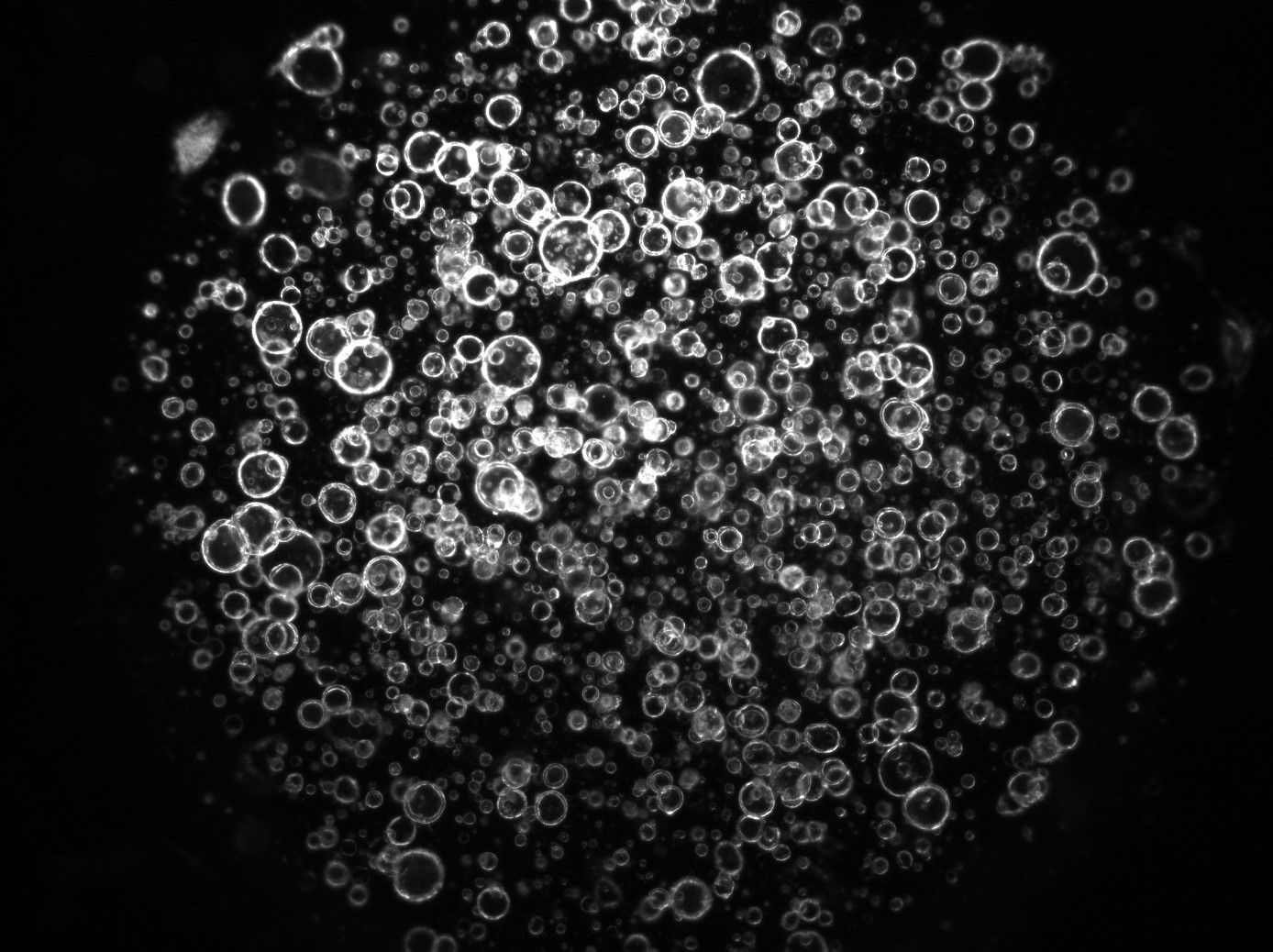

Supplement: Supplementary file 4 — Source Data [file 41467_2024_45605_MOESM4_ESM.zip › Source Data/Figures_Source_Data/figure 1/panel b/B20.jpg]

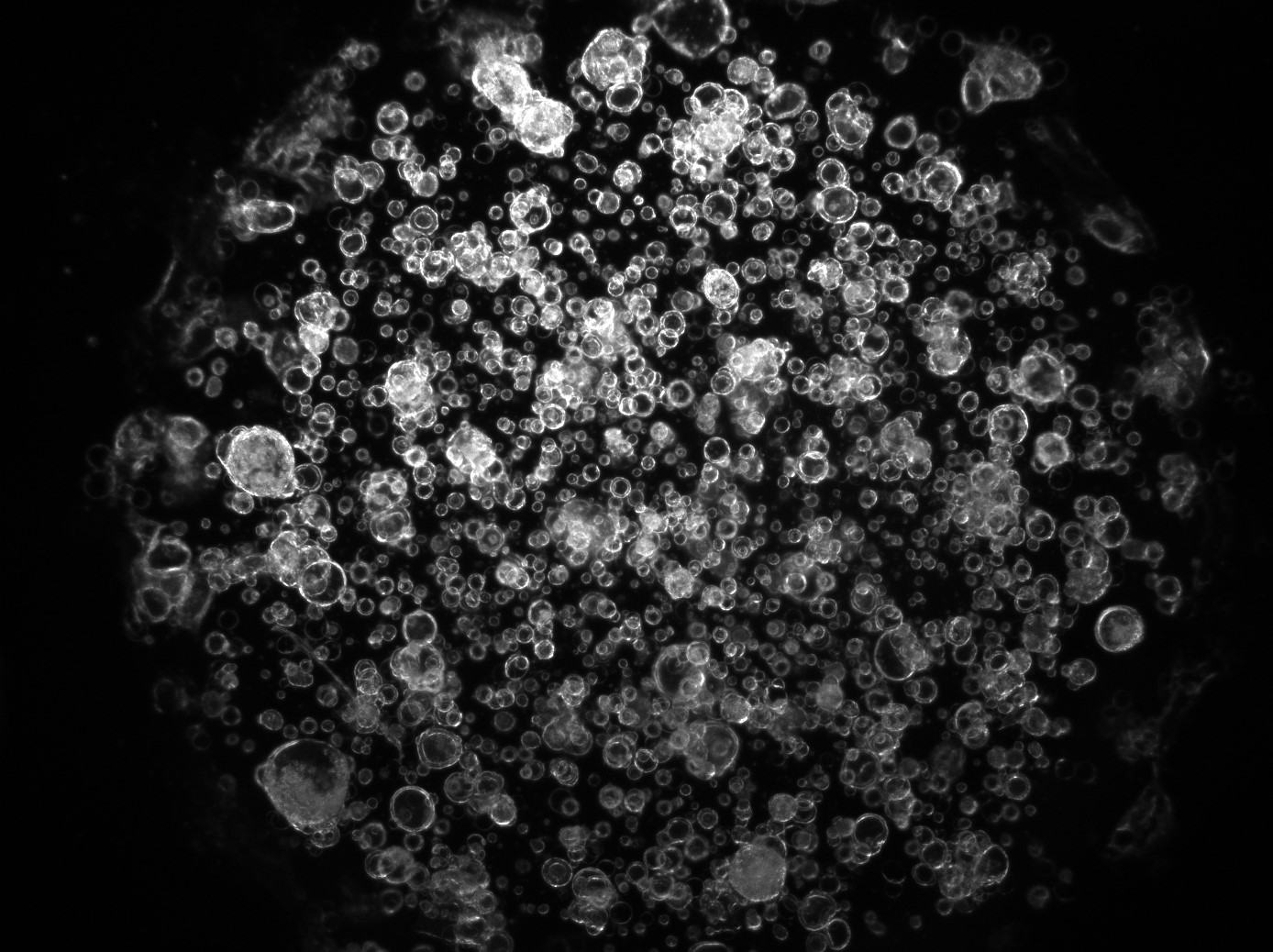

Supplement: Supplementary file 4 — Source Data [file 41467_2024_45605_MOESM4_ESM.zip › Source Data/Figures_Source_Data/figure 1/panel b/B11.jpg]

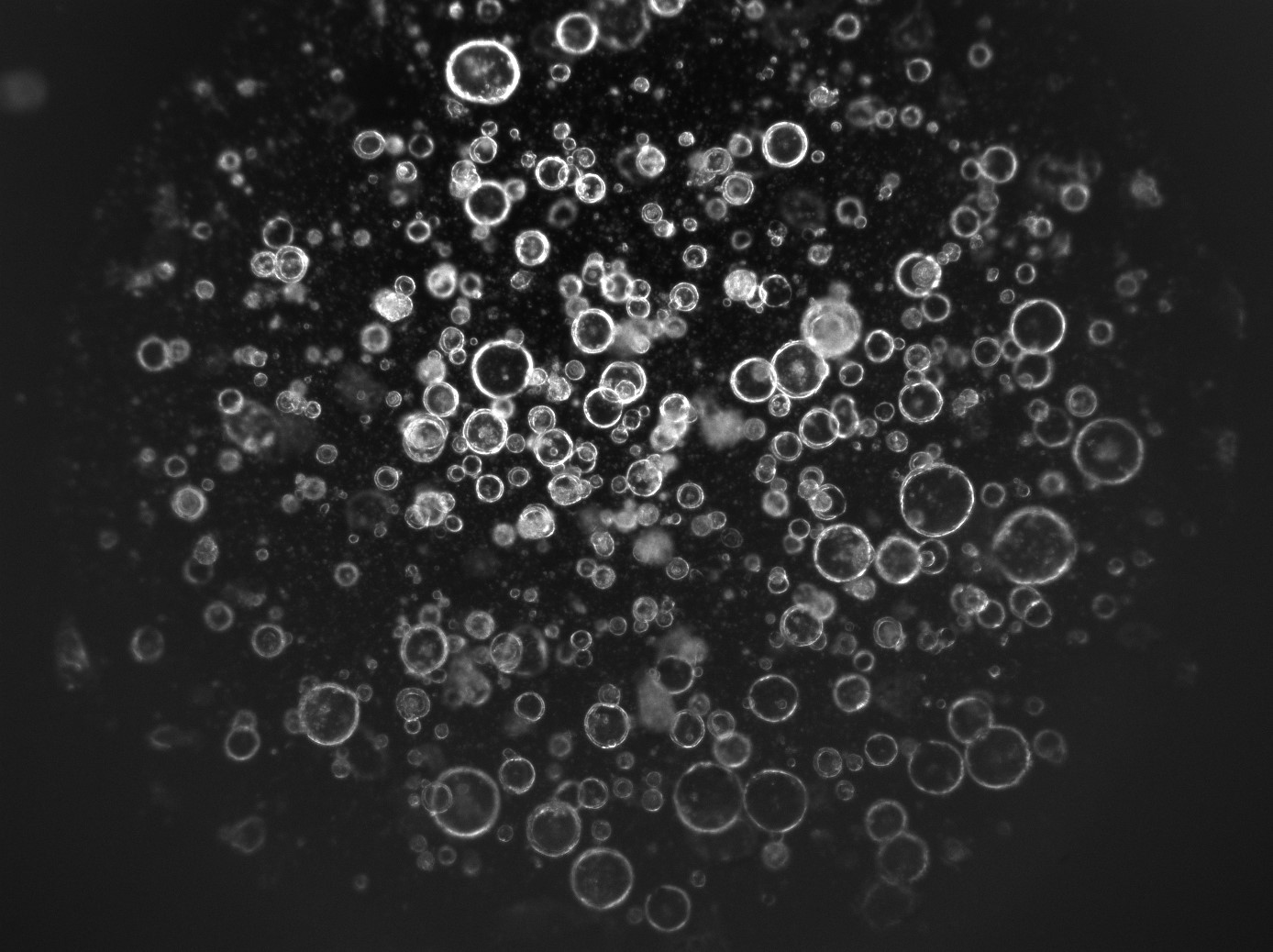

Supplement: Supplementary file 4 — Source Data [file 41467_2024_45605_MOESM4_ESM.zip › Source Data/Figures_Source_Data/figure 1/panel b/B10.jpg]

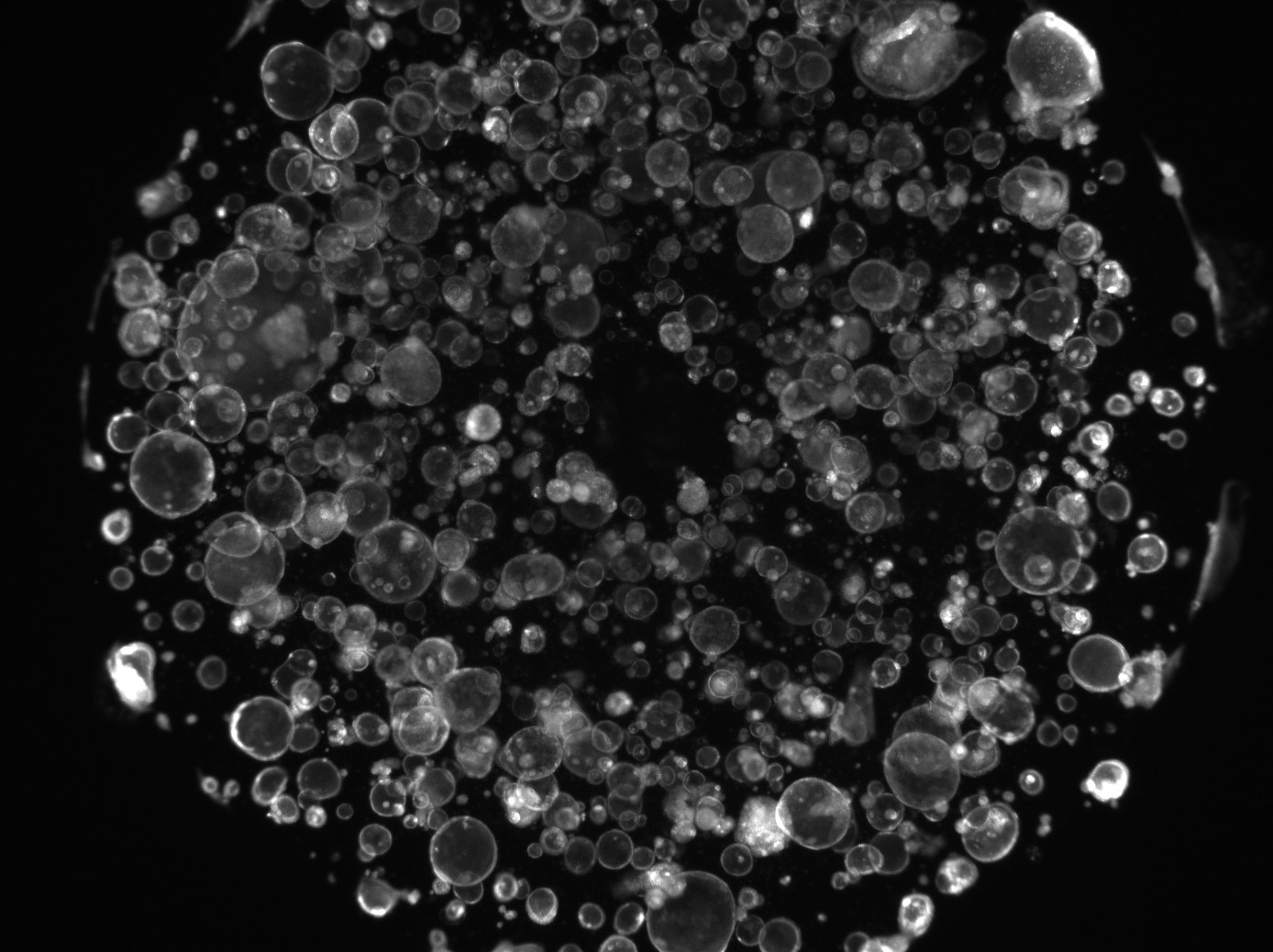

Supplement: Supplementary file 4 — Source Data [file 41467_2024_45605_MOESM4_ESM.zip › Source Data/Figures_Source_Data/figure 1/panel b/B5_TdTomato.jpg]

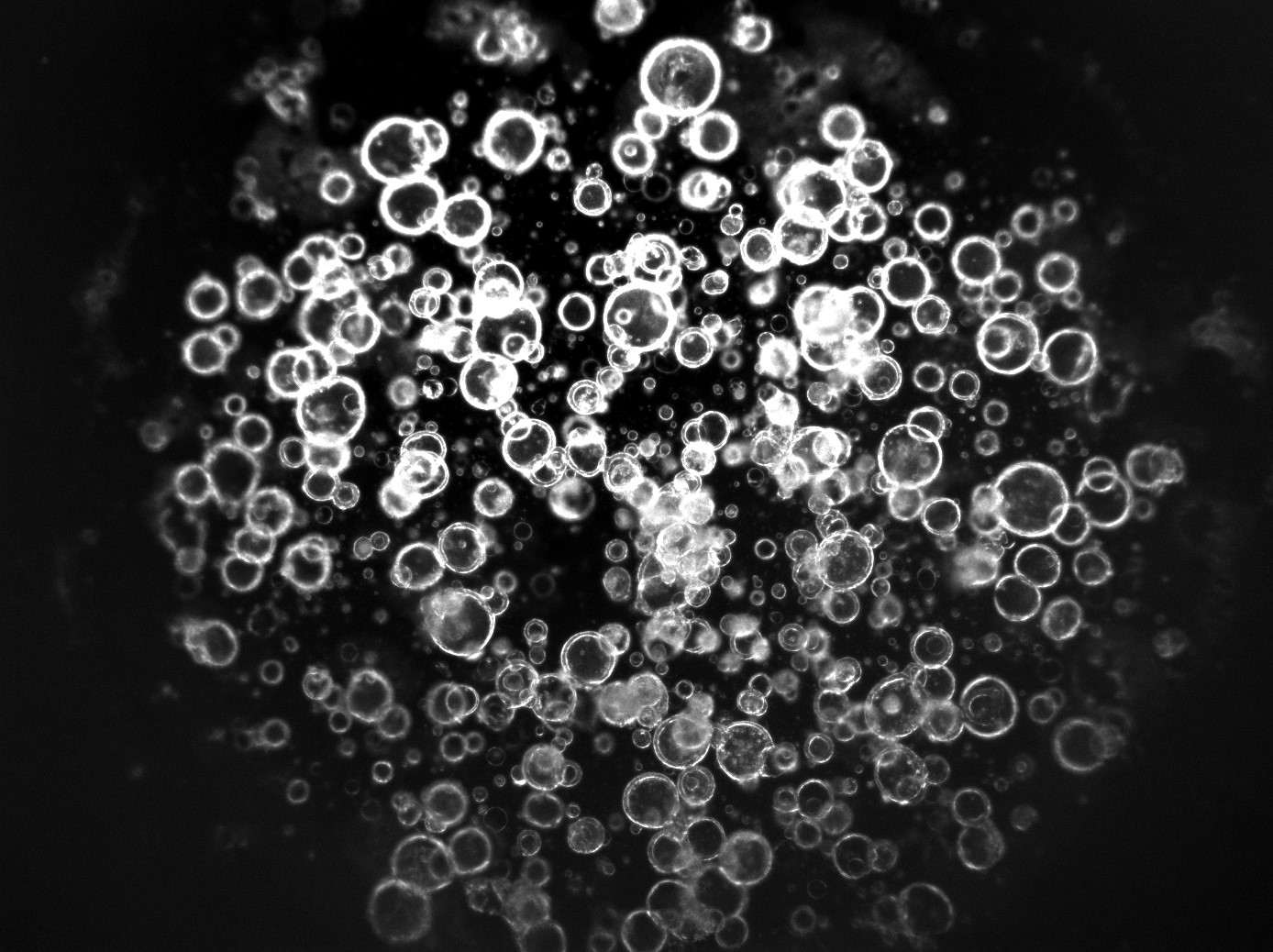

Supplement: Supplementary file 4 — Source Data [file 41467_2024_45605_MOESM4_ESM.zip › Source Data/Figures_Source_Data/figure 1/panel b/B12.jpg]

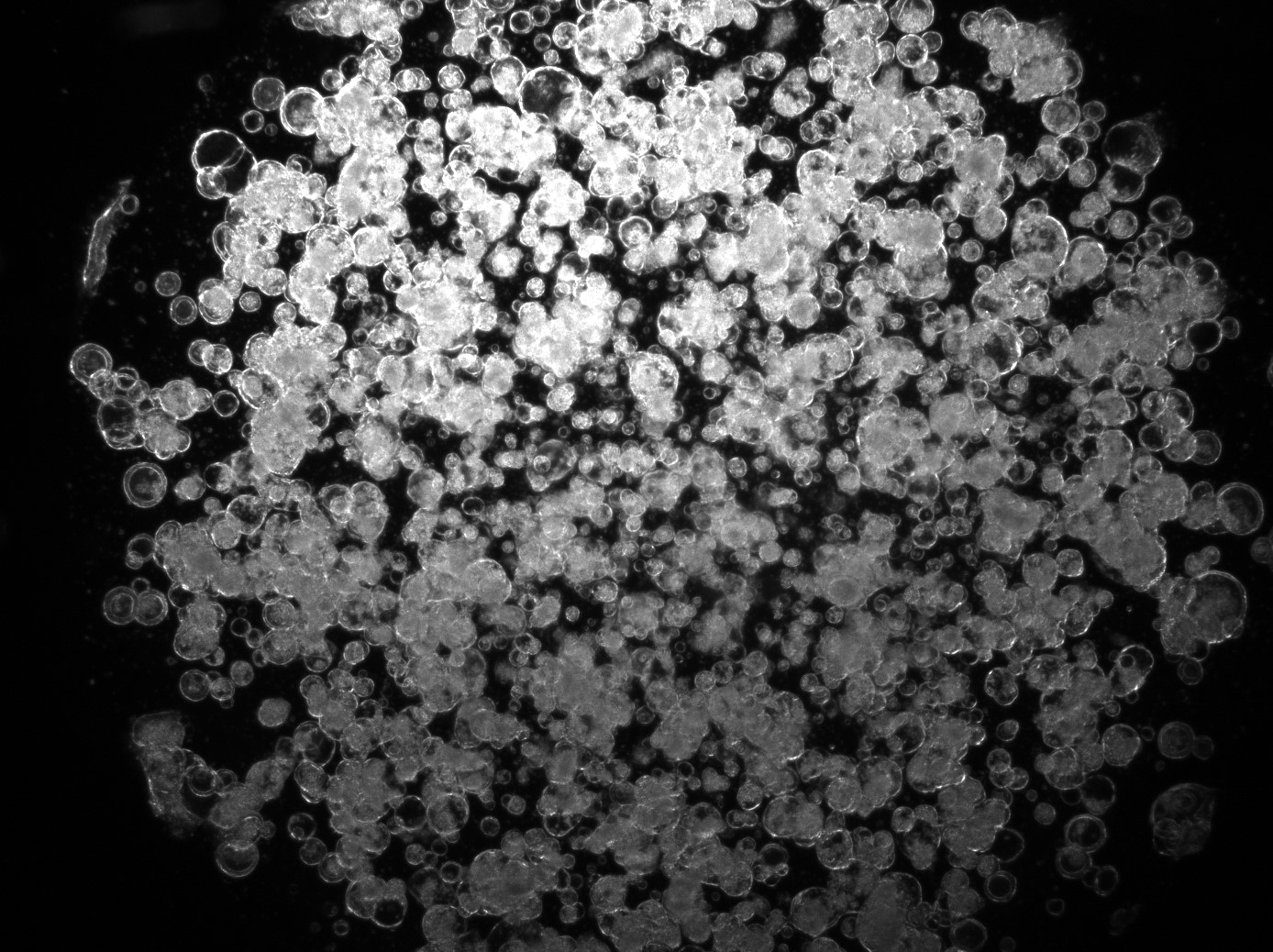

Supplement: Supplementary file 4 — Source Data [file 41467_2024_45605_MOESM4_ESM.zip › Source Data/Figures_Source_Data/figure 1/panel b/B13.jpg]

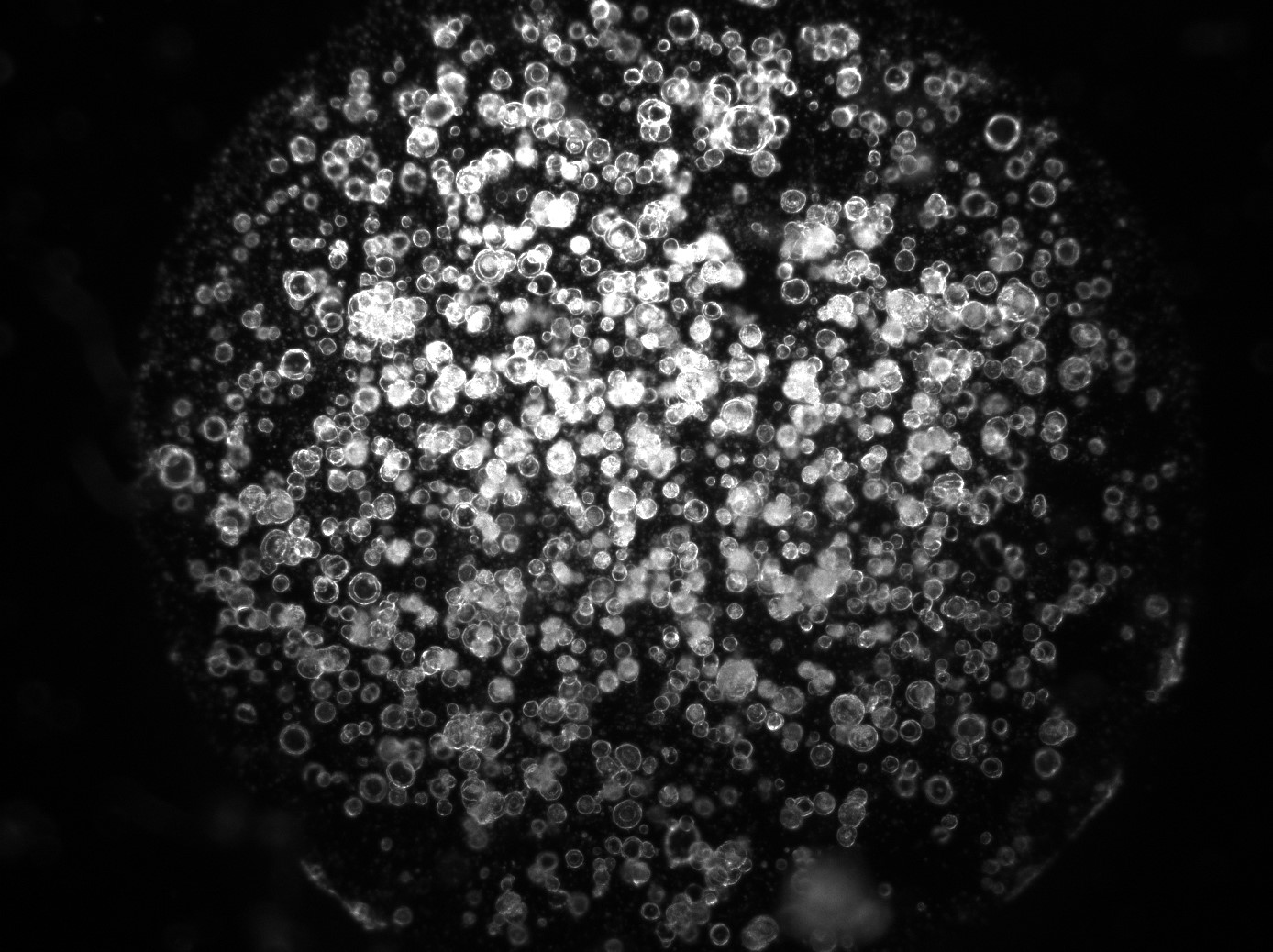

Supplement: Supplementary file 4 — Source Data [file 41467_2024_45605_MOESM4_ESM.zip › Source Data/Figures_Source_Data/figure 1/panel b/B17.jpg]

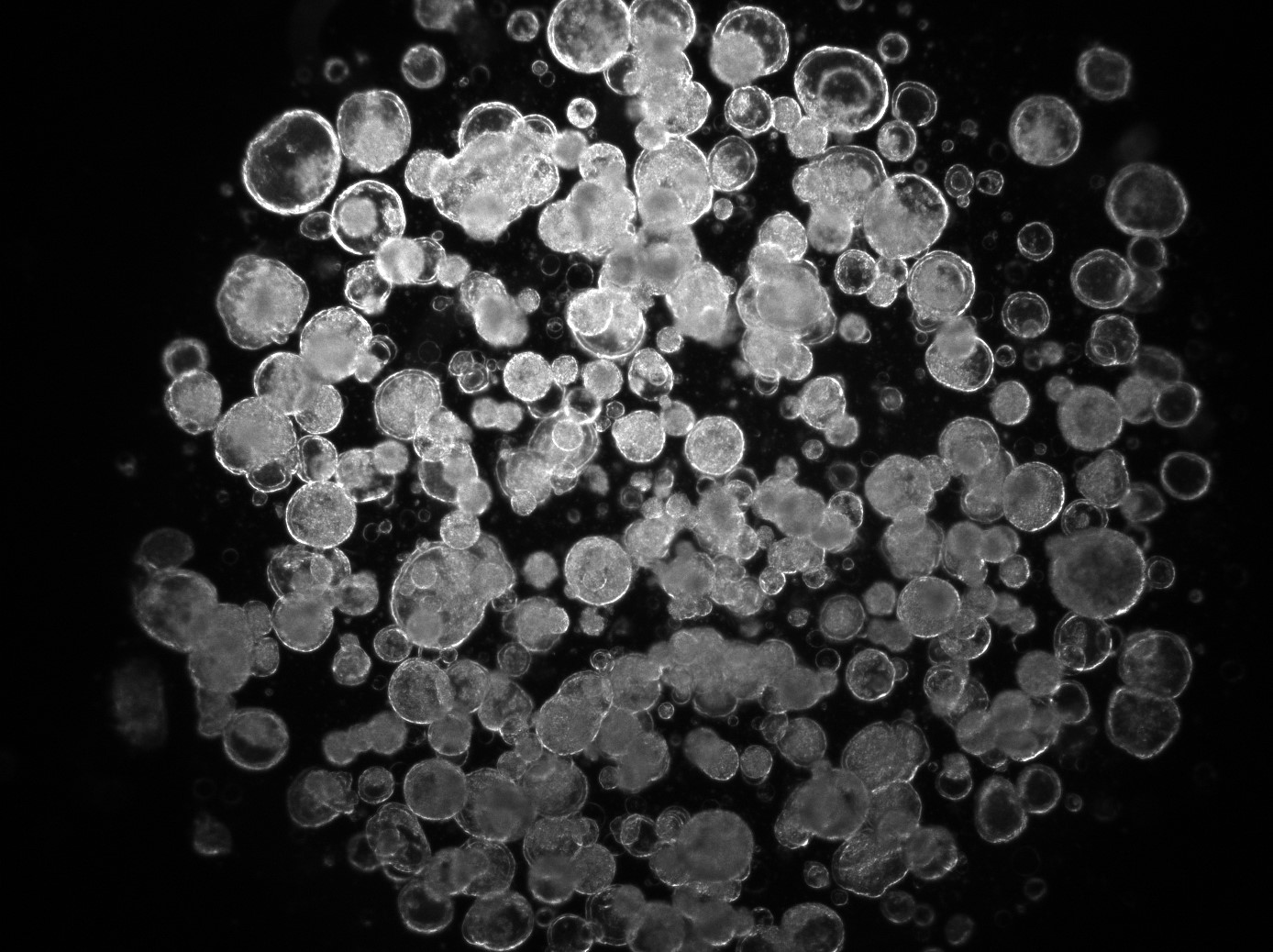

Supplement: Supplementary file 4 — Source Data [file 41467_2024_45605_MOESM4_ESM.zip › Source Data/Figures_Source_Data/figure 1/panel b/B16.jpg]

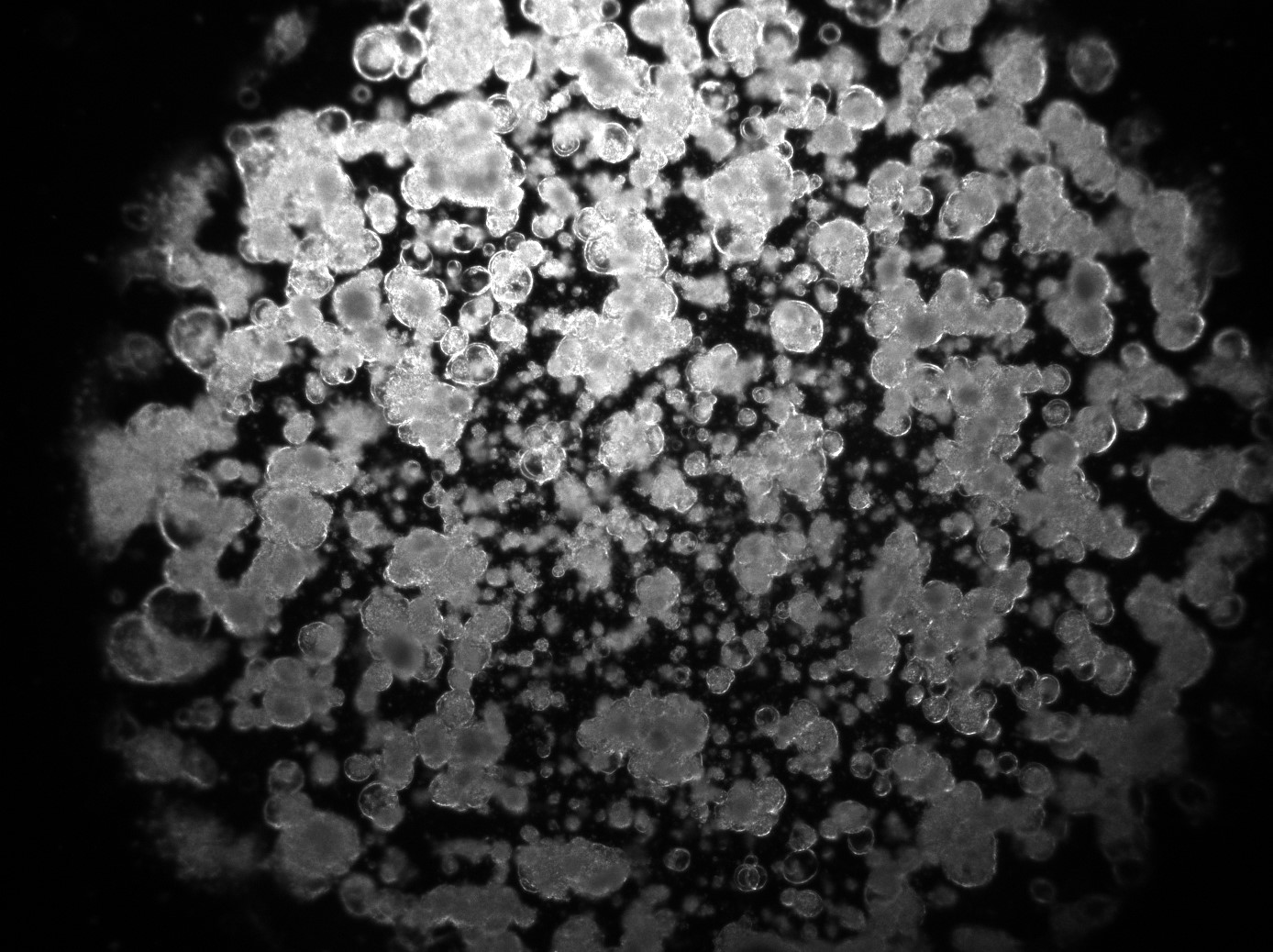

Supplement: Supplementary file 4 — Source Data [file 41467_2024_45605_MOESM4_ESM.zip › Source Data/Figures_Source_Data/figure 1/panel b/B14.jpg]

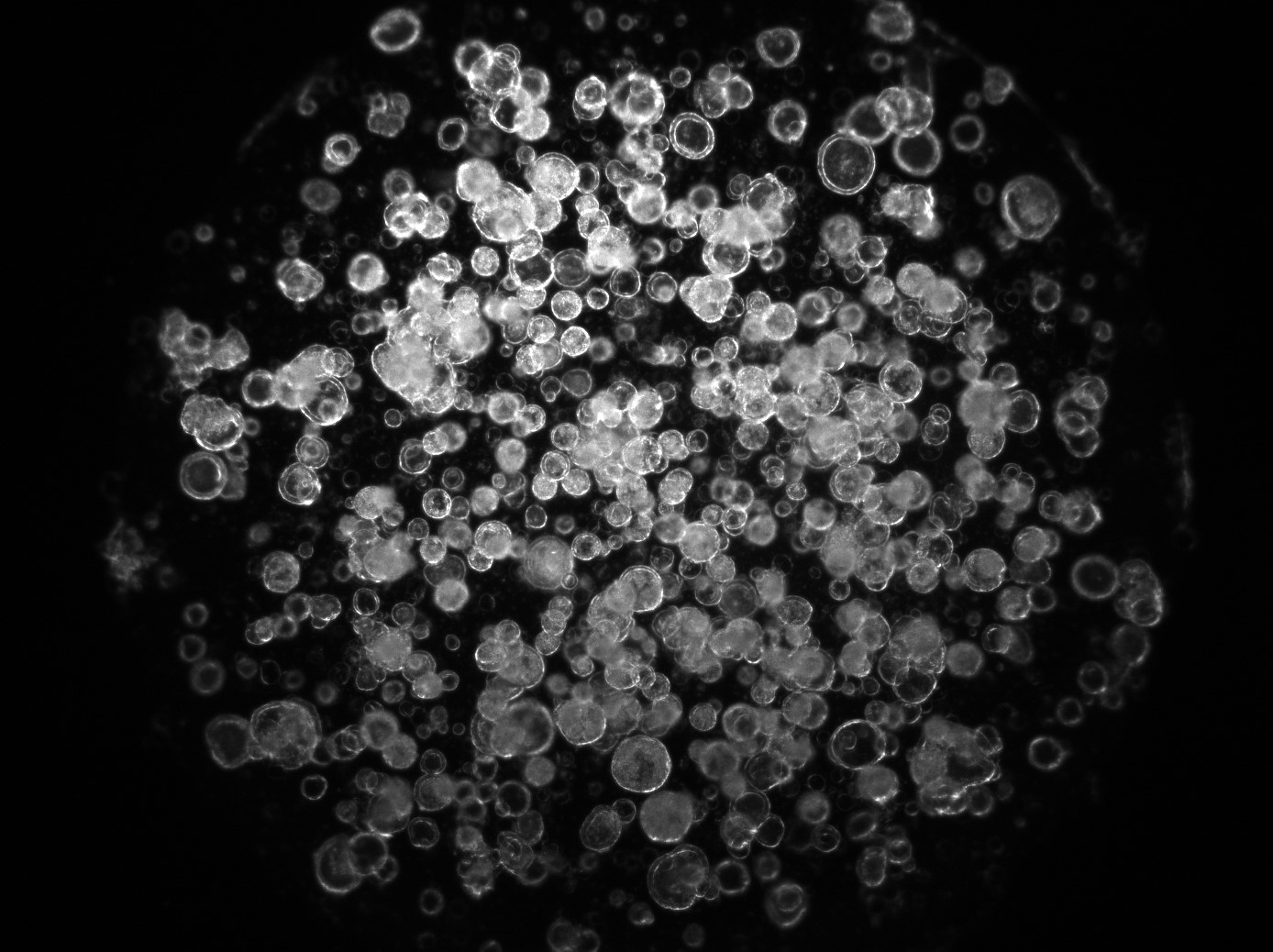

Supplement: Supplementary file 4 — Source Data [file 41467_2024_45605_MOESM4_ESM.zip › Source Data/Figures_Source_Data/figure 1/panel b/B15.jpg]

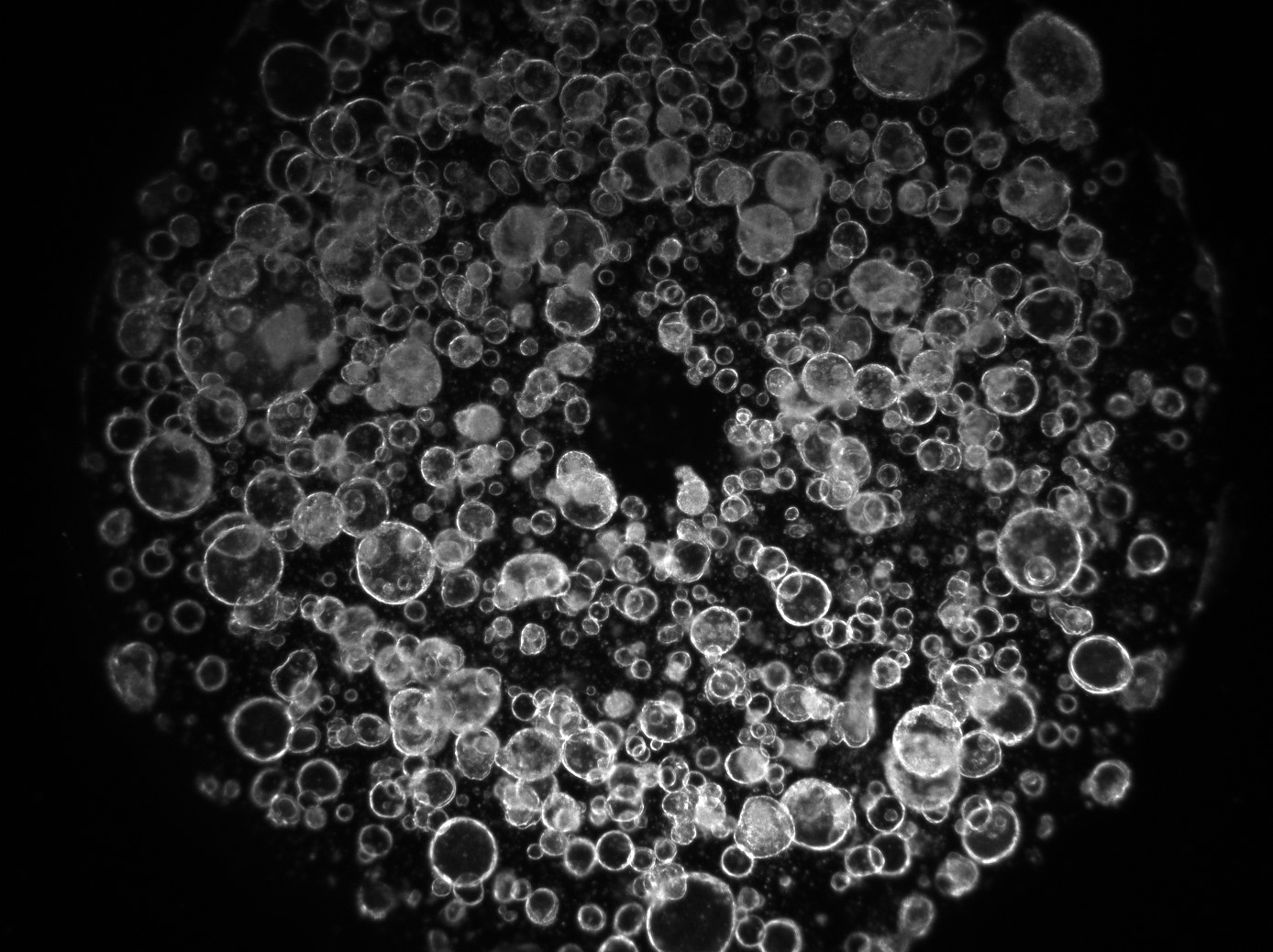

Supplement: Supplementary file 4 — Source Data [file 41467_2024_45605_MOESM4_ESM.zip › Source Data/Figures_Source_Data/figure 1/panel b/B5.jpg]

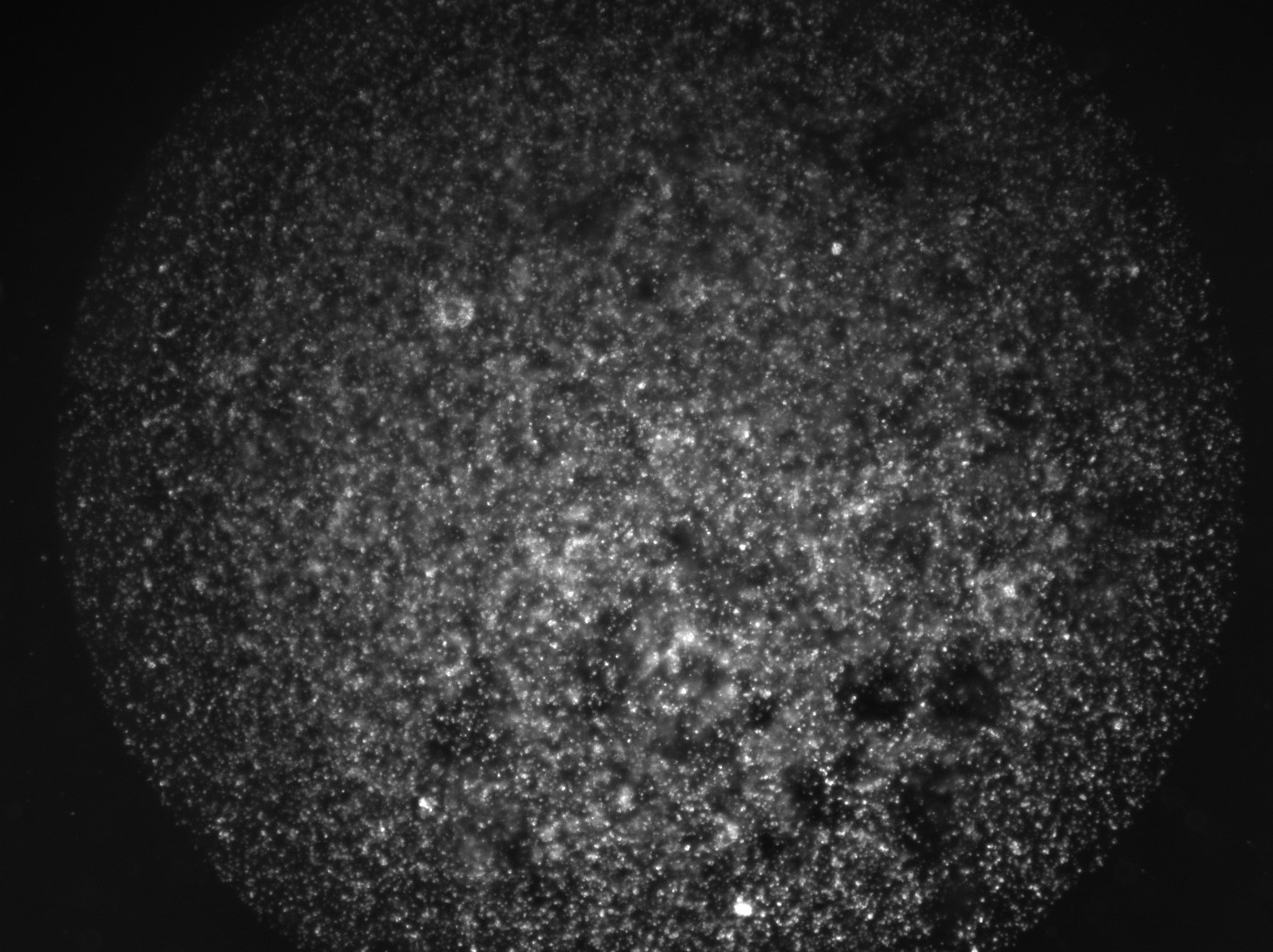

Supplement: Supplementary file 4 — Source Data [file 41467_2024_45605_MOESM4_ESM.zip › Source Data/Figures_Source_Data/figure 1/panel b/B4.jpg]

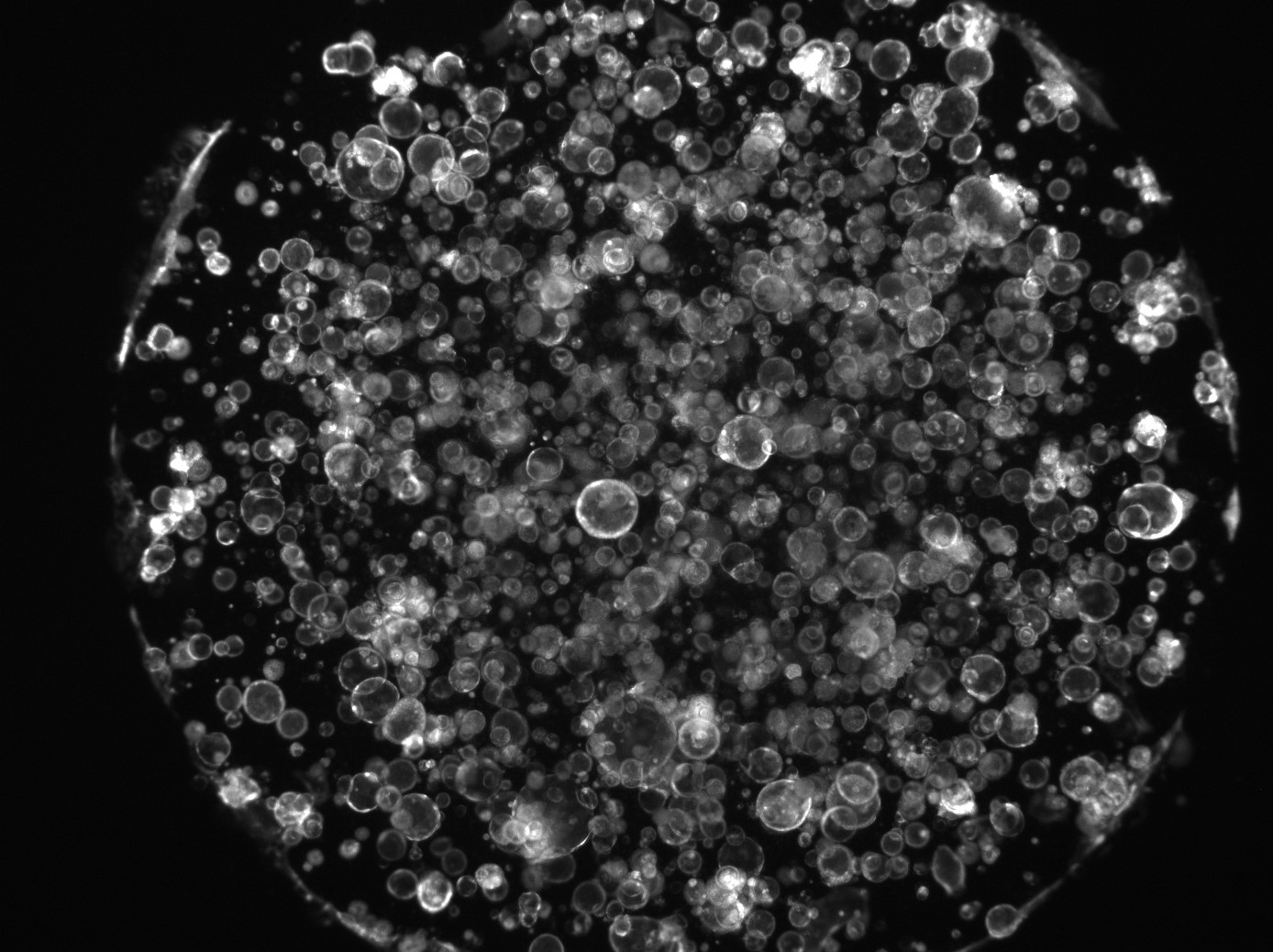

Supplement: Supplementary file 4 — Source Data [file 41467_2024_45605_MOESM4_ESM.zip › Source Data/Figures_Source_Data/figure 1/panel b/B21_TdTomato.jpg]

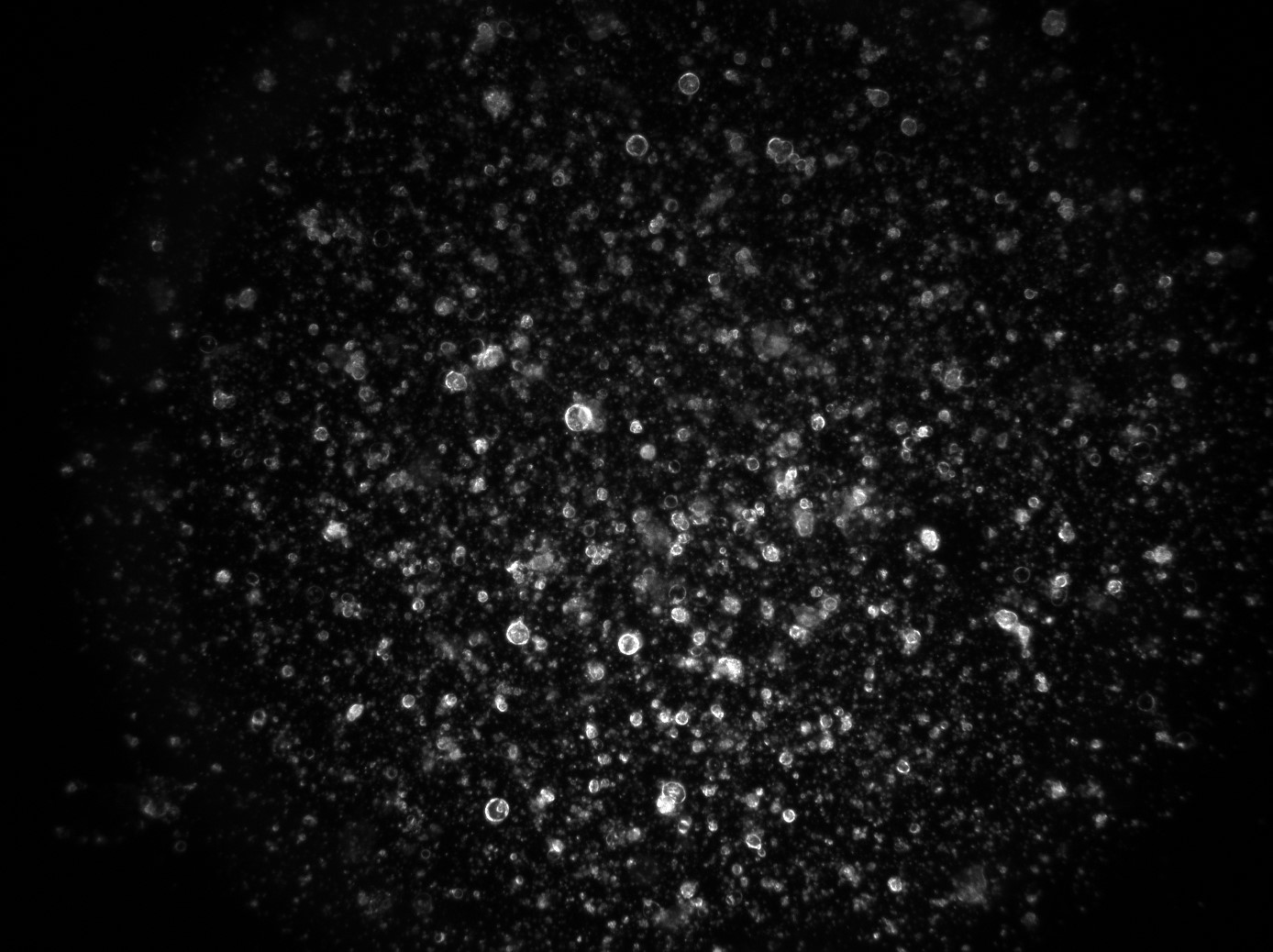

Supplement: Supplementary file 4 — Source Data [file 41467_2024_45605_MOESM4_ESM.zip › Source Data/Figures_Source_Data/figure 1/panel b/B6.jpg]

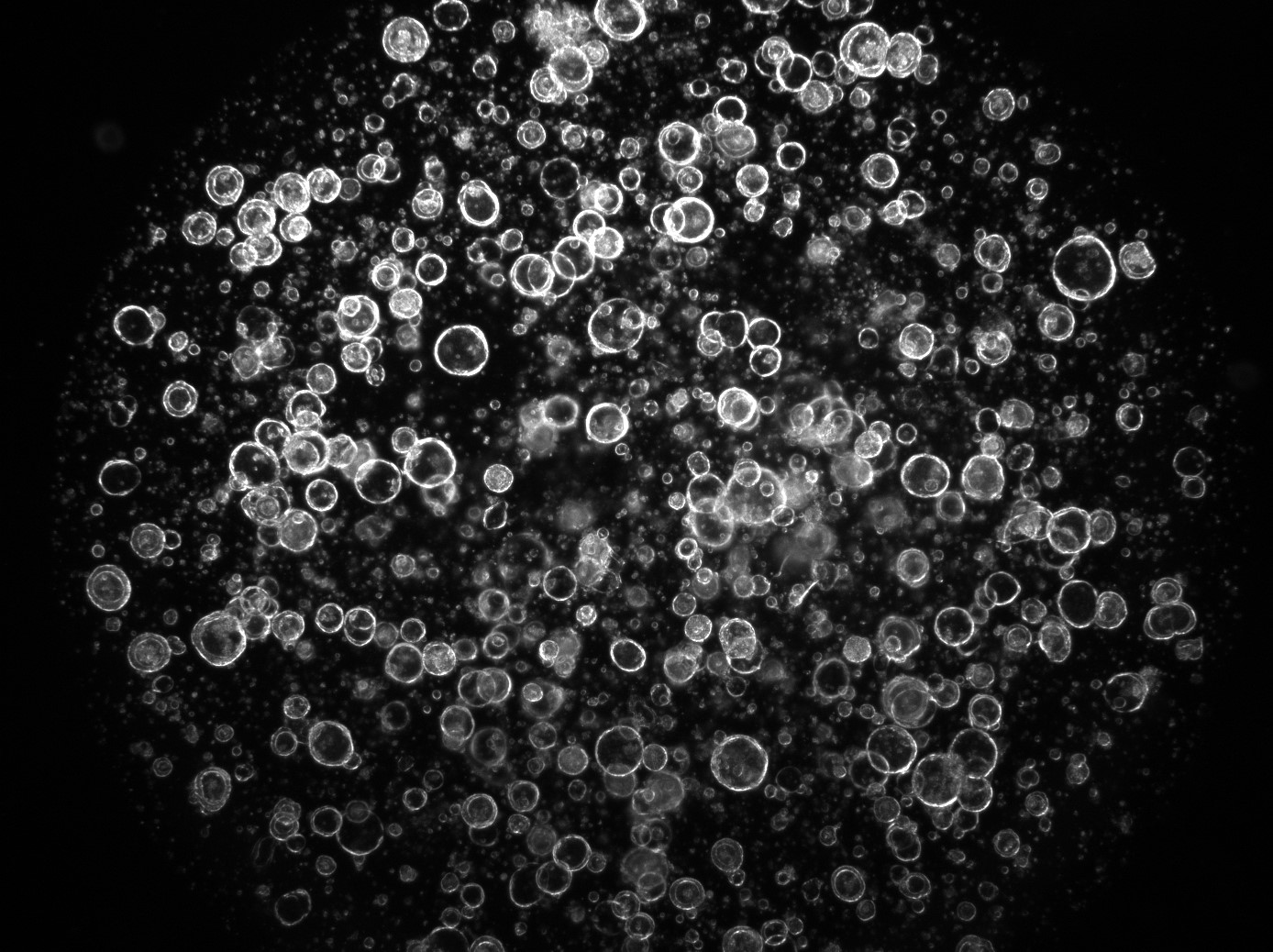

Supplement: Supplementary file 4 — Source Data [file 41467_2024_45605_MOESM4_ESM.zip › Source Data/Figures_Source_Data/figure 1/panel b/B7.jpg]

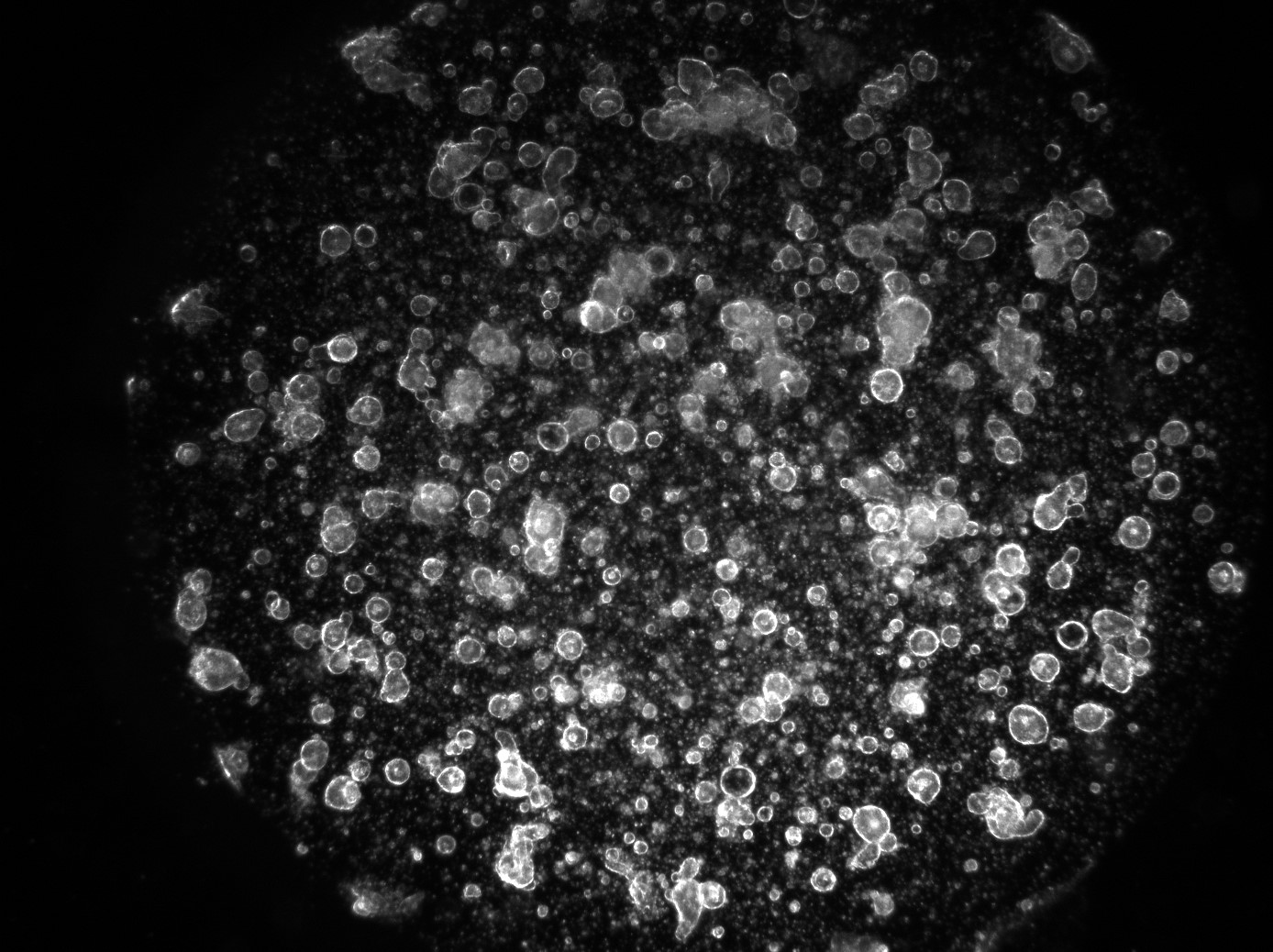

Supplement: Supplementary file 4 — Source Data [file 41467_2024_45605_MOESM4_ESM.zip › Source Data/Figures_Source_Data/figure 1/panel b/B3.jpg]

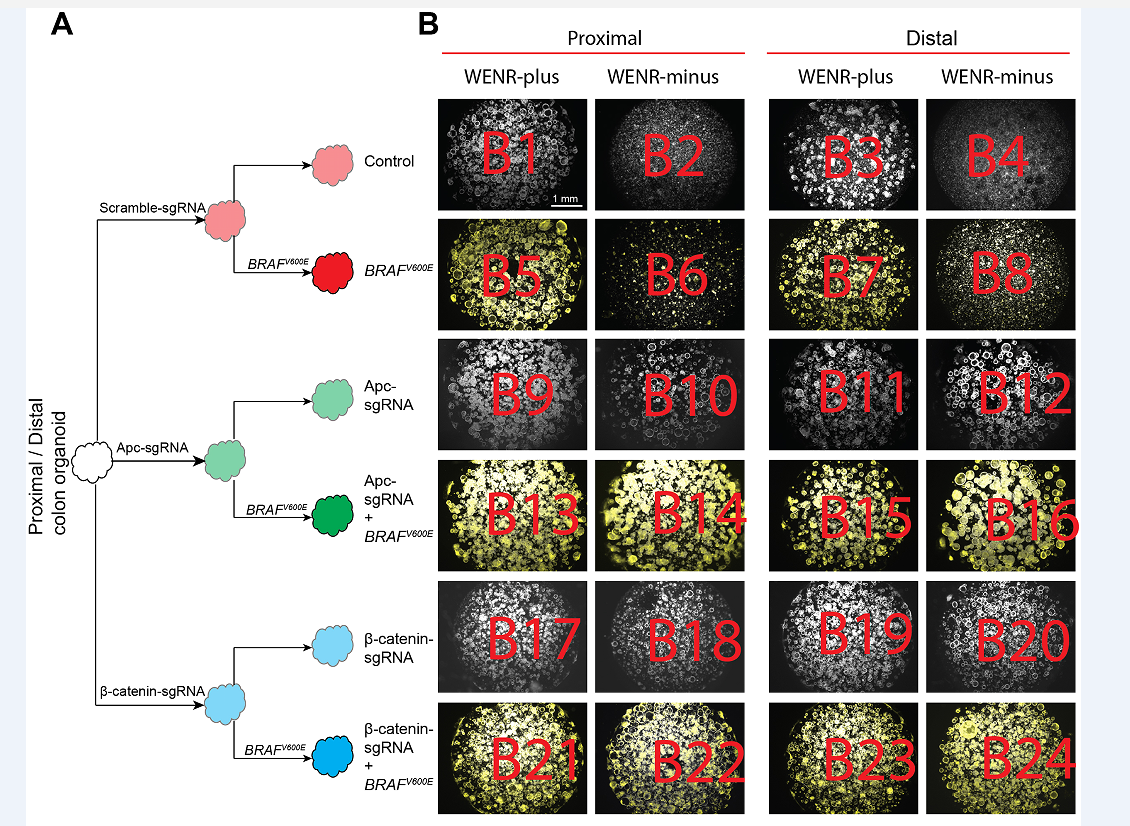

Supplement: Supplementary file 4 — Source Data [file 41467_2024_45605_MOESM4_ESM.zip › Source Data/Figures_Source_Data/figure 1/panel b/panel b.png]

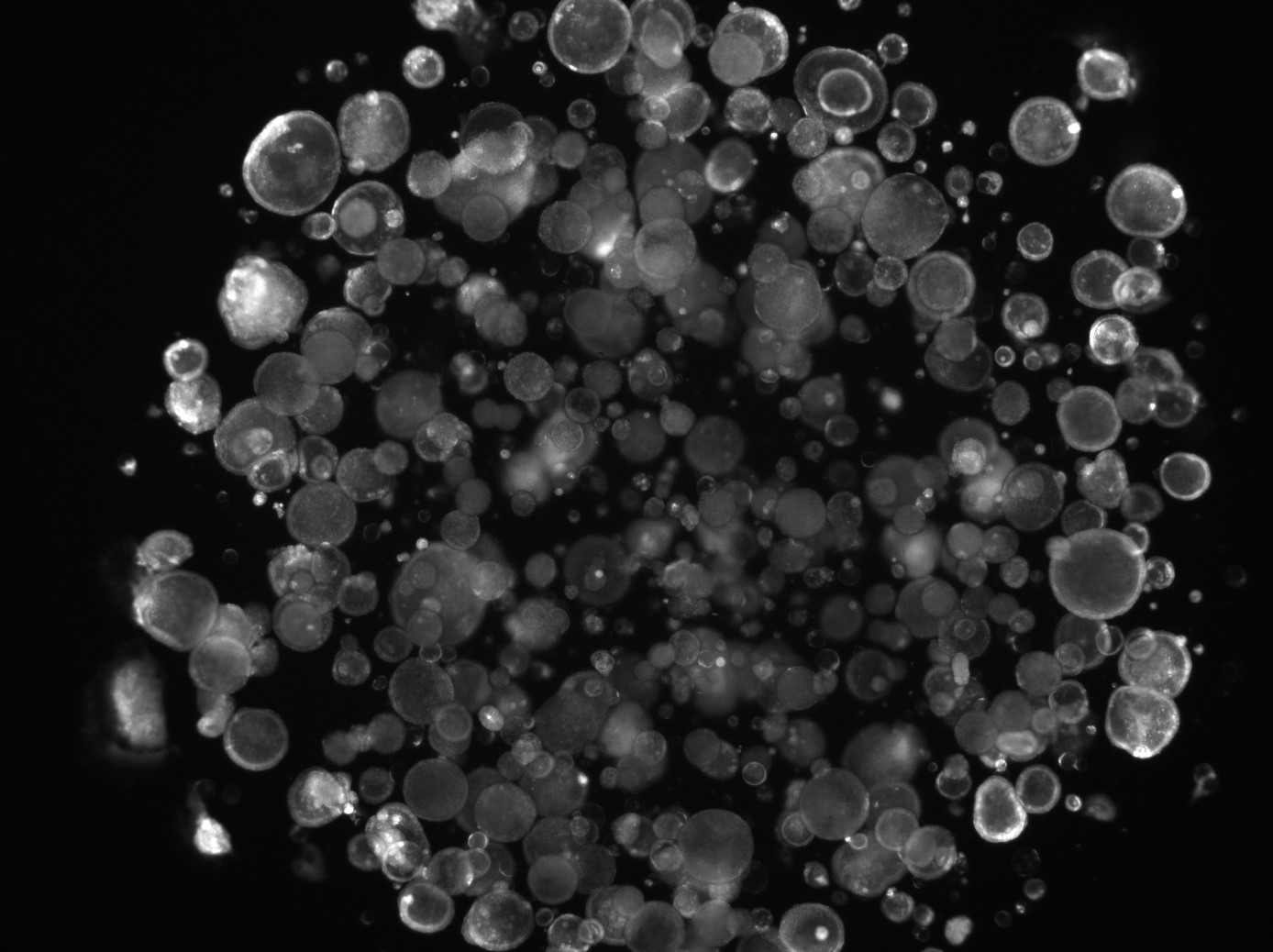

Supplement: Supplementary file 4 — Source Data [file 41467_2024_45605_MOESM4_ESM.zip › Source Data/Figures_Source_Data/figure 1/panel b/B16_TdTomato.jpg]
