# Supplementary material for: Tissue-location-specific transcription programs drive tumor dependencies in colon cancer
Source: Nat Commun. 2024 Feb 15;15:1384. doi: 10.1038/s41467-024-45605-4 (PMC10869357; doi:10.1038/s41467-024-45605-4)
Supplement: Supplementary file 4 — Source Data [file 41467_2024_45605_MOESM4_ESM.zip › Source Data/Figures_Source_Data/supplemental figure 6/panel f/panel f.pdf]

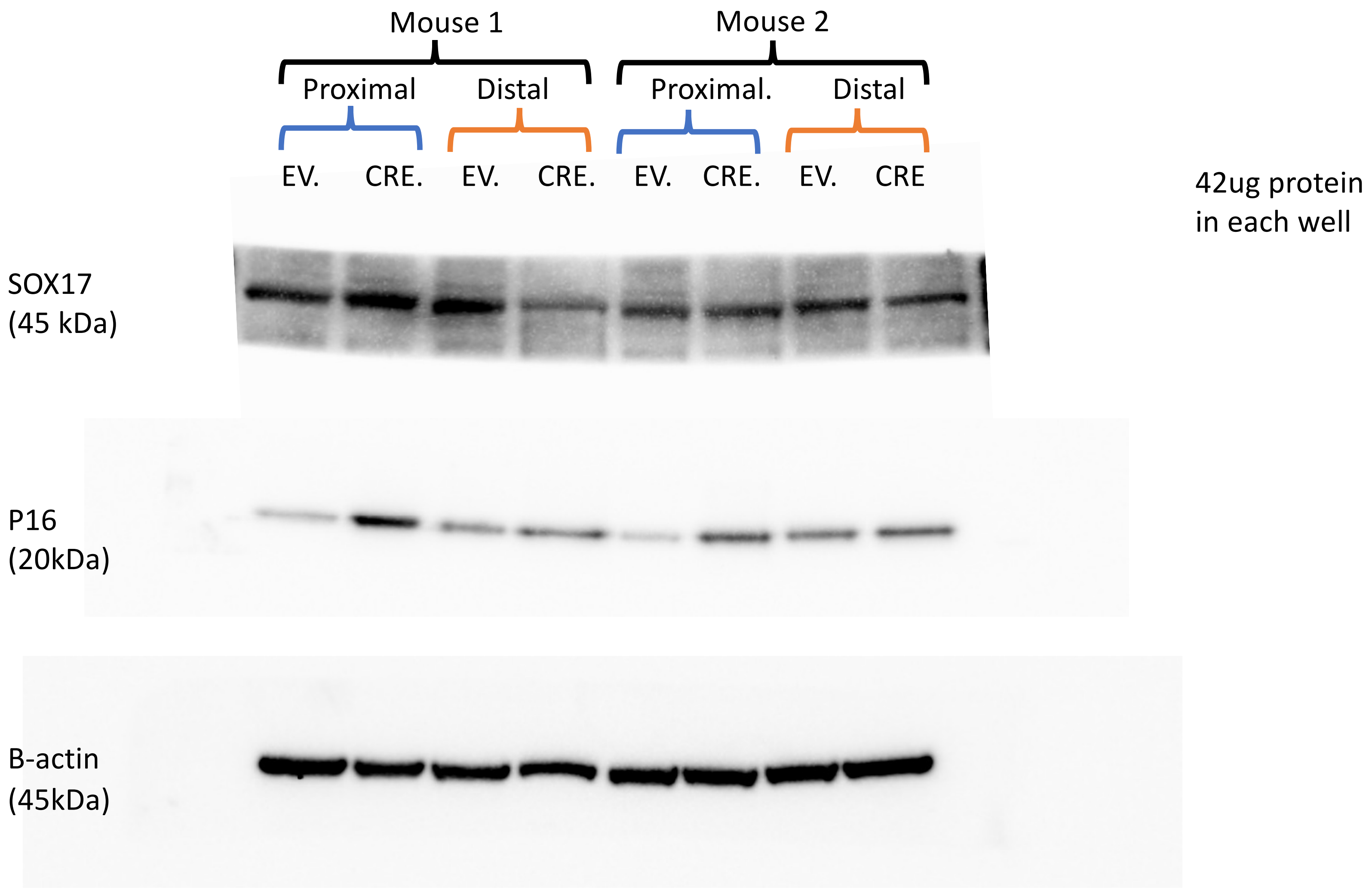
Mouse 1
Mouse 2
Proximal
Distal
Proximal.
Distal
EV.
CRE.
EV.
CRE.
EV.
CRE.
EV.
CRE
42ug protein
in each well
SOX17
(45 kDa)
P16
(20kDa)
B-actin
(45kDa)

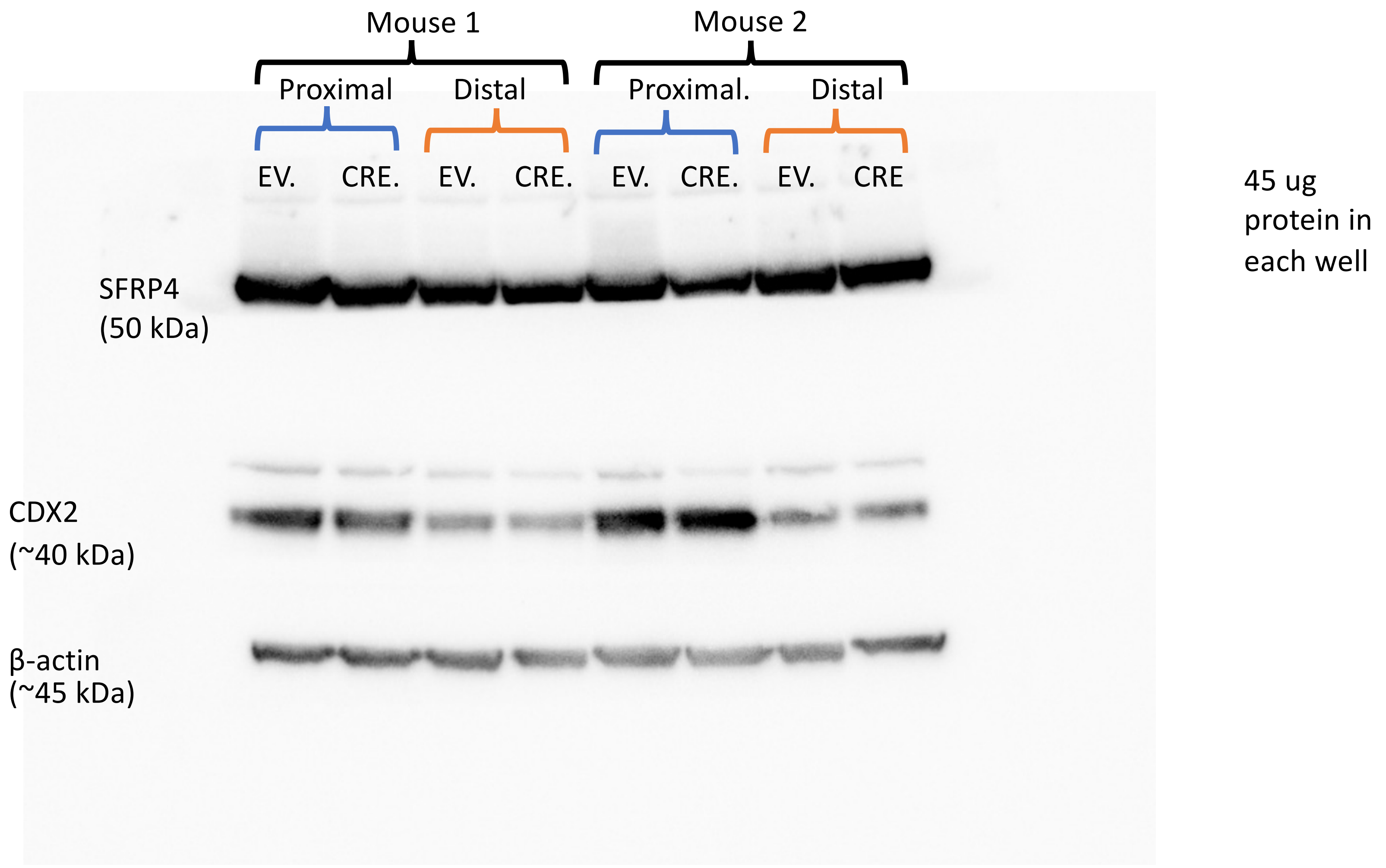
Mouse 1
Mouse 2
Proximal
Distal
Proximal.
Distal
EV.
CRE.
EV.
CRE.
EV.
CRE.
EV.
CRE
SFRP4
(50 kDa)
CDX2
(~40 kDa)
β-actin
(~45 kDa)
45 ug
protein in
each well
